# Robust induction of functional humoral response by a plant-derived Coronavirus-like particle vaccine candidate for COVID-19

**DOI:** 10.1038/s41541-023-00612-2

**Published:** 2023-02-13

**Authors:** Paulina Kaplonek, Deniz Cizmeci, Jessica Shih-Lu Lee, Sally A. Shin, Stephanie Fischinger, Philipe Gobeil, Stéphane Pillet, Nathalie Charland, Brian J. Ward, Galit Alter

**Affiliations:** 1 Ragon Institute of MGH, MIT, and Harvard, Cambridge, MA USA; 2 Medicago Inc., Quebec City, QC Canada; 3 Research Institute of the McGill University Health Centre, Montréal, QC Canada

**Keywords:** Drug development, Viral infection

## Abstract

Despite the success of existing COVID-19 vaccine platforms, the persistent limitations in global deployment of vaccines and waning immunity exhibited by many of the currently deployed vaccine platforms have led to perpetual outbreaks of SARS-CoV-2 variants of concern. Thus, there is an urgent need to develop new durable vaccine candidates, to expand the global vaccine pipeline, and provide safe and effective solutions for every country worldwide. Here we deeply profiled the functional humoral response induced by two doses of AS03-adjuvanted and non-adjuvanted plant-derived Coronavirus-like particle (CoVLP) vaccine candidate from the phase 1 clinical trial, at peak immunogenicity and six months post-vaccination. AS03-adjuvanted CoVLP induced robust and durable SARS-CoV-2 specific humoral immunity, marked by strong IgG1antibody responses, potent FcγR binding, and antibody effector function. Contrary to a decline in neutralizing antibody titers, the FcγR2A-receptor binding capacity and antibody-mediated effector functions, such as opsonophagocytosis, remained readily detectable for at least six months.

## Introduction

SARS-CoV-2, the causative agent of Coronavirus Disease-19 (COVID-19), has infected nearly 500 million people globally and caused more than 5 million deaths^1,2^. The spread, unpredictable nature of disease severity, and limitations in therapeutics have collectively driven the need for vaccines as an essential tool to fight this virus^3^. To address this need, we have experienced remarkable progress in vaccine discovery, with the emergence of several new vaccine platforms, including nucleic acid and vector-based vaccines^4^. Despite the success of these novel technologies, the global availability of vaccines, the emergence of variants of concern, and waning immunity continue to leave large segments of the global population vulnerable to COVID-19. Thus, alternate vaccines that can be deployed globally are still needed to promote immunity and inform booster strategies.

While neutralizing antibodies have been proposed as the key correlate of immunity against SARS-CoV-2^5–8^, accumulating data point to an even stronger association between binding antibodies and vaccine efficacy across diverse vaccine platforms^9–11^. Moreover, while vaccine-induced neutralizing antibodies wane rapidly over time^12,13^, binding antibodies appear to be more durable^14^, suggesting that non-neutralizing antibody functions may play an important role in long-term protection against disease. Furthermore, with the emergence of variants capable of evading neutralizing antibodies^15–17^, the protection against severe illness has been relatively preserved despite the loss of neutralization, arguing again that other vaccine-induced immune mechanisms likely contribute to longer-term protection. Along these lines, emerging data point to Fc-effector function as a correlate of immunity in survival of severe COVID-19^18,19^, to a critical role for Fc-effector function the monoclonal therapeutic resolution of infection in animal models^20^, as well as in vaccine-mediated protection in non-human primates^20,21^. Thus, while high neutralizing antibody titers can be sufficient for protection and may be essential for blocking transmission, additional functions of vaccine-induced binding antibodies that persist over time and potentially bind to VOCs may contribute to the longer-term attenuation of disease.

The plant-made Coronavirus-like particle (CoVLP) vaccine, recently licensed in Canada, by Medicago Inc, shows an efficacy in preventing COVID-19 caused by a different VOCs, ranging from 69.5% against symptomatic infection to 78.8% against moderate-to-severe disease^22^. The vaccine displays trimers of a recombinant spike (S) glycoprotein of SARS-CoV-2 (strain hCoV-19/USA/CA2/2020) embedded in a lipid bilayer 100–150 nm in diameter^23^. These VLPs form spontaneously in the leaf cells of *Nicotiana benthamiana* following transient transfection with *Agrobacterium tumifaciens* and closely resemble the size and structure of SARS-CoV-2 and trigger robust cellular and humoral responses^23,24^. Given our accumulating understanding of the importance of the size, stoichiometry, shape, and arrangement of vaccine antigens in driving optimized vaccine responses^25–28^, the delivery of spike in a viral-like conformation likely helps to promote these responses. However, whether the CoVLP vaccine induces durable and functional non-neutralizing antibodies or can be augmented functionally with an oil-in-water emulsion Adjuvant System 03 (AS03)^29–31^ remains unclear. Thus, here we applied systems serology to samples acquired during a Phase1 trial, where volunteers were randomized to receive two doses of CoVLP at three dose levels (3.75 μg, 7.5 μg, or 15 μg) 21 days apart, with or without Adjuvant System 03 (AS03: GlaxoSmithKline)^23^. SARS-CoV-2 WT and VOC-specific antibody isotype/subclass titers, Fc-receptor (FcγR) binding profiles, and Fc-functional activity were all assessed at peak immunogenicity (Day 42 or 21 days after the second dose) as well as at six months (Day 201 (D201)) post-peak immunogenicity. The CoVLP induced cellular immunity consistent with previous studies of T cell responses to plant-derived influenza vaccine candidates^24,32^. While, the AS03 adjuvant had a minimal impact on promoting cellular responses^23^, the adjuvant markedly improved SARS-CoV-2 specific humoral immunity both in terms of neutralizing antibodies and non-neutralizing, binding antibodies. However, the AS03 had relatively little impact on cellular responses^23^ but markedly improved SARS-CoV-2 specific humoral immune responses both in terms of neutralizing antibodies and non-neutralizing, binding antibodies. The latter responses were characterized by robust FcγR binding and antibody effector function. Some of the non-neutralizing antibody effector functions declined over time, parallel to a loss of both overall and neutralizing antibody levels. However, FcγR2A binding antibody with high opsonophagocytosis activity remained detectable in vaccinees over six months, pointing to the persistence of antibody effector functions following CoVLP-AS03 vaccination that may contribute to long-term protection against COVID-19.

## Results

### AS03-adjuvanted CoVLP triggers robust antibody response to wild-type SARS-CoV-2 antigens across different vaccine doses

Given the global rise of VOCs that exhibit variable neutralization resistance^15–17^, a deeper understanding of functional humoral immunity, mainly using vaccines that can be deployed without special storage or administration requirements, is urgently needed to inform future vaccine development and boosting strategies for the globe.

**Fig. 1:**
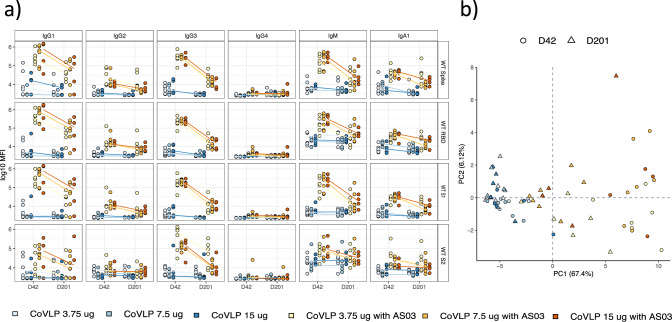
AS03-adjuvanted CoVLP triggers robust antibody response to wild-type SARS-CoV-2 antigens across different vaccine doses. **a** The dot plots show the univariate comparisons of WT Spike-specific IgG1, IgG2, IgG3, IgG4, IgM, and IgA across the vaccinees that received different doses (3.75, 7.5, and 15 µg) of CoVLP without AS03 (shades of blue, *n* = 16) and CoVLP with AS03 (shades of yellow, *n* = 1*7*) and at days 42 and 201. Lines connect the median values of groups. **b** A principal component analysis (PCA) was applied to all samples and data to examine the impact of vaccine dose with and without AS03 adjuvant (different shades of blue and orange) and time since vaccination (day 42 in circles and day 201 in triangles).

Thus, here using Systems Serology we aimed to deeply profile the vaccine-induced humoral response induced by the Medicago coronavirus-like particles (CoVLP). SARS-CoV-2 specific antibody isotype/subclass titers, Fcγ-receptor (FcγR) binding profiles, and antibody effector functions were interrogated at peak immunogenicity (day 42) and six months after receiving the second dose (day 201) in a group of vaccinees (Table 1) who received different doses of CoVLP (3.75, 7.5, and 15 µg) with and without the AS03 adjuvant (*n* = 17 and *n* = 16, respectively). At peak immunogenicity (day 42), the adjuvanted CoVLP-induced cross-isotype responses across all vaccinees to the SARS-CoV-2 wildtype (WT) antigens, such as spike (S), receptor-binding domain (RBD), S1 and S2 domains of Spike protein (Fig. 1a, Supplementary Table 1), marked by minimal differences across the vaccine doses. Previous COVID-19 vaccines have induced robust IgG and IgA levels against SARS-CoV-2 antigens, dominated by IgG1 and IgG3 responses, responsible for FcγR-mediated effector functions^33–35^. Similarly, the addition of the AS03 adjuvant resulted in a highly significant increase of Spike-specific IgG1, IgG3, IgM, and IgA responses, compared to the non-adjuvanted group. Moreover, analysis of antibody profile six months later pointed to a decline in antibody titers across both groups, however, the IgG1 response remained detectable, with no discernable dose-effect observed on day 201. As limited differences were observed across the doses at a univariate level, we used a principal component analysis (PCA) to further determine whether a multivariate difference existed across the groups (Fig. 1b). Adjuvanted profiles clearly separated from non-adjuvanted antibody profiles (PC1 = 67.4% of the maximum variation in the dataset). Interestingly, different vaccine doses were largely indistinguishable in both groups.

**Table 1:** Characteristics of vaccinated participants.

	CoVLP + AS03 *n* = *17*	CoVLP *n* = *16*
Age (median, years)	34	33.5
Sex at birth (female)	11	9
Race, No.		
White	17	15
Black	0	1
Asian	0	0
Multi-racial	0	0
Ethnicity, No.		
Hispanic or Latino	16	16
Body mass index (BMI)	22.8	23.2

Participants were part of phase 1 randomized controlled trial of CoVLP vaccine candidate.

### The addition of AS03 adjuvant significantly increased the magnitude and Fcγ-receptor binding properties of the antibody immune response to CoVLP

**Fig. 2:**
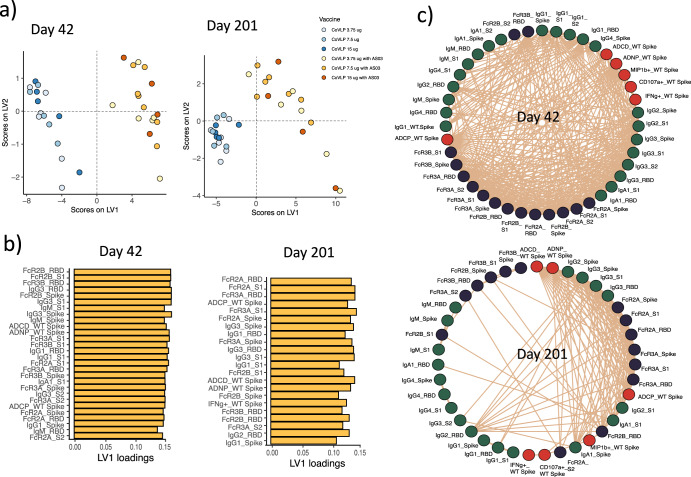
CoVLP adjuvanted with AS03 triggers robust antibody response to various wild-type SARS-CoV-2 antigens. **a**, **b** A Least Absolute Shrinkage Selection Operator (LASSO) was used to reduce the feature dimensionality and ultimately select antibody features that discriminated between vaccinees that received the CoVLP with AS03 (yellow, *n* = 17) and CoVLP without AS03 (blue, *n* = 16). **a** The partial least square discriminant analysis (PLS-DA) was then used to visualize the separation between the samples based on the LASSO selected features, where each dot represents an individual vaccine. **b** The bar graph shows the ranking of the LASSO selected features based on a Variable of Importance (VIP) score. **c** The network analysis shows the additional antibody features that were correlated with the LASSO selected features, thus likely to be as important in driving the separation. The network was built using a threshold of absolute Spearman rho <0.7 and a BH-adjusted *p*-value lower than 0.01. Nodes were colored based on the type of measurement: antibody titers (green), FcRs binding (black), and antibody-mediated effector functions (red). The connecting lines denote all positive correlations (no negative correlations were observed).

To gain a deeper appreciation for the overall humoral immune response induced across the vaccinated population, SARS-CoV-2 specific isotype, subclass, FcγR binding, and functional responses were all integrated. Like single doses response, addition of the AS03 adjuvant induced a wholistically more robust humoral immune response at day 42 (Fig. S1). While the response at day 201 clearly contracted in both the adjuvanted and non-adjuvanted arm, responses remained higher in the adjuvanted group across the isotype/subclasses, the FcγR binding levels, and the antibody effector functions. Moreover, to define the specific humoral differences across the adjuvanted and non-adjuvanted arm at each time point, we used a least absolute shrinkage and selection operator (LASSO) to identify the minimal antibody profile differences across the arms and then utilized a partial least square discriminant analysis (PLS-DA) to visualize if the profiles were distinct from one another. Striking differences were observed in the CoVLP-induced humoral immune response programmed with and without AS03 (Fig. 2a). At peak immunogenicity (day 42), all discriminant features were enriched in the adjuvanted group, marked by the highly expanded receptor-binding domain (RBD) and S1-specific FcγR binding antibodies, S1 and Spike isotype/subclass levels, enhanced Spike-specific complement depositing antibodies, neutrophils, and monocytes phagocytosis-inducing antibodies, and even some S2-specific IgG3 and FcγR binding levels. Similar features were enriched in CoVLP+AS03 group at day 201 (Fig. 2b). Moreover, closer inspection of the overall correlational structure of the humoral immune response pointed to highly correlated immunity across both groups (Fig. 2c). The network analysis on day 42 shows the remarkably strong correlation between nearly all antibody, FcγR-binding and effector functions features across multiple antigenic targets. However, with waning immunity, several relationships were lost over time at day 201, albeit a strong correlation persisted for SARS-CoV-2 specific phagocytic antibodies, binding to the opsonophagocytic FcγR2a and FcγR2b receptors, and highly functional IgG3 levels at day 201, pointing to the persistence of a specific subpopulation of antibodies that may be induced by CoVLP+AS03 that may lead to durable functional immunity following vaccination. In addition, an overall stronger and broader correlation was observed in the adjuvanted population at day 42 (Fig. S2A). On day 201, the antibody profiles remained significantly divergent across the adjuvanted and non-adjuvanted groups (Fig. S2B). Again, all the discriminating features that resolved the two vaccine groups were enriched among the volunteers in the adjuvanted vaccine groups and were marked by robust RBD- and S1- FcγR binding, Spike-monocyte phagocytosis, complement deposition, and neutrophil activation. Moreover, after six months, the overall architecture of the humoral immune response was less highly correlated but maintained a robust correlation between FcγR and antibody effector functions in the adjuvanted group. Thus collectively, while the CoVLP induced a humoral immune response, the addition of AS03 significantly increased the magnitude and the quality of the humoral immune response, maintaining enhanced FcγR binding and function for at least six months.

### AS03-adjuvanted CoVLP vaccine triggers antibody response with persistent opsonophagocytic activity

**Fig. 3:**
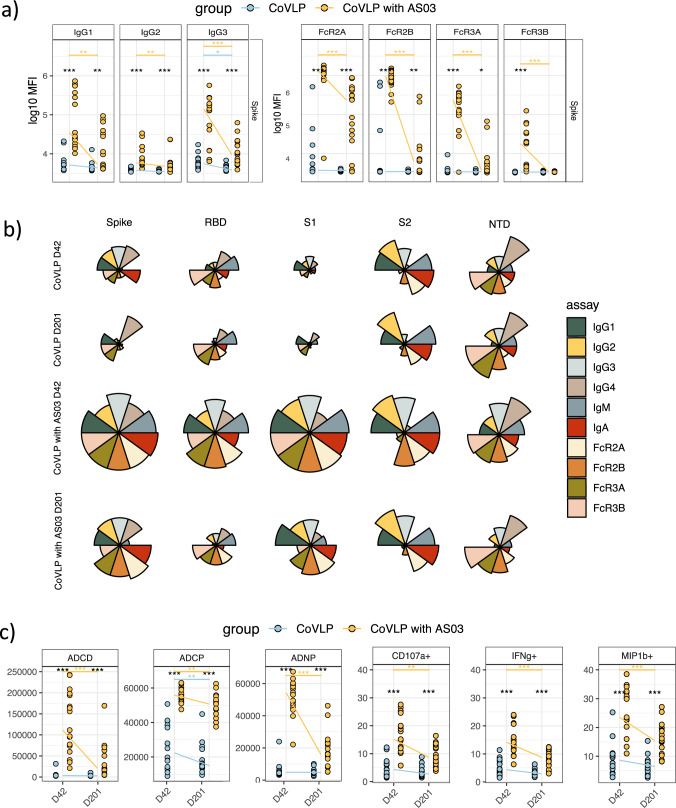
A distinguished humoral profile with a stable Fcγ2A-binding profile and antibody-dependent cellular phagocytosis (ADCP) over the six months differentiate the CoVLP AS03-adjuvanted group. **a** The dot plots show the univariate comparisons of WT Spike-specific IgG1, IgG2, IgG3, as well as FcγR2A-, FcγR2B-, FcγR3A- and FcγR3B-binding levels across the vaccinees that received the CoVLP with AS03 (yellow, *n* = 17) and CoVLP without AS03 (blue, *n* = 16) at days 42 and 201. Lines connect the median values of groups. Differences were defined using a Wilcoxon rank-sum test, and all *p* values were corrected for multiple comparisons using the Benjamini-Hochberg (BH) method, with ***p < 0.001, **p < 0.01, *p < 0.05. **b** The rose plots show the median of mean fluorescent intensity (MFI) values across the recipients of CoVLP with AS03 and CoVLP without AS03 at days 42 and 20 groups. Plots represent the WT SARS-CoV-2 Spike-, RBD-, S1-, S2- and NTD-specific responses. Each wedge represents IgG1, IgG2, IgG3 antibody level, and FcγR2A-, FcγR2B, FcγR3A- and FcγR3B-binding, respectively. The size of the wedge depicts the magnitude of the value. **c** Antibody-dependent complement deposition (ADCD), antibody-dependent cellular phagocytosis (ADCP), antibody-dependent neutrophil phagocytosis (ADNP), and antibody-dependent NK-cells activation (ADNK) measured by a percentage of CD107α positive cells, as well as a level of IFNγ and MIP1β secretion) across CoVLP with AS03 (yellow) and CoVLP without AS03 (blue) vaccinated individuals at days 42 and 201. Mann–Whitney *U*-test corrected for multiple comparisons with the Benjamini-Hochberg (BH) method was used. The adjusted ***p < 0.001, **p < 0.01, *p < 0.05.

To examine the overall changes over time across the humoral immune profiles more clearly, in addition to the analysis of antibody response for separate doses (Fig. 1), we next compared SARS-CoV-2 WT Spike-specific antibody subclass, FcγR-binding, and Fcγ-effector functions at days 42 and 201 with all dose levels combined. Robust Spike-specific IgG1, IgG3, FcγR2A-, FcγR2B-, and FcγR3a- binding were observed at day 42 among the CoVLP-AS03 adjuvanted vaccinees (Fig. 3a). Moreover, analysis of the overall humoral immune response across Spike domains further pointed towards a broadly expanded CoVLP/AS03 S1-specific response and S2-specific IgG1, IgG2, IgG3, FcγR2A, and FcγR2B binding at day 42 (Fig. 3b). Interestingly, the RBD-specific functional antibody response is lost over the time for CoVLP with and without AS03, suggesting that the majority of induced functional antibody responses may be directed outside of the RBD which further highlight the importance of non-neutralizing antibody responses that may play a critical part in response to VOCs. Strikingly, while most of these responses decreased on day 201, FcγR2A-binding levels remained robustly detectable across whole Spike, S1, and S2-domains, suggesting that particular FcγR2A dependent functions, such as phagocytosis, may persist for a prolonged period of time.

Thus, we next compared whether Fc-effector functions were selectively preserved over time. Significant differences were noted across unadjuvanted and adjuvanted CoVLP antibody profiles, with significantly higher levels of antibody-dependent complement deposition (ADCD), antibody-dependent cellular phagocytosis (ADCP), antibody-dependent neutrophil phagocytosis (ADNP), and antibody-dependent NK cell activation (ADNK) in CoVLP+AS03 immunized individuals (Fig. 3c). Moreover, while some functions, including ADCD and ADNP, declined sharply over the six-month follow-up, all functional responses remained above the limit of detection on day 201. Importantly, ADCP and ADNK activity (measured mainly by MIP-1b secretion) remained relatively high over six months, especially marked by robust ADCP across all CoVLP+AS03 immunized individuals. These data pointed to the persistence of these antibody effector functions that could likely continue to provide rapid opsonophagocytic protection upon exposure. In addition, to define whether differences in functional properties of CoVLP-induced antibodies were mediated by differences in Fc glycosylation, we investigated the Spike-specific antibody glycosylation profiles at day 42 and 201 in group of individuals vaccinated with CoVLP with and without AS03 (Fig. S3). A comparison of the overall representation of major sugar classes revealed significantly lower fucosylation level for CoVLP + AS03 group at day 201 compared to day 42 (Fig. S3A). In addition, the trend towards higher sialylation and bisection was noticed for CoVLP + AS03 group at day 201. These data suggest that vaccination may drive reduced fucosylation in antigen-specific antibodies globally, either directly or via increased bisection^36^, but that the degree of fucosylation may vary in the setting of an adjuvant and over time.

### AS03-adjuvanted CoVLP vaccine induces functional antibody responses across various SARS-CoV-2 VOCs

**Fig. 4:**
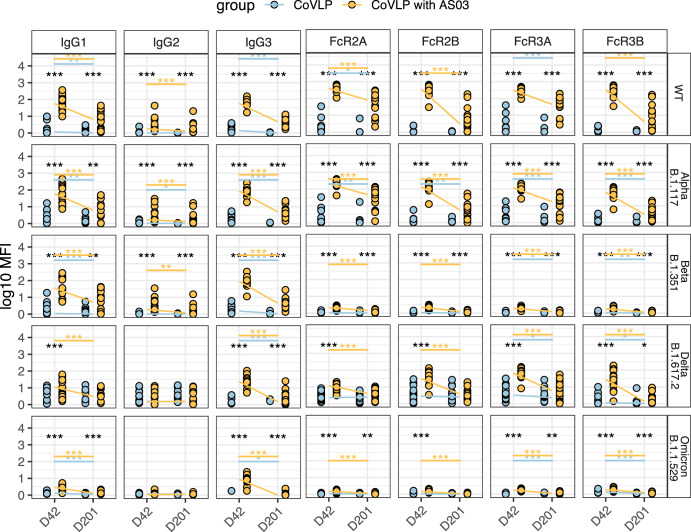
AS03-adjuvanted CoVLP vaccination candidate induces cross-VOC humoral immunity. **a** The dot plots show the univariate comparisons of Alpha B.1.17, Beta B.1.351, Delta B.1.617.2, and Omicron B.1.1.529 SARS-CoV-2 VOCs Spike-specific IgG1, IgG2, IgG3, as well as FcγR2A-, FcγR2B-, FcγR3A- and FcγR3B-binding levels across the vaccinees that received the CoVLP with AS03 (yellow, *n* = 17) and CoVLP without AS03 (blue, *n* = 16) at days 42 and 201. Lines connect the median values of groups. All data were divided by negative control. Differences were defined using a Wilcoxon rank-sum test, and all *p* values were corrected for multiple comparisons using the Benjamini-Hochberg (BH) method, with ***p < 0.001, **p < 0.01, *p < 0.05.

Finally, given the rapid emergence of several variants of concern (VOCs), able to partially evade vaccine-induced neutralization^15,37^, we aimed to determine whether antibody FcγR binding, and functionality may also persist against VOCs. Thus, we analyzed antibody responses and functionality against Alpha (B.1.1.7), Beta (B.1.351), Delta (B.1.617.2), and Omicron (B.1.1.529) S proteins in CoVLP vaccinated individuals (Fig. 4, Fig. S4, and Table S1). Robust IgG1 and IgG3 titers were induced in CoVLP/AS03 immunized volunteers across the Alpha, Beta, and Delta VOCs at day 42, but the antibody level significantly decreased over six months. Importantly, high levels of Spike VOC FcγR2A, FcγR2B, and FcγR3A binding antibodies were detected at day 42 for Alpha, Beta, and Delta in the CoVLP+AS03 immunized individuals. The CoVLP + AS03 vaccine triggers IgG3 antibody response against Omicron SARS-CoV-2 VOC; however, they are not able to bind to FcγR.

Like WT Spike, FcγR2A binding titers at day 201 persisted across all variants tested, pointing to the potential opsonophagocytic protection against several VOCs. Collectively, these data demonstrate the robust induction of SARS-CoV-2 specific immunity using the CoVLP+AS03 vaccine, resulting in the generation of highly functional broad VOC-recognizing humoral immune responses with highly selective and persistent opsonophagocytic functions for up to six months following vaccination.

## Discussion

Among the new COVID-19 vaccines platforms, the Medicago VLPs, produced in *N. benthamian* plants, represents a novel strategy to generate highly immunogenic vaccines economically. While mRNA and vector-based platforms deliver the Spike antigen template that must be further processed (i.e., transcription, expression) by the host cell that the immune system can then detect, CoVLP delivers the “ready-to-use” Spike antigen assembled on nanoparticles. The CoVLPs present antigen in a similar manner to the arrangement that B cells may observe in the setting of infection. With emerging data pointing to a critical role in B-cell receptor clustering as a crucial modulator of the magnitude, durability, and quality of the humoral immune response^38^, this vaccine format has the potential to deliver unique signals required to promote highly functional B cell immune responses. Interestingly, while CoVLP alone induced modest SARS-CoV-2 antibody responses at the doses tested in the Phase 1 trial, the addition of the AS03 adjuvant led to both higher magnitude and more durable humoral responses, most evident in the highly functional opsonophagocytic antibody profile.

VLP-format vaccines have been used commercially for several viral pathogens, including Human Papilloma (HPV)^39^ and Hepatitis B Virus (HBV)^40^. The success of VLPs has been attributed to their ability to present arrayed antigens to the immune system that can trigger B-cell receptor cross-linking and generate strong and durable humoral immune responses^25–28^. While VLPs alone can induce some level of immunity, the addition of powerful adjuvants, such as AS04 in the Cervarix vaccine (Human papillomavirus vaccine)^41^, clearly improved the breadth of the vaccine-induced immune response and reduced the number of doses required to achieve long-lived protection. Moreover, appreciation of the robust protection against SARS-CoV-2 afforded by a distinct RBD-coated nanoparticle coupled to AS03, in non-human primates, linked to more highly functional antibodies, pointed to the promise of combining CoVLP with AS03^42,43^. Here we present the first data for the combination of a SARS-CoV-2 VLP vaccine with AS03 tested in humans, demonstrating robust induction of highly functional and durable antibodies. Moreover, this platform elicited strong and durable FcγR binding antibodies across VOCs and the S1 and S2 domains, likely to be key to eliciting broad immune responses outside of mutational hotspots in the S1 domain required for protection against future variants of concern.

Previous studies highlighted the impact of adjuvants in tuning antibody subclass, FcR-binding, and Fc-effector function^44–46^. Beyond its ability to drive a robust Th1 cytokine profile critical for promoting both innate and adaptive immune responses, AS03 has also been shown to increase antigen uptake by monocytes and promote enhanced antigen presentation to prime T cell immunity, key to both driving higher magnitude, quality, and durability of adaptive immunity^47^. For example, the AS03 adjuvanted H7N9 influenza vaccine showed a superior hemagglutination inhibition titer ratio compared to the MF59 adjuvanted formulation^48^. However, MF59 induced robust opsonophagocytic functions but did not induce antibody-mediated NK cell-activating antibodies in humans^49^. Conversely, in non-human primate studies, a robust opsonophagocytic response was induced by AS03 along with a robust NK cell-activating response^50^. Furthermore, AS03 incorporation into malaria and HIV vaccines drove high antigen-specific antibody levels^51,52^. Finally, both in mice and in vitro AS03 has resulted in a transient increase CCL2, CCL3, IL-6, CSF3, and CXCL1^47^ and AS03 has been shown to improve the activation of naïve B cells or pre-existing memory B cells^11,53^. Thus, AS03 may provide a unique capacity to enhance antibody responses during boosting.

In the current study, we observed similar enhancement of multi-functional antibody profiles induced in the CoVLP and AS03 immunization setting, with robust opsonophagocytic and NK cell-activating antibodies persisting for at least six months, pointing to a possible role for the antigen in shaping antibody functionality or the importance of pre-existing immunity as a potential modulator of antibody effector function. However, given our emerging appreciation for the central importance of opsonophagocytic antibody activity^19^ as a strong predictor of survival of severe disease as well as antibody-dependent cytotoxicity (ADCC) as a correlate of survival following convalescent plasma therapy^54,55^, these data argue that the unusually preserved functional SARS-CoV-2 specific response induced by CoVLP+AS03 may be important in providing more durable protection against SARS-CoV-2 and its variants.

While both convalescent and vaccine-induced antibodies show a consistent decrease in neutralization across VOCs for naturally acquired and vaccine-induced immunity^15,17,56–59^, emerging data point to increased flexibility in Fc-effector functions across VOCs^33,60^. Specifically, Spike-specific antibodies induced by mRNA vaccines^33^ and Coronavac^61^ continue to interact with Fc-receptors and recruit Fc-effector function. Likewise, here we observed robust FcγR binding across VOCs at peak immunogenicity and for at least six months after the second dose. Precisely, FcγR2A binding remained readily detectable in most CoVLP+AS03 immunized individuals against Alpha, Beta, Delta, and Omicron variants. These data suggest that Fc-effector functions persist across SARS-CoV-2 VOCs for extended periods of time. In addition, it has been shown that T cell immunity also plays an important role in the control of SARS-CoV-2 infection, contributing to protection against hospitalization and death^62^ and has been linked to protection against cross- VOCs^63^. Study of CoVLP+AS03 in non-human primates has been shown to induce antigen-specific IL-2 + CD4 T cells and increased numbers of polyfunctional CD4 T cells able to produce IL-2, IFN-γ, and TNF-α^24^. Similarly, balanced T cell responses with significant increases in hemagglutinin-specific polyfunctional CD4 T cells were observed in recipients of the seasonal influenza plant-based quadrivalent VLP vaccine candidate^32^. Because the CoVLP+AS03 vaccine also elicits T cell immunity^24^, that are intrinsically cross-reactive and durable, the persistence of functional antibodies may act as the first line of defense over time, providing an early barrier of protection against VOCs while T cells expand, proliferate, home, and ultimately eliminate the infection.

Because the CoVLP+AS03 vaccine also elicits T cell immunity^24^, that are intrinsically cross-reactive and durable, the persistence of functional antibodies may act as the first line of defense over time, providing an early barrier of protection against VOCs while T cells expand, proliferate, home, and ultimately eliminate the infection.

There are some limitations to our study. First, samples were obtained from the phase 1 clinical trial, that seeks to test the safety of the vaccine; therefore, due to ethical considerations we had access to a relatively small number of subjects that received the CoVLP vaccine with and without AS03 (*n* = 17 and *n* = 16, respectively). Second, only healthy participants between the ages of 18- and 55 years were included in this first study. Thus, we did not profile the immune response in older individuals or individuals with other co-morbidities. In addition, due to limited sample volume, we could not investigate the functional properties of the antibodies toward specific regions of WT SARS-CoV-2 Spike protein and different VOCs. Yet, despite these limitations, this study represents the first comprehensive analysis of the CoVLP-induced antibody profile that may protect SARS-CoV-2 even with a loss of neutralization.”

In summary, cold chain, manufacturing capacity, and lack of the technology transfer have limited the global deployment of several highly effective new-generation COVID-19 vaccines. Here we present the robust induction of highly functional humoral immune response after vaccination with two doses of plant-derived Coronavirus-like particle (CoVLP) vaccine candidate administered with AS03. We demonstrate that the CoVLP-induced antibodies can persist for at least six months and cross-react with several SARS-CoV-2 variants of concern, such as Alpha, Beta, and Delta. Thus, CoVLP+AS03 may be an important additional tool for the on-going fight against SARS-CoV-2.

## Methods

### CoVLP vaccine and adjuvants

The full-length S glycoprotein of SARS-CoV-2, strain hCoV-19/USA/CA2/2020, corresponding in sequence to nucleotides 21,563–25,384 from EPI_ISL_406036 in the GISAID database (https://www.gisaid.org/), was expressed in *N. benthamiana* plants using *A. tumifaciens* transfection, and the downstream purification processes were very similar to those previously described to produce VLPs bearing influenza hemagglutinin proteins. In this system, Spike protein expression is not plasmid-driven per se. Rather, the *Agrobacterium* vector cuts a defined segment of the plasmid and transfers it to the nucleus of the plant cells. This segment remains episomal for some time before being degraded (hence, transient expression) and drives the expression of Spike protein. For the CoVLP vaccine candidate, the Spike protein was modified with R667G, R668S, and R670S substitutions at the S1/S2 cleavage site to increase stability and K971P and V972P substitutions to stabilize the protein in pre-fusion conformation. The signal peptide was replaced with the protein disulfide isomerase from alfalfa, and the transmembrane (TM) domain and cytoplasmic tail (CT) of S protein were replaced with TM/CT from influenza H5 A/Indonesia/5/2005 to increase VLP assembly and budding. Expression of the Spike protein was driven using the double 35 S promoter and proprietary 5′ and 3′ untranslated regions developed to maximize mRNA stability and protein translation. The TBSV P19 suppressor of gene silencing, used under license from Plant Bioscience Limited, is co-expressed to maximize the transient expression of Spike protein. The self-assembled VLPs bearing Spike protein trimers were isolated from the plant matrix and subsequently purified using a process similar to that described for the influenza vaccine candidates. Briefly, *N. benthamiana* plants were grown in a controlled greenhouse environment for approximately five weeks before being exposed to the *A. tumefaciens* transfer vector by vacuum infiltration. After infiltration, plants were placed in a growth chamber under optimal conditions for CoVLP production for up to 6 d. Aerial parts of the plants were then harvested, and the VLPs were released using a proprietary extraction method. The bulk drug substance containing concentrated CoVLPs was then purified using a series of standard industrial filtration and chromatography unit operations steps. The AS03 adjuvant, an oil-in-water emulsion containing DL-α-tocopherol (11.69 mg per dose) and squalene (10.86 mg per dose), was supplied by GlaxoSmithKline.

### Vaccine preparation and injection

The CoVLP vaccine was mixed with AS03 adjuvant, containing DL-α-tocopherol (53.76 mg ml^−1^) and squalene (43.44 mg ml^−1^) directly before injection (CoVLP:AS03 were gently mixed 1:1 volume:volume) and a final dose: 3.75 μg, 7.5 μg or 15 μg of CoVLP + 0.25 ml of AS03 per dose in a volume of 0.50 ml for injection was withdrawn^23^. Each participant received two intramuscular doses in a volume of 0.5 ml 21 days apart (unadjuvanted formulations were administered in a volume of 0.25 ml). The participants and the personnel collecting the safety information and working in testing laboratories remained blinded to treatment allocation. Day 42 (post-second dose), and day 201 (six months after the second dose) serum was processed for immune outcomes as described previously.

### Study design

This phase 1 randomized controlled trial (NCT04450004) was conducted at two sites in Quebec City (Syneos Health Clinique Inc.) and Montreal (Syneos Health Clinique Inc.) as previously described^23^. The study was approved by a central research ethics review board as well as the Health Products and Food Branch of Health Canada and was carried out in accordance with the Declaration of Helsinki and the principles of Good Clinical Practices. Participants were recruited from existing databases of volunteers, and written informed consent was obtained from all study participants. The health status was assessed by medical history, physical examination, and clinical laboratory findings, including detection of anti-N antibodies to SARS-CoV-2 (Elecsys, Roche Diagnostics). Healthy seronegative participants 18–55 years of age were randomized into groups in a 1:1 ratio. Given the relatively small group size and the number of experimental questions being addressed (that is, optimal dose, need for an adjuvant, and best adjuvant), no formal power calculations were performed.

### Luminex profiling

Antibody isotyping and Fcγ-receptor (FcγR) binding were conducted by multiplexed Luminex assay, as previously described^64,65^. Briefly, SARS-CoV-2 antigens were used to profile specific humoral immune responses. Antigens were coupled to magnetic Luminex beads (Luminex Corp) by carbodiimide-NHS ester-coupling (Thermo Fisher). Antigen-coupled microspheres were washed and incubated with plasma samples at an appropriate sample dilution (1:500 for IgG1 and all low-affinity Fcγ- receptors and 1:100 for all other readouts) for 2 h at 37 °C in 384-well plates (Greiner Bio-One). The high-affinity FcR was not tested due to its minimal role in tuning antibody effector function^66^. Unbound antibodies were washed away, and antigen-bound antibodies were detected by using a PE-coupled detection antibody for each subclass and isotype (IgG1, IgG3, IgA1, and IgM; Southern Biotech), and Fcγ-receptors were fluorescently labeled with PE before addition to immune complexes (FcγR2a, FcγR3a; Duke Protein Production facility). After one hour of incubation, plates were washed, and flow cytometry was performed with an IQue (Intellicyt), and analysis was performed on IntelliCyt ForeCyt (v8.1). PE median fluorescent intensity (MFI) is reported as a readout for antigen-specific antibody titers. To ensure assay quality, we detected no reactivity at day 0 in tested subjects across all Luminex-tested antigens and readouts.

### Effector functional assays

Antibody-dependent cellular phagocytosis (ADCP), antibody-dependent neutrophil phagocytosis (ADNP), antibody-dependent complement deposition (ADCD), and antibody-dependent NK activation assays were performed as previously described^67–69^.

SARS-CoV-2 Spike proteins were coupled to yellow/green (505/515) or red/orange (565/580) fluorescent Neutravidin-conjugated beads (Thermo Fisher) for ADCP/ADNP and ADCD, respectively. Immune complexes were formed by incubating the diluted pooled samples (ADCP and ADNP 1:100 dilution) with the antigen-coupled beads for two h at 37 °C. For ADCP, 1.25 × 10^5^ THP-1 cells/mL were added to the immune complexes and incubated for ~18 h at 37 °C. After the incubation, THP-1 cells were washed and fixed with 4% paraformaldehyde (PFA) (Alfa Aesar). For ADNP, the immune complex were incubated with 5 × 10^5^ cells/ml of RBC-lysed whole blood for one h at 37 °C. After incubation, cells were washed and stained for CD66b+(Biolegend) to identify neutrophils, and then fixed in 4% PFA.

For ADCD, the antigen-coupled beads were incubated with the diluted pooled samples (1:10 dilution) for 2 h at 37 °C to form immune complexes. The immune complexes were washed and lyophilized guinea pig complement (Cedarlane) in gelatin veronal buffer with calcium and magnesium (GBV++) (Boston BioProducts) was added for 30 min (complement was reconstituted according to manufacturer’s instruction). The deposition of complement was detected by fluorescein-conjugated goat IgG fraction to guinea pig Complement C3 (Mpbio).

All the assays were acquired by flow cytometry with iQue (Intelluicyt) and the analysis was performed using IntelliCyt ForeCyt. The phagocytosis score was calculated (% cells positive × Median Fluorescent Intensity of positive cells) for ADCP and ADNP. ADCD was reported as the median of C3 deposition.

For antibody-dependent NK cell degranulation SARS-CoV-2 antigens were coated to 96-well ELISA at the protein concentration of 2 μg/ml, incubated at 37 °C for 2 h and blocked with 5% BSA at 4 °C overnight. NK cells were isolated from whole blood from healthy donors (by negative selection using RosetteSep (STEMCELL), then separated using a ficoll gradient. NK cells were rested overnight in media supplemented with IL-15. Serum samples were diluted at 1:25. After blocking, samples were added to coated plates, and immune complexes were formed for two hours at 37 °C. After 2 h, NK cells were prepared (antiCD107a– phycoerythrin (PE) – Cy5 (BD, 1:40, clone: H4A3), brefeldin A (10 µg/ml) (Sigma), and GolgiStop (BD)), and added to each well. for 5 h at 37 °C. The cells were stained for surface markers using anti-CD56 PE-Cy7 (BD, 1:200, clone: B159), anti-CD16 APC-Cy5 (BD, 1:200, clone: 3G8), and anti-CD3 PacBlue (BD, 1:800, UCHT1) and permeabilized with FIX & PERM Cell Permeabilization Kit (Thermo Fisher). After permeabilization cells were stained for intracellular markers MIP1β (BD, 1:50, clone: D21–1351) and IFNγ (BD, 1:17, clone: B27). The flow cytometry was performed. NK cells were defined as CD3-CD16 + CD56+ and frequencies of degranulated (CD107a+), INFγ+ and MIP1β + NK cells determined on an iQue analyzer (Intellicyt).

### Glycan analysis of spike-specific IgG-Fc

Carboxy magnetic beads (Cytiva) were coated with WT SAR-CoV-2 Spike protein by carbodiimide-NHS ester-coupling method. Spike-specific antibodies were isolated from serum samples by incubating 25 μL of serum with 25 μL of antigen-coupled beads overnight at 4 °C. Excess protein was washed off the beads with NEB buffer. IgG-bound beads were resuspended in digestion buffer containing 1 µL IdeZ (NEB Catalog #P0770S) for 2 h at 37 °C. Supernatants were taken from this IdeZ reaction and glycans were labeled with APTS according to manufacturer specifications in the Glycan Assure Kit (Life Technologies # A38928). Samples were run with a LIZ 600 DNA ladder in Hi-Di formamide and analyzed on a 3500xL genetic analyzer (Applied Biosystems) capillary electrophoresis instrument. Data were analyzed using ThermoFisher Glycan Assure Analysis software; peaks were assigned based on migration of standards of known glycans and peak area was calculated. The measured peak areas per sample were totaled to report a relative frequency of each glycan structure identified as previously described^70^.

### Statistics

Data analysis was performed using R version 4.0.2 (2020-06-22). Comparisons between groups were performed using Mann–Whitney *U*-test test followed by Benjamini Hochberg (BH) correction (*p < 0.05, **p < 0.01, ***p < 0.001, ****p < 0.0001). Antigen responses were compared using Wilcoxon-signed rank test followed by BH.

All Luminex data were log-transformed, and all features were scaled and centered. Dots represent the geometric mean fluorescent intensity (gMFI) value of replicates for each serum sample.

Models were built using a combination of the least absolute shrinkage and selection operator (LASSO) for feature selection and then classification using partial least square discriminant analysis (PLS-DA) with the LASSO-selected features using R package “ropls” version 1.20.0^71^ and “glmnet” version 4.0.2. Model accuracy was assessed using ten-fold cross-validation. For each test fold, LASSO-based feature selection was performed on logistic regression using the training set for that fold. LASSO was repeated 100 times, and features selected at least 90 times out of 100 were identified as selected features. PLS-DA classifier was applied to the training set using the selected features and prediction accuracy was recorded. Selected features were ordered according to their Variable Importance in Projection (VIP) score and the first two latent variables (LVs) of the PLS-DA model were used to visualize the samples. A co-correlate network analysis was carried out to identify features that highly correlate with the LASSO selected features, and thus are potentially equally important for discriminating the vaccination arms. Correlations for the co-correlate network were performed using Spearman method followed by Benjamini-Hochberg multiple correction^52^. The co-correlate network was generated using R package “network” version 1.16.0^53^. All other figures were generated using ggplot2^54^.

### Reporting summary

Further information on research design is available in the Nature Research Reporting Summary linked to this article.

## Supplementary information

Supplementary Material

REPORTING SUMMARY

## Data Availability

All anonymized data collected during the trial and associated with this study can be provided. Request should be directed to galter@mgh.harvard.edu. To gain access, data requestors will need to sign a data access agreement, and access will be granted for non-commercial research purposes only.

## Code availability

No previously unreported custom computer code or algorithm were used to generate results. All code can be provided by request directed to galter@mgh.harvard.edu
