# Supplementary material for: Robust induction of functional humoral response by a plant-derived Coronavirus-like particle vaccine candidate for COVID-19
Source: NPJ Vaccines. 2023 Feb 13;8:13. doi: 10.1038/s41541-023-00612-2 (PMC9924894; doi:10.1038/s41541-023-00612-2)
Supplement: Supplementary file 1 — Supplementary Material [file 41541_2023_612_MOESM1_ESM.pdf]

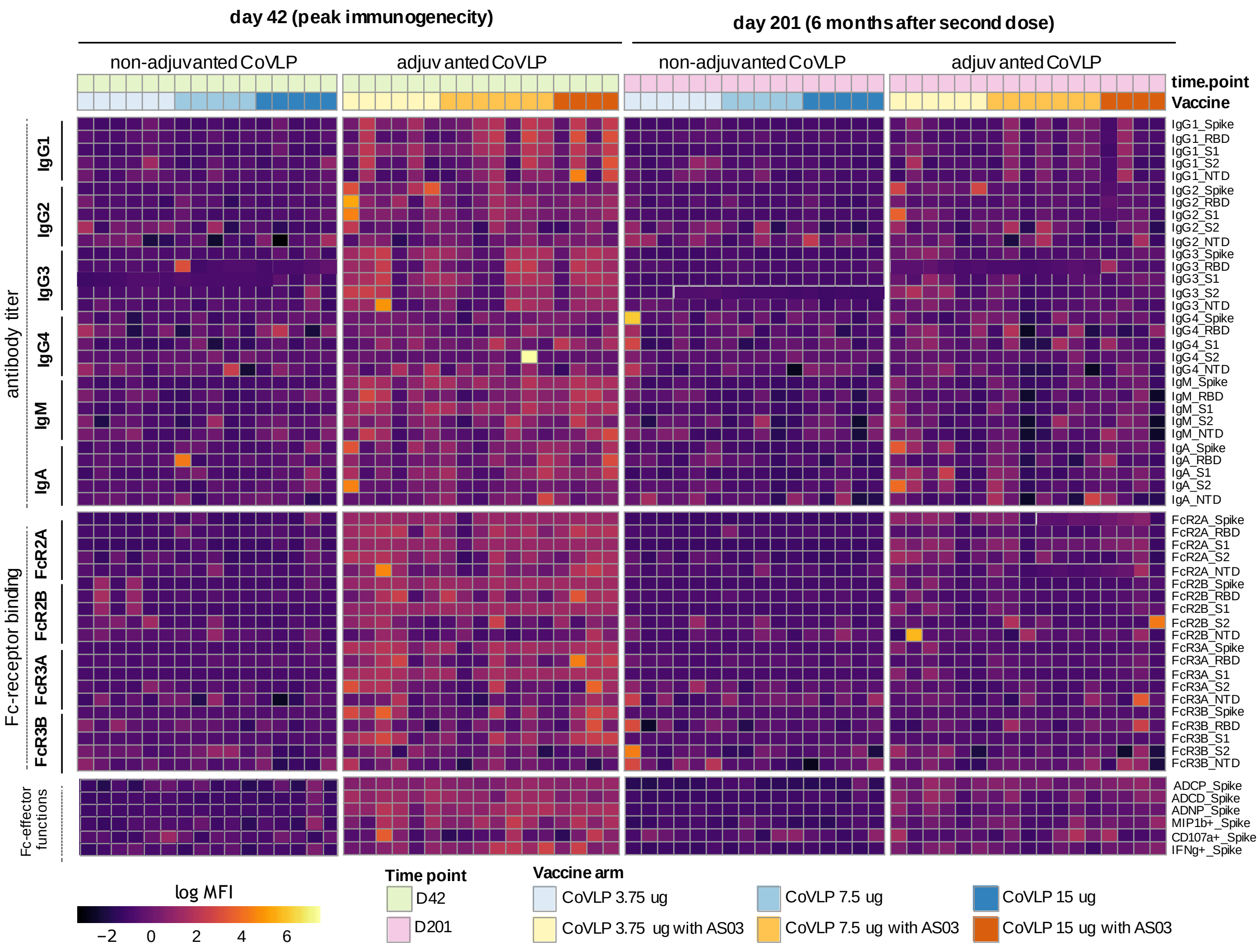

Supplementary Figure 1. AS03 adjuvanted and non-unadjuvanted CoVLP vaccine triggers robust antibody response to multiple wild-type SARS-CoV-2 antigens. The heatmap summarizes the SARS-COV-2 WT -specific IgG1, IgG2, IgG3, IgA1, and IgM titers as well as the ability of the SARS-CoV-2 specific antibodies to bind to the low-affinity Fcγ-receptors (FcγR2A, Fcγ2B, Fcγ3a, and Fcγ3b) across the vaccinees that received different doses of CoVLP with AS03 (orange, $n = 17$) and CoVLP without AS03 (blue, $n = 16$) at day 42 (green) and 201 (pink). Each column represents a different individual. Each row represents a distinct feature that was analyzed.

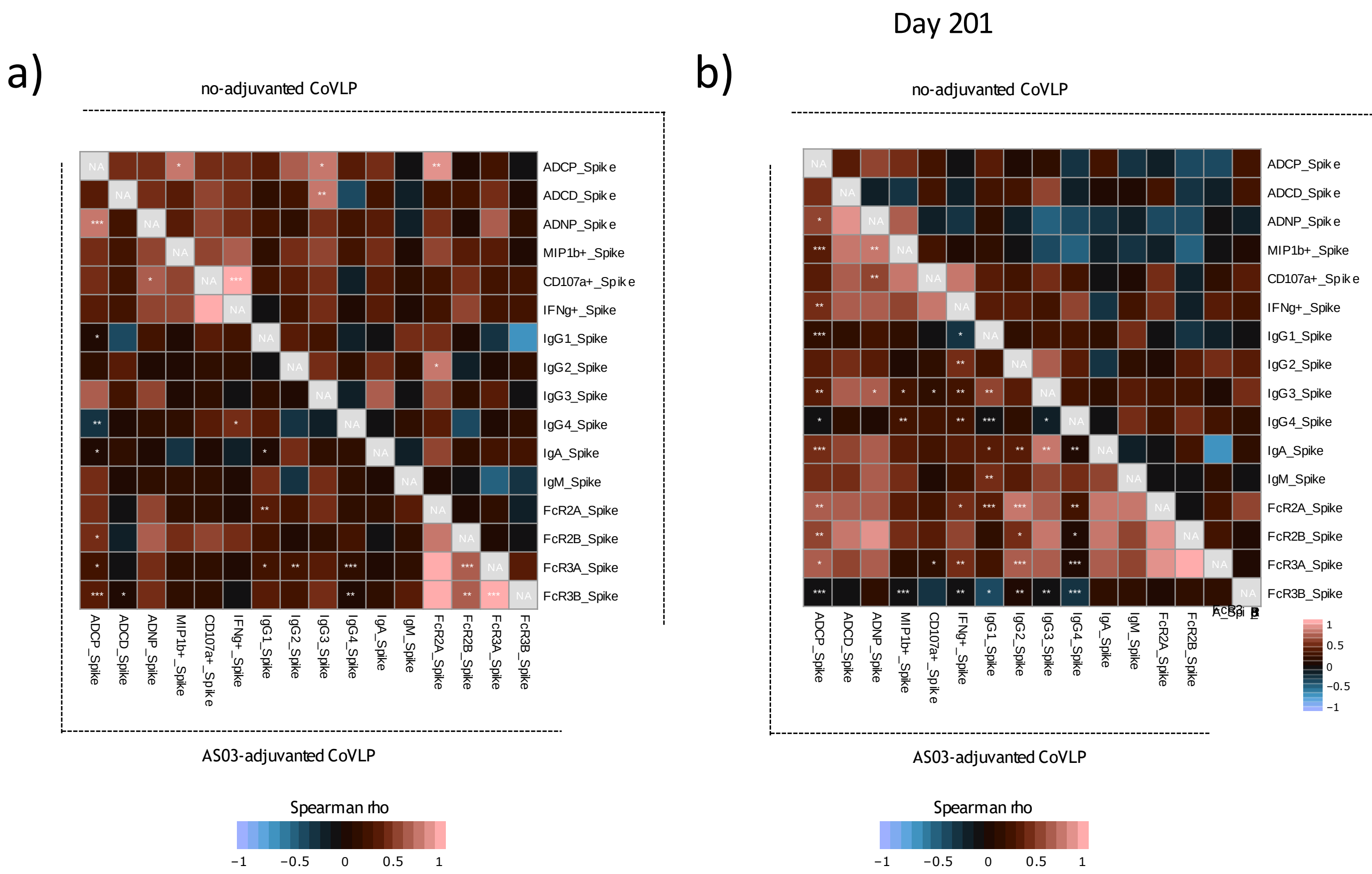

Supplementary Figure 2. Broad relationships between different antibody features were observed in the AS03 adjuvanted CoVLP group. The Spearman correlation heatmap shows relationships across titer, Fcγ-receptors (FcγR2A, Fcγ2B, Fcγ3a, and Fcγ3b) binding and functional properties of WT Spike-specific antibody on day **(A)** 42 and **(B)** 201. The upper triangle represents the group without adjuvant and the lower triangle AS03 adjuvanted arm. Spearman correlations were performed and corrected for multiple comparisons using the Benjamini Hochberg (BH) method. The adjusted p < 0.001 ***, p < 0.01 **, p < 0.05 *.

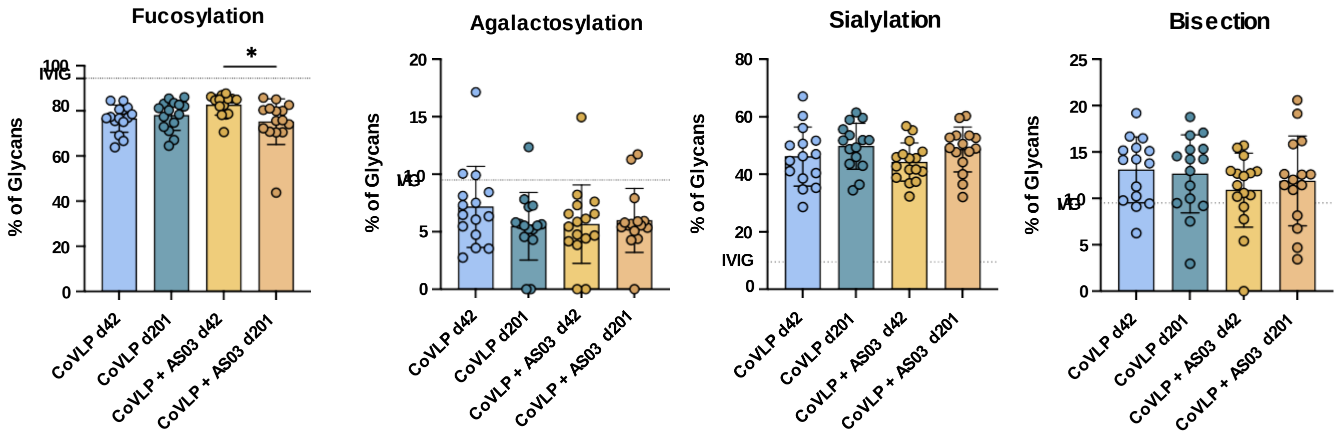

Supplementary Figure 3. The overall fucosylation level of Spike-specific IgG changes over time in AS03 adjuvanted CoVLP group. The percentage of major sugar classes, including fucose, galactose, sialic acid, and a bisecting N-acetylglucosamine, of Spike-specific antibodies in CoVLP with AS03 and CoVLP without AS03 at day 21 and day 201 measured by. Each bar shows the mean ± SD and each dot represents single sample. The dotted line shows the glycosylation level of control IVIG. Differences were defined by one way ANOVA with Tukey's multiple comparison test. The adjusted $p < 0.001$ ***, $p < 0.01$ **, $p < 0.05$ *.

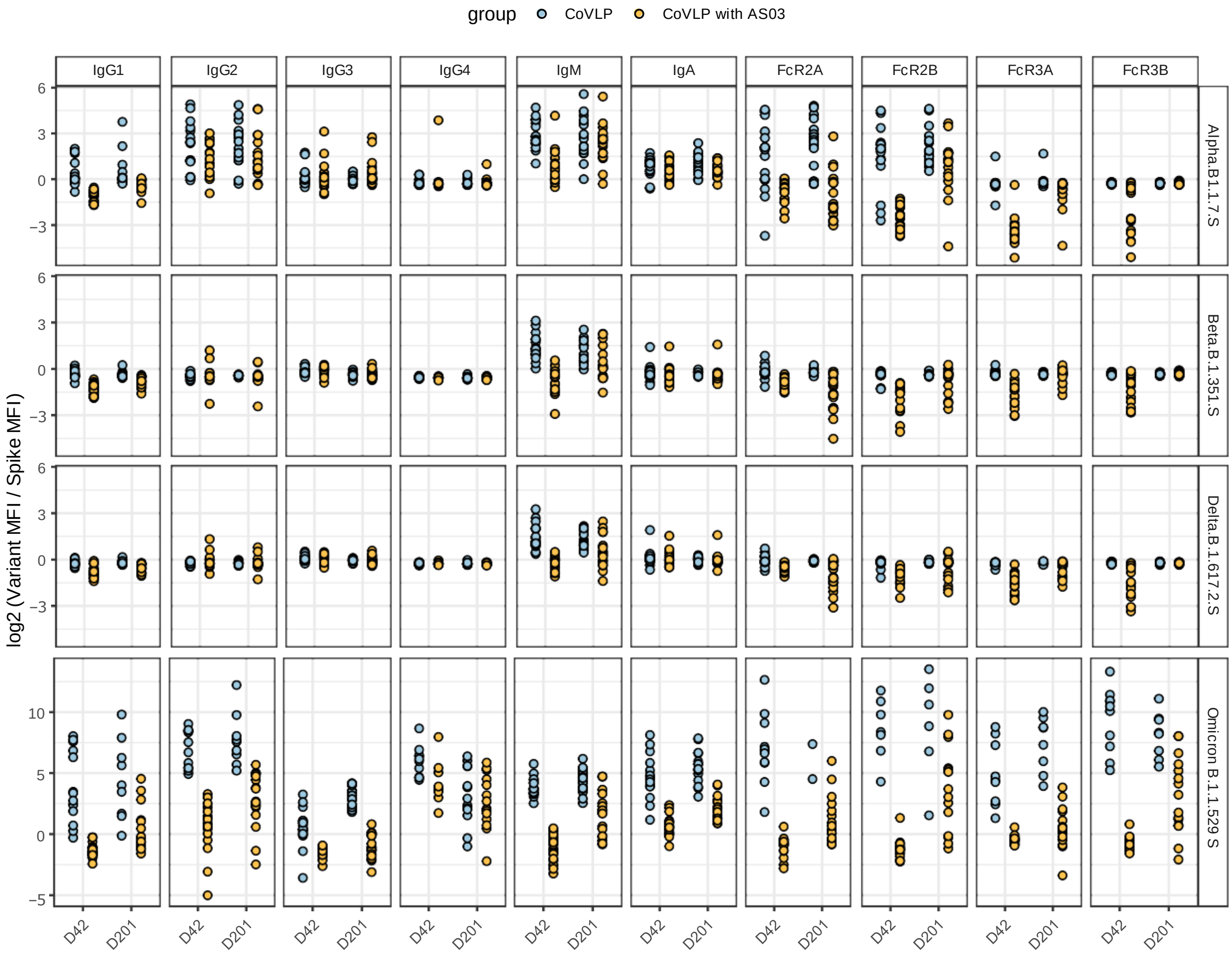

Supplementary Figure 4. Fold-change differences in the cross-VOC response over WT SARS-CoV-2 Spike binding. The dot plots show the univariate comparisons of Alpha B.1.17, Beta B.1.351, Delta B.1.617.2 and Omicron B.1.1.529 SARS-CoV-2 VOCs Spike-specific IgG1, IgG2, IgG3, as well as FcγR2A-, FcγR2B-, FcγR3A- and FcγR3B-binding levels in comparison to WT binding levels. CoVLP with AS03 shown in yellow (n =17) and CoVLP without AS03 shown in blue (n = 16). Data was log2 transformed.

Supplementary Table 1. The statistical summary

feature_group	feature	group	assay	antigen	type	group1	group2	p	p.adj (BH)	log2FC.day42overdayD201
ADCD_Alpha.B1.1.7.S_CoVLP	ADCD_Alpha.B1.1.7.S	CoVLP	ADCD	Alpha.B1.1.7.S	Function	D42	D201	0.0934	0.170318	0.298643297
ADCD_Alpha.B1.1.7.S_CoVLP with AS03	ADCD_Alpha.B1.1.7.S	CoVLP with AS03	ADCD	Alpha.B1.1.7.S	Function	D42	D201	7.63E-05	0.00044	2.652222082
ADCD_Beta.B.1.351.S_CoVLP	ADCD_Beta.B.1.351.S	CoVLP	ADCD	Beta.B.1.351.S	Function	D42	D201	0.348	0.476818	0.154631752
ADCD_Beta.B.1.351.S_CoVLP with AS03	ADCD_Beta.B.1.351.S	CoVLP with AS03	ADCD	Beta.B.1.351.S	Function	D42	D201	0.00385	0.010157	3.915685188
ADCD_Delta.B.1.617.2.S_CoVLP	ADCD_Delta.B.1.617.2.S	CoVLP	ADCD	Delta.B.1.617.2.S	Function	D42	D201	0.000763	0.002426	0.545738994
ADCD_Delta.B.1.617.2.S_CoVLP with AS03	ADCD_Delta.B.1.617.2.S	CoVLP with AS03	ADCD	Delta.B.1.617.2.S	Function	D42	D201	1.53E-05	0.00019	2.07410967
ADCD_Gamma.P1.S_CoVLP	ADCD_Gamma.P1.S	CoVLP	ADCD	Gamma.P1.S	Function	D42	D201	0.00763	0.018371	0.29645571

ADCD_Gamma.P1.S_CoVLP with AS03	ADCD_Gamma.P1.S	CoVLP with AS03	ADCD	Gamma.P1.S	Function	D42	D201	0.000381	0.001432	1.950884219
ADCD_Spike_CoVLP	ADCD_Spike	CoVLP	ADCD	Spike	Function	D42	D201	0.193	0.295457	0.197478387
ADCD_Spike_CoVLP with AS03	ADCD_Spike	CoVLP with AS03	ADCD	Spike	Function	D42	D201	0.000214	0.000915	2.433367192
ADCP_Alpha.B1.1.7.S_CoVLP	ADCP_Alpha.B1.1.7.S	CoVLP	ADCP	Alpha.B1.1.7.S	Function	D42	D201	1	1	-0.068203381
ADCP_Alpha.B1.1.7.S_CoVLP with AS03	ADCP_Alpha.B1.1.7.S	CoVLP with AS03	ADCP	Alpha.B1.1.7.S	Function	D42	D201	0.854	0.893637	-0.011841167
ADCP_Beta.B.1.351.S_CoVLP	ADCP_Beta.B.1.351.S	CoVLP	ADCP	Beta.B.1.351.S	Function	D42	D201	0.744	0.791692	-0.081303866
ADCP_Beta.B.1.351.S_CoVLP with AS03	ADCP_Beta.B.1.351.S	CoVLP with AS03	ADCP	Beta.B.1.351.S	Function	D42	D201	0.000214	0.000915	0.565227008
ADCP_Delta.B.1.617.2.S_CoVLP	ADCP_Delta.B.1.617.2.S	CoVLP	ADCP	Delta.B.1.617.2.S	Function	D42	D201	0.00058	0.001893	0.184166672
ADCP_Delta.B.1.617.2.S_CoVLP with AS03	ADCP_Delta.B.1.617.2.S	CoVLP with AS03	ADCP	Delta.B.1.617.2.S	Function	D42	D201	0.0887	0.166648	0.210373976

ADCP_Gamma.P1.S_CoVLP	ADCP_Gamma.P1.S	CoVLP	ADCP	Gamma.P1.S	Function	D42	D201	0.105	0.187338	0.109652023
ADCP_Gamma.P1.S_CoVLP with AS03	ADCP_Gamma.P1.S	CoVLP with AS03	ADCP	Gamma.P1.S	Function	D42	D201	0.0174	0.039229	0.11399449
ADCP_Spike_CoVLP	ADCP_Spike	CoVLP	ADCP	Spike	Function	D42	D201	0.00418	0.010471	0.5726724
ADCP_Spike_CoVLP with AS03	ADCP_Spike	CoVLP with AS03	ADCP	Spike	Function	D42	D201	0.000839	0.002601	0.137687979
ADNP_Alpha.B1.1.7.S_CoVLP	ADNP_Alpha.B1.1.7.S	CoVLP	ADNP	Alpha.B1.1.7.S	Function	D42	D201	0.94	0.963306	0.356441578
ADNP_Alpha.B1.1.7.S_CoVLP with AS03	ADNP_Alpha.B1.1.7.S	CoVLP with AS03	ADNP	Alpha.B1.1.7.S	Function	D42	D201	7.63E-05	0.00044	0.876913622
ADNP_Beta.B.1.351.S_CoVLP	ADNP_Beta.B.1.351.S	CoVLP	ADNP	Beta.B.1.351.S	Function	D42	D201	0.597	0.701687	-0.089512983
ADNP_Beta.B.1.351.S_CoVLP with AS03	ADNP_Beta.B.1.351.S	CoVLP with AS03	ADNP	Beta.B.1.351.S	Function	D42	D201	1.53E-05	0.00019	2.822801598
ADNP_Delta.B.1.617.2.S_CoVLP	ADNP_Delta.B.1.617.2.S	CoVLP	ADNP	Delta.B.1.617.2.S	Function	D42	D201	0.159	0.2544	0.100964107

ADNP_Delta.B.1.617.2.S_CoVLP with AS03	ADNP_Delta.B.1.617.2.S	CoVLP with AS03	ADNP	Delta.B.1.617.2.S	Function	D42	D201	1.53E-05	0.00019	1.064876799
ADNP_Gamma.P1.S_CoVLP	ADNP_Gamma.P1.S	CoVLP	ADNP	Gamma.P1.S	Function	D42	D201	0.706	0.764398	0.017123984
ADNP_Gamma.P1.S_CoVLP with AS03	ADNP_Gamma.P1.S	CoVLP with AS03	ADNP	Gamma.P1.S	Function	D42	D201	1.53E-05	0.00019	1.246985584
ADNP_Spike_CoVLP	ADNP_Spike	CoVLP	ADNP	Spike	Function	D42	D201	0.597	0.701687	-0.002053755
ADNP_Spike_CoVLP with AS03	ADNP_Spike	CoVLP with AS03	ADNP	Spike	Function	D42	D201	1.53E-05	0.00019	1.758393482
CD107a+_Beta.B.1.351.S_CoVLP	CD107a+_Beta.B.1.351.S	CoVLP	CD107a+	Beta.B.1.351.S	Function	D42	D201	0.597	0.701687	0.103420323
CD107a+_Beta.B.1.351.S_CoVLP with AS03	CD107a+_Beta.B.1.351.S	CoVLP with AS03	CD107a+	Beta.B.1.351.S	Function	D42	D201	0.712	0.764398	0.119666137
CD107a+_Gamma.P1.S_CoVLP	CD107a+_Gamma.P1.S	CoVLP	CD107a+	Gamma.P1.S	Function	D42	D201	0.348	0.476818	-0.121446175
CD107a+_Gamma.P1.S_CoVLP with AS03	CD107a+_Gamma.P1.S	CoVLP with AS03	CD107a+	Gamma.P1.S	Function	D42	D201	0.263	0.395297	0.123197772

CD107a+_Spike_CoVLP	CD107a+_Spike	CoVLP	CD107a+	Spike	Function	D42	D201	0.193	0.295457	0.684154248
CD107a+_Spike_CoVLP with AS03	CD107a+_Spike	CoVLP with AS03	CD107a+	Spike	Function	D42	D201	0.000656	0.002113	0.805559761
FcR2A_Alpha.B1.1.7.S_CoVLP	FcR2A_Alpha.B1.1.7.S	CoVLP	FcR2A	Alpha.B1.1.7.S	FcR	D42	D201	0.433	0.565179	0.056764827
FcR2A_Alpha.B1.1.7.S_CoVLP with AS03	FcR2A_Alpha.B1.1.7.S	CoVLP with AS03	FcR2A	Alpha.B1.1.7.S	FcR	D42	D201	7.63E-05	0.00044	3.93548713
FcR2A_Beta.B.1.351.S_CoVLP	FcR2A_Beta.B.1.351.S	CoVLP	FcR2A	Beta.B.1.351.S	FcR	D42	D201	0.00101	0.003055	0.267063158
FcR2A_Beta.B.1.351.S_CoVLP with AS03	FcR2A_Beta.B.1.351.S	CoVLP with AS03	FcR2A	Beta.B.1.351.S	FcR	D42	D201	3.05E-05	0.000222	5.054797125
FcR2A_Delta.B.1.617.2.S_CoVLP	FcR2A_Delta.B.1.617.2.S	CoVLP	FcR2A	Delta.B.1.617.2.S	FcR	D42	D201	0.0335	0.071009	0.186961158
FcR2A_Delta.B.1.617.2.S_CoVLP with AS03	FcR2A_Delta.B.1.617.2.S	CoVLP with AS03	FcR2A	Delta.B.1.617.2.S	FcR	D42	D201	3.05E-05	0.000222	4.619336244
FcR2A_Gamma.P1.S_CoVLP	FcR2A_Gamma.P1.S	CoVLP	FcR2A	Gamma.P1.S	FcR	D42	D201	0.706	0.764398	-0.003982988

FcR2A_Gamma.P1.S_CoVLP with AS03	FcR2A_Gamma.P1.S	CoVLP with AS03	FcR2A	Gamma.P1.S	FcR	D42	D201	0.0395	0.081633	0.04779554
FcR2A_NTD_CoVLP	FcR2A_NTD	CoVLP	FcR2A	NTD	FcR	D42	D201	0.495	0.616884	0.027313177
FcR2A_NTD_CoVLP with AS03	FcR2A_NTD	CoVLP with AS03	FcR2A	NTD	FcR	D42	D201	0.000107	0.000553	0.671393072
FcR2A_Omicron.B.1.1.529.S_CoVLP	FcR2A_Omicron.B.1.1.529.S	CoVLP	FcR2A	Omicron.B.1.1.529.S	FcR	D42	D201	0.159	0.2544	0.528198316
FcR2A_Omicron.B.1.1.529.S_CoVLP with AS03	FcR2A_Omicron.B.1.1.529.S	CoVLP with AS03	FcR2A	Omicron.B.1.1.529.S	FcR	D42	D201	0.00029	0.001179	1.055031951
FcR2A_RBD_CoVLP	FcR2A_RBD	CoVLP	FcR2A	RBD	FcR	D42	D201	0.528	0.638751	-0.007100659
FcR2A_RBD_CoVLP with AS03	FcR2A_RBD	CoVLP with AS03	FcR2A	RBD	FcR	D42	D201	4.58E-05	0.000316	5.014547681
FcR2A_S1_CoVLP	FcR2A_S1	CoVLP	FcR2A	S1	FcR	D42	D201	0.528	0.638751	0.025083785
FcR2A_S1_CoVLP with AS03	FcR2A_S1	CoVLP with AS03	FcR2A	S1	FcR	D42	D201	3.05E-05	0.000222	3.264229028
FcR2A_S2_CoVLP	FcR2A_S2	CoVLP	FcR2A	S2	FcR	D42	D201	0.298	0.434729	0.187871881
FcR2A_S2_CoVLP with AS03	FcR2A_S2	CoVLP with AS03	FcR2A	S2	FcR	D42	D201	0.00029	0.001179	3.155493716

FcR2A_Spike_CoVLP	FcR2A_Spike	CoVLP	FcR2A	Spike	FcR	D42	D201	0.0507	0.1014	0.030613815
FcR2A_Spike_CoVLP with AS03	FcR2A_Spike	CoVLP with AS03	FcR2A	Spike	FcR	D42	D201	3.05E-05	0.000222	2.506704785
FcR2B_Alpha.B1.1.7.S_CoVLP	FcR2B_Alpha.B1.1.7.S	CoVLP	FcR2B	Alpha.B1.1.7.S	FcR	D42	D201	0.669	0.740679	0.247865039
FcR2B_Alpha.B1.1.7.S_CoVLP with AS03	FcR2B_Alpha.B1.1.7.S	CoVLP with AS03	FcR2B	Alpha.B1.1.7.S	FcR	D42	D201	1.53E-05	0.00019	3.999768677
FcR2B_Beta.B.1.351.S_CoVLP	FcR2B_Beta.B.1.351.S	CoVLP	FcR2B	Beta.B.1.351.S	FcR	D42	D201	0.13	0.222345	0.069670122
FcR2B_Beta.B.1.351.S_CoVLP with AS03	FcR2B_Beta.B.1.351.S	CoVLP with AS03	FcR2B	Beta.B.1.351.S	FcR	D42	D201	1.53E-05	0.00019	6.530503782
FcR2B_Delta.B.1.617.2.S_CoVLP	FcR2B_Delta.B.1.617.2.S	CoVLP	FcR2B	Delta.B.1.617.2.S	FcR	D42	D201	0.495	0.616884	0.022874199
FcR2B_Delta.B.1.617.2.S_CoVLP with AS03	FcR2B_Delta.B.1.617.2.S	CoVLP with AS03	FcR2B	Delta.B.1.617.2.S	FcR	D42	D201	1.53E-05	0.00019	7.168402974

FcR2B_Gamma.P1.S_CoVLP	FcR2B_Gamma.P1.S	CoVLP	FcR2B	Gamma.P1.S	FcR	D42	D201	0.632	0.718972	0.01873027
FcR2B_Gamma.P1.S_CoVLP with AS03	FcR2B_Gamma.P1.S	CoVLP with AS03	FcR2B	Gamma.P1.S	FcR	D42	D201	3.05E-05	0.000222	0.181189279
FcR2B_NTD_CoVLP	FcR2B_NTD	CoVLP	FcR2B	NTD	FcR	D42	D201	0.706	0.764398	-0.005314172
FcR2B_NTD_CoVLP with AS03	FcR2B_NTD	CoVLP with AS03	FcR2B	NTD	FcR	D42	D201	0.747	0.791692	0.003882353
FcR2B_Omicron.B.1.1.529.S_CoVLP	FcR2B_Omicron.B.1.1.529.S	CoVLP	FcR2B	Omicron.B.1.1.529.S	FcR	D42	D201	0.49	0.616853	-0.10462473
FcR2B_Omicron.B.1.1.529.S_CoVLP with AS03	FcR2B_Omicron.B.1.1.529.S	CoVLP with AS03	FcR2B	Omicron.B.1.1.529.S	FcR	D42	D201	1.53E-05	0.00019	1.92229289
FcR2B_RBD_CoVLP	FcR2B_RBD	CoVLP	FcR2B	RBD	FcR	D42	D201	0.669	0.740679	0.033659506
FcR2B_RBD_CoVLP with AS03	FcR2B_RBD	CoVLP with AS03	FcR2B	RBD	FcR	D42	D201	3.05E-05	0.000222	1.446736505
FcR2B_S1_CoVLP	FcR2B_S1	CoVLP	FcR2B	S1	FcR	D42	D201	1	1	-0.018712974
FcR2B_S1_CoVLP with AS03	FcR2B_S1	CoVLP with AS03	FcR2B	S1	FcR	D42	D201	1.53E-05	0.00019	7.768554512
FcR2B_S2_CoVLP	FcR2B_S2	CoVLP	FcR2B	S2	FcR	D42	D201	0.94	0.963306	0.019756029

FcR2B_S2_CoVLP with AS03	FcR2B_S2	CoVLP with AS03	FcR2B	S2	FcR	D42	D201	0.0638	0.123613	0.854992882
FcR2B_Spike_CoVLP	FcR2B_Spike	CoVLP	FcR2B	Spike	FcR	D42	D201	0.433	0.565179	0.008502268
FcR2B_Spike_CoVLP with AS03	FcR2B_Spike	CoVLP with AS03	FcR2B	Spike	FcR	D42	D201	1.53E-05	0.00019	7.272990238
FcR3A_Alpha.B1.1.7.S_CoVLP	FcR3A_Alpha.B1.1.7.S	CoVLP	FcR3A	Alpha.B1.1.7.S	FcR	D42	D201	0.025	0.053913	-0.104380143
FcR3A_Alpha.B1.1.7.S_CoVLP with AS03	FcR3A_Alpha.B1.1.7.S	CoVLP with AS03	FcR3A	Alpha.B1.1.7.S	FcR	D42	D201	3.05E-05	0.000222	2.974395405
FcR3A_Beta.B.1.351.S_CoVLP	FcR3A_Beta.B.1.351.S	CoVLP	FcR3A	Beta.B.1.351.S	FcR	D42	D201	0.94	0.963306	-0.05556843
FcR3A_Beta.B.1.351.S_CoVLP with AS03	FcR3A_Beta.B.1.351.S	CoVLP with AS03	FcR3A	Beta.B.1.351.S	FcR	D42	D201	3.05E-05	0.000222	4.148187799
FcR3A_Delta.B.1.617.2.S_CoVLP	FcR3A_Delta.B.1.617.2.S	CoVLP	FcR3A	Delta.B.1.617.2.S	FcR	D42	D201	0.375	0.508197	-0.021264352
FcR3A_Delta.B.1.617.2.S_CoVLP with AS03	FcR3A_Delta.B.1.617.2.S	CoVLP with AS03	FcR3A	Delta.B.1.617.2.S	FcR	D42	D201	3.05E-05	0.000222	4.620756108

FcR3A_Gamma.P1.S_CoVLP	FcR3A_Gamma.P1.S	CoVLP	FcR3A	Gamma.P1.S	FcR	D42	D201	0.528	0.638751	-0.016263243
FcR3A_Gamma.P1.S_CoVLP with AS03	FcR3A_Gamma.P1.S	CoVLP with AS03	FcR3A	Gamma.P1.S	FcR	D42	D201	0.000381	0.001432	0.308830463
FcR3A_NTD_CoVLP	FcR3A_NTD	CoVLP	FcR3A	NTD	FcR	D42	D201	0.0934	0.170318	-0.061188307
FcR3A_NTD_CoVLP with AS03	FcR3A_NTD	CoVLP with AS03	FcR3A	NTD	FcR	D42	D201	0.487	0.616204	0.01440918
FcR3A_Omicron.B.1.1.529.S_CoVLP	FcR3A_Omicron.B.1.1.529.S	CoVLP	FcR3A	Omicron.B.1.1.529.S	FcR	D42	D201	0.000305	0.001201	1.171845548
FcR3A_Omicron.B.1.1.529.S_CoVLP with AS03	FcR3A_Omicron.B.1.1.529.S	CoVLP with AS03	FcR3A	Omicron.B.1.1.529.S	FcR	D42	D201	1.53E-05	0.00019	1.554293317
FcR3A_RBD_CoVLP	FcR3A_RBD	CoVLP	FcR3A	RBD	FcR	D42	D201	0.528	0.638751	-0.033191786
FcR3A_RBD_CoVLP with AS03	FcR3A_RBD	CoVLP with AS03	FcR3A	RBD	FcR	D42	D201	0.00209	0.005958	0.490570495
FcR3A_S1_CoVLP	FcR3A_S1	CoVLP	FcR3A	S1	FcR	D42	D201	0.433	0.565179	-0.037650402
FcR3A_S1_CoVLP with AS03	FcR3A_S1	CoVLP with AS03	FcR3A	S1	FcR	D42	D201	3.05E-05	0.000222	6.388132052

FcR3A_S2_CoVLP	FcR3A_S2	CoVLP	FcR3A	S2	FcR	D42	D201	0.0155	0.035266	-0.066542392
FcR3A_S2_CoVLP with AS03	FcR3A_S2	CoVLP with AS03	FcR3A	S2	FcR	D42	D201	0.00258	0.007031	0.102824967
FcR3A_Spike_CoVLP	FcR3A_Spike	CoVLP	FcR3A	Spike	FcR	D42	D201	0.632	0.718972	-0.033799446
FcR3A_Spike_CoVLP with AS03	FcR3A_Spike	CoVLP with AS03	FcR3A	Spike	FcR	D42	D201	3.05E-05	0.000222	5.945812613
FcR3B_Alpha.B1.1.7.S_CoVLP	FcR3B_Alpha.B1.1.7.S	CoVLP	FcR3B	Alpha.B1.1.7.S	FcR	D42	D201	0.433	0.565179	-0.010072323
FcR3B_Alpha.B1.1.7.S_CoVLP with AS03	FcR3B_Alpha.B1.1.7.S	CoVLP with AS03	FcR3B	Alpha.B1.1.7.S	FcR	D42	D201	0.000504	0.001689	0.102904073
FcR3B_Beta.B.1.351.S_CoVLP	FcR3B_Beta.B.1.351.S	CoVLP	FcR3B	Beta.B.1.351.S	FcR	D42	D201	0.348	0.476818	0.030789777
FcR3B_Beta.B.1.351.S_CoVLP with AS03	FcR3B_Beta.B.1.351.S	CoVLP with AS03	FcR3B	Beta.B.1.351.S	FcR	D42	D201	4.58E-05	0.000316	0.62064168
FcR3B_Delta.B.1.617.2.S_CoVLP	FcR3B_Delta.B1.617.2.S	CoVLP	FcR3B	Delta.B.1.617.2.S	FcR	D42	D201	0.632	0.718972	-0.009115117

FcR3B_Delta.B.1.617.2.S_CoVLP with AS03	FcR3B_Delta.B.1.617.2.S	CoVLP with AS03	FcR3B	Delta.B.1.617.2.S	FcR	D42	D201	0.000214	0.000915	0.281264764
FcR3B_Gamma.P1.S_CoVLP	FcR3B_Gamma.P1.S	CoVLP	FcR3B	Gamma.P1.S	FcR	D42	D201	0.706	0.764398	0.007722704
FcR3B_Gamma.P1.S_CoVLP with AS03	FcR3B_Gamma.P1.S	CoVLP with AS03	FcR3B	Gamma.P1.S	FcR	D42	D201	0.000107	0.000553	0.359650996
FcR3B_NTD_CoVLP	FcR3B_NTD	CoVLP	FcR3B	NTD	FcR	D42	D201	0.375	0.508197	-0.010336651
FcR3B_NTD_CoVLP with AS03	FcR3B_NTD	CoVLP with AS03	FcR3B	NTD	FcR	D42	D201	0.712	0.764398	-0.011851416
FcR3B_Omicron.B.1.1.529.S_CoVLP	FcR3B_Omicron.B.1.1.529.S	CoVLP	FcR3B	Omicron.B.1.1.529.S	FcR	D42	D201	3.05E-05	0.000222	2.001173362
FcR3B_Omicron.B.1.1.529.S_CoVLP with AS03	FcR3B_Omicron.B.1.1.529.S	CoVLP with AS03	FcR3B	Omicron.B.1.1.529.S	FcR	D42	D201	1.53E-05	0.00019	1.85236287
FcR3B_RBD_CoVLP	FcR3B_RBD	CoVLP	FcR3B	RBD	FcR	D42	D201	0.433	0.565179	0.006020565
FcR3B_RBD_CoVLP with AS03	FcR3B_RBD	CoVLP with AS03	FcR3B	RBD	FcR	D42	D201	0.0887	0.166648	0.020961599
FcR3B_S1_CoVLP	FcR3B_S1	CoVLP	FcR3B	S1	FcR	D42	D201	0.144	0.239733	-0.010643901

FcR3B_S1_CoVLP with AS03	FcR3B_S1	CoVLP with AS03	FcR3B	S1	FcR	D42	D201	3.05E-05	0.000222	3.86583763
FcR3B_S2_CoVLP	FcR3B_S2	CoVLP	FcR3B	S2	FcR	D42	D201	0.433	0.565179	0.014962648
FcR3B_S2_CoVLP with AS03	FcR3B_S2	CoVLP with AS03	FcR3B	S2	FcR	D42	D201	0.306	0.443789	0.012867062
FcR3B_Spike_CoVLP	FcR3B_Spike	CoVLP	FcR3B	Spike	FcR	D42	D201	0.744	0.791692	-0.002027681
FcR3B_Spike_CoVLP with AS03	FcR3B_Spike	CoVLP with AS03	FcR3B	Spike	FcR	D42	D201	7.63E-05	0.00044	2.392762144
IFNg+_Beta.B.1.351.S_CoVLP	IFNg+_Beta.B.1.351.S	CoVLP	IFNg+	Beta.B.1.351.S	Function	D42	D201	0.298	0.434729	-0.104693668
IFNg+_Beta.B.1.351.S_CoVLP with AS03	IFNg+_Beta.B.1.351.S	CoVLP with AS03	IFNg+	Beta.B.1.351.S	Function	D42	D201	0.243	0.367463	0.480743452
IFNg+_Gamma.P1.S_CoVLP	IFNg+_Gamma.P1.S	CoVLP	IFNg+	Gamma.P1.S	Function	D42	D201	0.9	0.933891	-0.757326358
IFNg+_Gamma.P1.S_CoVLP with AS03	IFNg+_Gamma.P1.S	CoVLP with AS03	IFNg+	Gamma.P1.S	Function	D42	D201	0.487	0.616204	0.275551153
IFNg+_Spike_CoVLP	IFNg+_Spike	CoVLP	IFNg+	Spike	Function	D42	D201	0.0577	0.112674	0.596351927

IFNg+_Spike_CoVLP with AS03	IFNg+_Spike	CoVLP with AS03	IFNg+	Spike	Function	D42	D201	0.000107	0.000553	0.688049732
IgA_Alpha.B1.1.7.S_CoVLP	IgA_Alpha.B1.1.7.S	CoVLP	IgA	Alpha.B1.1.7.S	Titer	D42	D201	0.632	0.718972	0.335800045
IgA_Alpha.B1.1.7.S_CoVLP with AS03	IgA_Alpha.B1.1.7.S	CoVLP with AS03	IgA	Alpha.B1.1.7.S	Titer	D42	D201	0.12	0.209577	0.492628585
IgA_Beta.B.1.351.S_CoVLP	IgA_Beta.B.1.351.S	CoVLP	IgA	Beta.B.1.351.S	Titer	D42	D201	0.00919	0.0213	0.262158566
IgA_Beta.B.1.351.S_CoVLP with AS03	IgA_Beta.B.1.351.S	CoVLP with AS03	IgA	Beta.B.1.351.S	Titer	D42	D201	0.145	0.239733	0.680243556
IgA_Delta.B.1.617.2.S_CoVLP	IgA_Delta.B.1.617.2.S	CoVLP	IgA	Delta.B.1.617.2.S	Titer	D42	D201	0.0182	0.0403	0.271802209
IgA_Delta.B.1.617.2.S_CoVLP with AS03	IgA_Delta.B.1.617.2.S	CoVLP with AS03	IgA	Delta.B.1.617.2.S	Titer	D42	D201	0.145	0.239733	0.737967489
IgA_Gamma.P1.S_CoVLP	IgA_Gamma.P1.S	CoVLP	IgA	Gamma.P1.S	Titer	D42	D201	0.00418	0.010471	0.258930594
IgA_Gamma.P1.S_CoVLP with AS03	IgA_Gamma.P1.S	CoVLP with AS03	IgA	Gamma.P1.S	Titer	D42	D201	0.015	0.034444	1.012389351

IgA_NTD_CoVLP	IgA_NTD	CoVLP	IgA	NTD	Titer	D42	D201	0.669	0.740679	-0.026267233
IgA_NTD_CoVLP with AS03	IgA_NTD	CoVLP with AS03	IgA	NTD	Titer	D42	D201	0.0714	0.137265	0.076307403
IgA_Omicron.B.1.1.529.S_CoVLP	IgA_Omicron.B.1.1.529.S	CoVLP	IgA	Omicron.B.1.1.529.S	Titer	D42	D201	0.00131	0.003868	0.358343467
IgA_Omicron.B.1.1.529.S_CoVLP with AS03	IgA_Omicron.B.1.1.529.S	CoVLP with AS03	IgA	Omicron.B.1.1.529.S	Titer	D42	D201	0.000504	0.001689	0.755186141
IgA_RBD_CoVLP	IgA_RBD	CoVLP	IgA	RBD	Titer	D42	D201	0.562	0.673314	0.011396389
IgA_RBD_CoVLP with AS03	IgA_RBD	CoVLP with AS03	IgA	RBD	Titer	D42	D201	0.000839	0.002601	0.297070795
IgA_S1_CoVLP	IgA_S1	CoVLP	IgA	S1	Titer	D42	D201	0.00336	0.00896	0.169108106
IgA_S1_CoVLP with AS03	IgA_S1	CoVLP with AS03	IgA	S1	Titer	D42	D201	0.0569	0.112674	1.013982026
IgA_S2_CoVLP	IgA_S2	CoVLP	IgA	S2	Titer	D42	D201	0.669	0.740679	0.088272405
IgA_S2_CoVLP with AS03	IgA_S2	CoVLP with AS03	IgA	S2	Titer	D42	D201	0.459	0.595979	0.553360148
IgA_Spike_CoVLP	IgA_Spike	CoVLP	IgA	Spike	Titer	D42	D201	0.00418	0.010471	0.276056851
IgA_Spike_CoVLP with AS03	IgA_Spike	CoVLP with AS03	IgA	Spike	Titer	D42	D201	0.159	0.2544	0.891988832

IgG1_Alpha.B1.1.7.S_CoVLP	IgG1_Alpha.B1.1.7.S	CoVLP	IgG1	Alpha.B1.1.7.S	Titer	D42	D201	0.348	0.476818	0.209346637
IgG1_Alpha.B1.1.7.S_CoVLP with AS03	IgG1_Alpha.B1.1.7.S	CoVLP with AS03	IgG1	Alpha.B1.1.7.S	Titer	D42	D201	0.000504	0.001689	1.506968767
IgG1_Beta.B.1.351.S_CoVLP	IgG1_Beta.B.1.351.S	CoVLP	IgG1	Beta.B.1.351.S	Titer	D42	D201	0.0934	0.170318	0.212355078
IgG1_Beta.B.1.351.S_CoVLP with AS03	IgG1_Beta.B.1.351.S	CoVLP with AS03	IgG1	Beta.B.1.351.S	Titer	D42	D201	0.000504	0.001689	2.143170286
IgG1_Delta.B.1.617.2.S_CoVLP	IgG1_Delta.B.1.617.2.S	CoVLP	IgG1	Delta.B.1.617.2.S	Titer	D42	D201	0.144	0.239733	0.286278997
IgG1_Delta.B.1.617.2.S_CoVLP with AS03	IgG1_Delta.B.1.617.2.S	CoVLP with AS03	IgG1	Delta.B.1.617.2.S	Titer	D42	D201	0.000504	0.001689	2.593245076
IgG1_Gamma.P1.S_CoVLP	IgG1_Gamma.P1.S	CoVLP	IgG1	Gamma.P1.S	Titer	D42	D201	0.175	0.272956	0.16872621
IgG1_Gamma.P1.S_CoVLP with AS03	IgG1_Gamma.P1.S	CoVLP with AS03	IgG1	Gamma.P1.S	Titer	D42	D201	0.000504	0.001689	1.994064554
IgG1_NTD_CoVLP	IgG1_NTD	CoVLP	IgG1	NTD	Titer	D42	D201	0.348	0.476818	0.041490806

IgG1_NTD_CoVLP with AS03	IgG1_NTD	CoVLP with AS03	IgG1	NTD	Titer	D42	D201	0.0505	0.1014	0.218135425
IgG1_Omicron.B.1.1.529.S_CoVLP	IgG1_Omicron.B.1.1.529.S	CoVLP	IgG1	Omicron.B.1.1.529.S	Titer	D42	D201	0.000153	0.00069	1.761400815
IgG1_Omicron.B.1.1.529.S_CoVLP with AS03	IgG1_Omicron.B.1.1.529.S	CoVLP with AS03	IgG1	Omicron.B.1.1.529.S	Titer	D42	D201	1.53E-05	0.00019	2.842106173
IgG1_RBD_CoVLP	IgG1_RBD	CoVLP	IgG1	RBD	Titer	D42	D201	0.175	0.272956	0.123500835
IgG1_RBD_CoVLP with AS03	IgG1_RBD	CoVLP with AS03	IgG1	RBD	Titer	D42	D201	0.00107	0.003197	0.751528169
IgG1_S1_CoVLP	IgG1_S1	CoVLP	IgG1	S1	Titer	D42	D201	0.00919	0.0213	0.062052399
IgG1_S1_CoVLP with AS03	IgG1_S1	CoVLP with AS03	IgG1	S1	Titer	D42	D201	0.00029	0.001179	3.280499942
IgG1_S2_CoVLP	IgG1_S2	CoVLP	IgG1	S2	Titer	D42	D201	0.211	0.321031	0.109456135
IgG1_S2_CoVLP with AS03	IgG1_S2	CoVLP with AS03	IgG1	S2	Titer	D42	D201	0.00258	0.007031	0.450789126
IgG1_Spike_CoVLP	IgG1_Spike	CoVLP	IgG1	Spike	Titer	D42	D201	0.13	0.222345	0.241885545
IgG1_Spike_CoVLP with AS03	IgG1_Spike	CoVLP with AS03	IgG1	Spike	Titer	D42	D201	0.000504	0.001689	2.702108882

IgG2_Alpha.B1.1.7.S_CoVLP	IgG2_Alpha.B1.1.7.S	CoVLP	IgG2	Alpha.B1.1.7.S	Titer	D42	D201	0.9	0.933891	0.140236342
IgG2_Alpha.B1.1.7.S_CoVLP with AS03	IgG2_Alpha.B1.1.7.S	CoVLP with AS03	IgG2	Alpha.B1.1.7.S	Titer	D42	D201	0.323	0.463029	0.097020608
IgG2_Beta.B.1.351.S_CoVLP	IgG2_Beta.B.1.351.S	CoVLP	IgG2	Beta.B.1.351.S	Titer	D42	D201	0.0443	0.090797	0.078221318
IgG2_Beta.B.1.351.S_CoVLP with AS03	IgG2_Beta.B.1.351.S	CoVLP with AS03	IgG2	Beta.B.1.351.S	Titer	D42	D201	6.10E-05	0.000398	0.469334285
IgG2_Delta.B.1.617.2.S_CoVLP	IgG2_Delta.B.1.617.2.S	CoVLP	IgG2	Delta.B.1.617.2.S	Titer	D42	D201	0.00418	0.010471	0.105303614
IgG2_Delta.B.1.617.2.S_CoVLP with AS03	IgG2_Delta.B.1.617.2.S	CoVLP with AS03	IgG2	Delta.B.1.617.2.S	Titer	D42	D201	0.000153	0.00069	0.384188825
IgG2_Gamma.P1.S_CoVLP	IgG2_Gamma.P1.S	CoVLP	IgG2	Gamma.P1.S	Titer	D42	D201	0.0577	0.112674	0.044355212
IgG2_Gamma.P1.S_CoVLP with AS03	IgG2_Gamma.P1.S	CoVLP with AS03	IgG2	Gamma.P1.S	Titer	D42	D201	0.000153	0.00069	0.515610642
IgG2_NTD_CoVLP	IgG2_NTD	CoVLP	IgG2	NTD	Titer	D42	D201	0.117	0.207257	-0.010907142

IgG2_NTD_CoVLP with AS03	IgG2_NTD	CoVLP with AS03	IgG2	NTD	Titer	D42	D201	0.632	0.718972	0.022039736
IgG2_Omicron.B.1.1.529.S_CoVLP	IgG2_Omicron.B.1.1.529.S	CoVLP	IgG2	Omicron.B.1.1.529.S	Titer	D42	D201	0.159	0.2544	0.309856311
IgG2_Omicron.B.1.1.529.S_CoVLP with AS03	IgG2_Omicron.B.1.1.529.S	CoVLP with AS03	IgG2	Omicron.B.1.1.529.S	Titer	D42	D201	0.579	0.690346	-0.382088973
IgG2_RBD_CoVLP	IgG2_RBD	CoVLP	IgG2	RBD	Titer	D42	D201	0.274	0.409349	0.035603434
IgG2_RBD_CoVLP with AS03	IgG2_RBD	CoVLP with AS03	IgG2	RBD	Titer	D42	D201	0.000305	0.001201	0.263372729
IgG2_S1_CoVLP	IgG2_S1	CoVLP	IgG2	S1	Titer	D42	D201	0.0386	0.081125	0.070695535
IgG2_S1_CoVLP with AS03	IgG2_S1	CoVLP with AS03	IgG2	S1	Titer	D42	D201	6.10E-05	0.000398	0.771073522
IgG2_S2_CoVLP	IgG2_S2	CoVLP	IgG2	S2	Titer	D42	D201	1	1	-0.052335244
IgG2_S2_CoVLP with AS03	IgG2_S2	CoVLP with AS03	IgG2	S2	Titer	D42	D201	0.159	0.2544	0.122426971
IgG2_Spike_CoVLP	IgG2_Spike	CoVLP	IgG2	Spike	Titer	D42	D201	0.0214	0.046554	0.099373193
IgG2_Spike_CoVLP with AS03	IgG2_Spike	CoVLP with AS03	IgG2	Spike	Titer	D42	D201	0.00058	0.001893	0.295512974

IgG3_Alpha.B1.1.7.S_CoVLP	IgG3_Alpha.B1.1.7.S	CoVLP	IgG3	Alpha.B1.1.7.S	Titer	D42	D201	0.00629	0.015599	0.541564541
IgG3_Alpha.B1.1.7.S_CoVLP with AS03	IgG3_Alpha.B1.1.7.S	CoVLP with AS03	IgG3	Alpha.B1.1.7.S	Titer	D42	D201	0.000381	0.001432	3.473418085
IgG3_Beta.B.1.351.S_CoVLP	IgG3_Beta.B.1.351.S	CoVLP	IgG3	Beta.B.1.351.S	Titer	D42	D201	0.00101	0.003055	0.713851241
IgG3_Beta.B.1.351.S_CoVLP with AS03	IgG3_Beta.B.1.351.S	CoVLP with AS03	IgG3	Beta.B.1.351.S	Titer	D42	D201	0.000153	0.00069	4.363726481
IgG3_Delta.B.1.617.2.S_CoVLP	IgG3_Delta.B.1.617.2.S	CoVLP	IgG3	Delta.B.1.617.2.S	Titer	D42	D201	0.00336	0.00896	0.741382299
IgG3_Delta.B.1.617.2.S_CoVLP with AS03	IgG3_Delta.B.1.617.2.S	CoVLP with AS03	IgG3	Delta.B.1.617.2.S	Titer	D42	D201	0.000153	0.00069	4.251692931
IgG3_Gamma.P1.S_CoVLP	IgG3_Gamma.P1.S	CoVLP	IgG3	Gamma.P1.S	Titer	D42	D201	0.00418	0.010471	0.454568524
IgG3_Gamma.P1.S_CoVLP with AS03	IgG3_Gamma.P1.S	CoVLP with AS03	IgG3	Gamma.P1.S	Titer	D42	D201	0.000153	0.00069	4.499993584
IgG3_NTD_CoVLP	IgG3_NTD	CoVLP	IgG3	NTD	Titer	D42	D201	0.528	0.638751	-0.030845319

IgG3_NTD_CoVLP with AS03	IgG3_NTD	CoVLP with AS03	IgG3	NTD	Titer	D42	D201	0.00168	0.004902	1.059902275
IgG3_Omicron.B.1.1.529.S_CoVLP	IgG3_Omicron.B.1.1.529.S	CoVLP	IgG3	Omicron.B.1.1.529.S	Titer	D42	D201	0.0182	0.0403	0.49416099
IgG3_Omicron.B.1.1.529.S_CoVLP with AS03	IgG3_Omicron.B.1.1.529.S	CoVLP with AS03	IgG3	Omicron.B.1.1.529.S	Titer	D42	D201	1.53E-05	0.00019	4.420632974
IgG3_RBD_CoVLP	IgG3_RBD	CoVLP	IgG3	RBD	Titer	D42	D201	0.13	0.222345	0.139979455
IgG3_RBD_CoVLP with AS03	IgG3_RBD	CoVLP with AS03	IgG3	RBD	Titer	D42	D201	0.00209	0.005958	2.565670909
IgG3_S1_CoVLP	IgG3_S1	CoVLP	IgG3	S1	Titer	D42	D201	0.00919	0.0213	0.418051478
IgG3_S1_CoVLP with AS03	IgG3_S1	CoVLP with AS03	IgG3	S1	Titer	D42	D201	0.000107	0.000553	4.112397283
IgG3_S2_CoVLP	IgG3_S2	CoVLP	IgG3	S2	Titer	D42	D201	0.105	0.187338	0.202855445
IgG3_S2_CoVLP with AS03	IgG3_S2	CoVLP with AS03	IgG3	S2	Titer	D42	D201	0.00258	0.007031	3.349699102
IgG3_Spike_CoVLP	IgG3_Spike	CoVLP	IgG3	Spike	Titer	D42	D201	0.00919	0.0213	0.520470835
IgG3_Spike_CoVLP with AS03	IgG3_Spike	CoVLP with AS03	IgG3	Spike	Titer	D42	D201	0.000107	0.000553	3.892679429

IgG4_Alpha.B1.1.7.S_CoVLP	IgG4_Alpha.B1.1.7.S	CoVLP	IgG4	Alpha.B1.1.7.S	Titer	D42	D201	0.632	0.718972	0.003488627
IgG4_Alpha.B1.1.7.S_CoVLP with AS03	IgG4_Alpha.B1.1.7.S	CoVLP with AS03	IgG4	Alpha.B1.1.7.S	Titer	D42	D201	0.487	0.616204	0.008851363
IgG4_Beta.B.1.351.S_CoVLP	IgG4_Beta.B.1.351.S	CoVLP	IgG4	Beta.B.1.351.S	Titer	D42	D201	0.464	0.596228	0.016435774
IgG4_Beta.B.1.351.S_CoVLP with AS03	IgG4_Beta.B.1.351.S	CoVLP with AS03	IgG4	Beta.B.1.351.S	Titer	D42	D201	0.12	0.209577	0.046774981
IgG4_Delta.B.1.617.2.S_CoVLP	IgG4_Delta.B.1.617.2.S	CoVLP	IgG4	Delta.B.1.617.2.S	Titer	D42	D201	0.98	0.996066	-0.006824355
IgG4_Delta.B.1.617.2.S_CoVLP with AS03	IgG4_Delta.B.1.617.2.S	CoVLP with AS03	IgG4	Delta.B.1.617.2.S	Titer	D42	D201	0.174	0.272956	0.040153765
IgG4_Gamma.P1.S_CoVLP	IgG4_Gamma.P1.S	CoVLP	IgG4	Gamma.P1.S	Titer	D42	D201	0.669	0.740679	0.014668791
IgG4_Gamma.P1.S_CoVLP with AS03	IgG4_Gamma.P1.S	CoVLP with AS03	IgG4	Gamma.P1.S	Titer	D42	D201	0.145	0.239733	0.027025247
IgG4_NTD_CoVLP	IgG4_NTD	CoVLP	IgG4	NTD	Titer	D42	D201	0.404	0.544522	0.01486588

IgG4_NTD_CoVLP with AS03	IgG4_NTD	CoVLP with AS03	IgG4	NTD	Titer	D42	D201	0.00258	0.007031	0.031305584
IgG4_Omicron.B.1.1.529.S_CoVLP	IgG4_Omicron.B.1.1.529.S	CoVLP	IgG4	Omicron.B.1.1.529.S	Titer	D42	D201	0.175	0.272956	-0.003231908
IgG4_RBD_CoVLP	IgG4_RBD	CoVLP	IgG4	RBD	Titer	D42	D201	0.339	0.476818	0.032160054
IgG4_RBD_CoVLP with AS03	IgG4_RBD	CoVLP with AS03	IgG4	RBD	Titer	D42	D201	0.103	0.186453	0.035345223
IgG4_S1_CoVLP	IgG4_S1	CoVLP	IgG4	S1	Titer	D42	D201	0.0934	0.170318	-0.031787904
IgG4_S1_CoVLP with AS03	IgG4_S1	CoVLP with AS03	IgG4	S1	Titer	D42	D201	0.072	0.137354	0.042886342
IgG4_S2_CoVLP	IgG4_S2	CoVLP	IgG4	S2	Titer	D42	D201	0.821	0.862746	0.001018062
IgG4_S2_CoVLP with AS03	IgG4_S2	CoVLP with AS03	IgG4	S2	Titer	D42	D201	0.0277	0.059221	0.01891417
IgG4_Spike_CoVLP	IgG4_Spike	CoVLP	IgG4	Spike	Titer	D42	D201	0.348	0.476818	-0.023239269
IgG4_Spike_CoVLP with AS03	IgG4_Spike	CoVLP with AS03	IgG4	Spike	Titer	D42	D201	0.193	0.295457	0.047849468
IgM_Alpha.B1.1.7.S_CoVLP	IgM_Alpha.B1.1.7.S	CoVLP	IgM	Alpha.B1.1.7.S	Titer	D42	D201	1	1	-0.197604632
IgM_Alpha.B1.1.7.S_CoVLP with AS03	IgM_Alpha.B1.1.7.S	CoVLP with AS03	IgM	Alpha.B1.1.7.S	Titer	D42	D201	7.63E-05	0.00044	1.632424562

IgM_Beta.B.1.351.S_CoVLP	IgM_Beta.B.1.351.S	CoVLP	IgM	Beta.B.1.351.S	Titer	D42	D201	0.298	0.434729	-0.157502628
IgM_Beta.B.1.351.S_CoVLP with AS03	IgM_Beta.B.1.351.S	CoVLP with AS03	IgM	Beta.B.1.351.S	Titer	D42	D201	3.05E-05	0.000222	2.187088619
IgM_Delta.B.1.617.2.S_CoVLP	IgM_Delta.B.1.617.2.S	CoVLP	IgM	Delta.B.1.617.2.S	Titer	D42	D201	0.464	0.596228	-0.085683885
IgM_Delta.B.1.617.2.S_CoVLP with AS03	IgM_Delta.B.1.617.2.S	CoVLP with AS03	IgM	Delta.B.1.617.2.S	Titer	D42	D201	1.53E-05	0.00019	2.502590469
IgM_Gamma.P1.S_CoVLP	IgM_Gamma.P1.S	CoVLP	IgM	Gamma.P1.S	Titer	D42	D201	0.562	0.673314	-0.077511974
IgM_Gamma.P1.S_CoVLP with AS03	IgM_Gamma.P1.S	CoVLP with AS03	IgM	Gamma.P1.S	Titer	D42	D201	1.53E-05	0.00019	3.332938756
IgM_NTD_CoVLP	IgM_NTD	CoVLP	IgM	NTD	Titer	D42	D201	0.348	0.476818	-0.065996943
IgM_NTD_CoVLP with AS03	IgM_NTD	CoVLP with AS03	IgM	NTD	Titer	D42	D201	0.0202	0.044333	0.492878261
IgM_Omicron.B.1.1.529.S_CoVLP	IgM_Omicron.B.1.1.529.S	CoVLP	IgM	Omicron.B.1.1.529.S	Titer	D42	D201	0.98	0.996066	-0.127516793

IgM_Omicron.B.1.1.529.S_CoVLP with AS03	IgM_Omicron.B.1.1.529.S	CoVLP with AS03	IgM	Omicron.B.1.1.529.S	Titer	D42	D201	0.00665	0.016169	1.375198889
IgM_RBD_CoVLP	IgM_RBD	CoVLP	IgM	RBD	Titer	D42	D201	0.669	0.740679	0.088660823
IgM_RBD_CoVLP with AS03	IgM_RBD	CoVLP with AS03	IgM	RBD	Titer	D42	D201	0.000504	0.001689	1.32382591
IgM_S1_CoVLP	IgM_S1	CoVLP	IgM	S1	Titer	D42	D201	0.821	0.862746	-0.091035076
IgM_S1_CoVLP with AS03	IgM_S1	CoVLP with AS03	IgM	S1	Titer	D42	D201	1.53E-05	0.00019	3.944337923
IgM_S2_CoVLP	IgM_S2	CoVLP	IgM	S2	Titer	D42	D201	0.323	0.463029	-0.436676846
IgM_S2_CoVLP with AS03	IgM_S2	CoVLP with AS03	IgM	S2	Titer	D42	D201	0.000153	0.00069	1.365735071
IgM_Spike_CoVLP	IgM_Spike	CoVLP	IgM	Spike	Titer	D42	D201	0.706	0.764398	0.12386703
IgM_Spike_CoVLP with AS03	IgM_Spike	CoVLP with AS03	IgM	Spike	Titer	D42	D201	1.53E-05	0.00019	3.857300969
MIP1b+_Beta.B.1.351.S_CoVLP	MIP1b+_Beta.B.1.351.S	CoVLP	MIP1b+	Beta.B.1.351.S	Function	D42	D201	0.632	0.718972	0.032146645
MIP1b+_Beta.B.1.351.S_CoVLP with AS03	MIP1b+_Beta.B.1.351.S	CoVLP with AS03	MIP1b+	Beta.B.1.351.S	Function	D42	D201	0.00665	0.016169	0.654983505

MIP1b+_Gamma.P1.S_CoVLP	MIP1b+_Gamma.P1.S	CoVLP	MIP1b+	Gamma.P1.S	Function	D42	D201	0.528	0.638751	-0.427698488
MIP1b+_Gamma.P1.S_CoVLP with AS03	MIP1b+_Gamma.P1.S	CoVLP with AS03	MIP1b+	Gamma.P1.S	Function	D42	D201	0.284	0.421749	0.205810856
MIP1b+_Spike_CoVLP	MIP1b+_Spike	CoVLP	MIP1b+	Spike	Function	D42	D201	0.0507	0.1014	0.413710097
MIP1b+_Spike_CoVLP with AS03	MIP1b+_Spike	CoVLP with AS03	MIP1b+	Spike	Function	D42	D201	1.53E-05	0.00019	0.606711171

Gating strategy (A) Luminex assay, **(B)** ADCP, **(C)** ADNP, **(D)** ADCD, and **(E)** ADNK

A)

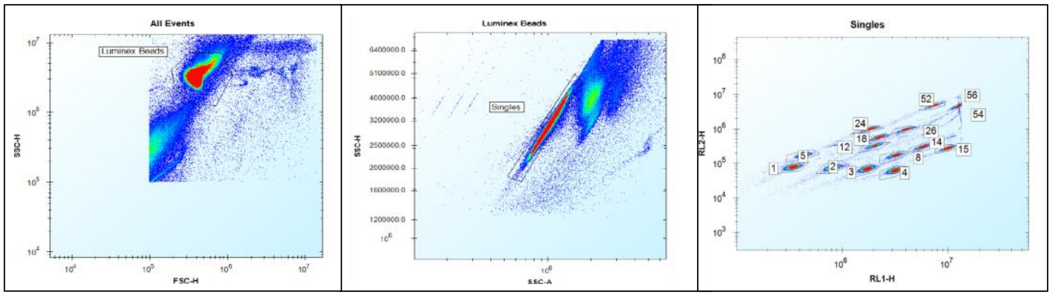

B)

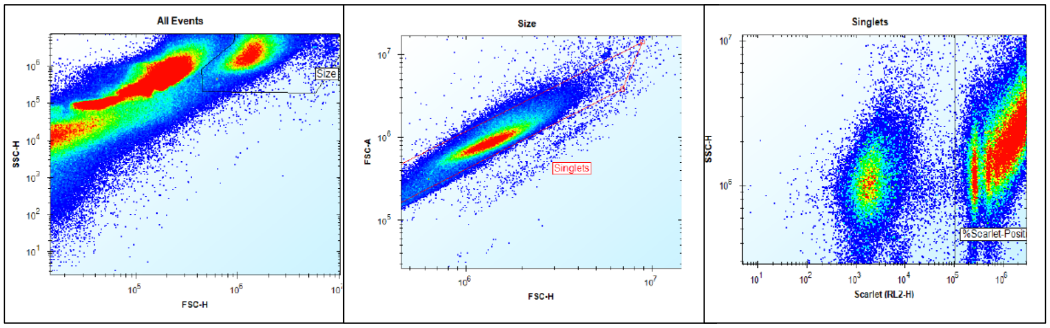

C)

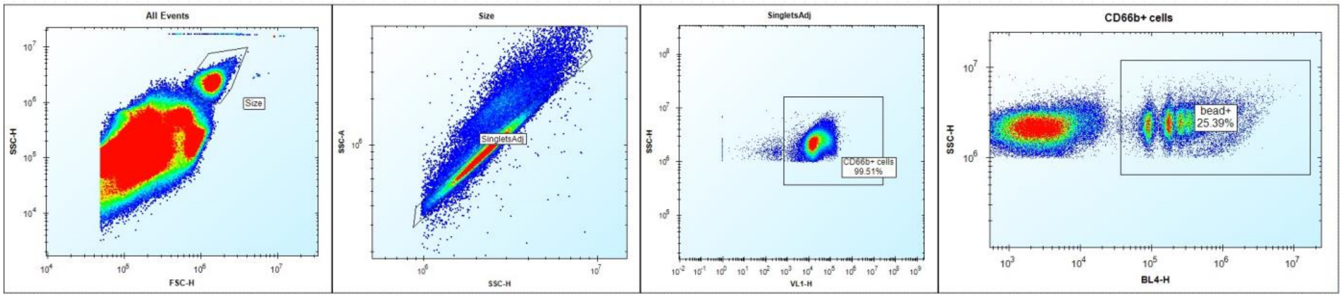

D)

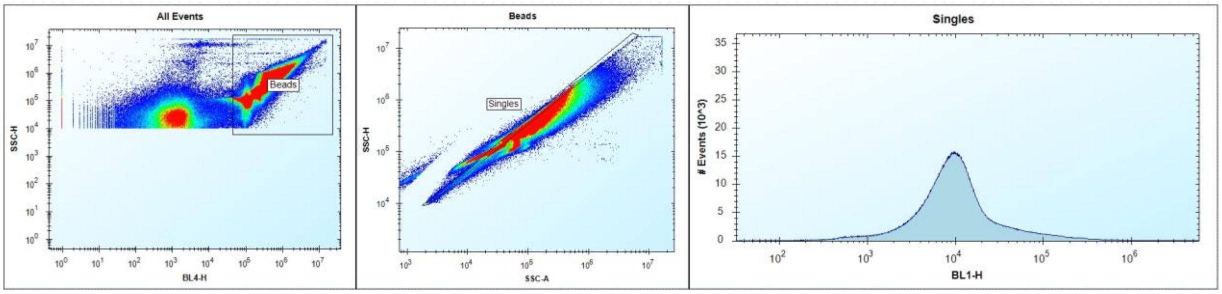

E)

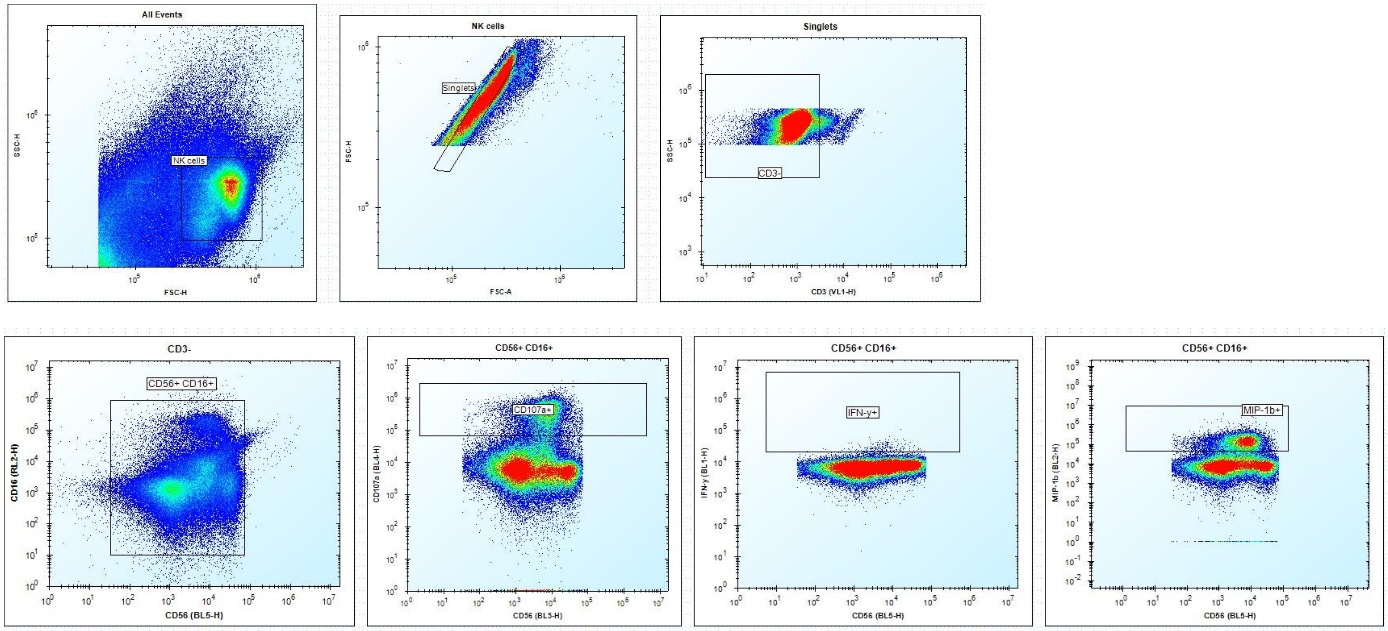

Clinical Protocol

A Randomized, Partially-Blinded, Dose-Ranging Phase 1 Study to Assess the Safety, Tolerability, and Immunogenicity of a Recombinant Coronavirus-Like Particle COVID-19 Vaccine in Adults 18-55 Years of Age

CP-PRO-CoVLP-019; Phase 1

Coronavirus-Like Particle COVID-19 Vaccine

Name of Sponsor: Medicago R&D Inc.
1020 route de l'Église, bureau 600Québec (Qc), Canada G1V 3V9

Status: Final version 3.0

Date: 21-Sep-2020

Confidential Information

Information in this protocol is confidential and should not be disclosed, other than to those directly involved in the execution or the ethical review of the study, without written authorization from Medicago R&D Inc. (herein after known as "Medicago"), and its affiliates. This study will be conducted in accordance with applicable Good Clinical Practice (GCP) guidelines, the United States Code of Federal Regulations (CFR), and International Conference on Harmonization (ICH) guidelines.

INVESTIGATOR AGREEMENT

I have read the version 3.0 (dated 21-Sep-2020) Protocol No. CP-PRO-CoVLP-019 titled, "A Randomized, Partially-Blinded, Dose-Ranging Phase 1 Study to Assess the Safety, Tolerability, and Immunogenicity of a Recombinant Coronavirus-Like Particle COVID-19 Vaccine in Adults 18-55 Years of Age".

I have fully discussed the objectives of this study and the contents of this protocol with the Sponsor's representative.

I understand that the information in this protocol is confidential and should not be disclosed, other than to those directly involved in the execution or the ethical review of the study, without prior written authorization from Medicago. It is, however, permissible to discuss information contained in this protocol with a subject in order to obtain consent once Institutional Review Board (IRB) approval is obtained.

I agree to conduct this study according to this protocol and to comply with its requirements, subject to ethical and safety considerations and guidelines, and to conduct the study in accordance with International Conference on Harmonisation (ICH) guidelines, Good Clinical Practice (GCP), Investigational New Drug (IND) regulations, and other applicable regulatory requirements.

I understand that the Sponsor may decide to suspend or prematurely terminate this study at any time for whatever reason and that such a decision will be communicated to me in writing. Conversely, should I decide to withdraw from execution of the study, I will communicate myintention immediately in writing to the Sponsor.

Principal Investigator:

Name (typed or printed): ______________________________

Institution and Address: ______________________________

____

____

Telephone Number: ______________________________

______________________________ ______________________________

Signature Date (YYYY-MM-DD)

Note: If the address or the telephone number of the Investigator changes during the course ofthe study, written notification will be provided by the Investigator to the Sponsor, and a protocol amendment will not be required.

TABLE OF CONTENTS

SIGNATURES ... 2
INVESTIGATOR AGREEMENT ... 4
TABLE OF CONTENTS ... 5
LIST OF TABLES ... 10
SYNOPSIS ... 11
ABBREVIATIONS ... 24
INTRODUCTION ... 26
Background ... 26
Background of the Investigational Product ... 27
Background of the Adjuvant AS03 ... 28
Background of the Adjuvant CpG 1018 ... 29
Pre-clinical Studies ... 30
Clinical Studies ... 30
Overall Rationale for the Study ... 30
OBJECTIVES ... 30
Primary Objectives ... 30
Secondary Objectives ... 31
Exploratory Objectives ... 31
STUDY ADMINISTRATIVE STRUCTURE ... 32
STUDY DESIGN AND RATIONALE ... 32
Overview of Study Design ... 32
Rationale of Study Design ... 33
Randomization ... 33
Blinding 34
Dose Selection and Dosage Regimen ... 34
Route of Administration ... 34
Study Duration ... 34
STUDY POPULATION ... 34
Inclusion Criteria ... 34
Exclusion Criteria ... 36
Prior and Concomitant Therapy ... 38

Prohibited Therapy 38
TREATMENT ALLOCATION AND BLINDING 39
Randomization 39
Blinding 39
DOSAGE AND ADMINISTRATION 41
SPECIMENS AND CLINICAL SUPPLIES 41
Management of Samples 41
Collection of Samples 42
Clinical Supplies 42
TREATMENT COMPLIANCE 42
STUDY EVALUATIONS 43
Study Procedures 43
Overview 43
General COVID-19 Precautions at Clinical Sites 43
Screening (Visits 1) 44
Dose 1 Vaccination (Day 0) (Visit 2) 45
Prior to Vaccination 46
Vaccination 46
Thirty Minutes Post-vaccination 47
Day 1 and Day 8 (Telephone Contact) 49
Day 3 Follow-up (Visit 3) 50
Day 21 Dose 2 Vaccination (Visit 4) 50
Pre-vaccination 51
Dose 2 Vaccination 51
Post-Dose 2 Vaccination 52
Day 22 and Day 29 (Telephone Contact) 52
Day 24 (Visit 5) 52
Day 42 (Visit 6) 52
Monthly Calls Thereafter (Telephone Contact) 53
10.1.12 Day 201 (Visit 7) 54
Final Visit – Day 386 (Visit 8) 54
Unscheduled Clinical Site Visit 55

Safety 55
Evaluations 55
Solicited Local and Systemic Adverse Events 56
Adverse Events and SARS-CoV-2 Positive Reports 57
Clinical Laboratory Tests 58
Vital Signs 59
Physical Examinations 59
Pregnancy 59
Safety Endpoints 60
Primary Endpoints 60
Secondary Endpoints 60
Exploratory Endpoints 60
Immunogenicity 61
Immunogenicity Evaluations 61
Immunogenicity Endpoints 61
Primary Endpoints 61
Secondary Endpoints 61
Exploratory Endpoints 62
SUBJECT COMPLETION/WITHDRAWAL 62
Temporary Contraindications 62
Screening Failures 62
Contraindications for Subsequent Vaccination 62
Removal of Subjects from Treatment or Assessment 63
Follow-up of Discontinuations 64
Lost to Follow-up Procedures 64
Interruption of the Study 64
STATISTICAL METHODS 65
Analysis Populations 65
Safety Analysis Set 65
Intention-To-Treat Set 65
Per Protocol Set 65
Sample Size Determination 66

Day 21 Data Transfer, Day 42 Data Analysis, and Interim Analysis 66
Safety Analyses 66
Analysis of Primary Endpoints 67
Analysis of Secondary Endpoints 67
Analysis of Exploratory Endpoints 67
Immunogenicity Analyses 68
Analysis of Primary Endpoints 68
Analysis of Secondary Endpoints 68
Analysis of Exploratory Endpoints 69
Baseline and Subject Disposition 69
ADVERSE EVENT REPORTING 70
Definitions 70
Serious Adverse Events 70
Adverse Events 70
Adverse Events of Special Interest 71
Adverse Events of Special Interest for the Coronavirus-Like Particle COVID-19 Vaccine 71
Vaccine Enhanced Disease 71
Hypersensitivity Reactions 72
Adverse Events of Special Interest for the Adjuvants 72
Potential Immune-Mediated Diseases and Other AESI as Listed in Section 19.5 72
Expectedness of an Adverse Drug Reaction 73
Initial SAE, AESI, and Pregnancy Reports Reporting by the Investigator 73
Follow-up Reporting by the Investigator 74
Reporting of SAEs Occurring after Study Termination 74
Causal Relationship 74
Reporting of SAEs to Health Authorities and IRB 75
Independent Data Monitoring Committee 75
Safety Review and Stopping Rules 76
INVESTIGATIONAL PRODUCT INFORMATION 77
Identity of Investigational Product 77
Study Vaccine Composition 78

Preparation and Administration of Study Vaccine 78
Preparation, Handling, Storage, and Precautions for Use.. 79
Packaging79
Labeling 79
Drug Accountability... 79
STUDY-SPECIFIC MATERIALS 80
ETHICAL ASPECTS 80
Study-Specific Design Considerations 80
Regulatory Ethics Compliance 80
Investigator Responsibilities 80
Independent Ethics Committee or Institutional Review Board 81
Informed Consent.. 82
Privacy of Personal Data.. 82
ADMINISTRATIVE REQUIREMENTS 82
Protocol Amendments.. 82
Regulatory Documentation 83
Regulatory Approval/Notification 83
Source Documentation... 83
Diary and Memory Aid.. 84
Case Report Form Completion 84
Quality Control (Study Monitoring) 85
Quality Assurance 85
Record Retention 85
Study Completion/Termination 86
Study Completion 86
Study Termination 86
Registration of Clinical Studies and Disclosure of Results 86
Publication Policy 87
REFERENCES 87
APPENDICES 92
Appendix 1 – Sample Ruler to Measure Local Adverse Events.. 92
Appendix 2 – Subject Diary Sample Pages 93

Appendix 3 – Subject Memory Aid Sample Pages 99
Appendix 4 – Three-Day Safety Summary (Sample) 106
Appendix 5 – List of Potential Immune-Mediated Diseases 107

LIST OF TABLES

Table 1 Time and Events Schedule: General Information 21
Table 2 Study Administrative Structure 32
Table 3 Study Treatment Groups 33
Table 4 Estimated Blood Volume Drawn 43
Table 5 Severity Grades for Solicited Local and Systemic Adverse Events 56
Table 6 Clinical Laboratory Tests 58

SYNOPSIS

**Sponsor:**	Medicago R&D Inc.
**Investigational Product:**	Coronavirus-Like Particle (CoVLP) COVID-19 Vaccine
**Active Substance(s):**	The Coronavirus-Like Particle COVID-19 Vaccine is composed of recombinant spike (S) glycoprotein expressed as virus-like particles (VLPs) and will be administered unadjuvanted and adjuvanted with CpG 1018 (manufactured by Dynavax) or AS03 (manufactured by GlaxoSmithKline).
**Protocol Title:**	A Randomized, Partially Blinded, Dose-Ranging Phase 1 Study to Assess the Safety, Tolerability, and Immunogenicity of a Recombinant Coronavirus-Like Particle COVID-19 Vaccine in Adults 18-55 Years of Age
**Protocol Number:**	CP-PRO-CoVLP-019
**Development Phase:**	Phase 1
**Study Center(s):**	The study will be conducted at multiple sites in Canada.
**Study Rationale:**	The Phase 1 study is intended to assess the safety and immunogenicity profile of a plant-derived Coronavirus-Like Particle COVID-19 Vaccine in healthy adults and unblinded results generated from this study will inform design decisions (i.e. most safe and effective dose level[s] and regimen[s], more effective adjuvant) of a subsequent Phase 2 study.<br><br>The study will be a randomized, partially-blinded, prime-boost, staggered dose-escalation Phase 1 study intended to assess the safety, tolerability, and immunogenicity of the Coronavirus-Like Particle COVID-19 Vaccine at three dose levels (3.75 µg, 7.5 µg, and 15 µg VLP) unadjuvanted or adjuvanted with either CpG 1018 or AS03 in healthy adults 18 to 55 years of age, who have been tested for the absence of SARS-CoV-2 antibodies. At each dose level, the vaccine will initially be administered to a small number of subjects. Vaccination of the remaining subjects at the same dose level and the next higher vaccine dose level will be administered with approval of the Independent Data Monitoring Committee (IDMC). The same process will be followed for the second vaccine administration. All subjects will receive two doses of the same treatment group (dose level and adjuvant) of the vaccine 21 days apart and data collected will be

	used to determine if one or two doses is/are required of the Coronavirus-Like Particle COVID-19 Vaccine.<br><br>All subjects will be followed for a period of 12 months after the second administration of the vaccine for safety and immunogenicity testing at the end of the follow-up period.
**Planned Study Period:**	The total planned study period is approximately 14 months, from screening procedures up to the end of the study.
**Study Objectives**<br><br>**Primary Objectives:**	The primary objectives of this study are:<br><br>To assess the safety and tolerability of the Coronavirus-Like Particle COVID-19 Vaccine, as determined by the occurrence(s) of:<br>Immediate adverse events (AEs) within 30 minutes aftereach vaccination;<br>Solicited local and systemic AEs up to 7 days after each vaccination;<br>Unsolicited AEs, serious adverse events (SAEs), AEs leading to withdrawal, AEs of special interest (AESIs), anddeaths up to 21 days after each vaccination;<br>Subjects with normal and abnormal urine, haematological,and biochemical values.<br>To assess the immunogenicity of the Coronavirus-Like Particle COVID-19 Vaccine up to 21 days after each vaccination, as determined by:<br>Neutralizing antibody (Nab) titers induced by the vaccine against the SARS-CoV-2 virus;<br>Interferon (IFN)-γ enzyme-linked immuno spot assay (ELISpot) response induced by the vaccine against the SARS-CoV-2 virus to assess T helper 1 (Th1) type response;<br>Interleukin (IL)-4 ELISpot response induced by the vaccine against the SARS-CoV-2 virus to assess T helper 2 (Th2) type response.
**Secondary Objectives:**	The secondary objectives of this study are:<br><br>To assess the immunogenicity of the Coronavirus-Like Particle COVID-19 Vaccine up to 21 days after each vaccination, as determined by the:<br>IgG and/or IgM (enzyme-linked immunosorbent assay [ELISA]) titers induced by the vaccine against the SARS-CoV-2 virus.

	To assess the immunogenicity of the Coronavirus-Like Particle COVID-19 Vaccine up to 201 and 386 days after thefirst vaccination, as determined by the:<br>Neutralizing antibody titers induced by the vaccine againstthe SARS-CoV-2 virus;<br>IFN-γ ELISpot response induced by the vaccine against the SARS-CoV-2 virus;<br>IL-4 ELISpot response induced by the vaccine against the SARS-CoV-2 virus;<br>IgG and/or IgM (ELISA) titers induced by the vaccine against the SARS-CoV-2 virus.<br>To assess the safety and tolerability of the Coronavirus-Like Particle COVID-19 Vaccine, as determined by the occurrence(s) of:<br>SAEs, AEs leading to withdrawal, AESIs, and deaths from 22 days after the last vaccination up to the end of the study.
**Exploratory Objectives:**	The exploratory objectives of this study are:<br>To assess the specific cell-mediated immune (CMI) response induced by the Coronavirus-Like Particle COVID-19 Vaccine against the SARS-CoV-2 virus up to 21 days after each vaccination and up to 201 and 386 days after the first vaccination, as measured by the percentage of CD4+ T cells expressing functional markers;<br>To measure the levels of serum IgE antibodies directed to plant-specific glycans prior to vaccination (Day 0), up to 21 days after the second vaccination (Day 42) and up to 201 and 386 days after the first vaccination (Day 201 andDay 386);<br>To measure the occurrence(s) of laboratory-confirmed (virologic or serologic methods) SARS-CoV-2 infection (withor without symptoms) up to the end of the study;<br>If deemed necessary, to further characterize the immune response and the safety profile of the Coronavirus-Like Particle COVID-19 Vaccine.
**Number of Planned Subjects:**	Approximately 180 subjects are planned for enrollment into nine treatment groups (Coronavirus-Like Particle COVID-19 Vaccine [3.75 µg, 7.5 µg, and 15 µg VLP] unadjuvanted or adjuvanted with CpG 1018 or AS03) in a 1:1:1:1:1:1:1:1:1 ratio.
**Sample Size Determination:**	The sample size of approximately 180 subjects with 20 subjects in each treatment group will make it possible to perform the initial evaluation of the vaccine immunogenicity and detect gross differences in rates of adverse events. The sample size is

	not large enough to detect all types, including less frequent or rare, adverse reactions. The objective of this study is to quantify the type, percentage, intensity, duration, and relationship of short-term post-vaccination events to determine if they differ clinically among the treatment groups.
**Study Population:**	Healthy male and female subjects, with no known comorbidities, aged 18 to 55 years will be included in the study.
**Dosage and Administration:**	Subjects will receive two intramuscular (IM) injections 21 days apart (Day 0 and Day 21), into the deltoid region of the alternating arm (each arm will be injected once), with their assigned vaccine:<br><br>Group 1: 3.75 µg of the Coronavirus-Like Particle COVID-19 Vaccine unadjuvanted, or<br>Group 2: 3.75 µg of the Coronavirus-Like Particle COVID-19 Vaccine adjuvanted with CpG 1018, or<br>Group 3: 3.75 µg of the Coronavirus-Like Particle COVID-19 Vaccine adjuvanted with AS03, or<br>Group 4: 7.5 µg of the Coronavirus-Like Particle COVID-19 Vaccine unadjuvanted, or<br>Group 5: 7.5 µg of the Coronavirus-Like Particle COVID-19 Vaccine adjuvanted with CpG 1018, or<br>Group 6: 7.5 µg of the Coronavirus-Like Particle COVID-19 Vaccine adjuvanted with AS03, or<br>Group 7: 15 µg of the Coronavirus-Like Particle COVID-19 Vaccine unadjuvanted, or<br>Group 8: 15 µg of the Coronavirus-Like Particle COVID-19 Vaccine adjuvanted with CpG 1018, or<br>Group 9: 15 µg of the Coronavirus-Like Particle COVID-19 Vaccine adjuvanted with AS03.<br><br>The volume of each injection will be 0.25 to 0.75 mL, depending on the presence or absence of adjuvant.
**Study Design:**	This is a randomized, partially-blinded, prime-boost, staggered dose-escalating study with approximately 180 healthy male and female subjects 18 to 55 years of age, who have been tested for the absence of SARS-CoV-2 antibodies. The subjects will be enrolled into nine treatment groups with three dose levels (3.75 µg, 7.5 µg, and 15 µg VLP) to receive Coronavirus-Like Particle COVID-19 Vaccine unadjuvanted or adjuvanted with CpG 1018 or AS03:

	Dose Level 1: Sixty subjects will be randomized to receive two injections of dose level 1 (3.75 µg) of the Coronavirus-Like Particle COVID-19 Vaccine unadjuvanted or adjuvanted with CpG 1018 or AS03 on Days 0 and 21 (one injection on each of these days). The three-day safety data after vaccination of the first 10 % of subjects enrolled (6 subjects) will be collected and reviewed by the IDMC, prior to permitting vaccination of the next 30 % of subjects enrolled (18 subjects) at the same dose level 1 and escalating to vaccination of subjects with dose level 2. The three-day safety data after vaccination of these 18 subjects at same dose level 1 will be collected and reviewed by the IDMC, prior to permitting vaccination of the remaining subjects at the same dose level 1. The same process will be followed for the second vaccine administration. In addition, the vaccinations of the first 6 subjects at the lowest dose level will be staggered so that each vaccination must be performed at least30 minutes apart.<br><br>Dose Level 2: Sixty subjects will be randomized to receive two injections of dose level 2 (7.5 µg) of the Coronavirus-Like Particle COVID-19 Vaccine unadjuvanted or adjuvanted with CpG 1018 or AS03 on Days 0 and 21 (one injection on each of these days). The three-day safety data after vaccination of the first 10 % of subjects enrolled (6 subjects) will be collected and reviewed by the IDMC, prior to permitting vaccination of the next 30 % of subjects enrolled (18 subjects) at the same dose level 2 and escalating to vaccination of subjects with dose level 3. The three-day safety data after vaccination of these 18 subjects at same dose level 2 will be collected and reviewed by the IDMC, prior to permitting vaccination of the remaining subjects at the same dose level 2. The same process will be followed for the second vaccine administration.<br><br>Dose Level 3: Sixty subjects will be randomized to receive two injections of dose level 3 (15 µg) of the Coronavirus-Like Particle COVID-19 Vaccine unadjuvanted or adjuvanted with CpG 1018 or AS03 on Days 0 and 21 (one injection on each of these days). The three-day safety data after vaccination of the first 10 % of subjects enrolled (6 subjects) will be collected and reviewed by the IDMC, prior to permitting vaccination of the next 30 % of subjects enrolled (18 subjects) at the same dose level 3. The three-day safety data after vaccination of these 18 subjects at same dose level 3 will be collected and reviewed by the IDMC, prior to permitting vaccination of the remaining subjects at the same dose level 3. The same process will be followed for the second vaccine administration.

	Subjects will be screened up to seven days in advance of the first vaccine administration and will demonstrate a satisfactory baseline medical assessment by history, general physical examination, haematological and biochemical, and serological analysis. In addition, a test for the absence of SARS-CoV-2 antibodies will be performed at screening and prior to vaccination on Day 0, as well as on Day 42, Day 201, and Day 386. On Days 0 and 21, vaccine administration will occur. Telephone contacts will be made one day, and eight days after each vaccine administration as well as monthly after Day 42 until the final study visit (Day 386), specifically for review of the subject's safety and concomitant medication data. Visits to the Investigator site will occur three days after each vaccine administration (Day 3 and Day 24) for key safety assessments and 21 days after each vaccine administration (Day 21 and Day 42) for safety and immunogenicity assessments. Subjects will have monthly telephone calls after Day 42 and will return to the Investigator site on Day 201 and Day 386 for safety follow-ups and immunogenicity assessments (six-month and 12-month follow-ups).
**Blinding:**	In this study, a partially-blind design is applied whereby the following individuals will not have access to treatment allocation (i.e. remain "blind") throughout the entire study duration: the subjects, the Investigators and all personnel involved in the clinical conduct of the study (except the staff involved in the preparation and administration of the study vaccine, the quality assurance auditor, and quality control reviewers), Medicago clinical staff and medical staff involved insafety evaluations (e.g. causality assessments), and all personnelinvolved in sample analysis at the central and testing (Nab, ELISA, ELISpot, and flow cytometry) laboratories. The partially-blind trial design will also allow all study staff to remain unblinded to dose level only in order to manage the staggered ascending dose escalation and IDMC coordination.<br><br>The IDMC and the independent statistician involved in the preparation of the safety data summary for the IDMC reviews will have access to or be aware of treatment allocation (i.e. be "unblinded"). The IDMC will be reviewing safety data during the study and will be making recommendations throughout the study.<br><br>In addition, select personnel of the third-party statistical team will have access to treatment allocation (i.e. be "unblinded") for the entire duration of the study since these personnel will be

	involved in the Day 21 data transfer and/or the Day 42 data analysis (see Statistical Methods section below).<br><br>A small number of Medicago personnel will have access to the Day 21 data and the Day 42 data analysis (i.e. be “unblinded” to results and/or treatment allocation) and will be able to look at data throughout the study. The Medicago personnel will include senior personnel in Scientific and Medical Affairs, Biostatistics, Safety, Product Development, and Regulatory Affairs.
**Safety Evaluations:**	Clinical safety methods will include repeated urine, blood chemistry and haematology testing. Safety and tolerability endpoints will include immediate adverse events (30 minutes after each vaccination), solicited local and systemic adverse events (up to seven days after each vaccination), unsolicited AEs up to Day 21 following each vaccine administration, SAEs,AEs leading to withdrawal, AESIs, and deaths up to the end of the study.
**Immunogenicity Evaluations:**	Immunogenicity will be evaluated by the humoral immune response (Nab assay and IgG and/or IgM ELISA) and the CMI response induced by the Coronavirus-Like Particle COVID-19 Vaccine in subjects on Day 0, Day 21, Day 42, Day 201, and Day 386 as well as the antibody response directed against plant glycans on Days 0, 42, 201 and 386.
**Primary Endpoint:**	**Safety:**<br><br>The primary safety endpoints in this study are:<br>Percentage, intensity, and relationship to vaccination of immediate AEs (30 minutes after each vaccination);<br>Percentage, intensity, and relationship to vaccination of solicited local and systemic AEs (for seven days following each vaccine administration);<br>Percentage, intensity, and relationship of unsolicited AEs for21 days following each vaccine administration;<br>Occurrences of SAEs, AEs leading to withdrawal, AESIs (including vaccine-enhanced disease [VED]), and deaths for21 days following each vaccine administration;<br>Number and percentage of subjects with normal and abnormal clinically significant urine, haematological and biochemical values prior to and three days following each vaccination.<br><br>**Immunogenicity:**<br><br>The primary immunogenicity endpoints in this study are:

	Neutralizing antibody (NAb assay) response induced by the vaccine against the SARS-CoV-2 virus 21 days after each vaccination which will be analyzed using the following parameter: geometric mean titers (GMT), seroconversion (SC) rate (4-fold rise in Nab titer), and geometric mean fold rise (GMFR);<br>Specific Th1 CMI response induced by the vaccine against the SARS-CoV-2 virus 21 days after each vaccination, as measured by IFN-γ ELISpot;<br>Specific Th2 CMI response induced by the vaccine against the SARS-CoV-2 virus 21 days after each vaccination, as measured by IL-4 ELISpot.
**Secondary Endpoints:**	**Safety:**<br>The secondary safety endpoints in this study are:<br>Occurrences of SAEs, AEs leading to withdrawal, AESIs (including VED), and deaths from 22 days after the last vaccination up to Day 201;<br>Occurrences of SAEs, AEs leading to withdrawal, AESIs (including VED), and deaths from Day 202 up to the end ofthe study (Day 386).<br>**Immunogenicity:**<br>The secondary immunogenicity endpoints in this study are:<br>Specific antibody response induced by the vaccine against the SARS-CoV-2 virus 21 days after each vaccination and on Day 201 and Day 386, as measured by total IgG and/or IgM levels and will be analyzed using the following parameters: GMT and GMFR;<br>Neutralizing antibody response induced by the treatment groups against the SARS-CoV-2 virus on Day 201 and Day 386 will be analyzed using the following parameters:GMT, SC rate (4-fold rise in Nab titer), and GMFR;<br>Specific Th1 CMI response induced by the Vaccine against the SARS-CoV-2 virus on Day 201 and Day 386, as measuredby IFN-γ ELISpot;<br>Specific Th2 CMI response induced by the Vaccine against the SARS-CoV-2 virus on Day 201 and Day 386, as measuredby IL-4 ELISpot.
**Exploratory Endpoints:**	**Immunogenicity:**<br>The exploratory immunogenicity endpoints in this study are:<br>Specific CMI response induced by the vaccine against the SARS-CoV-2 virus 21 days after each vaccination and on

	Day 201 and Day 386, as measured by the percentage of CD4+ T cells expressing functional markers;<br>Specific antibody response induced by the vaccine against plant glycans 21 days after the second vaccination and on Day 201 and Day 386, as measured by serum IgE levels directed against cross-reactive carbohydrate determinate (CCD) MUXF3 using bromelain glycoprotein;<br>If deemed necessary, further characterization of the immune response of the Coronavirus-Like Particle COVID-19 Vaccine.<br>**Safety:**<br>The exploratory safety endpoints in this study are:<br>If deemed necessary, further characterization of the safety profile of the Coronavirus-Like Particle COVID-19 Vaccine;<br>Occurrence(s) of laboratory-confirmed (virologic or serologic methods) SARS-CoV-2 infection (with or without symptoms)up to the end of the study.
**Statistical Methods:**	**Populations:**<br>Descriptive statistical analyses will be performed on pre-defined population sets (the safety analysis set [SAS], the Intention-to-Treat [ITT] set, and the per protocol [PP] set) according to the Statistical Analysis Plan (SAP).<br>All safety analyses will be performed using the SAS. The analyses of all immunogenicity endpoints will be performed using the PP set and the ITT set. Analyses using the PP set will be considered the primary analyses for these objectives.
	**Day 21 Data Transfer, Day 42 Data Analysis, and Interim Analysis:**<br>The third-party statistical team will receive safety and immunogenicity data after Day 21 (last subject) and will perform data analysis after Day 42 (last subject). The data transfer and data analysis will involve select individuals from the third-party statistical team and Medicago who will be unblinded (as described in Blinding section above).<br>The Day 21 and Day 42 data analysis will allow discussions of the clinical data to inform and finalize design decisions (i.e. most effective dose level, more effective adjuvant) of the subsequent Phase 2 study, without having to wait until after the end of the follow-up period for study completion.

	**Interim Analysis:**<br>An interim analysis will be performed after the last subject has completed Day 201 assessments and selected tables, listings, and figures (as applicable) will be generated according to the SAP, by the third-party statistical team. The results of the interim analysis will be used to prepare the clinical study report(up to Day 201) for the purpose of reporting to regulatory agencies the safety and immune response profile of the Coronavirus-Like Particle COVID-19 Vaccine up to six monthsafter the second vaccination. The results from the analysis of data up to Day 386 will be presented in an addendum to the clinical study report.<br>**Statistical Analyses:**<br>All descriptive and inferential statistical analyses will be performed using Statistical Analysis System® (SAS®) software (version 9.4 or higher).<br>In general, categorical data will be summarized using the number and percent of subjects in each category and continuousdata will be summarized using descriptive statistics (mean or geometric mean, median, standard deviation, minimum, and maximum).<br>**Safety Analyses:**<br>Safety and tolerability endpoints will be summarized by treatment using descriptive statistics.<br>**Immunogenicity Analyses:**<br>For the immunogenicity analyses, point estimates and two-sided 95 % confidence interval (CI) for primary and secondary immunogenicity endpoints and responses for the treatment groups will be calculated. GMT will be compared among treatment groups by using Analysis of Variance (ANOVA) on log-transformed data. GMFR will be derived by using Analysis of Covariance (ANCOVA) to model the difference in the log of the titer values between Day 21/42/201/386 and Day 0, with treatment group as main effect and baseline titer as covariate. For GMT and GMFR comparisons, Tukey's test will be performed to show p-values for the pairwise comparisons between treatment groups. Fisher's exact tests or chi square tests will be used to compare SC among treatment groups. The CMI response will be compared among treatment groups using appropriate non-parametric (Wilcoxon) models.

Table 1 Time and Events Schedule: General Information

**Visit Type**	**Screening**	**Vaccination**	**Post-vaccination Visit/Contacts**			**Vaccination**	**Post-vaccination Visits/Contacts**						
**Study Day**	**Day -7 to 0**	**Day 0**	**Day 1 (+ 1)**	**Day 3 (-1)**	**Day 8 (± 1)**	**Day 21 (± 2)[7]**	**Day 22 (+ 1)**	**Day 24 (- 1)**	**Day 29 (± 1)**	**Day 42 (± 2)**	**Monthly Calls (± 14)[8]**	**Day 201 (± 14)**	**Day 386 (± 14)**
**Visit Number**	**1**	**2**	**Phone**	**3**	**Phone**	**4**	**Phone**	**5**	**Phone**	**6**	**Phone**	**7**	**8**
Informed consent	X												
Test for SARS-CoV-2	X	X[10,11]								X		X	X
Demographics	X												
Medical history/prior medication	X	X[1]											
Inclusion/exclusion criteria	X	X[1]											
History/symptom-directed physical examination	X[2]												
Vital Signs	X	X[3]		X		X[3]		X		X		X	X
Height, weight, and BMI	X	X											
Urinalysis	X			X		X[10]		X					
Pregnancy test (Urine)		X[10]				X[10]				X			
Pregnancy test[4] (Serum)	X												
Serology tests for HIV, Hepatitis B, and Hepatitis C	X												
Blood chemistry And Haematology	X			X		X[10]		X					
Immunogenicity -Serology (Nab assay, ELISA, and anti-plant glycans IgE antibodies)[9]		X[10]				X[9, 10]				X		X	X
Immunogenicity – CMI Response (PBMC)		X[10]				X[10]				X		X	X
Randomization		X											

Visit Type	Screening	Vaccination	Post-vaccination Visit/Contacts			Vaccination	Post-vaccination Visits/Contacts						
**Study Day**	**Day -7 to 0**	**Day 0**	**Day 1 (+ 1)**	**Day 3 (-1)**	**Day 8 (± 1)**	**Day 21 (± 2)** [7]	**Day 22 (+ 1)**	**Day 24 (- 1)**	**Day 29 (± 1)**	**Day 42 (± 2)**	**Monthly Calls (± 14)**[8]	**Day 201 (± 14)**	**Day 386 (± 14)**
**Visit Number**	**1**	**2**	**Phone**	**3**	**Phone**	**4**	**Phone**	**5**	**Phone**	**6**	**Phone**	**7**	**8**
Vaccine admin		X				X							
Immediate surveillance (30 minutes)		X				X							
Provide and collect diary and/or memory aid instructions		X				X				X		X	X
Oral digital thermometer and instructions on AEs[5]		X				X							
Collection of solicited local/ systemic AEs		X	X	X	X	X	X	X	X				
Concomitant medications		At any time during the study period											
AEs, SAEs, AESIs, pregnancy reports and laboratory-confirmed reports of SARS-CoV-2 infection (with or without symptoms)[6]		At any time during the study period											
Termination record													X

[1] Record any changes in medical history and medications and confirmation that the subject continues to meet all inclusion and no exclusion criteria since screening.

[2] History/symptom-directed physical examinations will not be routinely performed at any other visits, unless new complaints or concerns are raised by either the study subject or study staff, and if deemed to be necessary by the Investigator.

[3] Record prior to study vaccine, after the observation period and as deemed necessary.

[4] In all females of childbearing potential; at screening it will be tested in serum and at Days 0, 21, and 42 it will be tested in urine.

[5] After vaccination, subjects will be instructed on the diary and memory aid provided for their use for recording AEs and concomitant medication use.

[6] AEs will be collected up to Day 42; SAEs, AEs leading to withdrawal, and AESIs will be collected through to the end of the study. Investigators should report SAEs, AESIs, and pregnancy reports to Sponsor within 24 hours of the Investigator learning of the event. Specific contacts for the collection of information regarding all of these events will occur on Day 201 and Day 386 for SAEs, AEs leading to withdrawal, and AESI. Also, all laboratory-

Visit Type	Screening	Vaccination	Post-vaccination Visit/Contacts			Vaccination	Post-vaccination Visits/Contacts						
Study Day	Day -7 to 0	Day 0	Day 1 (+ 1)	Day 3 (-1)	Day 8 (± 1)	Day 21 (± 2) [7]	Day 22 (+ 1)	Day 24 (- 1)	Day 29 (± 1)	Day 42 (± 2)	Monthly Calls (± 14)[8]	Day 201 (± 14)	Day 386 (± 14)
Visit Number	1	2	Phone	3	Phone	4	Phone	5	Phone	6	Phone	7	8

confirmed reports of SARS-CoV-2 infection (with or without symptoms) will be collected through to the end of the study.

[7] All efforts will be done to have subjects returning on planned date for Day 21 activities. If for any reason the visit is done before or after this planned date, subsequent visits/procedures (Days 22, 24, 29, 42, 201, and 386 and the monthly calls) will be adjusted accordingly.

[8] Subjects should be reached once a month with no more than 45 days between phone contacts (use Day 42 date as starting reference or last day of contact with the clinic site or Investigator if a second vaccination did not occur).

[9] On Day 21, the immunogenicity – serology blood sample will be collected for the Nab assay and ELISA only. Anti-plant glycan IgE antibodies will only be measured on Days 0 (prior to vaccination), 42, 201, and 386.

[10] Blood and urine samples are to be collected prior to vaccination on the respective day.

[11] Vaccination on Day 0 can proceed prior to receiving the results of the confirmatory SARS-CoV-2 test on Day 0.

ABBREVIATIONS

AE	adverse event
AESI	adverse event of special interest
ANOVA	analysis of variance
BMI	body mass index
BP	blood pressure
CBER	Center for Biologics Evaluation and Research
CCD	cross-reactive carbohydrate determinate
CI	confidence interval
CMI	cell-mediated immune (response)
eCRF	electronic case report form
ELISA	enzyme-linked immunosorbent assay
ELISpot	enzyme-linked immuno spot assay
FDA	Food and Drug Administration
GCP	good clinical practice
GMFR	geometric mean fold rise or seroconversion factor
GMT	geometric mean titer
HR	heart rate
IB	Investigator's Brochure
ICF	informed consent form
ICH	International Council for Harmonisation
IDMC	Independent Data Monitoring Committee
IEC	independent ethics committee
IFN-	interferon gamma
IM	intramuscular
IRB	institutional review board
IRT	interactive response technology
ITT	intention-to-treat
MedDRA	Medical Dictionary for Regulatory Activities
Nab	neutralizing antibody
OT	oral temperature
PBMC	peripheral blood mononuclear cell
pIMD	potential immune-mediated disease
PP	per protocol
RR	respiratory rate
SAE	serious adverse event
SAP	statistical analysis plan
SAS	safety analysis set
SAS®	Statistical Analysis System®

SC	seroconversion
VED	vaccine-enhanced disease
VLP	virus-like particle
WHO	World Health Organization
US	United States

INTRODUCTION

Background

A cluster of pneumonia cases of unknown aetiology was identified in the city of Wuhan in Hubei province of China in December 2019 [Zhu 2020]. The clinical manifestations included fever, cough, and fatigue, while other symptoms include sputum production, headache, haemoptysis, diarrhoea, dyspnoea, and lymphopenia [Lake 2020]. Clinical features revealed by a chest computed tomography (CT) scan presented as pneumonia, however, there were abnormal features such as acute respiratory distress syndrome, acute cardiac injury, and incidence of multiple organ failure that led to death in some cases [Chen 2020]. The symptoms of the disease were more severe in older age groups with comorbidities, while hypertension, type 2 diabetes, asthma and chronic obstructive pulmonary disease (COPD) were also identified as risk factors [Liu 2020a, Yang 2020]. A novel coronavirus, Severe Acute Respiratory Syndrome Coronavirus2 (SARS-CoV-2), formerly known as the 2019 novel Coronavirus (2019-nCoV), was identified as the agent that caused the pneumonia outbreak, and the disease was subsequently named ‘coronavirus disease 2019’, or COVID-19 [Guan 2020, Zhu 2020]. The rapidly evolving situation with SARS-CoV-2 infection in China and spread of the disease across many countries prompted the World Health Organization (WHO) to declare a pandemic in March 2020 [WHO 2020].

Coronaviruses are frequent causes of respiratory infections where six major species are known to cause human infections besides the SARS-CoV-2. Previous outbreaks of coronavirus infections include the severe acute respiratory syndrome (SARS) and the Middle East respiratory syndrome (MERS), which have been characterized as a great public health threat [Liu 2020]. In 2002– 2003, severe acute respiratory syndrome coronavirus (SARS-CoV) emerged from bat and palm civet and infected over 8 000 people and caused about 800 deaths [Cheng 2007]. In 2012, MiddleEast respiratory syndrome coronavirus (MERS-CoV) was discovered as the causative agent of a severe respiratory syndrome in Saudi Arabia, with nearly 2 500 confirmed cases and 858 deaths. It remains endemic in Middle East, and dromedary camel is thought to be the zoonotic reservoir host of MERS-CoV [Memish 2020]. Less virulent coronavirus species include NL63, 229E, OC43 and HKU14 that account for 10 to 30 % of common cold cases, with only occasional spreading to the lower respiratory tract [Paules 2020, Su 2016].

Coronaviruses are enveloped positive-sense RNA viruses ranging from 60 nm to 140 nm in diameter with spike-like projections on its surface giving it a crown-like appearance under the electron microscope. SARS-CoV-2 particles consist of a helical nucleocapsid structure, formed by the association between nucleocapsid (N) phosphoproteins and the viral genomic RNA, whichis surrounded by a lipid bilayer where three types of structural proteins are inserted: the spike (S), the membrane (M), and the envelope (E) proteins. SARS-CoV-2 uses its S glycoprotein, a main target for the neutralizing antibody, to bind to its receptor angiotensin-converting enzyme 2 (ACE2), and mediate membrane fusion and virus genome release into the cytosol of an infected cell. Each monomer of trimeric S protein is about 180 kDa, and contains two subunits, S1 and S2, mediating attachment and membrane fusion, respectively [Chen 2020a, Ou 2020].

Since there is currently no effective treatment available for coronavirus infections, significant efforts have been made to the development of vaccines and therapeutic drugs. Most of therapeutic approaches that are currently being tested are based on repurposing the therapeutic agents previously designed for other applications. These agents can either directly target the virus replication cycle or aim boosting the innate antiviral immune responses or attenuating the damage induced by dysregulated inflammatory responses [Ahn 2020, Stebbing 2020].

Vaccination is the most effective strategy for preventing infectious diseases since it appears to be more cost-effective than therapeutic drugs and reduces morbidity and mortality without causing long-lasting undesirable effects. Although three human coronaviruses emerged worldwide over the past two decades, causing considerable threat to global health, there is still no approved vaccines for human coronaviruses. Research groups around the world are accelerating the development of COVID-19 vaccines using various approaches [Ahn 2020, Lu 2020].

A protective role of both humoral and cell-mediated immune responses against coronaviruses has been suggested [Enjuanes 2016]. Antibody responses against S protein, the most abundant protein of SARS-CoV-2, have been shown to protect from the infection [Du 2009, Ou 2020].
The SARS-CoV outbreak of 2002–2003 demonstrated that while being protective, antibody titers were found to be relatively short-lived in convalescent patients, dropping substantially by two years post-infection [Tang 2011]. In contrast, T cell responses remained detectable up to 11 years post-infection, and thus showed a great promise for SARS-CoV vaccine development [Ng 2016]. Current evidences strongly indicated that Th1 type response is a key for successful control of SARS-CoV and MERS-CoV and probably also SARS-CoV-2, as well as for minimising the risk of severe post-vaccination adverse reactions [Honda-Okubo 2015]. These observations direct the vaccine development efforts to prevent SARS-CoV-2 infection.

A number of biopharmaceutical companies and academic laboratories are in the race to develop the prophylactic vaccine against SARS-CoV-2 by using several platforms including mRNA, DNA, inactivated virus, live viral vectors, recombinant proteins, peptides, and virus-like particles (VLPs) [Thanh Le 2020]. The vaccine candidate developed by Medicago R&D Inc. (Medicago) is plant-derived VLPs expressing the S protein of SARS-CoV-2 integrated into lipid bilayer of the nanoparticles that resemble the native structure of SARS-CoV-2 viruses, allowing them to be easily recognized by the immune system. In contrast to the native viruses, these plant-derived VLPs lack viral genetic material, and therefore are non-replicative and non-infections [Medicago 2020]. Based on positive results of pre-clinical testing of the vaccine candidate, Medicago plans to initiate this clinical study.

Background of the Investigational Product

Medicago has developed a plant-based system (*Nicotiana benthamiana*) for transient expression of recombinant viral proteins, and this system has been used to produce VLPs bearing the SARS-CoV-2 S glycoprotein. Previously, Medicago used the same manufacturing platform to produce the Quadrivalent VLP Influenza Vaccine intended for active immunization for the prevention of influenza disease caused by influenza A subtype viruses and type B viruses contained in the

vaccine. This plant-based platform may be able to address several limitations of the currently predominant vaccine manufacturing processes:

The S protein in the VLPs is based on the genetic sequence of circulating SARS-CoV-2 viruses;

Plant-based VLP manufacturing does not require the use of live viruses. Thus, inactivationby chemicals and then splitting by a detergent before injection into humans are not needed,as is the process for living non-attenuated viruses. These processes are known to influenceantigenicity, and antigens displayed on the VLPs are not impacted by the potentially denaturing treatments;

The plant-based technology used for the Coronavirus-Like Particle COVID-19 Vaccine produces S protein without introducing undesirable mutations that can occur when live coronaviruses or viral vectors are propagated in cell-based production platforms. The S protein is presented on VLPs in a pre-fusion conformation that resembles the native structure of SARS-CoV-2 viruses, allowing it to be easily recognized by the immune system to induce a robust neutralizing antibody response and reduce the risk of vaccine enhanced disease [Lambert 2020]. Very few microbial pathogens can infect both plants andhumans so the risk of exposure to potentially pathogenic adventitious agents is greatly reduced;

Medicago's previous clinical data suggests that plant-made VLP vaccines induce not only antibodies, but also strong CD4+ T cell immunity which may be important for both the persistence of immunity and the provision of better protection [Channappanavar 2014].

Nicotiana benthamiana is a non-transgenic plant that is a distant wild relative from Australia of the tobacco plant, *Nicotiana tabacum*. The transfer vector used to move targeted DNA constructs into the plant cells is the bacterium *Agrobacterium tumefaciens*. This vector then directs the expression of the desirable viral protein. The SARS-CoV-2 S protein is aligned at the plant plasma membrane via a transmembrane domain, and buds out of the plant plasma membrane in the form of VLPs. Thus, the viral S protein is anchored in a lipid bilayer of plant cell origin.

Background of the Adjuvant AS03

The adjuvant AS03 is an established effective adjuvant used in the formulations for Arepanrix™ and Pandemrix™ and is manufactured by GlaxoSmithKline. The AS03-adjuvanted pandemic influenza vaccines have been shown to be more immunogenic than non-adjuvanted vaccines, offering the potential of cross-clade immunity and feasibility of antigen-sparing. High efficacy and effectiveness have been demonstrated for AS03-adjuvanted H1N1 pandemic influenza vaccines in a wide range of populations [Garcon 2012]. Clinical data with AS03-adjuvanted antigen-sparing formulations have shown that immunization against influenza caused by the potential pandemic subtypes H5N1, H1N1, H7N1, H7N9, and H9N2 has demonstrated satisfactory immunogenic potency, as measured by haemagglutinin-inhibition titers, with reduced antigen doses in adults [Baz 2013, Jackson 2015, Lansbury 2017, Leroux-Roels 2007, Madan 2017, Madan 2017a, Madan 2017b, McElhaney 2013, Yang 2013, Yin 2011]. Also,

AS03-adjuvanted H5N1 vaccines were shown to induce cross-clade neutralizing antibody responses [Leroux-Roels 2007] and antibody affinity maturation [Khurana 2018].

Data from clinical trials with over 55 000 vaccinated subjects showed that AS03-adjuvanted influenza vaccines exhibited an acceptable safety profile [Cohet 2019, Garcon 2012, Vaughn 2014]. Increased reactogenicity, both local and general, is consistently noted for AS03-adjuvanted vaccines compared with the corresponding unadjuvanted vaccines [Garcon 2012, Launay 2013, Nolan 2014, Waddington 2010]. Most symptoms were mild to moderate in intensity and of short duration. An increased risk of narcolepsy was observed in some individualsafter the vaccination campaign with Pandemrix™ in 2009-2010. A similar risk of narcolepsy wasnot identified with other non-adjuvanted influenza vaccines or other AS03-adjuvanted vaccines, like Arepanrix™ [Montplaisir 2014, Cohet 2015]. Current data suggest that cases of narcolepsy seen immediately following the 2009-2010 pandemic were the result of an immune cascade, triggered by CD4 T cell cross-reactivity to HA proteins from the H1N1 virus itself and hypocretin.

Background of the Adjuvant CpG 1018

The adjuvant CpG 1018 is an established effective adjuvant used in the formulation for Heplisav-B® and is manufactured by Dynavax. Heplisav-B® is a hepatitis B virus (HBV) vaccine comprised of recombinant, yeast cell-derived hepatitis B surface antigen (HBsAg) and CpG 1018. The CpG 1018 in Heplisav-B® is a synthetic 22-mer phosphorothioate oligodeoxynucleotide (PS ODN) containing an immunostimulatory sequence that is an agonist for the toll-like receptor 9 (TLR9). The desired biological activity of CpG 1018 is to enhance the generation of antibodies to HBsAg by mimicking the immunostimulatory activity of single-stranded viral and bacterial DNA.

Cumulatively, 10 049 subjects (10 038 subjects 18 years of age and older and 11 subjects below 18 years old) and 386 adult subjects with chronic kidney disease 18 years of age and older have received Heplisav-B® in clinical trials. Heplisav-B® was well tolerated in clinical trials in healthy adults [Heplisav-B® 2020]. The reactogenicity profile of Heplisav-B® is similar to Engerix-B (an alum adjuvanted HBV vaccine) in time course and severity. Compared with Engerix-B, Heplisav-B® caused more local reactions and fewer systemic reactions. Common adverse reactions from Heplisav-B® included injection site pain, erythema, and swelling, headache, malaise, myalgia, and fatigue. Serious adverse events, autoimmune adverse events, and deaths were infrequent in the Heplisav-B® clinical program and similar in frequency between Heplisav-B® and Engerix-B. No deaths have been considered by the Investigator to be related to Heplisav-B®. Interim analyses of an ongoing post-marketing study of over 30 000 Heplisav-B® recipients and more than 35 000 Engerix-B recipients has raised no safety concerns. The important identified risks for CpG 1018 are anaphylaxis, bursitis, and vasovagal syncope, and an important potential risk for CpG 1018 is immune-mediated disease. Based on the data from over 40 000 adult vaccinations to date, this potential risk remains “theoretical” as it is for other vaccines.

Pre-clinical Studies

For comprehensive preclinical information regarding the safety and toxicity of the Coronavirus-Like Particle COVID-19 Vaccine, refer to the current Investigator's Brochure (IB).

Clinical Studies

No clinical studies of the Coronavirus-Like Particle COVID-19 Vaccine have been conducted to date.

Overall Rationale for the Study

The Phase 1 study is intended to assess the safety and immunogenicity profile of the Coronavirus-Like Particle COVID-19 Vaccine in healthy adults and unblinded results generated from this study will inform design decisions (i.e. most safe and effective dose level[s] and regimen[s], more effective adjuvant) of a subsequent Phase 2 study.

The study will be a randomized, partially-blinded, prime-boost, staggered dose-escalation Phase 1 study intended to assess the safety, tolerability, and immunogenicity of the Coronavirus-Like Particle COVID-19 Vaccine at three dose levels (3.75 µg, 7.5 µg, and 15 µg VLP) unadjuvanted or adjuvanted with either CpG 1018 or AS03 in healthy adults 18 to 55 years of age, who have been tested for the absence of SARS-CoV-2 antibodies. At each dose level, the vaccine will initially be administered to a small number of subjects. Vaccination of the remaining subjects at the same dose level and the next higher vaccine dose level will be administered with approval of the IDMC. The same process will be followed for the second vaccine administration. All subjects will receive two doses of the same dose level of the vaccine 21 days apart and data collected will be used to determine if one or two doses is/are required of the Coronavirus-Like Particle COVID-19 Vaccine.

All subjects will be followed for a period of 12 months after the second administration of the vaccine for safety and immunogenicity testing at the end of the follow-up period.

OBJECTIVES

Primary Objectives

The primary objectives of this study are:

To assess the safety and tolerability of the Coronavirus-Like Particle COVID-19 Vaccine,as determined by the occurrence(s) of:

Immediate AEs within 30 minutes after each vaccination;

Solicited local and systemic AEs up to 7 days after each vaccination;

Unsolicited AEs SAEs, AEs leading to withdrawal, AESIs, and deaths up to 21 days aftereach vaccination;

Subjects with normal and abnormal urine, haematological, and biochemical values;

To assess the immunogenicity of the Coronavirus-Like Particle COVID-19 Vaccine up to21 days after each vaccination, as determined by:

Neutralizing antibody titers induced by the vaccine against the SARS-CoV-2 virus;

IFN-γ ELISpot response induced by the vaccine against the SARS-CoV-2 virus to assessTh1 type response;

IL-4 ELISpot response induced by the vaccine against the SARS-CoV-2 virus to assessTh2 type response.

Secondary Objectives

The secondary objectives of this study are:

To assess the immunogenicity of the Coronavirus-Like Particle COVID-19 Vaccine up to21 days after vaccination, as determined by the:

IgG and/or IgM (ELISA) titers induced by the vaccine against the SARS-CoV-2 virus;

To assess the immunogenicity of the Coronavirus-Like Particle COVID-19 Vaccine up to201 and 386 days after the first vaccination, as determined by the:

Neutralizing antibody titers induced by the vaccine against the SARS-CoV-2 virus;

IFN-γ ELISpot response induced by the vaccine against the SARS-CoV-2 virus;

IL-4 ELISpot response induced by the vaccine against the SARS-CoV-2 virus;

IgG and/or IgM (ELISA) titers induced by the vaccine against the SARS-CoV-2 virus;

To assess the safety and tolerability of the Coronavirus-Like Particle COVID-19 Vaccine,as determined by the occurrence(s) of:

SAEs, AEs leading to withdrawal, AESIs, and deaths from 22 days after the last vaccination up to the end of the study.

Exploratory Objectives

The exploratory objectives of this study are:

To assess the specific CMI response induced by the Coronavirus-Like Particle COVID-19 Vaccine against the SARS-CoV-2 virus up to 21 days after each vaccination and up to 201and 386 days after the first vaccination, as measured by the percentage of CD4+ T cells expressing functional markers;

To measure the levels of serum IgE antibodies directed to plant-specific glycans prior to vaccination (Day 0), up to 21 days after the second vaccination (Day 42) and up to 201 and386 days after the first vaccination (Day 201 and Day 386);

To measure the occurrence(s) of laboratory-confirmed (virologic or serologic methods) SARS-CoV-2 infection (with or without symptoms) up to the end of the study;

If deemed necessary, to further characterize the immune response and the safety profile ofthe Coronavirus-Like Particle COVID-19 Vaccine.

STUDY ADMINISTRATIVE STRUCTURE

Table 2 Study Administrative Structure

Role	Name and Address
**Sponsor**	Medicago R&D Inc.<br>1020, route de l'Église, bureau 600,<br>Sainte-Foy (Qc) Canada G1V 3V9
Medical Officer:	Brian J. Ward, MD
Medical Monitor:	Alexander Makarkov, MD, Ph.D.
Director, Clinical Studies:	Sébastien Soucy, CCRP
Medical Writer	Jiwanjeet Dhaliwall, MSc

STUDY DESIGN AND RATIONALE

Overview of Study Design

This is a randomized, partially-blind, prime-boost, staggered dose-escalating study with approximately 180 healthy male and female subjects 18 to 55 years of age, who have been tested for the absence of SARS-CoV-2 antibodies. The subjects will be enrolled into nine treatment groups with 3 dose levels (3.75 µg, 7.5 µg, and 15 µg VLP) to receive Coronavirus-Like Particle COVID-19 Vaccine unadjuvanted or adjuvanted with CpG 1018 or AS03:

Dose Level 1: Sixty subjects will be randomized to receive two injections of dose level 1 (3.75 µg) of the Coronavirus-Like Particle COVID-19 Vaccine unadjuvanted or adjuvanted with CpG 1018 or AS03 on Days 0 and 21 (one injection on each of these days).

Dose Level 2: Sixty subjects will be randomized to receive two injections of dose level 2 (7.5 µg) of the Coronavirus-Like Particle COVID-19 Vaccine unadjuvanted or adjuvanted with CpG 1018 or AS03 on Days 0 and 21 (one injection on each of these days)

Dose Level 3: Sixty subjects will be randomized to receive two injections of dose level 3 (15 µg) of the Coronavirus-Like Particle COVID-19 Vaccine unadjuvanted or adjuvanted with CpG 1018 or AS03 on Days 0 and 21 (one injection on each of these days).

For each dose level group, the three-day safety data after vaccination of the first 10 % of subjects enrolled (6 subjects) in the group will be collected and reviewed by the IDMC, prior to permitting vaccination of the next 30 % of subjects enrolled (18 subjects) in the dose level groupand escalating to vaccination of subjects at the next higher dose level (until reaching the highest dose level). The three-day safety data after vaccination of these 18 subjects in the group will be collected and reviewed by the IDMC, prior to permitting vaccination of the remaining subjects in the dose level group. The two planned three-day safety data reviews by the IDMC for each dose level will occur after each vaccination in the study. The first 10 % of subjects in each dose level

group may be vaccinated in the same day at the clinical sites. In addition, the vaccinations of the first 6 subjects at the lowest dose level will be staggered so that each vaccination must be performed at least 30 minutes apart. After IDMC recommendation to proceed is received, no more than 18 subjects (from any dose level group) will be dosed at a clinical site per day to respect physical distancing.

The subjects will be enrolled in a 1:1:1:1:1:1:1:1:1 ratio into one of nine treatment groups, as presented in Table 3.

Table 3 Study Treatment Groups

Dose Level Group	Treatments	Treatment Group	No. of Subjects	Dose Level
1	Coronavirus-Like Particle COVID-19 Vaccine	1	20	3.75 µg
	Coronavirus-Like Particle COVID-19 Vaccine + CpG 1018	2	20	3.75 µg
	Coronavirus-Like Particle COVID-19 Vaccine + AS03	3	20	3.75 µg
2	Coronavirus-Like Particle COVID-19 Vaccine	4	20	7.5 µg
	Coronavirus-Like Particle COVID-19 Vaccine + CpG 1018	5	20	7.5 µg
	Coronavirus-Like Particle COVID-19 Vaccine + AS03	6	20	7.5 µg
3	Coronavirus-Like Particle COVID-19 Vaccine	7	20	15 µg
	Coronavirus-Like Particle COVID-19 Vaccine + CpG 1018	8	20	15 µg
	Coronavirus-Like Particle COVID-19 Vaccine + AS03	9	20	15 µg

All subjects will receive IM two doses of the vaccine 21 days apart and the data collected will beused to determine if one or two doses is/are required.

Subjects will be screened up to seven days in advance of the first vaccine administration and will demonstrate a satisfactory baseline medical assessment by history, general physical examination, haematological, biochemical, and serological analysis. In addition, a test for the absence of SARS-CoV-2 antibodies will be performed at screening and prior to vaccination on Day 0, as well as on Day 42, Day 201, and Day 386. On Days 0 and 21, vaccine administration will occur. Telephone contacts will be made one day and eight days after each vaccine administration as well as monthly after Day 42 until the final study visit (Day 386), specifically for review of the subject's safety and concomitant medication data. Visits to the Investigator site will occur three days after each vaccination (Day 3 and Day 24) for key safety assessments and 21 days after each vaccination (Day 21 and Day 42) for key safety and immunogenicity assessments. Subjects will have monthly telephone calls after Day 42 and will return to the Investigator site on Day 201and Day 386 for the safety follow-ups and immunogenicity assessments (6-month and 12-month follow-ups).

Rationale of Study Design

Randomization

Randomization will be used to minimize bias in the assignment of subjects to treatment groups within each dose level group, to increase the likelihood that known and unknown subject

attributes (e.g. demographic and baseline characteristics) are evenly balanced across treatment groups and to enhance the validity of statistical comparisons across treatment groups.

Blinding

A partially-blinded trial design consisting of both observer-blind and open-label elements will be utilized in this study. Observer-blinded treatment trial design elements will be used to reduce potential bias during data collection and evaluation of the study endpoints. Details of who will remain blinded during the study are presented in Section 6.2.

Open-label trial design elements will be used to allow a small number of Medicago personnel to remain unblinded throughout the study (see Section 6.2) to make informed design decisions of the Phase 2 study based on the Day 42 data analysis (see Section 12.3) results, without having to wait until after the end of the follow-up period for study completion.

Open-label trial design elements will also allow all study staff to remain unblinded to dose level in order to manage the staggered ascending dose escalation and IDMC coordination.

Dose Selection and Dosage Regimen

In this study, three dose levels (3.75 µg, 7.5 µg, and 15 µg) of the Coronavirus-Like Particle COVID-19 Vaccine will be tested in a two-dose regimen, with and without an adjuvant, to eventually establish a safe and effective treatment.

Route of Administration

The route of administration used for the Coronavirus-Like Particle COVID-19 Vaccine is the intramuscular route, specifically in the deltoid muscle of the arm, since it is a more viable route of administration (compared to an oral route) and has better absorption compared to the subcutaneous route.

Study Duration

The duration of this study will be approximately eight months, from screening procedures up to the end of the study.

STUDY POPULATION

Inclusion Criteria

Subjects must meet all of the following inclusion criteria at the Screening (Visit 1) and/or Vaccination (Visit 2) visits to be eligible for participation in this study; no protocol waivers are allowed. All Investigator assessment-based judgements must be carefully and fully documented in the source documents:

Subjects must have read, understood, and signed the informed consent form (ICF) prior to participating in the study; subjects must also complete study-related procedures and communicate with the study staff at visits and by phone during the study;

At the Screening visit (Visit 1), male and female subjects must be 18 to 55 (has not yet had his/her 56th birthday) years of age, inclusive;

At Screening (Visit 1) and Vaccination (Visit 2), subject must have a body mass index (BMI) of ≥ 18.5 and < 25 kg/m^2;

Subjects are considered by the Investigator to be reliable and likely to cooperate with the assessment procedures and be available for the duration of the study;

Subjects must be healthy (no clinically significant health concerns) as determined by medical history, physical examination, vital signs, and clinical laboratory tests. Investigatordiscretion will be permitted with this inclusion criterion;

Female subjects of childbearing potential must have a negative serum pregnancy test resultat Screening (Visit 1) and a negative urine pregnancy test result at Vaccination (Visit 2 andVisit 4).

Non-childbearing females are defined as:

Surgically-sterile (defined as bilateral tubal ligation, hysterectomy or bilateral oophorectomy performed more than one month prior to the first study vaccination); or

Post-menopausal (absence of menses for 12 consecutive months and age consistentwith natural cessation of ovulation);

Female subjects of childbearing potential must use an effective method of contraception forone month prior to vaccination (Visit 2) and agree to continue employing highly effective birth control measures for at least one month after the last administration of the investigational product (or in the case of early termination, she must not plan to become pregnant for at least one month after her last study vaccination). The following relationshipor methods of contraception are considered to be highly effective:

Combined (estrogen and progestogen containing) hormonal contraception associatedwith inhibition of ovulation:

Oral;

Intravaginal;

Transdermal;

Progestogen-only hormonal contraception associated with inhibition of ovulation:

Oral;

Injectable;

Implantable;

Intra-uterine device with or without hormonal release;

Vasectomised partner, provided that this partner is the sole sexual partner of the studyparticipant and that the vasectomised partner has received a medical assessment of thesurgical success;

Credible self-reported history of heterosexual vaginal intercourse abstinence prior toand for at least one month after the last administration of the investigational product;

Female partner.

Exclusion Criteria

Subjects who meet any of the following criteria at the Screening (Visit 1) and/or Vaccination (Visit 2) visits will not be eligible for participation in this study; no protocol waivers are allowed. All Investigator assessment-based judgements must be thoroughly documented in the source documents:

Clinically significant acute or chronic pulmonary (including but not limited to chronic obstructive pulmonary disease or asthma), cardiovascular (including but not limited to arterial hypertension, coronary artery disease, or congestive heart failure), renal, metabolic (including but not limited to type 2 diabetes), or other somatic (medical) or neuropsychiatricillness within 3 months prior to Screening (Visit 1), excessive alcohol use or drug abuse, as determined by medical history, physical examination, vital signs, and clinical laboratory tests.

Investigator discretion is permitted with this exclusion criterion and must be carefully andfully documented in the source documents;

Any unexplained clinical syndrome (including, but not limited to, chronic fatigue syndrome, Raynaud's syndrome, unexplained pain syndromes such as fibromyalgia, etc.);

Acute disease defined as presence of any moderate or severe acute illness with or without afever within 48 hours prior to Vaccination (Visit 2);

Prior exposure to SARS-CoV-2 as determined by detection of IgM or IgG antibodies against SARS-CoV-2 at Screening (Visit 1) and Vaccination (Visit 2);

Any confirmed or suspected current immunosuppressive condition or immunodeficiency, including cancer, human immunodeficiency virus (HIV), hepatitis B or C infection (subjectswith a history of cured hepatitis B or C infection without any signs of immunodeficiency at present time are allowed). Investigator discretion is permitted with this exclusion criterion;

Current autoimmune disease (such as rheumatoid arthritis, systemic lupus erythematosus or multiple sclerosis). Investigator discretion is permitted with this exclusion criterion, and subjects may be eligible to participate with appropriate written justification in the source document (i.e. subjects with a history of autoimmune disease who are disease-free without treatment for three years or more, or on stable thyroid replacement therapy, mild psoriasis [i.e. a small number of minor plaques requiring no systemic treatment], etc.);

Administration of any medication or treatment that may alter the vaccine immune responses,such as:

Systemic glucocorticoids within one month prior to Vaccination (Visit 2). Inhaled, nasal, dermal, intraarticular, ophthalmic and other topical glucocorticoids are permitted;

Cytotoxic, antineoplastic, or immunosuppressant drugs - within 36 months prior to Vaccination (Visit 2);

Any immunoglobulin preparations or blood products, blood transfusion – within6 months prior to Vaccination (Visit 2);

Administration of any vaccine within 30 days prior to Vaccination (Visit 2); planned administration of any vaccine during the study (up to blood sampling on Day 42 of the

study). Immunization on an emergency basis during the study will be evaluated on case-by-case basis by the Investigator;

Administration of any other SARS-CoV-2 / COVID-19, or other experimental coronavirus vaccine at any time prior to or during the study;

Known current or previous laboratory-confirmed SARS-CoV-1 or SARS-CoV-2 / COVID-19 infection as documented by a positive PCR test or positive serological test;

Subjects at high risk of contracting SARS-CoV-2/COVID-19 infection, including but not limited to the individuals with known close contact of anyone with laboratory-confirmed SARS-CoV-2 / COVID-19 infection within 2 weeks prior to vaccine administration, those who traveled outside Canada for any duration within 30 days before the study vaccination, healthcare workers in acute care hospitals, rehabilitation hospitals, mental health hospitals, long term care facilities, emergency departments, and others who through their work must come into close face-to-face contact with their clients or patients (including, but not limitedto, physiotherapists, dentists, hair dressers/barbers, etc.);

Use of any investigational or non-registered product within 30 days or 5 half-lives, whichever is longer, prior to Vaccination (Visit 2) or planned use during the study period;

Have a rash, dermatological condition, tattoos, muscle mass, or any other abnormalities at injection site that may interfere with injection site reaction rating. Investigator discretion will be permitted with this exclusion criterion;

Use of any prescription medication on a regular basis for more than 30 continuous days within the last 3 months, with the following exceptions:

Medications listed among the methods of contraception in inclusion criterion 7;

Subjects on stable thyroid replacement therapy, as noted in exclusion criterion 6.

Use of any prescription antiviral drugs with the intention of COVID-19 prophylaxis, including those that are thought to be effective for prevention of COVID-19 but have notbeen licensed for this indication, within one month prior to Vaccination (Visit 2);

Use of prophylactic medications (e.g. antihistamines [H1 receptor antagonists], nonsteroidalanti-inflammatory drugs [NSAIDs], systemic and topical glucocorticoids, non-opioid and opioid analgesics) within 24 hours prior to the Vaccination (Visit 2) to prevent or pre-empt symptoms due to vaccination;

History of allergy to any of the constituents of the Coronavirus-Like Particle COVID-19 Vaccine, vaccine adjuvants, or tobacco;

History of anaphylactic allergic reactions to plants or plant components (including fruits andnuts);

Currently smoke more than 10 cigarettes per month or any use of vaping products;

Subjects with a history of Guillain-Barré Syndrome;

Any female subject who has a positive or doubtful pregnancy test result prior to vaccinationor who is lactating;

Subjects identified as an Investigator or employee of the Investigator or clinical site withdirect involvement in the proposed study, or identified as an immediate family member (i.e. parent, spouse, natural or adopted child) of the Investigator or employee with direct involvement in the proposed study, or any employees of Medicago.

Prior and Concomitant Therapy

New or changed medications reported by the subject after the first vaccination and through to the end of the study will be recorded in the source documents as a concomitant medication as per the conditions outlined in the next paragraph. Since AEs may be secondary to new medications, the Investigator will explore the reasons for the change or for the new medication intake and document these AEs, if any.

Concomitant medications must be reported (reason for use, dates of administration, dosage, and route) if the use meets the following conditions:

Within 30 days preceding vaccination: any treatments and/or medications specifically contraindicated (e.g. vaccines, any immunoglobulins or other blood products, or any immune modifying drugs, etc.);

From randomization to Day 42, inclusive: any medication (including, but not limited to, over-the-counter medicines such as aspirin or antacids), vitamins, and mineral supplements;

From Day 43 to the end of the study, inclusive: any concomitant medication(s) administered to treat an AESI, SAE, or AE leading to withdrawal; any concomitant medication used to treat an AE that occurred before Day 42 and that is still being usedafterwards (i.e. on-going use);

Any concomitant medication used to treat conditions reported as medical history;

Any investigational medication or vaccine; any vaccine not foreseen in the study protocol.

Prohibited Therapy

The following medications or therapies are prohibited during the conduct of this study:

Administration of any vaccine (other than the study vaccine) up to blood sampling onDay 42 of the study. Immunization on an emergency basis during the study will be evaluated on case-by-case basis by the Investigator;

Use of any investigational or non-registered product during the study period. Subjects may not participate in any other investigational or marketed drug study while participating in thisstudy until after the study;

Administration of any medication or treatment that may alter the vaccine immune responses,such as:

Systemic glucocorticoids;

Cytotoxic, antineoplastic, or immunosuppressant drugs;

Any immunoglobulin preparations or blood products, or blood transfusion.

Administration of such medications should be specifically avoided up to blood sampling onDay 42 of the study. Use of any medication or treatment that may alter the vaccine immuneresponses due to a medical need during the study will be evaluated on case-by-case basis bythe Investigator;

Use of prophylactic medications (e.g. antihistamines [H1 receptor antagonists], nonsteroidalanti-inflammatory drugs [NSAIDs], systemic and topical glucocorticoids, non-opioid and

opioid analgesics) within 24 hours prior to administration of the second dose of the vaccine(Visit 4) to prevent or pre-empt symptoms due to vaccination;

Use of any prescription antiviral drugs with the intention of COVID-19 prophylaxis, including those that are thought to be effective for prevention of COVID-19 but have notbeen licensed for this indication, during the study.

Given that an important objective of this study is to evaluate the tolerability of the study vaccine, the use of prophylactic medications to prevent or pre-empt symptoms due to vaccination is specifically prohibited up to seven days after each vaccination (end of collection of solicited symptoms). A prophylactic medication is a medication administered in the absence of ANY symptom and in anticipation of a reaction to the vaccination (e.g. an antipyretic is considered to be prophylactic when it is given in the absence of fever or any other symptoms, to prevent fever from occurring, vitamins used to boost immune system, etc.).

If one of the first three criteria are met by a subject during the study (after vaccination), the subject may still remain in the study however the inclusion of the subject’s data within the PP set, ITT set, or SAS may be impacted.

TREATMENT ALLOCATION AND BLINDING

Randomization

Potential study subjects will be screened and assigned a six-digit subject number. Once all screening procedures, including Day 0 pre-randomization procedures, have been completed and the study eligibility is confirmed by the Investigator, the randomization numbers will be allocated to subjects within the appropriate treatment group by the randomization system. If a subject is randomized in error (i.e. does not meet eligibility criteria) and has not been vaccinated,then another eligible subject can be randomized to replace this subject.

Once a randomization number has been assigned, it will not be re-used for any reason. No subjects will be randomized into the study more than once. If a randomization number is allocated incorrectly, no attempt will be made to remedy the error once the study vaccine has been dispensed: the subject will continue on the study with the assigned randomization number and associated treatment. The study staff will notify the Sponsor Contact as soon as the error is discovered without disclosing the study vaccine administered. Admission of subsequent eligible subjects will continue using the next unallocated number in the sequence.

Subjects will be enrolled into one of nine treatment groups, based on a computer-generated randomization schedule prepared by or under the supervision of Medicago before the study. The randomization number and treatment will be recorded along with the six-digit subject number for each subject in the investigational product accountability log.

Blinding

This is a partially-blinded study: the subjects, the Investigators, and all personnel involved in the clinical conduct of the study (except the staff involved in the preparation and administration of the study vaccine, the quality assurance auditor, and quality control reviewers), Medicago

clinical staff and medical staff involved in safety evaluations (e.g. causality assessments), and all personnel involved in sample analysis at the central and testing (Nab, ELISA, ELISpot, and flow cytometry) laboratories will not have access to treatment allocation (i.e. randomization codes) for the entire duration of the study. Partially-blinded trial design will also allow all study staff to remain unblinded to dose level only in order to manage the staggered ascending dose escalation and IDMC coordination.

The IDMC and the independent statistician involved in the preparation of the safety data summary for the IDMC reviews will have access to or be aware of treatment allocation (i.e. be “unblinded”). The IDMC will be reviewing safety data during the study and will be making recommendations throughout the study.

In addition, select personnel of the third-party statistical team will have access to treatment allocation (i.e. be “unblinded”) for the entire duration of the study since these personnel will be involved in the Day 21 data transfer and/or the Day 42 data analysis (see Section 12.3).

A small number of Medicago personnel will have access to the Day 21 data and the Day 42 data analysis (i.e. be “unblinded” to results and/or treatment allocation) and will be able to look at data throughout the study. The Medicago personnel will include senior personnel in Scientific and Medical Affairs, Biostatistics, Safety, Product Development, and Regulatory Affairs. The unblinded procedure will be strictly followed, and the process of unblinding and the selected individuals will be documented in writing.

Since there will be differences in the Coronavirus-Like Particle COVID-19 Vaccine and adjuvant preparations (e.g. possibly physical appearance), the site staff involved in the preparation and administration of the treatments will not be involved in any activity that could introduce a bias, such as the evaluations of reactogenicity or AEs experienced by the subjects following vaccination.

Any code break will be documented and reported to Medicago (or its designee) in a timely manner. In a medical emergency, the Investigator may unblind the treatment for that subject without prior consultation with the Sponsor. In such an event, the Investigator will need to contact the responsible Medical Monitor as soon as possible after the unblinding to discuss the case. The treatment allocation should not be disclosed by the Investigator to the responsible Medical Monitor.

The third-party statistical team will receive safety and immunogenicity data after Day 21 (last subject) and will perform data analysis after Day 42 (last subject). The data transfer and data analysis will involve select individuals from the third-party statistical team and Medicago who will be unblinded (as described above). The Day 21 and Day 42 data analysis will allow discussions of the clinical data to inform and finalize design decisions (i.e. most effective dose level, more effective adjuvant) of the subsequent Phase 2 study, without having to wait until after the end of the follow-up period for study completion.

DOSAGE AND ADMINISTRATION

Subjects will receive two IM injections 21 days apart (Day 0 and Day 21), into the deltoid region of the alternating arm (each arm will be injected once), of their assigned vaccine:

Group 1: 3.75 µg of the Coronavirus-Like Particle COVID-19 Vaccine unadjuvanted, or

Group 2: 3.75 µg of the Coronavirus-Like Particle COVID-19 Vaccine adjuvanted withCpG 1018, or

Group 3: 3.75 µg of the Coronavirus-Like Particle COVID-19 Vaccine adjuvanted withAS03, or

Group 4: 7.5 µg of the Coronavirus-Like Particle COVID-19 Vaccine unadjuvanted, or

Group 5: 7.5 µg of the Coronavirus-Like Particle COVID-19 Vaccine adjuvanted withCpG 1018, or

Group 6: 7.5 µg of the Coronavirus-Like Particle COVID-19 Vaccine adjuvanted withAS03, or

Group 7: 15 µg of the Coronavirus-Like Particle COVID-19 Vaccine unadjuvanted, or

Group 8: 15 µg of the Coronavirus-Like Particle COVID-19 Vaccine adjuvanted withCpG 1018, or

Group 9: 15 µg of the Coronavirus-Like Particle COVID-19 Vaccine adjuvanted withAS03.

The volume of each injection will be 0.25 to 0.75 mL, depending on the presence or absence of adjuvant.

SPECIMENS AND CLINICAL SUPPLIES

Management of Samples

Blood samples for biochemistry, haematology, and serology (for HIV, Hepatitis B, and Hepatitis C) will be collected at screening and/or during the study as well as urine samples for urinalysis. Serum and urine (dipstick or similar) pregnancy testing will be performed for femalesof childbearing potential. Blood samples will also be collected for the testing of SARS-CoV-2 antibodies at screening and during the study.

Blood samples for immunogenicity analysis will be collected during the study. Should a subject be discontinued from the study, a sample will be drawn at the time of the final visit and sent to Medicago for analysis. Bioanalysis of the immunogenicity blood samples will be conducted at central laboratories.

Complete information on the handling, storage, and shipment of all laboratory samples will be described in the study-specific documentation.

Collection of Samples

At screening, Days 0 (prior to study treatment administration), 42, 201, and 386, the test for SARS-CoV-2 antibodies will require a blood sample. However, only the results from screening are needed to confirm eligibility requirements prior to vaccination (i.e. SARS-CoV-2 antibody negative). In addition, subjects who are invited to an unscheduled clinical site visit (see Section 10.1.14) will also have a blood sample collected at this visit to test for SARS-CoV-2 antibodies.

Blood samples for biochemistry and haematology will be collected at screening and Days 3, 21, and 24. Serology tests for HIV, Hepatitis B, and Hepatitis C will be conducted at screening. Sample collected during the screening visit should be performed under fasting conditions (approximately 12 hours) for cholesterol and triglyceride analysis. Note that cholesterol and triglyceride analysis will be performed only at screening. Urine samples will be collected at screening and Days 3, 21, and 24.

Pregnancy testing will also be performed for all females of childbearing potential. A serum pregnancy test will be performed during screening. A urine (dipstick or similar) pregnancy test will be performed at Day 0 prior to randomization and at Days 21 and 42. Women of child bearing potential must have negative serum pregnancy test result from screening and urine pregnancy test result from Day 0 available prior to vaccination.

Blood samples will be collected from each subject prior to study treatment administrations on Days 0 and 21, and on Days 42, 201, and 386 for serological (immunogenicity) and CMI assays. In addition, subjects who are invited to an unscheduled clinical site visit (see Section 10.1.14) will also have additional blood samples collected at this visit for serological (immunogenicity) and CMI assays.

Blood samples for measuring IgE antibody titers against plant-specific glycans will be collected prior to study treatment administration on Day 0, and on Days 42, 201, and 386.

Clinical Supplies

The study center will be provided with or be responsible for the provision of supplies for blood collection and shipment (e.g. aliquot tubes, aliquot labels, aliquot storage, shipping boxes, and accompanying manifests) as described in the study-specific documentation. Sites will be authorized to use their own materials if agreed to by Medicago (or its designee).

TREATMENT COMPLIANCE

Study treatments will be prepared and administered by trained unblinded site staff via IM injection to each subject at each Investigator site. In addition, the Investigator or designated study center personnel will maintain a log of all study treatments dispensed and administered to the subject. Investigational product inventory and accountability will be managed throughout the study by unblinded site staff (details will be provided in the study-specific documentation). If an eligible subject refuses vaccination post-randomization, the reason will be documented in the source and electronic case report form (eCRF).

STUDY EVALUATIONS

Study Procedures

Overview

The Time and Events Schedule: General Information (see Table 1) summarizes the frequencyand timing of scheduled assessments applicable to this study.

All subjects will have blood sampled. Subjects will have blood volumes drawn of approximately 307 mL over a period of 386 days (Table 4).

Table 4 Estimated Blood Volume Drawn

Type of Sample	Volume per Sample (mL)	Number of Samples per Subject									Total Volume of Blood per Subject (mL)
		Visit1	Visit2	Visit3	Visit4	Visit5	Visit6	Visit7	Visit8	Total	
Test for SARS-CoV-2 antibodies	5	1	1				1	1	1	5	25
Biochemistry, haematology, serology (HIV, hepatitis B & C, pregnancy test)	8	1		1	1	1				4	32
Serology for immunogenicity (Nab assay, ELISA, anti-plant glycans IgE antibodies)	10		1		1		1	1	1	5	50
CMI response (PBMC)	40		1		1		1	1	1	5	200
Total volume of blood per subject (mL)		13	55	8	58	8	55	55	55		307

Subjects who are invited to an unscheduled clinical site visit (see Section 10.1.14) will have additional blood samples collected for serological (immunogenicity) and CMI assays as well as the test for SARS-CoV-2 antibodies. This will result in an additional blood draw volume of 55 mL at the visit.

General COVID-19 Precautions at Clinical Sites

Sites participating in this study will have processes in place locally for following recommendations of the local Public Health authorities for the management of COVID-19. Subjects will be asked to follow good hygiene and safe physical distancing measures such as wearing a face mask on site, frequent hand hygiene, and maintaining a physical distance of 2 meters (or 6 feet) from others, except during procedures where staff must come into close proximity of subjects (e.g. vaccination, blood draws, collecting vital sign measurements).

Staff at the clinical sites will also follow the same good hygiene and safe physical distancing measures as the subjects at the site. In addition, the staff will be responsible for disinfecting materials and/or areas between each use by subjects and staff. All staff involved with on-site procedures will have a back-up member who is qualified to perform the same duties / responsibilities in the event that a member of the staff is infected with SARS-CoV-2 or comes into contact with an individual known to have COVID-19 and is restricted to self-isolation and following the recommendations of the local Public Health authorities for the management of COVID-19.

At each clinical site visit and telephone contact, subjects will be asked if they are experiencing any symptoms of COVID-19, or they have tested positive for COVID-19, or have had contact with individuals who are known to be infected with SARS-CoV-2. Moreover, if any of these conditions have occurred, then the subjects will be asked to follow all of the recommended actions (e.g. quarantine, re-testing) by local Public Health authorities. In addition, the subjects will be asked to not visit the clinic site until after they have fully recovered (i.e. no more clinical symptoms) and completed all of the recommended actions (e.g. quarantine, re-testing) by local Public Health authorities.

Screening (Visits 1)

The following procedures will be performed at the initial screening visit (Visit 1):

Review and proceed with the signature of the ICF. The Investigator or his/her designee, will fully inform the subject of the nature and scope of the study, potential risks and benefits of participation, and the study procedures involved and will answer all questions prior to requesting the subject's signature on the ICF. The subject's consent must be obtained prior to performing any study-related procedures; the consent process must be clearly recorded and a copy of the signed ICF retained in the source documents. A copy ofthe ICF must be provided to the subject;

After ICF signed, the following procedures will be performed:

Review the inclusion and exclusion criteria and determine the subject's eligibility to participate in this study. The eligibility must be confirmed by the Investigator after review of all procedures and findings and prior to randomization;

Collect and review demographics (gender, date of birth, age, race, and ethnicity) and body measurements (BMI, weight [kg or lbs], and height [cm or inches]) data. BMI is to be calculated as body weight (kg) divided by the square of height (m); the BMI result willbe rounded to one decimal place using the standard convention. For the measurement of body weight, subjects will be lightly clothed, without shoes;

Collect and review medical history, including the grade of any medical conditions (medical conditions are to be graded using the same scale as for AEs; see Section 13.1.2).The medical history should record significant problems active at the time of screening and present within the preceding six months. Problems that have been clinically inactive for more than six months preceding screening, but which might alter the subject's current

or future medical management, should also be noted (e.g. cancer, autoimmune disease,known mitral valve prolapse or a remote history of a seizure disorder);

Review and record current and previous medication use (up to 30 days prior to studyvaccine administration), with the following exception (refer to exclusion criterion 7):

For subjects who have been administered the following medication or treatment, reviewand record current and previous medication use up to the time period specified for the medication or treatment:

cytotoxic, antineoplastic, or immunosuppressant drugs – within 36 months prior tovaccination;

any immunoglobulin preparations or blood products, blood transfusions – within 6months prior to vaccination;

Perform a history- or symptom-directed physical examination. The physical examinationwill be performed by the Investigator or sub-Investigator;

Perform a vital signs measurement, including resting blood pressure (BP), heart rate (HR), respiratory rate (RR), and oral temperature (OT). OT will be collected in degrees Celsius or Fahrenheit using a digital thermometer. The OT measurement should not be collected immediately following consumption of a hot or cold beverage or after smoking.The BP will be taken after the subject has been in a seated position as per the site's standard procedure. The BP should be taken by cuff (manual or automated are both acceptable). BP, HR, and RR may be repeated once if judged necessary. All measurements (including any repeats) will be recorded in the source documents. Inclusion of subjects with an out-of-range BP or HR or RR measurement will be based onthe Investigator's judgment;

Collect screening blood samples for biochemistry, haematology, and serology (HIV, Hepatitis B, and Hepatitis C screening) for analysis as well as to test for SARS-CoV-2antibodies. Sample blood collection at the screening visit should be performed under fasting conditions (approximately 12 hours) for cholesterol and triglyceride analysis;

Perform urinalysis on all subjects;

Perform serum pregnancy testing for female subjects of childbearing potential at screening.

Dose 1 Vaccination (Day 0) (Visit 2)

Emergency equipment must be available on site and appropriate treatment must be instituted as soon as possible in the event of anaphylaxis or any other immediate hypersensitivity reaction. The Investigator will be on-site on vaccine administration days and for the duration of the observation period (minimum of 30 minutes after vaccination) for the last subject dosed on that day. The Investigator will be available on call for the remainder of the study. A physician shouldbe immediately available at the clinical site to administer treatment or to apply procedures for any immediate AEs/SAEs.

Prior to Vaccination

If the subject meets the criterion for the absence of SARS-CoV-2 antibodies at screening,the following procedures will be performed:

Record changes in medical history and medications and confirm that the subject continues to meet all inclusion and no exclusion criteria;

Perform a vital signs measurement, including resting BP, HR, RR, and OT. OT will be collected in degrees Celsius or Fahrenheit using a digital thermometer. The OT measurement should not be collected immediately following consumption of a hot or coldbeverage or after smoking. The BP will be taken after the subject has been in a seated position as per the site's standard procedure. The BP should be taken by cuff (manual or automated are both acceptable). BP, HR, and RR may be repeated once if judged necessary. All measurements (including any repeats) will be recorded in the source documents. Inclusion of subjects with an out-of-range BP or HR or RR measurement willbe based on the Investigator's judgment;

Measure the BMI; for this visit, only weight will be measured while the height will be obtained from that measured at the initial screening visit. The BMI result will be roundedto one decimal place using the standard convention. For the measurement of body weight,subjects will be lightly clothed, without shoes;

Collect a urine sample for female subjects of childbearing potential and perform a urinedipstick (or similar) pregnancy test. No study vaccine must be administered to a childbearing potential female until a negative result is obtained and documented;

If the subject is judged eligible for the study and is still willing to participate in the study, randomize the subject into the study;

After confirmation of eligibility, collect baseline blood samples for immunogenicity (serology [Nab assay, ELISA, and plant-specific glycans IgE antibodies] and CMI) assessments and collect a blood sample to confirm the absence of SARS-CoV-2 antibodies; prepare and store these samples until shipment to the analytical laboratory.

Vaccination

Once all pre-vaccination procedures have been completed and subject eligibility determined, the subject will be randomized and the study vaccine will be administered IM into the deltoid muscle of the non-dominant (if possible) arm, as described in the Investigational Product Management Manual. In order to prevent possible confounding of vaccination site reactions, whenever possible, blood samples will not be collected from the same arm as the one used for vaccination. The arms used, both for blood collection and vaccination, will be documented in the source documents. A 23 gauge needle of at least one inch or 2.5 cm in length can be used for vaccination. The preparation and administration of the study vaccine will be performed by an unblinded site staff member.

Thirty Minutes Post-vaccination

The post-vaccination observations will be performed by a blinded site staff member. The following safety observation procedures will be performed for all subjects immediately following study vaccine administration:

Subjects will remain in the clinic for at least 30 minutes post-vaccination for observation. The observation period will include an assessment of immediate solicited local and systemic AEs. Solicited local and systemic AEs occurring within 30 minutes post- vaccination will be recorded in the subject diary and corresponding eCRF. All unsolicited AEs occurring within 30 minutes post-vaccination will be recorded in the subject source and AE eCRF. Any unusual signs or symptoms reported during the initial 30 minutes post-vaccination will result in continued close monitoring. Based on their condition, subjects may be asked to remain in the clinic for their safety for more than 30 minutes after vaccination (the reason will be recorded in the source documents). All data (including the assessment of solicited local and systemic AEs) will be recorded in the source documents during and after the observation period. Refer to Section 10.2.1 for details regarding the assessment of AEs and/or solicited local and systemic AEs;

During the observation period, subjects will be provided with a measurement device template (Section 19.1) for measuring (in mm) solicited local AEs of erythema (redness) and swelling and an oral digital thermometer for recording daily temperature (in degrees Celsius or Fahrenheit). Subjects will also be provided with a diary (Section 19.2) and willbe shown how to enter their data in the diary. Each subject will be provided with the following instructions on the measurements they are to make:

How to collect his/her OT in degrees Celsius or Fahrenheit with the provided digital thermometer:

From the evening of Day 0 to the evening of Day 7, the subject will measure his/her OTat approximately the same time each evening and will record the results;

The OT should not be collected immediately following consumption of a hot or coldbeverage or after smoking.

The subject is to also take his/her OT if he/she feels feverish and to record the highest temperature of the day. In the event that a temperature ≥ 38.0 °C or ≥ 100.4 °F is obtained, the subject will be allowed to take over-the-counter antipyretics (e.g. acetaminophen/paracetamol, aspirin, naproxen, or ibuprofen) and will be advised to increase the frequency of OT measurements to approximately every four hours, untilhe/she is no longer febrile (fever is defined as a temperature of ≥ 38.0 ºC or ≥ 100.4 °F). The subject is to document medication intake, which will be reviewed bythe site personnel;

How to measure any solicited local AEs, including erythema (redness) and swelling diameter at the injection site using the measurement template supplied for this purpose;subjects will also be requested to evaluate pain at the injection site. Local AEs will be assessed every day starting in the evening of Day 0 and up to the evening of Day 7 and

the results will be recorded. The severity of solicited local AEs will be graded accordingto the Food and Drug Administration (FDA) Guidance for Industry: Toxicity Grading Scale for Healthy Adult and Adolescent Volunteers Enrolled in Preventive Vaccine Clinical Trials [FDA 2007], as presented in Table 5.

How to grade, on a daily basis from the evening of Day 0 through to the evening of Day 7, each of the solicited systemic AEs and their severity (as per the same guidanceused for solicited local AEs; see Table 5) [FDA 2007] and to record the worst grade ofthe day for each of these solicited systemic AE. The instructions will include how to examine and grade swelling in the neck, axilla, groin, and chest wall and to record anyunusual feeling and/or swelling;

Subjects will be advised that they will be asked about the occurrence of any symptoms orevents requiring medical attention and the use of any concomitant medication during the 21-day post-vaccination period, after each vaccination, and until the end of the study. Subjects will also be provided with a memory aid (Section 19.3) to record unsolicited AEs and any concomitant medication use and will be shown how to enter their data in thememory aid;

Subjects will be instructed to contact the clinical site for any unsolicited AEs and/or solicited local and systemic AEs of greater than Grade 2 (moderate). Based on their condition, the Investigator may request that the subject return to the clinic for evaluation;

Subjects will be advised on emergency contact information and instructions for contacting study personnel. Subjects will be advised to immediately contact the Investigator (or his/her designee) in the event of an SAE or a medical emergency. Subjects will be provided with a phone contact number and be instructed to call if anysuspected reaction to the vaccination is felt to be significant or of concern;

Subjects will be advised to notify their health care professional(s) (e.g. primary care physician) that they are participating in a clinical research study of a SARS-CoV-2 / COVID-19 vaccine;

After the 30-minute observation period (allowed window of + 15 minutes) is completed, measure vital signs (BP, HR, RR, and OT) as described in Section 10.2.1.4. Any out-of-range measurements will be assessed by the Investigator (or his/her designee) and any further action will be determined upon his/her medical decision;

Instruct the subjects to perform measurements of local and systemic AEs at approximatelythe same time each day from Day 0 to Day 7 (preferably in the evening);

Advise subjects to report any COVID-19-like symptoms (refer to list in the memory aid) they may be experiencing, or if they are tested positive for COVID-19, or have contact withindividuals who are known to be infected with SARS-CoV-2 to the clinical site and follow all of the recommended actions (e.g. quarantine, re-testing) by local Public Health authorities. Also, the subjects will be asked to not visit the clinic site until after they have fully recovered (i.e. no more clinical symptoms) and completed all of the recommended actions (e.g. quarantine, re-testing) by local Public Health authorities;

Provide appointments (date and time) for the next planned visit to the clinical site (Day 3)and for the Days 1 and 8 phone contacts;

The subject will be released from the clinical site once all Day 0 post-vaccination procedures have been completed and the subject is in stable condition.

Day 1 and Day 8 (Telephone Contact)

The post-vaccination phone contacts will be performed by blinded site staff members. The following procedures will be performed during the phone contact (acceptable interval of + 1 day for Day 1, and ± 1 day for Day 8):

Ask the subjects about any difficulties in recording their data, any change in health, anyvisits to health care facilities and/or medical practitioners, and any use of concomitant medications. Record the information in the source documents;

Ask the subjects if they experienced any COVID-19-like symptoms, were tested positive for COVID-19, or had any contact with individuals who are known to have COVID-19. Subjects who develop COVID-19-like symptoms or have had contact with individuals withCOVID-19 will be asked to provide information regarding the symptoms they are experiencing and whether they sought any medical help. Subjects with a documented SARS-CoV-2 infection will be invited to an unscheduled clinical site visit (see Section 10.1.14 for detailed procedures). Also, the subjects will be asked to not visit theclinic site until after they have fully recovered (i.e. no more clinical symptoms) and completed all of the recommended actions (e.g. quarantine, re-testing) by local Public Health authorities;

For any unsolicited AEs and/or solicited local and systemic AEs of greater than Grade 2 (moderate), the Investigator should be informed within 24 hours of the time the clinical siteis made aware of the event. The Investigator may request that the subject return to the clinicfor evaluation;

Advise subjects to immediately contact the Investigator (or his/her designee) in the event ofany AE that requires medical attention. Subjects will be provided with a phone contact number and be instructed to call if any suspected reaction to the vaccination is felt to be significant or of concern;

Instruct the subjects to contact the clinical site immediately in case they experienced any COVID-19-like symptoms, were tested positive for COVID-19, or had any contact with individuals who are known to have COVID-19. Subjects will be reminded to use their memory aid to facilitate accurate reporting of their symptoms;

Remind the subjects how to measure and record any solicited local and systemic AEs. Subjects should also be reminded to record any changes in health, including changes inAEs and changes in medications;

Remind subjects of their next appointment (date and time) for the clinical visit and/or the next telephone contact. Subjects will also be reminded to record their data in their diary andmemory aid in a timely manner.

In the event that a subject cannot be reached via phone, he/she may be contacted by text message or via email (if these contacts are available). However, the phone should be the initial and preferred means of communication. At least three attempts within allowed time window should be made in order to reach the subject.

Day 3 Follow-up (Visit 3)

The post-vaccination follow-up visit procedures will be performed by blinded site staff members. The following procedures will be performed during the Day 3 (- 1 day):

Ask the subjects if they experienced any COVID-19-like symptoms, were tested positive for COVID-19, or had any contact with individuals who are known to have COVID-19. Subjects who develop COVID-19-like symptoms or have had contact with individuals withCOVID-19 will be asked to provide information regarding the symptoms they are experiencing and whether they sought any medical help. Subjects with a documented SARS-CoV-2 infection will be invited to an unscheduled clinical site visit (see Section 10.1.14 for detailed procedures). Also, the subjects will be asked to not visit theclinic site until after they have fully recovered (i.e. no more clinical symptoms) and completed all of the recommended actions (e.g. quarantine, re-testing) by local Public Health authorities;

Perform a vital signs measurement, including resting BP, HR, RR, and OT;

Perform urinalysis on all subjects;

Collect blood samples for biochemistry and haematology;

Review the diary and memory aid content with the subject to ensure appropriate completion. Corrections must be made by the subject him/herself and all corrections mustbe initialed and dated;

Review the subject's safety data and ensure all updates on concomitant medication usage and any changes in the subject's health (AEs, SAEs, or AESIs) are recorded appropriately;

Advise the subjects to immediately contact the Investigator or his/her designee, in the eventof any AE that requires a visit to an emergency room and/or hospitalization;

Instruct the subjects to contact the clinical site immediately in case they experienced any COVID-19-like symptoms, were tested positive for COVID-19, or had any contact with individuals who are known to have COVID-19. Subjects will be reminded to use their memory aid to facilitate accurate reporting of their symptoms;

Remind the subjects of their next telephone contact (date and time).

Day 21 Dose 2 Vaccination (Visit 4)

If for any reason the Day 21 visit is done before or after the planned date, then subsequent visits/procedures (Days 22, 24, 29, 42, and 201) will be adjusted accordingly.

Pre-vaccination

The pre-vaccination procedures will be performed by blinded site staff members. The following procedures will be performed during the Day 21 (± 2 days) visit prior to vaccination:

Ask the subjects if they experienced any COVID-19-like symptoms, were tested positive for COVID-19, or had any contact with individuals who are known to have COVID-19. Subjects who develop COVID-19-like symptoms or have had contact with individuals withCOVID-19 will be asked to provide information regarding the symptoms they are experiencing and whether they sought any medical help. Subjects with a documented SARS-CoV-2 infection will be invited to an unscheduled clinical site visit (see Section 10.1.14 for detailed procedures). Also, the subjects will be asked to not visit theclinic site until after they have fully recovered (i.e. no more clinical symptoms) and completed all of the recommended actions (e.g. quarantine, re-testing) by local Public Health authorities;

Perform a vital signs measurement, including resting BP, HR, RR, and OT;

Perform urinalysis on all subjects;

Perform urine (dipstick or similar) pregnancy testing for female subjects of childbearing potential at screening. No study vaccine must be administered to a childbearing potential female until a negative result is obtained and documented;

Collect blood samples for biochemistry and haematology;

Collect blood samples for immunogenicity (serology [Nab assay and ELISA] and CMI) assessments; prepare and store these samples until shipment to the analytical laboratories;

Collect and review the diary content with the subject to ensure appropriate completion. Review the memory aid with the subject as well. Corrections must be made by the subjecthim/herself and all corrections must be initialed and dated;

Review the subject's safety data and ensure all updates on concomitant medication usage and any changes in the subject's health (AEs, SAEs, or AESIs) are recorded appropriately;

Advise the subjects to immediately contact the Investigator or his/her designee, in the eventof any AE that requires a visit to an emergency room and/or hospitalization;

Assess the subject for contraindications to the second vaccination according to Section 11.3. If the subject has no contraindications to the second vaccination, proceed to register the visit in the interactive response technology (IRT) system;

Register the visit in the IRT system. The IRT system will assign the same dose assignmentat Day 21 as Day 0. If the subject has a contraindication for the second vaccination, recordthe status according to the IRT manual.

Dose 2 Vaccination

The same procedures will be performed during the administration of the vaccine on Day 21 as will be performed during the administration of the vaccine on Day 0 (see Section 10.1.4.2 for

detailed procedures), with the following exception regarding which arm to use for IM administration:

The study vaccine should be administered IM into the deltoid muscle of the alternate arm (i.e. the arm not used for the previous dose administration). Record the administration, leftor right arm, in the subject source documents.

Post-Dose 2 Vaccination

The same procedures will be performed following the administration of the vaccine on Day 21 as will be performed following the administration of the vaccine on Day 0 (see Section 10.1.4.3 for detailed procedures), with the following exceptions:

Provide appointments (date and time) for the next planned visit to the clinical site (Day 24)and for Days 22 and 29 telephone contacts;

The subject will be released from the clinical site once all post-vaccination procedures havebeen completed and the subject is in stable condition.

Day 22 and Day 29 (Telephone Contact)

The same procedures will be performed during the phone contacts on Day 22 and Day 29 as were performed during phone contacts on Day 1 and Day 8 (see Section 10.1.5 for detailed procedures).

Day 24 (Visit 5)

The same procedure will be performed during the Day 24 visit as were performed during the follow-up visit on Day 3 (see Section 10.1.6 for detail procedures).

Day 42 (Visit 6)

The same procedure will be performed during the Day 42 visit as were performed during the pre-vaccination portion of the visit on Day 21 (see Section 10.1.7.1 for detail procedures), with the following exceptions:

Collect and review the diary and memory aid content with the subject to ensure appropriate completion. Corrections must be made by the subject him/herself and all corrections must be initialed and dated. Provide subjects with just a memory aid for the collection of safety data from Day 43 to Day 201;

Do not collect blood samples for biochemistry and haematology;

Collect blood samples for immunogenicity (serology [Nab assay, ELISA, and plant-specific glycans IgE antibodies] and CMI) assessments and to test for SARS-CoV-2 antibodies; prepare and store these samples until shipment to the analytical laboratory;

Instruct the subjects to contact the clinical site immediately in case they experienced any COVID-19-like symptoms, were tested positive for COVID-19, or had any contact with

individuals who are known to have COVID-19. Subjects will be reminded to use their memory aid to facilitate accurate reporting of their symptoms;

Provide appointments (date and time) for the next telephone contacts and the next plannedvisit to the clinical site (Day 201).

Monthly Calls Thereafter (Telephone Contact)

Subjects should be contacted by telephone once every month (every 30 days ± 14 days; Day 42 visit date as starting reference or last day of contact with the clinic site or Investigator if a second vaccination did not occur). The post-vaccination monthly phone contacts will be performed by blinded site staff members. The following procedures will be performed during the phone contacts:

Ask the subjects if they experienced any COVID-19-like symptoms, were tested positive for COVID-19, or had any contact with individuals who are known to have COVID-19. Subjects who develop COVID-19-like symptoms or have had contact with individuals withCOVID-19 will be asked to provide information regarding the symptoms they are experiencing and whether they sought any medical help. Subjects with a documented SARS-CoV-2 infection will be invited to an unscheduled clinical site visit (see Section 10.1.14 for detailed procedures). Also, the subjects will be asked to not visit theclinic site until after they have fully recovered (i.e. no more clinical symptoms) and completed all of the recommended actions (e.g. quarantine, re-testing) by local Public Health authorities;

Ask the subjects about any change in health (AEs ongoing from Day 42, SAEs, or AESIs),any visits to health care facilities, and/or medical practitioners and use of any concomitant medications. Record the information in the source documents;

Advise the subjects to immediately contact the Investigator (or his/her designee), in theevent of any AE which require a visit to the emergency and/or hospitalization;

Instruct the subjects to contact the clinical site immediately in case they experienced any COVID-19-like symptoms, were tested positive for COVID-19, or had any contact with individuals who are known to have COVID-19. Subjects will be reminded to use their memory aid to facilitate accurate reporting of their symptoms;

Remind the subjects of their next appointment (date and time) for the next telephone contact and/or the next planned visit to the clinical site (Day 201);

Inform the subjects that during the subsequent visit to the clinical site, they will be questioned regarding any events that may have occurred since the last contact.

In the event that a subject cannot be reached via telephone, he/she may be contacted by text message or via email (if these contacts are available). However, the telephone should be the initial and preferred means of communication. At least three attempts within allowed time window should be made in order to reach the subject.

Day 201 (Visit 7)

The post-vaccination visit procedures will be performed by blinded site staff members. The following procedures will be performed during the Day 201 (± 14 days):

Ask the subjects if they experienced any COVID-19-like symptoms, were tested positive for COVID-19, or had any contact with individuals who are known to have COVID-19. Subjects who develop COVID-19-like symptoms or have had contact with individuals withCOVID-19 will be asked to provide information regarding the symptoms they are experiencing and whether they sought any medical help. Subjects with a documented SARS-CoV-2 infection will be invited to an unscheduled clinical site visit (see Section 10.1.14 for detailed procedures). Also, the subjects will be asked to not visit theclinic site until after they have fully recovered (i.e. no more clinical symptoms) and completed all of the recommended actions (e.g. quarantine, re-testing) by local Public Health authorities;

Review the subject's safety data and ensure all updates on concomitant medication usage and any changes in the subject's health (SAEs, AESIs, or AEs ongoing from Day 42) are recorded appropriately. Collect the subject's memory aid and provide a memory aid for thecollection of safety data from Day 202 to Day 386;

Confirm that the subject has continued to comply with protocol requirements (e.g. no use of prohibited concomitant medications, use of an adequate contraceptive method);

Perform a vital signs measurement, including resting BP, HR, RR, and OT;

Collect blood samples for immunogenicity (serology [Nab assay, ELISA, and plant-specific glycans IgE antibodies] and CMI) assessments and collect a blood sample to test for SARS-CoV-2 antibodies; prepare and store these samples until shipment to the analytical laboratories.

Final Visit – Day 386 (Visit 8)

The final visit procedures will be performed by blinded site staff members. The following procedures will be performed during the Day 386 (± 14 days):

Ask the subjects if they experienced any COVID-19-like symptoms, were tested positive for COVID-19, or had any contact with individuals who are known to have COVID-19. Subjects who developed COVID-19-like symptoms or had contact with individuals with COVID-19 will be asked to provide information regarding the symptoms they experiencedand whether they sought any medical help. Subjects with a documented SARS-CoV-2 infection will be invited to an unscheduled clinical site visit (see Section 10.1.14 for detailed procedures). If the subject is experiencing COVID-like symptoms at or near the time of the Day 386 visit, they will be asked to not visit the clinic site until after they havefully recovered (i.e. no more clinical symptoms) and completed all of the recommended actions (e.g. quarantine, re-testing) by local Public Health authorities;

Review the subject's safety data and ensure all updates on concomitant medication usageand any changes in the subject's health (SAEs, AESIs, or AEs ongoing from Day 42) arerecorded appropriately. Collect the subject's memory aid;

Confirm that the subject has continued to comply with protocol requirements (e.g. no use of prohibited concomitant medications, use of an adequate contraceptive method);

Perform a vital signs measurement, including resting BP, HR, RR, and OT;

Collect blood samples for immunogenicity (serology [Nab assay, ELISA, and plant-specific glycans IgE antibodies] and CMI) assessments and collect a blood sample to test for SARS-CoV-2 antibodies; prepare and store these samples until shipment to the analytical laboratories.

Any subject who withdraws consent from the study will be asked to undergo Day 386 visit (Visit 8) procedures within two weeks of withdrawal, if the subject agrees.

Unscheduled Clinical Site Visit

Subjects with a documented SARS-CoV-2 infection will be invited to perform an unscheduled clinical site visit after full clinical recovery and fulfillment of the local Public Health authorities' recommendations for self-isolation and management of COVID-19. The Investigator will collect information about the COVID-19 case and any required medical interventions and will retrospectively evaluate the disease severity. The following procedures will be performed by blinded site staff members during this visit:

Confirm the subject had a documented SARS-CoV-2 infection;

Confirm the subject has fully recovered from the clinical manifestation of COVID-19 andhas fulfilled the recommendations for managing their condition as per the local Public Health authorities;

The Investigator (or sub-Investigator) will collect information about the case of COVID-19,any required medical interventions, and retrospectively evaluate the disease severity;

Collect blood samples for immunogenicity (serology [Nab assay, ELISA, and plant-specific glycans IgE antibodies] and CMI) assessments, to assess humoral and cellular responses to SARS-CoV-2 infection, and to test for SARS-CoV-2 antibodies; prepare and store these samples until shipment to the analytical laboratories.

Safety

Evaluations

Safety and tolerability will be evaluated by solicited local and systemic adverse events (immediate solicited adverse events within 30 minutes post-vaccination and solicited adverse events up to seven days after each vaccination, where applicable), unsolicited AEs within 30 minutes post-vaccination and up to 21 days after each vaccination (where applicable), SAEs, AESIs, and AEs leading to withdrawal up to the end of the study. In addition, events will be monitored for possible VED, hypersensitivity components, and potential immune-mediated

diseases, from all reported events during the study (collected AEs, SAEs, AESIs, and AEs leading to withdrawal). Clinical safety methods will include repeated urine, blood chemistry, and haematology testing.

Solicited Local and Systemic Adverse Events

Subjects will be monitored for both solicited local AEs (erythema, swelling, and pain at the injection site) and solicited systemic AEs (fever, headache, fatigue, muscle aches, joint aches, chills, a feeling of general discomfort, swelling in the axilla, swelling in the neck, swelling in the groin, and swelling in the chest wall) from the time of each vaccination through seven days. While the subjects remain in the clinic following vaccine administration (at least 30 minutes), staff will monitor them for solicited local and systemic AEs; after release from the clinic facility, from the evening of each vaccination to the evening of the seventh day after each vaccination, subjects will measure and record their local and systemic AEs in their diaries.

The intensity of the solicited local and systemic AEs will be assessed by the subject, documented in the diary, and graded as: mild (1), moderate (2), severe (3) or potentially life threatening (4) (please refer to Table 5). The causal relationship with the study vaccine of the solicited local and systemic AEs will be assessed by the Investigator (definitely not related, probably not related, possibly related, probably related or definitely related) and documented in the Investigator section of the diary; see Section 13.1.8 for a definition of these causal relationships.

The Investigator should assess solicited AEs and determine if any meet the criteria for SAE. Any solicited local or systemic AEs that meet the criteria for SAE should be reported to the Sponsor within 24 hours (Section 13.1.5) and entered as an SAE in the eCRF.

Note: Fever is also a symptom of COVID-19. Subjects should be encouraged to contact the clinical site if they experience fever alone or associated with other symptoms such as chills, muscle aches, or malaise within the first three days after vaccination.

Table 5 Severity Grades for Solicited Local and Systemic Adverse Events

**Symptoms**	**Severity**				
	**None**	**Grade 1 (Mild)**	**Grade 2 (Moderate)**	**Grade 3 (Severe)**	**Grade 4 (Potentially life-threatening)**
**Injection Site Adverse Events (Local Adverse Events)**					
**Erythema (redness)**	< 25 mm	25 - 50 mm	51 - 100 mm	> 100 mm	Necrosis or exfoliative dermatitis
**Swelling**	< 25 mm	25 - 50 mm and does not interfere with activity	51 – 100 mm or interferes with activity	> 100 mm or prevents daily activity	Necrosis
**Pain**	None	Does not interfere with activity	Repeated use of non-narcotic pain reliever for more than 24 hours or interferes with activity	Any use of narcotic pain reliever or prevents daily activity	Results in a visit to emergency room (ER) or hospitalization

Symptoms	Severity				
	None	Grade 1 (Mild)	Grade 2 (Moderate)	Grade 3 (Severe)	Grade 4 (Potentially life-threatening)
**Solicited Systemic Adverse Events**					
**Fever (ºC or ºF)**	< 38.0 ºC<br>< 100.4 ºF	38.0 - 38.4 ºC<br>100.4 - 101.1 ºF	38.5 - 38.9 ºC<br>101.2 - 102.0 ºF	39.0 - 40.0 ºC<br>102.1 - 104.0 ºF	> 40.0 ºC<br>> 104.0 ºF
**Headache**	None	No interference with activity	Repeated use of non-narcotic pain reliever for more than 24 hours or some interference with activity	Significant; any use of narcotic pain reliever or prevents daily activity	Results in a visit to emergency room (ER) or hospitalization
**Fatigue**	None	No interference with activity	Some interference with activity	Significant; prevents daily activity	Results in a visit to emergency room (ER) or hospitalization
**Muscle aches**	None	No interference with activity	Some interference with activity	Significant; prevents daily activity	Results in a visit to emergency room (ER) or hospitalization
**Joint aches, chills, feeling of general discomfort or uneasiness (malaise), swelling in the axilla, swelling in the neck, swelling in the groin, swelling in the chest wall**	None	No interference with activity	Some interference with activity not requiring medical intervention	Prevents daily activity and requires medical intervention	Results in a visit to emergency room (ER) or hospitalization

Adverse Events and SARS-CoV-2 Positive Reports

All spontaneous unsolicited AEs occurring within 21 days after each vaccination will be reported in the “Adverse Event” screen in the subject’s eCRF, irrespective of intensity or whether or not they are considered to be vaccination-related. Thereafter, from the 43rd day to the end of the study, SAEs, AEs leading to withdrawal, and AESIs will be monitored and reported in the eCRF.

The intensity of unsolicited AEs will be graded as: mild (1), moderate (2), severe (3) or potentially life threatening (4), according to the FDA Guidance for Industry [FDA 2007]. Their causal relationship with the study vaccine will be assessed by the Investigator (definitely not related, probably not related, possibly related, probably related or definitely related); see Section 13.1.8 for a definition of these causal relationships.

All laboratory-confirmed reports of SARS-CoV-2 infection (with or without symptoms) will be monitored and reported in the subject’s eCRF for the entire duration the study. Symptomatic cases (i.e. COVID-19) will be reported on the “Adverse Event” screen of the subject’s eCRF. The Investigator will assess all of these events to determine if any meet the criteria for an SAE

(see Section 13.1.1) or AESI for VED (see Section 13.1.3.1.1) and follow the process for reporting SAEs or AESIs (see Section 13.1.5).

Clinical Laboratory Tests

Blood samples for biochemistry and hematology, HIV, Hepatitis B and Hepatitis C markers in serum, testing for SARS-CoV-2 antibodies, and urine samples for urinalysis will be collected according to the Time and Events Schedule (see Table 1). In addition, serum or urine samples from all females of childbearing potential for pregnancy testing will be collected. Any laboratory result outside of the testing laboratory's normal range will be classified as 'clinically significant' (CS) or 'not clinically significant' (NCS) by the site Investigator, with appropriate documentation. Any laboratory test performed at Screening with a value outside the normal range may be repeated (prior to vaccination) at the discretion of the Investigator. Repeated tests will be considered the Baseline results.

The Investigator must review the laboratory reports, document this review, and record any clinically relevant changes occurring during the study in the source documents. The tests to be performed by the laboratory are presented in Table 6.

Table 6 Clinical Laboratory Tests

**Biochemistry (serum):**	
Sodium	Alkaline phosphatase
Potassium	Alaninetransferase (ALT)
Urea	Aspartatetransferase (AST)
Creatinine	Gamma glutamyltransferase (GGT)
Glucose	Cholesterol (total, HDL, LDL)
Bilirubin (total)	Triglyceride
Albumin	Chloride
Total protein	Calcium
	Phosphorus
**Haematology:**	
Haemoglobin	Mean cell haemoglobin (MCH)
Hematocrit	Mean cell concentration (MCHC)
Red blood cells	Mean cell volume (MCV)
Platelets	Lymphocytes
Mean platelet volume (MPV)	Monocytes
White cell count (total, WBC)	Eosinophils
Neutrophils	Basophils

Serology:	
HIV<br>Hepatitis C	Hepatitis B
**Urinalysis:**	
Macroscopic examination (color, aspect)<br>pH<br>Specific gravity	Glucose<br>Protein<br>Blood

Other clinical laboratory tests include a test to measure SARS-CoV-2 antibodies at screening and on Days 0 (prior to vaccination), 42, 201, and 386 for all subjects, and a serum pregnancy test at screening and a urine (dipstick or similar) pregnancy test prior to randomization on Day 0 and on Days 21 and 42 for all female subjects of childbearing potential.

All protocol required safety laboratory parameters are defined in study-specific documentation.

Vital Signs

Vital signs measurements (resting BP, HR, RR, and OT) will be performed as part of screening procedures, prior to vaccination (on Day 0 and Day 21), and after the post-vaccination 30-minute surveillance period (and repeated if deemed necessary by the Investigator). In addition, vital signs measurements will be performed at the clinic site visits on Day 3, Day 24, Day 42, Day 201, and Day 386.

OT will be collected in degrees Celsius or Fahrenheit using a digital thermometer by the Sponsor. The OT measurement should not be collected immediately following consumption of a hot or cold beverage or after smoking.

BP will be taken after the subject has been in a seated position as per the site’s standard procedure. BP should be taken by cuff (manual or automated are both acceptable). BP, HR and RR may be repeated once if judged necessary. All measurements (including any repeats) will berecorded in the source documents.

Physical Examinations

A history- or symptom-directed physical examination will be performed by the Investigator (or sub-Investigator) as part of screening procedures.

Pregnancy

Female subjects who become pregnant during the study will be followed for safety. The Investigator, or his/her designee, will collect pregnancy information on any subject who becomes pregnant while participating in this study. The Investigator or his/her designee will record pregnancy information on the Pregnancy Report Form (for the template of the form, refer to study documentation) and submit it to the Sponsor Safety Contact (see Section 13.1.5) within 24 hours of learning of a subject's pregnancy. The subject will be followed to determine the outcome of the pregnancy. At the end of the pregnancy, whether full-term or premature, information on the status of the mother and child will be forwarded to the Sponsor Safety

Contact, if available. Generally, follow-up will be no longer than eight weeks following the estimated delivery date.

While pregnancy itself and elective termination of a pregnancy for non-medical reasons are not considered to be an AE/SAE, any pregnancy complication or elective termination of a pregnancy for medical reasons will be recorded in the Pregnancy Report Form or as an SAE and will be followed. A spontaneous abortion is always considered to be an SAE and will be reported as such. Furthermore, any SAE occurring as a result of a post-study pregnancy and considered reasonably related in time to receipt of the investigational product by the Investigator, will be reported to the Sponsor Safety Contact. While the Investigator is not obligated to actively seek this information from former study subjects, he/she may learn of a pregnancy through spontaneous reporting. Information on pregnancies identified during the screening phase/prior to vaccine administration do not need to be collected; this information need not be communicated to Medicago (or its designee).

Safety Endpoints

Primary Endpoints

Percentage, intensity, and relationship to vaccination of immediate AEs (30 minutes aftereach vaccination);

Percentage, intensity, and relationship to vaccination of solicited local and systemic adverseAEs (for seven days following each vaccine administration);

Percentage, intensity, and relationship of unsolicited AEs for 21 days following each vaccine administration;

Occurrences of SAEs, AEs leading to withdrawal, AESIs (including VED), and deaths for21 days following each vaccine administration;

Number and percentage of subjects with normal and abnormal clinically significant urine, haematological and biochemical values prior to and 3 days following each vaccination.

Secondary Endpoints

Occurrences of SAEs, AEs leading to withdrawal, AESIs (including VED), and deathsfrom 22 days after the last vaccination up to Day 201;

Occurrences of SAEs, AEs leading to withdrawal, AESIs (including VED), and deathsfrom Day 202 up to the end of the study (Day 386).

Exploratory Endpoints

If deemed necessary, further characterization of the safety profile of the Coronavirus-Like Particle COVID-19 Vaccine;

Occurrence(s) of laboratory-confirmed (virologic or serologic methods) SARS-CoV-2 infection (with or without symptoms) up to the end of the study.

Immunogenicity

Immunogenicity Evaluations

Immunogenicity will be evaluated by the humoral immune response (Nab assay and IgG and/or IgM ELISA) and the CMI response induced in subjects on Days 0, 21, 42, 201, and 386 as well as the IgE antibody response directed against plant glycans on Days 0, 42, 201, and 386.

The blood samples for immunogenicity will be analyzed in one or more central laboratories; information on processing and the central laboratories will be provided in the study-specific documentation.

Immunogenicity Endpoints

Point estimates and 95 % CI will be calculated for the primary and secondary immunogenicity endpoints and responses for the treatment groups will be compared using descriptive and inferential statistics.

Primary Endpoints

Neutralizing antibody (Nab assay) response induced by the vaccine against the SARS-CoV-2 virus 21 days after each vaccination will be analyzed using the following parameters: GMT, SC rate (4-fold rise in Nab titer), and GMFR);

Specific Th1 CMI response induced by the vaccine against the SARS-CoV-2 virus 21 daysafter each vaccination, as measured by IFN-γ ELISpot;

Specific Th2 CMI response induced by the vaccine against the SARS-CoV-2 virus 21 daysafter each vaccination, as measured by IL-4 ELISpot.

Secondary Endpoints

Specific antibody response induced by the vaccine against the SARS-CoV-2 virus 21 daysafter each vaccination and on Day 201 and Day 386, as measured by total IgG and/or IgMlevels and will be analyzed using the following parameters: GMT and GMFR;

Neutralizing antibody (Nab assay) response induced by the treatment groups against the SARS-CoV-2 virus on Day 201 and Day 386 will be analyzed using the following parameters: GMT, SC rate (4-fold rise in Nab titer), and GMFR;

Specific Th1 CMI response induced by the vaccine against the SARS-CoV-2 virus onDay 201 and Day 386, as measured by IFN-γ ELISpot;

Specific Th2 CMI response induced by the vaccine against the SARS-CoV-2 virus onDay 201 and Day 386, as measured by IL-4 ELISpot.

Exploratory Endpoints

Specific CMI response induced by the vaccine against the SARS-CoV-2 virus 21 days aftereach vaccination and on Day 201 and Day 386, as measured by the percentage of CD4+ T cells expressing functional markers;

Specific antibody response induced by the vaccine against plant glycans 21 days after the second vaccination and on Day 201 and Day 386, as measured by serum IgE levels directedagainst CCD MUXF3 using bromelain glycoprotein;

If deemed necessary, further characterization of the immune response of the Coronavirus-Like Particle COVID-19 Vaccine.

SUBJECT COMPLETION/WITHDRAWAL

Temporary Contraindications

An exclusion criterion that renders subjects ineligible for the study may be temporary in nature:

Acute disease defined as presence of any moderate or severe acute illness with or without afever within 48 hours prior to vaccination.

If, on the day of randomization, a subject is considered ineligible due to this "temporary contraindication", the subject should be considered as a screening failure. Following the resolution of such conditions, a subject may be rescreened (including the informed consent process) under a new number and, if considered eligible by the Investigator, be enrolled in the study.

Screening Failures

Screening failures are subjects who have signed the study-specific ICF but are not eligible for enrollment (subjects who were not randomized), due to failure on one or more of the inclusion or exclusion criteria or because the subject withdrew consent prior to randomization.

Recording of screening failures documented in the study records maintained at the participating clinical sites and recorded in the IRT system. Screen failures will not receive a safety follow-up. Any subjects who are considered as a screening failure should be indicated as such. A screening failure subject can be rescreened (under a new number). If the subject is rescreened (including the informed consent process), a new subject number will be allocated.

Contraindications for Subsequent Vaccination

The following events constitute absolute contraindication to the further administration of the study treatments at Day 21; if any of these events occur during the study, the subject will not receive an additional dose of vaccine but will continue with the monthly phone contacts (see Section 10.1.11) and the Day 201 scheduled visit (see Section 10.1.12) and the Day 386 scheduled visit (see Section 10.1.13) at the discretion of the Investigator:

Pregnancy (see Section 10.2.2);

Anaphylaxis in response to first vaccine administration;

Have SARS-CoV-2 infection, experience COVID-19-like symptoms, or have been in contact with someone known to have COVID-19. Subjects with a documented SARS-CoV-2 infection will be invited to an unscheduled clinical site visit (see Section 10.1.14 for detailed procedures);

Acute disease within 48 hours prior to vaccination (acute disease is defined as presence ofany moderate or severe acute illness with or without a fever);

Experience a possibly related, probably related, or definitely related SAE or Grade 3 orhigher AE that cannot be clearly attributed to another cause;

Discovery of any health condition which, in the Investigator's opinion, may place the subject at increased risk from receipt of further vaccination; or discovery of a change in thesubject's status which may render him/her unable to comply with protocol-mandated safetyfollow-up. If an AE occurred prior to or at the time of the Day 21 scheduled visit, the subject may be discontinued at the discretion of the Investigator or the subject may be vaccinated at a later date, however, within the time window specified in the protocol (see Section 10.1.7.1). The subject must be followed until resolution of the event as with any AE (see Section 13.1.2).

Removal of Subjects from Treatment or Assessment

Subjects will be advised that they are free to withdraw from the study at any time without prejudice to their future medical care by the physician or the institution. Subjects who withdraw or are withdrawn from the study after vaccination will not be replaced.

Every reasonable effort will be made to ensure that each subject complies with the protocol and completes all study visits. However, a subject may withdraw or be withdrawn from participationin the study if:

The subject withdraws consent;

The subject is lost to follow-up;

The subject is incarcerated or incapacitated during the conduct of the clinical study;

The subject has moved away from the study area and can no longer fulfill the terms of their participation in the clinical study;

The subject displays non-compliance to the terms of their participation in the clinical study(based on Investigator's or Medicago's [or its designee's] opinion);

The Investigator has lost confidence in the subject's ability to adhere to the terms of their participation in the clinical study (based on Investigator's opinion);

Safety reasons as judged by the Investigator and/or Medicago (or its designee);

Medicago or the regulatory authority(ies) terminates the clinical study.

Withdrawal subjects will be those who leave the study before Day 386, for whatever reason; withdrawal subjects will not be replaced. Any subject who withdraws consent from the study

will be asked to visit the clinical site within two weeks of withdrawal, if the subject agrees. The procedures performed for the final assessment will comprise of those for the Day 386 visit, if permitted by the subject. All withdrawal subjects must be reported to Medicago (and/or its designee). The reason for withdrawal should be documented in the subjects' records and in the appropriate section of the eCRF. The eCRF must be completed up to and including the time of the drop-out/final assessment.

A subject may be considered discontinued from treatment if the subject does not receive both vaccine administrations (i.e. second vaccine administration is not completed; see Section 11.3 for details). A subject discontinuation from the treatment may not necessarily be discontinued from the study as further study procedures and follow-up may be performed (safety and immunogenicity), if permitted by the subject. All discontinued from treatment subjects must be reported to Medicago (and/or its designee). The reason for discontinuation from treatment should be documented in the subjects' records and in the appropriate section of the eCRF. The eCRF must be completed up to and including the time of the drop-out/final assessment.

Follow-up of Discontinuations

All subjects who receive a study vaccine will be followed for safety until the end of the study, if permitted by the subject.

Lost to Follow-up Procedures

Every attempt will be made to contact study subjects who are lost to follow-up. At least three contacts will be attempted and recorded in the source documents. As a last resort, one registered letter requesting contact with the site will be sent to any subject with whom the clinic staff no longer has contact. All attempts at contact will be documented in the subject's source documents.

Interruption of the Study

The Investigator (in consultation with Medicago [or its designee]), Medicago, or Regulatory Authorities may interrupt or terminate this study for any reason. In addition, the IDMC may interrupt or halt the study for safety reasons (refer to Section 13.1.11). The Investigator will immediately, on discontinuance of the clinical study at the clinical site, inform both the study subjects and the Institutional Review Board (IRB) responsible for the study of the discontinuance, provide them with reasons for the discontinuance and advise them in writing of any potential health risks to the subjects themselves or to other persons. It is Medicago's (or its designee's) responsibility to report discontinuance of the study to the local regulatory agencies within the appropriate timeframe, providing them with the reasons for the discontinuance and advising them in writing of any potential health risks to the study subjects or to other persons. Medicago (or its designee) must then inform the Investigator that the appropriate notifications were done.

STATISTICAL METHODS

A general description of the statistical methods used to analyze the safety and immunogenicity data is outlined below. Complete details will be provided in the SAP, which will be finalized prior to database lock.

All descriptive and inferential statistical analyses will be performed using Statistical Analysis System® (SAS®) software (version 9.4 or higher).

Analysis Populations

Safety Analysis Set

The SAS is defined as all subjects who received either the Coronavirus-Like Particle COVID-19 Vaccine with or without an adjuvant. All safety analyses will be performed using the SAS. All subjects will have their safety analyzed after each vaccination according to the last vaccine they actually received, and after any vaccination (Day 0-Day 42 safety assessment) according to the vaccine received at the first dose.

Intention-To-Treat Set

The ITT set will consist of all subjects who were randomized in the study. Subjects who received the wrong treatment will be analyzed as randomized.

Per Protocol Set

The PP set will consist of a subset of the ITT set who completed the study with no major deviations related to subject eligibility, the ability to develop a valid immune response, prohibited medication use, or the immunogenicity analyses; and who received the Coronavirus- Like Particle COVID-19 Vaccine. For the Day 21 analysis, this should include the subjects who received the first vaccine dose and had Day 0 and Day 21 immunogenicity sample collections. For the Day 42 analysis, this should include the subjects who received both vaccine doses and had Day 0 and Day 42 immunogenicity sample collections. For the Day 201 analysis, this shouldinclude the subjects who received both vaccine doses and had Day 0 and Day 201 immunogenicity sample collections. For the Day 386 analysis, this should include the subjects who received both vaccine doses and had Day 0 and Day 386 immunogenicity sample collections. Subjects who had blood samples for immunogenicity taken outside of the time window for blood sample collection are to be excluded from the PP set for the specific visit.

Subjects who received the wrong treatment at the first vaccination, but for whom the treatment received can be unequivocally confirmed, will be analyzed as treated, provided they have no other deviations that compromise their data. Subjects who receive a treatment at the second vaccination that is not the same treatment received at the first vaccination will not be included inthe PP set at Day 42, Day 201, and Day 386; the subjects will be remain in the Day 21 analysis and analyzed as treated.

The analyses of all immunogenicity endpoints will be performed using the PP set, as the primary analysis population, and the ITT set, as a secondary analysis population.

Sample Size Determination

The sample size of approximately 180 subjects with 20 subjects in each treatment group will make it possible to perform the initial evaluation of the vaccine immunogenicity and detect gross differences in rates of adverse events. The sample size is not large enough to detect all types, including less frequent or rare, adverse reactions. The objective of this study is to quantify the type, percentage, intensity, duration, and relationship of short-term post-vaccination events to determine if they differ clinically among the treatment groups.

Day 21 Data Transfer, Day 42 Data Analysis, and Interim Analysis

After the last subject has completed Day 21 assessments, the available safety and immunogenicity data will be transferred to the unblinded member(s) of the third-party statistical team to utilize for the finalization of study design of the subsequent Phase 2 study. The data transfer will be confidential and strictly limited to the authorized unblinded staff members (see Section 6.2).

Data analysis will be performed after the last subject has completed Day 42 assessments and selected tables, listings, and figures (as applicable) will be generated according to the SAP, by the third-party statistical team. The results of the data analysis will be included in a Day 42 report, including group level unblinding. However, the subject level treatment assignments and randomization codes will remain confidential and strictly limited to the authorized unblinded staff members (see Section 6.2) until the final study unblinding. Moreover, these results will provide additional information to consider for the design of the subsequent Phase 2 study (i.e. most effective dose level, more effective adjuvant, dose schedule).

An interim analysis will be performed after the last subject has completed Day 201 assessments and selected tables, listings, and figures (as applicable) will be generated according to the SAP, by the third-party statistical team. The results of the interim analysis will be used to prepare the clinical study report (up to Day 201) for the purpose of reporting to regulatory agencies the safety and immune response profile of the Coronavirus-Like Particle COVID-19 Vaccine up to six months after the second vaccination. The results from the analysis of data up to Day 386 willbe presented in an addendum to the clinical study report.

Safety Analyses

The safety endpoints are defined in Section 10.2.3.

The original terms used in the eCRFs by Investigators to identify AEs will be coded using the Medical Dictionary for Regulatory Activities (MedDRA®). All eCRF reported AEs with onset post-vaccination will be included in the safety analyses.

Special attention will be given to those subjects who die, who discontinue from the study due to an AE, who experience an SAE (e.g. summaries, listings, and narrative preparation may be provided, as appropriate), or who experience an AESI (see Section 13.1.3).

Analysis of Primary Endpoints

The primary safety endpoints are defined in Section 10.2.3.1.

The immediate AEs (30 minutes after each vaccination) and solicited local and systemic AEs reported up to seven days after each vaccination will be summarized by treatment using descriptive statistics. In addition, all unsolicited AEs, SAEs, AEs leading to subject withdrawal, AESIs, and deaths reported up to 21 days after each vaccination will be summarized by treatment using descriptive statistics.

Laboratory (biochemical, haematological, and urine) data will be summarized by type of laboratory test. Reference ranges and markedly abnormal results (specified in the SAP) will be used in the summary of laboratory data. Descriptive statistics will be calculated for each laboratory test at Baseline and at each scheduled timepoint (Day 3, Day 21, and Day 24). Changes from Baseline results will be presented in pre- versus post-treatment cross tabulations (with classes for below, within, and above normal ranges).

Analysis of Secondary Endpoints

The secondary safety endpoints are defined in Section 10.2.3.2.

The SAEs, AEs leading to subject withdrawal, AESIs, and deaths reported from Day 43 to theend of the study will be summarized by treatment using descriptive statistics.

For the following categories, AE information will also be presented by gender (male and female) and race (Caucasian or White; Black or African American; Asian):

Immediate solicited and unsolicited AEs up to 30 minutes after each vaccination;

Solicited local and systemic AEs within first seven days after each vaccination;

Most frequent unsolicited AEs within 21 days after vaccination;

AESIs;

SAEs;

AEs leading to death;

AEs leading to withdrawal.

Analysis of Exploratory Endpoints

The exploratory safety endpoints are defined in Section 10.2.3.3.

The occurrence(s) of laboratory-confirmed SARS-CoV-2 infection (with or without symptoms) reported up to the end of the study will be summarized by treatment using descriptive statistics.

In addition, if deemed necessary, the safety profile of the Coronavirus-Like Particle COVID-19 Vaccine will be further characterized.

Immunogenicity Analyses

Analysis of Primary Endpoints

The primary immunogenicity endpoints are defined in Section 10.3.2.1. The

following analyses for the NAb assay will be performed on the PP set:

GMT (Day 0, Day 21, and Day 42): The point estimates and the corresponding two-sided95 % CI by treatment group will be calculated as the antilog of the mean and 95 % CI of log transformed titer values;

SC rate (Day 0, Day 21, and Day 42): The point estimates and the corresponding two-sided95 % CI for subjects achieving SC by treatment group will be calculated and reported;

GMFR: the geometric mean of the ratio of GMTs (Day 21/Day 0 and Day 42/Day 0).

The GMFR will be derived by using ANCOVA to model the difference in the log of the titer values between Day 21 and Day 0 and between Day 42 and Day 0, with treatment group as main effect and baseline titer as covariate.

GMT will be compared between treatment groups using the analysis of variance (ANOVA) model. GMFR will be compared using the ANCOVA model. For SC rate, Fisher's exact tests will be used to compare between the treatment groups.

The specific Th1 CMI response induced on Day 0, Day 21, and Day 42 will be measured by the number of T cells expressing IFN-γ, using ELISpot, for each treatment group. The specific Th2 CMI response induced on Day 0, Day 21, and Day 42 will be measured by the number of T cells expressing IL-4, using ELISpot, for each treatment group. The response will be compared between treatment groups and timepoints using appropriate non-parametric (Wilcoxon) models. Additional details will be provided in the SAP.

Analysis of Secondary Endpoints

The secondary immunogenicity endpoints are defined in Section 10.3.2.2.

The following analyses for the NAb assay will be performed on the immunogenicity PP set:

GMT (Day 201 and Day 386): The point estimates and the corresponding two-sided 95 %CI by treatment group will be calculated as the antilog of the mean and 95 % CI of log transformed titer values;

SC rate (Day 201 and Day 386): The point estimates and the corresponding two-sided 95 %CI for subjects achieving SC by treatment group will be calculated and reported;

GMFR: the geometric mean of the ratio of GMTs (Day 201/Day 0 and Day 386/Day 0).

For the analyses of the total IgG and/or IgM antibody response, the GMT will be calculated on Day 0, Day 21, Day 42, Day 201, and Day 386 as well as the GMFR (Day 21/Day 0, Day 42/Day 0, Day 201/Day 0, and Day 386/Day 0).

The GMFR will be derived by using ANCOVA to model the difference in the log of the titer values between Day 21 and Day 0, between Day 42 and Day 0, between Day 201 and Day 0, and between Day 386 and Day 0, with treatment group as main effect and baseline titer as covariate.

GMT will be compared between treatment groups using the analysis of variance (ANOVA) model. GMFR will be compared using the ANCOVA model. For SC rate, Fisher's exact tests will be used to compare between the treatment groups.

The specific Th1 CMI response induced on Day 201 will be measured by the number of T cells expressing IFN-γ, using ELISpot, for each treatment group. The specific Th2 CMI response induced on Day 201 and Day 386 will be measured by the number of T cells expressing IL-4, using ELISpot, for each treatment group. The response will be compared between treatment groups and timepoints using appropriate non-parametric (Wilcoxon) models. Additional details will be provided in the SAP.

Analysis of Exploratory Endpoints

The exploratory immunogenicity endpoints are defined in Section 10.3.2.3.

The CMI response induced on Day 0, Day 21, Day 42, Day 201, and Day 386 will be measured by the percentage of T cells expressing functional markers, using flow cytometry, for each treatment group. The responses will be compared between treatment groups and timepoints usingappropriate non-parametric (Wilcoxon) models. Additional details will be provided in the SAP.

The specific antibody response against plant glycans induced on Day 0, Day 42, Day 201, and Day 386 will be analyzed by evaluating the percentage of subjects with detectable IgE levels at each timepoint for each treatment group and the percentage of subjects with IgE levels, relative to baseline (Day 0), that are greater than the limit of detection at Day 42, Day 201, and Day 386for each treatment group. Additional details will be provided in the SAP.

Baseline and Subject Disposition

Demographic data will be presented in listings and summarized by treatment. Continuous variables including age, weight, height, and BMI will be summarized with mean, median, standard deviation, minimum, and maximum. Count and percentage of subjects will be presented for categorical variables such as sex at birth, race, ethnicity, and baseline SARS-CoV-2 testing results.

The number of subjects in different study disposition statuses will be summarized by number and, when applicable, percentage (based on vaccinated subjects). Subjects' completion/discontinuation status will be listed (including subject identifier, date of completion/early discontinuation and, for those who discontinued early, the specific reason(s) fordiscontinuation).

ADVERSE EVENT REPORTING

Timely, accurate, and complete reporting and analysis of safety information from clinical studies are crucial for the protection of subjects, Investigators, and the Sponsor, and are mandated by regulatory agencies worldwide.

Definitions

Serious Adverse Events

An SAE is any untoward medical occurrence (whether or not considered to be related to the study vaccine) that, at any dose:

Results in death;

Is life-threatening (at the time of the event);

Note: the term "life-threatening" in the definition of an SAE refers to an event that put the subject at risk of death at the time of the event; it does not refer to an event that hypotheticallymight have caused death if it were more severe;

Requires inpatient hospitalization (≥ 24 hours) or prolongation of existing hospitalization (elective hospitalizations/procedures for pre-existing conditions that have not worsened are excluded);

Results in persistent or significant disability/incapacity;

Is a congenital abnormality/birth defect;

Is another medically important event.

Medical and scientific judgment should be exercised in deciding whether expedited reporting is appropriate in other situations, such as important medical events that may not be immediately life-threatening or result in death or hospitalization but that may jeopardize the subject's health or may require an intervention to prevent one of the other outcomes listed in the definition above. These events should be considered serious. Examples of such events are intensive treatment in an emergency room for allergic bronchospasm, blood dyscrasias, or convulsions thatdo not result in hospitalization or the development of drug dependency or drug abuse. See Section 13.1.5 for initial SAE reporting by the Investigator.

Adverse Events

An AE or adverse experience is defined as any untoward medical occurrence in a subject or clinical investigation subject who was administered a pharmaceutical product, with or without a causal relationship with the treatment. An AE can be any unfavorable and unintended sign, symptom, or disease temporally associated with the use of a medicinal product, whether or not related to a medicinal product.

Information such as the date and time of onset and resolution (duration), intensity (defined below), seriousness, any required treatment or action taken, outcome, relationship to the investigational vaccine, and whether the AE caused withdrawal from the study will be collected.

The intensity of all AEs will be graded as mild (1), moderate (2) severe (3), or potentially life threatening (4), according to the FDA Guidance for Industry: Toxicity Grading Scale for Healthy Adult and Adolescent Volunteers Enrolled in Preventive Vaccine Clinical Trials [FDA 2007] for the events covered in the guidance and the following definitions for all other events:

Mild (Grade 1):	The AE is easily tolerated and does not interfere with usual activity;
Moderate (Grade 2):	The AE interferes with daily activity, but the subject is still able to function;
Severe (Grade 3):	The AE is incapacitating and the subject is unable to work or complete usual activity;
Potentially life-threatening (Grade 4):	The AE is likely to be life-threatening if not treated in a timely manner.

The Investigator will be instructed to closely monitor each subject who experiences an AE (whether ascribed to the investigational product or not) until the outcome of the AE has been determined.

If any of the solicited local or systemic AEs persist beyond Day 7 after each vaccination (when applicable), these will also be recorded as unsolicited AEs. In this case, the AE start will be set as eight days post-vaccination. The subject will be requested to note when the AE resolves and to report this information to the Investigator or clinic staff at the next visit at the clinical site or contact.

The clinical importance of AEs will be determined based upon the Investigator's judgment. The Investigator must ensure that any sample obtained to follow-up on an AE is properly labeled and stored. The Investigator and others responsible for care of the subjects should institute any supplementary investigations of significant AEs based on the clinical assessment of the likely causative factor. This may include seeking the opinion of a specialist in the field of the AE.

All unsolicited AEs occurring within 21 days after each vaccination must be reported in the "Adverse Event" screen in the subject's eCRF, irrespective of intensity or whether or not they are considered to be vaccination-related. Thereafter, from Day 43 through to Day 386, SAEs, AEs leading to withdrawal, and AESIs will be monitored and reported in the eCRF. Additionaldetails on eCRF entries can be found in the eCRF completion guidelines.

Adverse Events of Special Interest

Adverse Events of Special Interest for the Coronavirus-Like ParticleCOVID-19 Vaccine

Vaccine Enhanced Disease

Safety signal of VED after exposure to the Coronavirus-Like Particle COVID-19 Vaccine will be closely monitored and assessed by retrieving data for this AESI as follows: AEs within the system organ class (SOC): immune system disorders and high level group term (HLGT): lower

respiratory tract disorders (excluding obstruction and infection), cardiac disorders, signs and

symptoms not elsewhere classified (NEC), vascular disorders, heart failures NEC, arteriosclerosis, stenosis, vascular insufficiency and necrosis, cardiac arrhythmias, myocardial disorders, and vascular hemorrhagic disorders. High level term (HLT): renal failure and impairment and preferred term (PT): pericarditis, coagulopathy, deep vein thrombosis, pulmonary embolism, cerebrovascular accidents, peripheral ischemia, liver injury, Guillain-Barresyndrome, anosmia, ageusia, encephalitis, chilblains, vasculitis, erythema multiforme (based on standardized MedDRA® classification) [Law 2020, Law 2020a] that require inpatient hospitalization (≥ 24 hours) and have laboratory confirmed SARS-Cov-2 infection will be monitored for assessment of any potential case of VED. As this list can be updated during the study, any further changes to the AESI terms will be updated in the Signal Management Plan (SMP).

A sub-optimal immune response after vaccination may increase the risk of VED in subjects who receive a vaccine formulation retrospectively considered to be "sub-optimal" (i.e. subjects who received a lower dose level/regimen and/or non-adjuvanted formulation compared to the final formulation determined to be safe and effective). These subjects will be informed that they may be eligible to participate in a potential extension study of the Coronavirus-Like Particle COVID-19 Vaccine and in this study, they will be administered the final vaccine formulation.

Hypersensitivity Reactions

All reported events will also be monitored for hypersensitivity reactions after exposure to the Coronavirus-Like Particle COVID-19 Vaccine. In eight clinical studies conducted to date with the Quadrivalent VLP Influenza Vaccine (QVLP) produced using similar plant-based technology, all reported events were monitored for a possible hypersensitivity component (eventswere searched using both narrow and broad standardized MedDRA® queries). Based on these data, there was a single case of possible early anaphylactic reaction associated with use of QVLPin humans. A small number of subjects had potential hypersensitivity reactions judged to be related to vaccine administration (no more than 0.3 % of subjects in any given QVLP treatment group experienced one of these events) and the events were distributed fairly evenly among treatment groups, including the placebo and the active comparator groups. However, since severereactions are considered to be an important potential risk (based on the theoretical risk that using plants for the production of biotherapeutics may induce hypersensitivity), Medicago will require that appropriate medical treatment and supervision are available to manage any possible anaphylactic reactions in this study. To collect data on these events, Medicago will closely monitor and assess allergic reactions assessed by the site Investigators as related to the Investigational product as AESIs.

Adverse Events of Special Interest for the Adjuvants

Potential Immune-Mediated Diseases and Other AESI as Listed in Section 19.5

Potential immune-mediated diseases (pIMDs) are a subset of AEs that include autoimmune diseases and other inflammatory and/or neurologic disorders of interest which may or may not

have an autoimmune aetiology. Adverse events that need to be recorded and reported as pIMDs include those listed in Section 19.5.

However, the Investigator will exercise their medical and scientific judgement in deciding whether other diseases have an autoimmune origin (that is pathophysiology involving systemic or organ-specific pathogenic autoantibodies) and should also be recorded as a pIMD.

When there is enough evidence to make any of the diagnoses mentioned in Section 19.5, the AE must be reported as a pIMD. Symptoms, signs or conditions which might (or might not) represent the above diagnoses, should be recorded and reported as AEs but not as pIMDs until the final or definitive diagnosis has been determined, and alternative diagnoses have been eliminated or shown to be less likely.

In order to facilitate the documentation of pIMDs in the eCRF, a pIMD standard questionnaire and a list of preferred terms (PTs) and PT codes corresponding to the above diagnoses will be available to Investigators at study start.

Once a pIMD is diagnosed (serious or non-serious) in a study subject, the Investigator (or designate) must complete, date and sign an electronic Expedited Adverse Events Report.

Expectedness of an Adverse Drug Reaction

An “unexpected” adverse reaction is one in which the nature or severity of the event is not consistent with information in the current version of the IB. Expedited reporting to the regulatory authorities is required if an SAE occurs that is both unexpected and considered possibly, probably, or definitely related to the study vaccine.

Initial SAE, AESI, and Pregnancy Reports Reporting by the Investigator

Details on safety reporting are defined in study-specific documentation, including SAE processing, country-specific reporting requirements, regulatory unblinding, roles and responsibilities of different stake holders, and contact details of personnel responsible for safety reporting. A brief summary of salient information is presented below.

All post-vaccination SAEs, AESIs, and pregnancy reports will be reported from the time of receiving the study vaccine on Day 0 through to the end of the study (final scheduled visit). The Investigator (or designee) must report, by e-mail, all SAEs, AESIs, and pregnancy reports whether considered related to the study vaccine or not to Medicago within 24 hours of the Investigator learning of the event. The Investigator must also complete, sign, and date the paper SAE report form, and send, via e-mail, a copy to the Medicago safety e-mail address and the appropriate regional e-mail address within 24 hours of awareness of event:

Medicago Safety e-mail address: Medicago-ClinicalSafety@medicago.com

Sponsor Safety Contact: Dr. Asif Mahmood, M.D., Chief Safety Officer
e-mail: mahmooda@medicago.comTel.: 1.418.658.9393 ext.433

Serious AEs will be reported to the local (or contract) IRB by the Investigator according to the IRB's policy and procedures.

Follow-up Reporting by the Investigator

All SAEs, regardless of causality, will be followed as described in Section 13.1.2. When appropriate, documentation of any medical tests or examinations performed will be provided to document resolution/stabilization of the event.

Reporting of SAEs Occurring after Study Termination

All SAEs occurring during the safety follow-up period will be followed until resolution or for a period of 30 days from the final subject's visit (which ever occurs first), regardless of conclusion of the study. However, all related SAEs occurring during the safety follow-up period will be followed until resolution or stabilization.

A post-study AE/SAE is defined as any event that occurs outside of the AE/SAE detection period (post Day 386). Active follow-up for AEs or SAEs will continue until Day 386 for all subjects. However, after Day 386, if the Investigator learns of any SAE, including death, at anytime after a subject has been discharged from the study, and he/she considers the event to be reasonably related to the investigational product, the Investigator will promptly notify the Sponsor Safety Contacts for Reporting SAEs. These related SAEs will be followed until resolution or stabilization.

Causal Relationship

The Investigator must make the determination of relationship to the study vaccine for each AE. The Investigator should decide whether, in his/her medical judgment, there is a reasonable possibility that the event may have been caused by the investigational vaccine. If there is any valid reason, even if undetermined or untested, for suspecting a possible cause-and-effect relationship between the investigational vaccine and the occurrence of the AE, then the AE should be considered "definitely related", "probably related", or "possibly related". Otherwise, if no valid reason exists for suggesting a possible relationship, then the AE should be classified as "probably not related" or "definitely not related". The following guidance should be followed:

Definitely Not Related: The AE is clearly not related to the administration of the study vaccine. Another cause of the event is most plausible; and/or a clinically plausible temporal sequence is inconsistent with the onsetof the event and the study vaccine administration; and/or a causal relationship is considered biologically implausible.

Probably Not Related: There is no medical evidence to suggest that the AE is related to the study vaccine. The event can be readily explained by the subject's

underlying medical condition or concomitant therapy or lacks aplausible temporal relationship to the study vaccine.

Possibly Related: A direct cause and effect relationship between the study vaccine and the AE has not been demonstrated but there is a reasonable possibility that the event was caused by the study vaccine.

Probably Related: There probably is a direct cause and effect relationship between the AE and the study vaccine. A plausible biologic mechanism and temporal relationship exist and there is no more likely explanation.

Definitely Related: There is a direct cause and effect relationship between the AE and the study vaccine.

AE outcomes will be classified as recovered/resolved, not recovered/not resolved, recovered/resolved with sequelae, recovering/resolving, or death.

Reporting of SAEs to Health Authorities and IRB

Medicago or its designee will be responsible for reporting SAEs that are deemed both possibly related to the study vaccine and considered to be unexpected ('unexpected' refers to events that do not appear in the package labeling or in the study vaccine IB) to the regulatory authorities in an expedited manner.

The Investigator will be responsible for reporting the SAEs that meet IRB reporting requirements directly to the relevant ethical review board as soon as possible, and will also provide the ethical review board with any safety reports prepared by Medicago or its designee.

All SAEs that are suspected, unexpected serious adverse reactions (SUSARs) should be reported to regulatory authorities by phone or by facsimile transmission as soon as possible but, in no event, later than seven calendar days for deaths and life-threatening events, and 15 calendar days for other SAEs after Medicago's (or its designee's) initial receipt of the information. These events should be followed to resolution, stabilization, or return to baseline, regardless of conclusion of the study.

Independent Data Monitoring Committee

The IDMC will consist of a multidisciplinary group of three clinicians and/or physicians and one biostatistician who will, collectively, have extensive experience in the use of vaccines for patient treatment and in the conduct and safety monitoring of randomized clinical trials.

The IDMC will monitor the study conduct and review safety data at pre-defined times during the study for the purpose of providing recommendations to the Sponsor about whether:

subject vaccination at a dose level will continue;

subject vaccination at the next higher dose level will proceed;

the study will continue in the event of a pre-defined safety signal.

The IDMC review will ensure the ongoing safety of the subjects in the study and the scientific integrity of the study.

The IDMC will participate in 12 planned review meetings (two three-day safety data review meetings for each dose level in each dose regimen), as described in Section 13.1.11 and will be provided with an unblinded three-day safety data summary (refer to Section 19.4 and further defined in the IDMC charter) for each review meeting. Additional review meetings may be scheduled with the IDMC, if deemed necessary by the IDMC or the Sponsor.

Safety Review and Stopping Rules

For each dose level group, the three-day safety data after vaccination of the first 10 % of subjects enrolled (6 subjects) in the group will be reviewed by the IDMC, prior to permitting vaccination of the next 30 % of subjects enrolled (18 subjects) in the dose level group and escalating to vaccination of subjects at the next higher dose level (until reaching the highest dose level). The three-day safety data after vaccination of these 18 subjects in the group will also be reviewed by the IDMC, prior to permitting vaccination of the remaining subjects in the dose level group. The two planned three-day safety data reviews by the IDMC for each dose level will occur after each vaccination in the study. The reviews of three-day safety data by IDMC ensure ongoing safety of study subjects as well as maintaining study scientific integrity. These reviews will detect any early negative trends in the safety data from a subset of subjects vaccinated at each dose level and may necessitate a decision to not administer the study vaccine to the remaining study subjects. A phone call reminding study subjects to complete the diary will be made at Day 1 post-vaccination and the subjects will be asked to visit the clinical site on Day 3 for safety assessments. Early accumulated safety outcome data which will include all self-reported solicited local and systemic AEs, any unsolicited adverse and/or serious adverse events occurring following administration of each injection.

The IDMC will be provided the following unblinded three-day solicited local and systemic AEs for each subset of subjects enrolled at each dose level, after each vaccination:

Occurrences of erythema, swelling and pain at the injection site;

Occurrences of fever, headache, muscle aches, joint aches, fatigue, chills, and feelings of general discomfort or uneasiness;

Swelling in the axilla, groin, neck or chest wall;

Occurrence of any adverse or serious adverse events;

Concomitant medications, doctor or emergency room or hospital visits associated with an adverse event or serious adverse event.

The three-day safety data summary (refer to Section 19.4) at each dose level will be reviewed by IDMC before allowing vaccination at the next higher dose level. If the data shows the safety of the vaccine (see Stopping Rules below), the remaining subjects will receive their first vaccination and the site could proceed with vaccination of subjects at the next higher dose level. If the data does not show safety of the vaccine (see Stopping Rules below) at a dose level and thedecision from the IDMC review is to stop further use of this dose level, then the following will apply:

Subjects administered this dose level of the vaccine will be followed to the end of the studyfor all safety and immunogenicity outcomes, if subject permits;

Subjects originally planned to be administered this dose level will not be redistributed to the other dose level groups (i.e. the planned sample size for each dose level group will notchange);

The study will continue in accordance with the protocol for the dose levels deemed safe bythe IDMC.

Stopping Rules

Safety monitoring of safety signals will be performed throughout the study. Stopping rules or conditions for stopping this clinical trial would occur if there was clear evidence of harm or harmful effects such as SAEs related to the treatment (study vaccine). A SAE which was thought to be unrelated to the study vaccine would not warrant stopping the trial.

The following event(s) may result in a halt to the study, for further review and assessment of the event(s):

Any death occurring during the study;

Any vaccine-related SAE during the study;

Any life-threatening (Grade 4) vaccine-related AE during the study;

If 10 % or more of subjects in a single treatment group, experience the same orsimilar listed event(s) that cannot be clearly attributed to another cause:

a severe (Grade 3 or higher) vaccine-related AE during the study;

a severe (Grade 3 or higher) vaccine-related vital sign(s) abnormality;

a severe (Grade 3 or higher) vaccine-related clinical laboratory abnormality.

In the case that a pre-defined safety signal is met in any treatment group, subsequent dosing will result in at least a transient halt in the study to permit a complete evaluation of the reported event(s) and to consult an IDMC. A decision as to whether the study can progress as planned must be made and documented in the event of any safety signal. If a stopping rule has been met once all subjects have been vaccinated in the study, the IDMC will be notified by a Note To File (NTF) for their information purposes.

INVESTIGATIONAL PRODUCT INFORMATION

Identity of Investigational Product

The study vaccine is a Coronavirus-Like Particle COVID-19 Vaccine composed of recombinant spike (S) glycoprotein expressed as VLPs. The Coronavirus-Like Particle COVID-19 Vaccine will be administered alone or with the adjuvants CpG 1018 or AS03.

For more information regarding the Coronavirus-Like Particle COVID-19 Vaccine, refer to the current IB.

Study Vaccine Composition

The Coronavirus-Like Particle COVID-19 Vaccine is a sterile transparent slightly yellowish liquid suspension. The Coronavirus-Like Particle COVID-19 Vaccine will be supplied in 0.30 mL single dose vials. The S glycoprotein concentration in the vaccine will be 60 µg/mL, 30 µg/mL, and 15 µg/mL for final dosages of 15 µg, 7.5 µg, or 3.75 µg, respectively.

CpG 1018 is an established effective adjuvant licensed for Heplisav-B® (in the US). The adjuvant is a clear, colorless liquid and will be provided in the original sterile vial containers received from the manufacturer (Dynavax).

AS03 is an established effective adjuvant licensed for Arepanrix™ H1N1 and Arepanrix™ H5N1 (in Canada). The adjuvant is a whitish to yellowish homogeneous milky liquid emulsion and it will be provided in the original sterile vial container received from the manufacturer (GlaxoSmithKline). A 0.25 mL dose of AS03 represents one human dose.

Preparation and Administration of Study Vaccine

The study treatments (including the Coronavirus-Like Particle COVID-19 Vaccine and the adjuvants) will be prepared by unblinded staff members at the clinical site as described in the Investigational Product Management Manual. The prepared study treatment will subsequently be administered to subjects by an unblinded staff member.

Note: The personnel responsible for treatment preparation can also perform the vaccination (if certified and qualified to do it) as this person would already be unblinded. The unblinded staff members must not be involved in the evaluation of any AEs or reactogenicity evaluations following vaccination.

The products to be used for study treatment administration will be handled in a clean environment in an aseptic manner.

Coronavirus-Like Particle COVID-19 Vaccine will be administered on study Day 0 and Day 21 as an IM injection of 0.25 to 0.75 mL, depending on the presence or absence of adjuvant, into the deltoid muscle. A 23 gauge needle of at least one inch or 2.5 cm in length can be used for vaccination.

The product administered will be recorded in study-specific documentation (refer to Investigational Product Management Manual) by the clinical site, which will be separate from the study medication record for drug preparation. After drug accountability monitoring and reconciliation has been completed by the site and unblinded monitor, all study treatments (used and unused vials) will be destroyed locally upon Medicago’s approval or returned to Medicago (or its designee) in accordance with instructions provided in the Investigational Product Management Manual.

Further specific information relating to treatment preparation, storage, and shipment is provided in the Investigational Product Management Manual.

Preparation, Handling, Storage, and Precautions for Use

The study treatment (including the Coronavirus-Like Particle COVID-19 Vaccine and the adjuvants) should be stored in an access-restricted area between 2 ºC and 8 ºC and protected from light; the vaccine should, however, be at room temperature before administration (i.e. the vaccine should not be administered cold and should be taken out of the refrigerator at least five minutes prior to administration). In the event of a storage temperature deviation outside of the permitted window of 2 ºC to 8 ºC, the study treatment should be quarantined (at the required storage temperature) and Medicago (or its designee) contacted immediately.

Note: The vaccine must NEVER be frozen, since freezing destroys activity; any vaccine thathas been frozen must not be used.

Note: The vaccine or the adjuvants AS03 and CpG 1018 must NEVER be shaken or vortexed.

Note: The treatments must NOT be administered intravenously, subcutaneously, or intradermally.

The Investigational Product Management Manual provides additional details on treatment preparation, handling and storage.

Packaging

The Coronavirus-Like Particle COVID-19 Vaccine and the adjuvants will be packaged in boxes containing vials of the same product.

Labeling

The vials will have a product and study-specific label containing information that meets the applicable regulatory requirements.

The Coronavirus-Like Particle COVID-19 Vaccine and adjuvant vial labels will not be blinded. Blinding measures will be applied to maintain the observer-blindness of the blinded staff and to allow identification of the study treatment only by staff involved in the preparation/administration of the study vaccine.

Drug Accountability

The Investigator is responsible for ensuring that all study treatments received at the site are inventoried and accounted for throughout the study. The dispensing of study treatment to each subject must be documented on study-specific documentation.

The investigational products must be handled in strict accordance with the Investigational Product Management Manual and the vial label. The investigational products will be stored in a limited access area or in a locked cabinet under appropriate environmental conditions. The unblinded site staff will complete accountability for all investigational products (Coronavirus-Like Particle COVID-19 Vaccine, AS03, and CpG 1018). All unused study drugs must be available for verification by Medicago’s (or its designee’s) unblinded site monitor during on-site monitoring visits. The return of all unused study drugs to Medicago (or its designee) for

destruction will be documented on study-specific documentation. When the site is authorized by Medicago (or its designee) to destroy study drug supplies on site, this must also be documented.

The study drug should be dispensed under the supervision of the Investigator (or a qualified member of the investigational staff), who remains blinded as to which treatment is administered. The study drug will be supplied only to subjects participating in the study. Returned study drugs may not be relabeled or reassigned for use for other subjects. The Investigator agrees neither to dispense the study drug from, nor store it at, any site other than the study centers agreed upon with Medicago (or its designee).

STUDY-SPECIFIC MATERIALS

The Investigator will be provided with the following:

eCRF;

Forms and questionnaires for special assessments;

Thermometers and measurements template;

Study manuals.

ETHICAL ASPECTS

Study-Specific Design Considerations

On-going medical review will be performed by Medicago (or its designee) throughout the duration of the study; subjects will be given any new information that may affect their decision to continue participation in the study.

Blood samples will be collected from subjects for immunogenicity analysis, although not all will be analysed. Since blood sample collection is a minimally invasive procedure and the amount of blood collected will be a relatively small amount (Table 4), the benefits of obtaining this data outweigh the small level of risk associated with the collection of samples that may not require analysis.

Regulatory Ethics Compliance

Investigator Responsibilities

The Investigator is responsible for ensuring that the study is performed in accordance with the protocol, current International Conference on Harmonisation (ICH) guidelines on Good Clinical Practice (GCP), and applicable regulatory and country-specific requirements.

GCP is an international ethical and scientific quality standard for designing, conducting, recording, and reporting studies that involve the participation of human subjects. Compliance with this standard provides public assurance that the rights, safety, and well-being of study subjects are protected, consistent with the principles that originated in the Declaration of Helsinki, and that the study data are reliable.

Independent Ethics Committee or Institutional Review Board

Before the start of the study, the Investigator (or Medicago, where required) will provide the Independent Ethics Committee (IEC)/IRB with current and complete copies of the following documents (as required by local regulations):

Final protocol and, if applicable, amendments to the protocol;

Sponsor-approved ICF (and any other written materials to be provided to the subjects);

IB (or equivalent information) and amendments/addenda;

Information on compensation for study-related injuries or payments to subjects for participation in the study, if applicable;

Investigator's *curriculum vitae* or equivalent information (unless not required, as documented by the IEC/IRB);

If applicable, any Sponsor-approved subject recruiting materials;

Information regarding funding, name of the sponsor, institutional affiliations, other potential conflicts of interest, and incentives for subjects;

Any other documents that the IEC/IRB requests to fulfill its obligation.

This study will be undertaken only after the IEC/IRB has given full approval of the final protocol, amendments (if any, excluding the ones that are purely administrative in nature, with no consequences for subjects, data, or study conduct), the ICF, applicable recruiting materials, and subject compensation programs, and Medicago (or its designee) has received a copy of this approval. This approval letter must be dated and must clearly identify the IEC/IRB and the documents being approved. In addition, the Investigator must wait for written confirmation from Medicago (or its designee) that the study can be started.

During the study, the Investigator (or Medicago [or its designee], where required) will send the following documents and updates to the IEC/IRB for their review and approval, where appropriate:

Protocol amendments;

Revision(s) to ICF and any other written materials to be provided to subjects;

If applicable, new or revised subject recruiting materials approved by Medicago;

Revisions to compensation for study-related injuries or payment to subjects for participation in the study, if applicable;

New edition(s) of the IB and amendments/addenda;

Summaries of the study at intervals stipulated in guidelines of the IEC/IRB (at a minimum, annually);

Reports of AEs that are serious, unlisted/unexpected, and associated with the study drug(when applicable);

New information that may adversely affect the safety of the subjects or the conduct of thestudy;

Deviations from or changes to the protocol to eliminate immediate hazards to subjects;

Reports of deaths of subjects under the Investigator's care;

Notification if a new Investigator is responsible for the study at any of the sites;

Development Safety Update Report and Line Listings, where applicable;

Any other requirements of the IEC/IRB.

At least once a year, the IEC/IRB will be asked to review and reapprove this study. The re-approval should be documented in writing. At the end of the study, the Investigator (or Medicago [or its designee], where required) will notify the IEC/IRB of study completion.

Informed Consent

Each subject who participates in the study must first give written consent according to local requirements after the nature of the study has been fully explained, including the risks and requirements of the study. The consent form must be signed before performing any study-related activity. The consent form that is used must have been approved by regulatory authorities, Medicago, and the reviewing IEC/IRB. The informed consent should be in accordance with principles that originated in the Declaration of Helsinki, current ICH and GCP guidelines, applicable regulatory requirements, and Medicago policy. During the study, subjects will be given any new information that may affect their decision to continue their participation in the study. They will be told that their consent to participate is voluntary and that it may be withdrawn at any time, with no reason given, and without penalty or loss of benefits to which they would otherwise be entitled. Only subjects who are fully able to understand the risks, benefits, and potential AEs of the study and who provide their consent voluntarily will be enrolled.

Privacy of Personal Data

The collection and processing of personal data from subjects enrolled in this study will be limited to those data that are necessary to support the development, registration, and future marketing of the investigational product. The data must be collected and processed with adequate precautions to ensure confidentiality and compliance with applicable data privacy protection laws and regulations. Appropriate technical and organizational measures to protect the personal data against unauthorized disclosures or access, accidental or unlawful destruction, or accidental loss or alteration must be in place. Medicago (or its designee) personnel whose responsibilities require access to personal data agree to keep the identity of study subjects confidential.

ADMINISTRATIVE REQUIREMENTS

Protocol Amendments

Neither the Investigator nor Medicago will modify this protocol without a formal amendment. All protocol amendments must be issued and approved by Medicago, and signed and dated by the Investigator. Protocol amendments must not be implemented without prior IEC/IRB approval

or when the relevant competent authority has raised any grounds for non-acceptance, except when necessary in order to eliminate an immediate hazard to the subjects, in which case the amendment must be promptly submitted to the IEC/IRB and relevant competent authority. Documentation of amendment approval by the Investigator and IEC/IRB must be provided to Medicago or its designee.

Note that administrative changes may be implemented without prior IRB approval; however, the Investigator or Medicago, as applicable, must notify the IRB of any administrative changeand ensure that IRB acknowledges receipt of the administrative changes.

The Investigator is responsible for notifying the IRB of all protocol amendments and ensuring that the IRB has approved any amendments when local IRBs are used. When a central IRB is used, Medicago or its designee will inform the IRB on behalf of the sites.

During the course of the study, in situations where a departure from the protocol is unavoidable, the Investigator or other physician in attendance will contact the appropriate Medicago representative or its designee (see Contact Information pages provided separately). Except in emergency situations, this contact should be made before implementing any departure from the protocol. In all cases, contact with Medicago or its designee must be made as soon as possible to discuss the situation and to agree on an appropriate course of action. The data recorded in the eCRF and source documents will reflect any departure from the protocol, and the source documents will describe this departure and the circumstances that made a deviation necessary.

Regulatory Documentation

17.2.1 Regulatory Approval/Notification

This protocol and any amendment(s) must be submitted to the appropriate regulatory authorities in each respective country, as applicable. A study may not be initiated until all local regulatory requirements are met.

Source Documentation

At a minimum, source documentation must be available for the following information:

Subject identification, eligibility, and study identification;

ICF discussion and date of informed consent;

Dates of visits;

Results of safety and study procedures as required by the protocol;

Record of all reactions, AEs, and associated follow-ups;

Concomitant medication;

Drug receipt, dispensing, and return records;

Study drug preparation and administration information;

Date of study completion and reason for early discontinuation of study drug or withdrawalfrom the study, if applicable.

It is recommended that the author of an entry in the source documents be identifiable.

At a minimum, the type and level of detail of source data available for a study subject should be consistent with that commonly recorded at the site as a basis for standard medical care. If Investigator judgement was used in the determination of eligibility, an explanation for inclusion of the subject in the study must be provided in the source documents. Specific details required as source data for the study will be reviewed with the Investigator before the study and will be described in the monitoring guidelines (or other equivalent document).

17.3.1 Diary and Memory Aid

Subjects will be provided with diaries in which to record reactions and other safety information. Subject diaries are considered source documents; the first diary will be returned by the subject to the site on Day 21. When the subject receives a second vaccine administration, a second diary will be provided on Day 21 (after vaccination) and it will be returned by the subject to the site onDay 42. The method of return of the diaries will be defined in the study-specific documentation and all subject diaries will be kept with each subject's study chart. In the event the diary(ies) is/are lost by the subject, information collected by the coordinator or designee and information recalled by the subject will serve as the source data for this subject. The recalled information willbe captured in the source documents.

Corrections to data entered by the subject in the diary should only be performed by the subject him/herself, when possible. Clarifications can be made in the comment section of the diary by the study personnel if the subject is not present onsite to make the correction him/herself.

Subjects will be provided with memory aids to record information on unsolicited AEs, SAEs, AEs leading to withdrawal, AESIs, and reportable concomitant medications from Day 0 to Day 42, from Day 43 to Day 201, and from Day 202 to the end of the study (Day 386). These memory aids will be collected by the site and are intended to be used by the subjects to help them in reporting this information to site staff during phone contacts and clinical site visits.

Case Report Form Completion

An eCRF will be provided for each subject who is randomized and receives a dose of study drug. Screening failures will also be entered in an eCRF; data entered should include date of birth and reason for screening failure.

Data must be entered into the eCRFs in English. The Investigator must verify that all data entries in the eCRFs are accurate and correct. All eCRF entries, corrections, and alterations must be made by the Investigator or other authorized study-site personnel.

Please refer to the eCRF completion guidelines for details of data entry requirements.

Quality Control (Study Monitoring)

In accordance with applicable regulations, GCP, and Medicago (or its designee) procedures, the site will be contacted prior to the study start to review the protocol and study requirements with the clinical study staff. Medicago (or its designee) will review their responsibilities to satisfy regulatory, ethical, and company (Medicago) requirements.

The Investigator or institution, if applicable, will authorize and provide direct access to all relevant documents to allow review of the appropriate conduct of the study by Medicago's (or its designee's) monitor. The Investigator(s) must allocate their time and the time of their staff to the monitors, discuss any findings or issues, and take appropriate actions to maintain the quality and integrity of the study data and conduct.

Protocol deviations will be reviewed by Medicago (or its designee) to identify any non-compliances likely to have a significant effect on the safety and rights of a subject or the reliability and robustness of the data generated. These deviations will be included in the clinicalstudy report.

Quality Assurance

To ensure compliance with GCP and all other applicable regulatory requirements, Medicago (or its designee) may conduct quality assurance assessments and/or an audit of the site. Regulatory agencies may conduct regulatory inspections at any time during or after completion of the study.If an audit or an inspection is conducted, the Investigator and/or institution (if applicable) must agree to grant auditors or inspectors direct access to all relevant documents and to allocate their time and the time of their staff to discuss the conduct of the study and any findings or issues, and to implement corrective and/or preventative actions to address any findings or issues identified.

Record Retention

Following closure of the study, the Investigator must maintain all site study records in a safe and secure location. The records must be easily accessible when needed (e.g. for a Medicago [or its designee] audit or regulatory inspection) and must be available for review in conjunction with assessment of the facility, supporting systems, and relevant site staff.

When permitted by local laws and regulations or institutional policy, some or all of the records may be maintained in a format other than a hard copy (e.g. scanned electronic). The Investigator must ensure that all reproductions are legible and are a true and accurate copy of the original.

The Investigator must follow the time period for retaining the site records in order to comply with all applicable regulatory requirements. The minimum retention time will meet the strictest standard applicable to a particular site, as dictated by local laws and regulations, Medicago (or its designee) standard operating procedures (SOPs), and/or institutional requirements. The Investigator must contact Medicago (or its designee) prior to the disposal of any study documents at the end of the retention period.

If the responsible Investigator retires, relocates, or, for any other reason, withdraws from the responsibility of keeping the study records, custody must be transferred to another qualified person who will accept the responsibility and is approved by Medicago. Medicago (or its designee) must be notified in writing of the name and address of the new custodian. Under no circumstance shall the Investigator relocate or dispose of any study documents without having obtained prior written approval from Medicago (or its designee).

If it becomes necessary for Medicago or the appropriate regulatory authority to review any documentation relating to this study, the Investigator (or new custodian) must permit access to such reports.

Study Completion/Termination

Study Completion

The study will be considered to be completed with the last contact with the last subject participating in the study. The final data from the investigational site will be sent to Medicago (or designee) after completion of the final subject visit at that site. Investigational sites will be closed after study completion. An investigational site is considered closed when all required documents and study supplies have been collected, all data have been entered, monitored, locked, and signed and a site closure visit has been performed.

Study Termination

Medicago (or its designee) reserves the right to close the investigational site or terminate the study at any time for any reason at the sole discretion of Medicago. Investigational sites will be closed after study termination once Medicago (or its designee) has completed all study related tasks. Reasons for the early closure of an investigational site by Medicago (or its designee) or the Investigator may include, but are not limited to:

Failure of the Investigator to comply with the protocol, Medicago’s (or its designee’s) procedures, or GCP guidelines;

Inadequate recruitment of subjects by the Investigator;

Discontinuation of further drug development.

The Investigator may initiate site closure at any time, provided there is reasonable cause andsufficient notice is given in advance of the intended closure.

Site closure following an Investigator request can only be performed when all active study subjects have completed the study period, Medicago (or its designee) has collected all required documents and study supplies, and all data have been entered, monitored and locked.

Registration of Clinical Studies and Disclosure of Results

Study information from this study will be posted on publicly available clinical trial registries in countries where applicable and will include information required by law. In addition, the results

summary will be posted to the same clinical trial registries, to the extent specified by law, and will include information required by regulatory authorities.

Publication Policy

The data derived from this study are the property of Medicago and cannot be published without prior authorization from Medicago. Any publication activities (i.e. preparation and submission of abstracts and manuscripts) will be at the discretion of Medicago.

Any proposed publication regarding this study, not prepared by Medicago personnel, must be provided to Medicago for comments and review at least 45 days prior to its intended publication. The proposed publication shall not include any confidential information or protected information to preserve Intellectual Property rights; any such information must be removed from the proposed publication.

REFERENCES

Ahn DG, Shin HJ, Kim MH, Lee S, Kim HS, Myoung J, Kim BT, Kim SJ. Current status of epidemiology, diagnosis, therapeutics, and vaccines for novel coronavirus disease 2019 (COVID-19). J Microbiol Biotechnol. 2020 Mar 28;30(3):313-324. doi: 10.4014/jmb.2003.03011.

Baz M, Luke CJ, Cheng X, Jin H, Subbarao K. H5N1 vaccines in humans. Virus Res 2013;178:78-98. doi: 10.1016/j.virusres.2013.05.006.

Channappanavar R, Zhao J, Perlman S. T cell-mediated immune response to respiratory coronaviruses. Immunol Res. 2014 Aug;59(1-3):118-28. doi: 10.1007/s12026-014-8534-z.

Chen N, Zhou M, Dong X, Qu J, Gong F, Han Y, Qiu Y, Wang J, Liu Y, Wei Y, Xia J, Yu T, Zhang X, Zhang L. Epidemiological and clinical characteristics of 99 cases of 2019 novel coronavirus pneumonia in Wuhan, China: a descriptive study. Lancet. 2020 Feb 15;395(10223):507-513. doi: 10.1016/S0140-6736(20)30211-7.

Chen Y, Liu Q, Guo D. Emerging coronaviruses: Genome structure, replication, and pathogenesis. J Med Virol. 2020a Apr;92(4):418-423. doi: 10.1002/jmv.25681.

Cheng VC, Lau SK, Woo PC, Yuen KY. Severe acute respiratory syndrome coronavirus as an agent of emerging and reemerging infection. Clin Microbiol Rev. 2007 Oct;20(4):660-94.

Cohet C, Montplaisir J, Petit D, Quinn MJ, Ouakki M, Deceuninck G, et al. Applying the test-negative design to vaccine safety studies: risk of narcolepsy associated with A/H1N1 (2009) pandemic influenza vaccination in Quebec [abstract 713]. Pharmacoepidemiol Drug Saf 2015;24:406-407. doi: 10.1002/pds.

Cohet C, van der Most R, Bauchau V, Bekkat-Berkani R, Doherty TM, Schuind A, Tavares Da Silva F, Rappuoli R, Carcon N, Innis BL. Safety of AS03-adjuvanted influenza vaccines:A review of the evidence. Vaccine 2019;37(23):3006-3021. doi: 10.1016/j.vaccine.2019.04.048.

COVID-19: Medicago’s development programs. Available online: https://www.medicago.com/en/covid-19-programs (accessed on April 23, 2020).

Du L, He Y, Zhou Y, Liu S, Zheng BJ, Jiang S. The spike protein of SARS-CoV - a target for vaccine and therapeutic development. Nat Rev Microbiol. 2009 Mar;7(3):226-36. doi: 10.1038/nrmicro2090.

Enjuanes L, Zuñiga S, Castaño-Rodriguez C, Gutierrez-Alvarez J, Canton J, Sola I. Molecular basis of coronavirus virulence and vaccine development. Adv Virus Res. 2016;96:245-286. doi: 10.1016/bs.aivir.2016.08.003.
FDA. Guidance for Industry: Toxicity grading scale for healthy adult and adolescent volunteers enrolled in preventive vaccine clinical trials. 2007.
Garcon N, Vaughn DW, Didierlaurent AM. Development and evaluation of AS03, an Adjuvant System containing alpha-tocopherol and squalene in an oil-in-water emulsion. Expert Rev Vaccines 2012;11:349-366. doi: 10.1586/erv.11.192.
Guan WJ, Ni ZY, Hu Y, Liang WH, Ou CQ, He JX, Liu L, Shan H, Lei CL, Hui DSC, Du B, Li LJ, Zeng G, Yuen KY, Chen RC, Tang CL, Wang T, Chen PY, Xiang J, Li SY, Wang JL,Liang ZJ, Peng YX, Wei L, Liu Y, Hu YH, Peng P, Wang JM, Liu JY, Chen Z, Li G, Zheng ZJ, Qiu SQ, Luo J, Ye CJ, Zhu SY, Zhong NS; China medical treatment expert group for COVID-19. Clinical characteristics of coronavirus disease 2019 in China. N Engl J Med. 2020 Feb 28. doi: 10.1056/NEJMoa2002032.
Heplisav-B® (Hepatitis B Vaccine [Recombinant], Adjuvanted) solution for intramuscular injection [package insert]. Emeryville (CA): Dynavax Technologies Corporation, 2020[revised May 2020; cited 9 June 2020].
Honda-Okubo Y, Barnard D, Ong CH, Peng BH, Tseng CT, Petrovsky N. Severe acute respiratory syndrome-associated coronavirus vaccines formulated with delta inulin adjuvants provide enhanced protection while ameliorating lung eosinophilic immunopathology. J Virol. 2015 Mar;89(6):2995-3007. doi: 10.1128/JVI.02980-14.
Jackson LA, Campbell JD, Frey SE, Edwards KM, Keitel QA, Kotloff KL, Berry AA, Graham I, Atmar RL, Creech CB, Thomsen IP, Patel SM, Gutierrez AF, Anderson EL, Sahly HM, Hill H, Noah DL, Bellamy AR. Effect of varying doses of a monovalent H7N9 influenza vaccine with and without AS03 and MF59 adjuvants on immune response: a randomised clinical trial. JAMA 2015;314:237-46. doi: 10.1001/jama.2015.7916.
Khurana S, Coyle EM, Manischewitz J, King LR, Gao J, Germain RN, Schwartzberg PL, Tsang JS, Golding H, CHI Consortium. AS03-adjuvanted H5N1 vaccine promotes antibody diversity and affinity maturation, NAI titers, cross-clade H5N1 neutralization, but not H1N1 cross-subtype neutralization. NPJ Vaccines 2018;3:40. doi: 10.1038/s41541-018- 0076-2.
Lake MA. What we know so far: COVID-19 current clinical knowledge and research. Clin Med (Lond). 2020 Mar;20(2):124-127. doi: 10.7861/clinmed.2019-coron.
Lambert P-H, Ambrosino DM, Andersen SR, Baric RS, Black SB, Chen RT, Dekker CL, Didierlaurent AM, Graham BS, Martin SD, Molrine DC, Perlman S, Picard-Fraser PA, Pollard AJ, Qin C, Subbarao K, Cramer JP. Consensus summary report for CEPI/BC March 12-13, 2020 meeting: assessment of risk of disease enhancement with COVID-19vaccines. Vaccine, 2020 May 25. doi: https://doi.org/10.1016/j.vaccine.2020.05.064.
Lansbury L, Smith S, Beyer W, Karamehic E, Pasic-Juhas E, Sikira H, Mateus A, Oshitani H, Zhao H, Beck CR, Nguyen-Van-Tam JS. Effectiveness of 2009 pandemic influenza A(H1N1) vaccines: a systematic review and meta-analysis. Vaccine 2017;35:1996–2006.doi: 10.1016/j.vaccine.2017.02.059.
Launay O, Duval X, Fitoussi S, Jilg W, Kerdpanich A, Montellano M, Schwarz TF, Watanveerade V, Wenzel JJ, Zalcman G, Bambure V, Li P, Caplanusi A, Madan A, Gillard P, Vaughn DW. Extended antigen sparing potential of AS03-adjuvanted pandemic H1N1 vaccines in children, and immunologic equivalence of two formulations

of AS03-adjuvanted H1N1 vaccines: results from two randomised trials. BMC Infect Dis2013;13:435. doi: 10.1186/1471-2334-13-435.
Law B, Sturkenboom M. Safety platform for emergency vaccines (SPEAC). D2.3 Priority list of adverse events of special interest: COVID-19. Report v1.1. 2020 Mar 5.
Law B, Sturkenboom M. Safety platform for emergency vaccines (SPEAC). D2.3 Priority list of adverse events of special interest: COVID-19. Report v2.0. 2020a May 25.
Leroux-Roels I, Borkowski A, Vanwolleghem T, Drame M, Clement F, Hons E, Devaster J-M, Leroux-Roels G. Antigen sparing and cross-reactive immunity with an adjuvanted rH5N1 prototype pandemic influenza vaccine: a randomised controlled trial. Lancet 2007;370:580–9. doi: 10.1016/s0140-6736(07)61297-5.
Liu J, Zheng X, Tong Q, Li W, Wang B, Sutter K, Trilling M, Lu M, Dittmer U, Yang D. Overlapping and discrete aspects of the pathology and pathogenesis of the emerginghuman pathogenic coronaviruses SARS-CoV, MERS-CoV, and 2019-nCoV. J MedVirol. 2020 May;92(5):491-494. doi: 10.1002/jmv.25709.
Liu K, Chen Y, Lin R, Han K. Clinical features of COVID-19 in elderly patients: A comparison with young and middle-aged patients. J Infect. 2020a Mar 27. pii: S0163-4453(20)30116-X. doi: 10.1016/j.jinf.2020.03.005.
Lu S. Timely development of vaccines against SARS-CoV-2. Emerg Microbes Infect. 2020 Mar 8;9(1):542-544. doi: 10.1080/22221751.2020.1737580.
Madan A, Collins H, Sheldon E, Frenette L, Chu L, Friel D, Drame M, Vaughn DW, Innis BL, Schuind A. Evaluation of a primary course of H9N2 vaccine with or without AS03 adjuvant in adults: a phase I/II randomized trial. Vaccine 2017;35:4621–8. doi: 10.1016/j.vaccine.2017.07.013.
Madan A, Ferguson M, Rheault P, Seiden D, Toma A, Friel D, Soni J, Li P, Innis BL, Schuind A. Immunogenicity and safety of an AS03-adjuvanted H7N1 vaccine in adults 65 yearsof age and older: a phase II, observer-blind, randomized, controlled trial. Vaccine 2017a;35:1865–72. doi: 10.1016/j.vaccine.2017.02.057.
Madan A, Ferguson M, Sheldon E, Segall N, Chu L, Toma A, Rheault P, Friel D, Soni J, Li P, Innis BL, Schuind A. Immunogenicity and safety of an AS03-adjuvanted H7N1 vaccinein healthy adults: a phase I/II, observer-blind, randomized, controlled trial. Vaccine 2017b;35:1431–9. doi: 10.1016/j.vaccine.2017.01.054.
McElhaney JE, Beran J, Devaster JM, Esen M, Launay O, Leroux-Roels G, Ruiz-Palacios GM, van Essen GA, Caplanusi A, Claeys C, Durand C, Duval X, Idrissi ME, Falsey AR, Feldman G, Frey SE, Galtier F, Hwang S-J, Innis BL, Kovac M, Kremsner P, McNeil S, Nowakowski A, Richardus JH, Trofa A, Oostvogels L, Influence65 study group. AS03- adjuvanted versus non-adjuvanted inactivated trivalent influenza vaccine against seasonalinfluenza in elderly people: a phase 3 randomised trial. Lancet Infect Dis 2013;13:485- 496. doi: 10.1016/S1473-3099(13)70046-X.
Memish ZA, Perlman S, Van Kerkhove MD, Zumla A. Middle East respiratory syndrome. Lancet. 2020 Mar 28;395(10229):1063-1077. doi: 10.1016/S0140-6736(19)33221-0.
Montplaisir J, Petit D, Quinn MJ, Ouakki M, Deceuninck G, Desautels A, Mignot E, De Wals P. Risk of narcolepsy associated with inactivated adjuvanted AS03A/H1N1 (2009) pandemic influenza vaccine in Québec. PLoS One 2014;9(9):e108489. doi: 10.1371/journal.pone.0108489.

Ng OW, Chia A, Tan AT, Jadi RS, Leong HN, Bertoletti A, Tan YJ. Memory T cell responses targeting the SARS coronavirus persist up to 11 years post-infection. Vaccine. 2016 Apr 12;34(17):2008-14. doi: 10.1016/j.vaccine.2016.02.063.
Nolan T, Izurieta P, Lee BW, Chan PC, Marshall H, Booy R, Drame M, Vaughn DW. Heterologous prime-boost vaccination using an AS03B-adjuvanted influenza A(H5N1) vaccine in infants and children < 3 years of age. J Infect Dis 2014;210(11):1800-10. doi: 10.1093/infdis/jiu359.
Ou X, Liu Y, Lei X, Li P, Mi D, Ren L, Guo L, Guo R, Chen T, Hu J, Xiang Z, Mu Z, Chen X, Chen J, Hu K, Jin Q, Wang J, Qian Z. Characterization of spike glycoprotein of SARS-CoV-2 on virus entry and its immune cross-reactivity with SARS-CoV. Nat Commun. 2020 Mar 27;11(1):1620. doi: 10.1038/s41467-020-15562-9.
Paules CI, Marston HD, Fauci AS. Coronavirus infections-more than just the common cold. JAMA. 2020 Jan 23. doi: 10.1001/jama.2020.0757.
Stebbing J, Phelan A, Griffin I, Tucker C, Oechsle O, Smith D, Richardson P. COVID-19: combining antiviral and anti-inflammatory treatments. Lancet Infect Dis. 2020 Apr;20(4):400-402. doi: 10.1016/S1473-3099(20)30132-8.
Su S, Wong G, Shi W, Liu J, Lai ACK, Zhou J, Liu W, Bi Y, Gao GF. Epidemiology, genetic recombination, and pathogenesis of coronaviruses. Trends Microbiol. 2016 Jun;24(6):490-502. doi: 10.1016/j.tim.2016.03.003.
Tang F, Quan Y, Xin ZT, Wrammert J, Ma MJ, Lv H, Wang TB, Yang H, Richardus JH, Liu W, Cao WC. Lack of peripheral memory B cell responses in recovered patients with severe acute respiratory syndrome: a six-year follow-up study. J Immunol. 2011 Jun 15;186(12):7264-8. doi: 10.4049/jimmunol.0903490.
Thanh Le T, Andreadakis Z, Kumar A, Gómez Román R, Tollefsen S, Saville M, Mayhew S. The COVID-19 vaccine development landscape. Nat Rev Drug Discov. 2020 Apr 9. doi: 10.1038/d41573-020-00073-5.
Vaughn DW, Seifert H, Hepburn A, Dewe W, Li P, Drame M, Cohet C, Innis BL, Fries LF. Safety of AS03-adjuvanted inactivated split virion A(H1N1)pdm09 and H5N1 influenzavirus vaccines administered to adults: pooled analysis of 28 clinical trials. Hum Vaccin Immunother 2014;10(10):2942-57. doi: 10.4161/21645515.2014.972149.
Waddington CS, Walker WT, Oeser C, Reiner A, John T, Wilkins S, Casey M, Eccleston PE, Allen RJ, Okike I, Ladhani S, Sheasby E, Hoschler K, Andrews N, Waight P, Collinson AC, Heath PT, Finn A, Faust SN, Snape MD, Miller E, Pollard AJ. Safety and immunogenicity of AS03B adjuvanted split virion versus non-adjuvanted whole virion H1N1 influenza vaccine in UK children aged 6 months-12 years: open label, randomised,parallel group, multicentre study. BMJ 2010;340:c2649. doi: 10.1136/bmj.c2649.
World Health Organization. WHO Director-General's opening remarks at the media briefing on COVID-19 – 11 March 2020. https://www.who.int/dg/speeches/detail/who-director- general-s-opening-remarks-at-the-media-briefing-on-covid-19 ------------------------ 11-march-2020. Published 11 March 2020. Accessed April 23, 2020.
Yang J, Zheng Y, Gou X, Pu K, Chen Z, Guo Q, Ji R, Wang H, Wang Y, Zhou Y. Prevalence of comorbidities in the novel Wuhan coronavirus (COVID-19) infection: a systematic review and meta-analysis. Int J Infect Dis. 2020 Mar 12. pii: S1201-9712(20)30136-3. doi: 10.1016/j.ijid.2020.03.017.
Yang WH, Dionne M, Kyle M, Aggarwal N, Li P, Madariaga M, Godeaux O, Vaughn DW. Long term immunogenicity of an AS03-adjuvanted influenza A(H1N1)pdm09 vaccine in

young and elderly adults: an observer-blind, randomised trial. Vaccine 2013;31:4389-4397. doi: 10.1016/j.vaccine.2013.07.007.
Yin JK, Khandaker G, Rashid H, Heron L, Ridda I, Booy R. Immunogenicity and safety of pandemic influenza A (H1N1) 2009 vaccine: systematic review and meta-analysis. Influenza Other Respir Viruses 2011;5(5):299-305. doi: 10.1111/j.1750-2659.2011.00229.x.
Zhu N, Zhang D, Wang W, Li X, Yang B, Song J, Zhao X, Huang B, Shi W, Lu R, Niu P, Zhan F, Ma X, Wang D, Xu W, Wu G, Gao GF, Tan W; China novel coronavirus investigatingand research team. A novel coronavirus from patients with pneumonia in China, 2019. NEngl J Med. 2020 Feb 20;382(8):727-733. doi: 10.1056/NEJMoa2001017.

APPENDICES

The clinical documents included in the appendices are meant as examples only; the actual documents used during the trial may differ slightly.

Appendix 1 – Sample Ruler to Measure Local Adverse Events

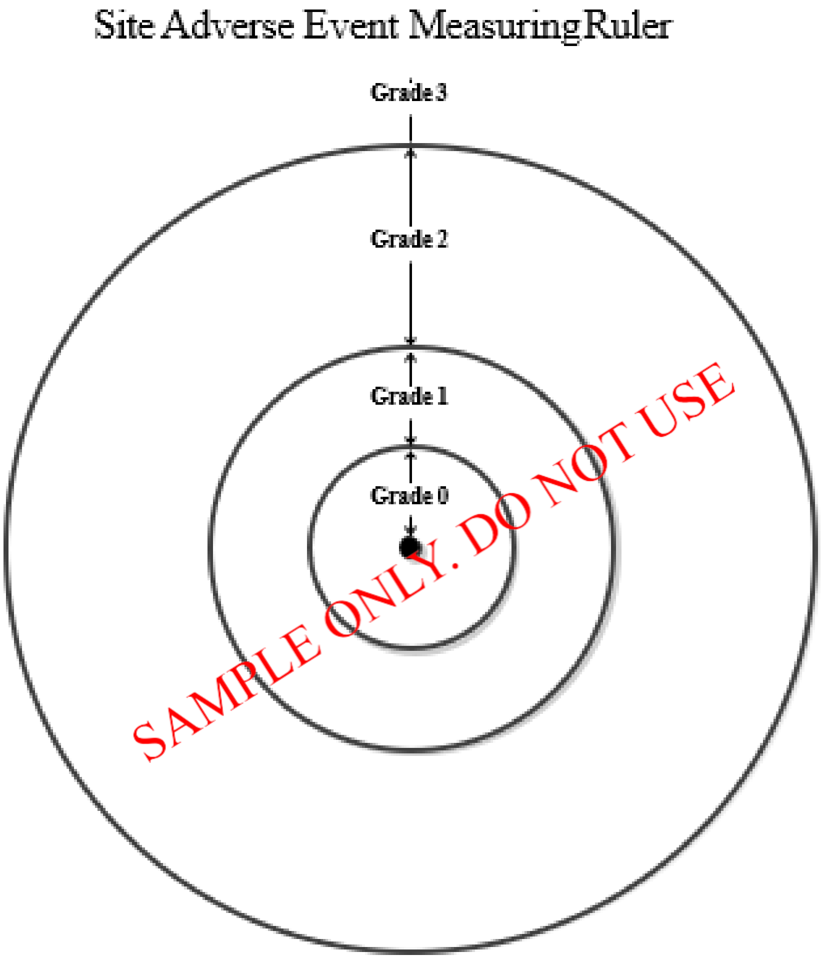

Grading for redness and swelling where the vaccine was given	
Grade 0	None or less than 25 mm
Grade 1	Between 25 mm and 50 mm
Grade 2	Between 51 and 100 mm
Grade 3	More than 100 mm
Grade 4	skin/tissue loss at the injection site (blister or ulcer formation) of any size

Appendix 2 – Subject Diary Sample Pages

DIARY (DAY 0 to DAY 7)	
**Study Name**	A Randomized, Partially-Blinded, Dose-Ranging Phase 1 Study to Assess the Safety, Tolerability, and Immunogenicity of a Recombinant Coronavirus-Like Particle COVID-19 Vaccine in Adults 18-55 Years of Age
**Protocol Number**	CP-PRO-CoVLP-019
**Sponsor**	Medicago R&D Inc.<br>1020, route de l'Église, bureau 600<br>Québec (Qc), Canada G1V 3V9
**Clinical site Address**	
**Principal Investigator Name**	
**Clinical Research Coordinator**	
**24-hour Emergency Number**	

SAMPLE ONLY. DO NOT USE

DIARY (DAY 0 to DAY 7) INSTRUCTIONS

You should fill in your Diary at approximately the same time, preferably in the evening.
You will need to refer to this Diary during the Telephone Calls (on Day 1 and Day 8).
You need to bring this Diary with you at the Day 3 and Day 21 visits.

Fill in the Day 0 to Day 7 tables EVERY DAY for the first 7 days after you receive your vaccine dose.
Fill in each day's column by entering the WORST grade for each symptom that you had during the period.
Grayed out sections are to be left blank and will be completed by the site personnel.
To complete this document, use ink. No pencil or liquid paper is allowed.
If you made a mistake, cross out the incorrect information, initial and date, and write the correct answerbeside.
Date should be recorded with 2 digits for day, 3 letters for month and 4 digits for year

January	JAN	April	APR	July	JUL	October	OCT
February	FEB	May	MAY	August	AUG	November	NOV
March	MAR	June	JUN	September	SEP	December	DEC

SAMPLE ONLY, DO NOT USE

TEMPERATURE:

To record the temperature, use the thermometer you were given at the clinic. Do not drink, eat food orsmoke prior to taking your temperature.
You should take your temperature for the first 7 days following vaccination at approximately the sametime each evening and at any other time if you feel feverish.
You will need to record the <u>highest temperature of the day</u> on page 4.
If your temperature is 38°C or 100.4°F or higher, you are allowed to take over-the-counter antipyretics (e.g., acetaminophen/paracetamol, aspirin, naproxen, or ibuprofen) and you should increase the frequencyof temperature measurements to approximately every 4 hours, until you don't have a fever anymore.
Medication intake needs to be documented in this document and will be reviewed by the site personnel.

DIARY (DAY 0 to DAY 7) INSTRUCTIONS

SYMPTOMS:

Complete the table referring to the following periods:
For the 1st column: the period between the vaccination and the 30 minutes post-vaccination; For the 2nd column: the period between the last evaluation (30 minutes post-vaccination) andthe evening;
For the remaining columns: the period since the previous evaluation of the symptoms.
If the symptom is not present, indicate 0.
If the symptom is present, a grade should be indicated.
Evaluate the grade of all other symptoms according to the definitions:
0= No symptom
1= Does not interfere with activity
2= Repeated use of non-narcotic pain reliever (e.g. Advil, Tylenol) >24 hours or interfereswith activity but does not require medical intervention
3= Any use of narcotic pain reliever (e.g. codeine, morphine) or prevents daily activityand require medical intervention
4= Visit to Emergency room or hospitalization
List any other problems (not listed on pages 4 to 6) on the Memory Aid (Day 0 to Day 21) page “sideeffects (symptoms)”.
Symptoms listed on pages 4 to 6 which persist longer than 7 days after study vaccine administrationmust also be listed on the Memory Aid (Day 0 to Day 21) page “side effects (symptoms)”.

SAMPLE ONLY. DO NOT USE

You received the study vaccine on: |__|__| |__|__|__| |__|__|__|__| at: |__|__| : |__|__|
dd mmm yyyy hh mm

DIARY (DAY 0 to DAY 7)										
**Day 0 to Day 7**		**30 min, Post-dose**	**Evening, day of dose**	**1st Day after dose**	**2nd Day after dose**	**3rd Day after dose**	**4th Day after dose**	**5th Day after dose**	**6th Day after dose**	**7th Day after dose**
**Date (dd-mmm-yyyy)**										
Oral Temperature	°C / °F									
	Causality									
Redness where the injection was given	Grade									
	Causality									
	Use the measuring tool given to you at the site to estimate the size of the red area around the injection site and indicate the grade above.									
Swelling where the injection was given	Grade									
	Causality									
	Use the measuring tool given to you at the site to estimate the size of the swollen area around the injection site and indicate the grade above.									
Pain at vaccine injection site	Grade									
	Causality									
	**0=** No symptom; **1=** Does not interfere with activity; **2=** Repeated use of non-narcotic pain reliever >24 hours or interferes with activity; **3=** Any use of narcotic pain reliever or prevents daily activity; **4=** Visit to Emergency room or hospitalization.									
**Comments:**										

MD initials and date: |__|__|__|__|__|__|__|__|__|
dd mmm yyyy

LE ON
OT USE

DIARY (DAY 0 to DAY 7)

Day 0 to Day 7		30 min, Post-dose	Evening, day of dose	1st Day after dose	2nd Day after dose	3rd Day after dose	4th Day after dose	5th Day after dose	6th Day after dose	7th Day after dose
Headache	Grade									
	Causality									
**GRADES** **0=** No symptom; **1=** Does not interfere with activity; **2=** Repeated use of non-narcotic pain reliever >24 hours or interferes with activity; **3=** Any use of narcotic pain reliever or prevents daily activity; **4=** Visit to Emergency room or hospitalization.										
Muscle aches	Grade									
	Causality									
**GRADES** **0=** No symptom; **1=** Does not interfere with activity; **2=** Interferes with activity; **3=** Prevents daily activity; **4=** Visit to Emergency room or hospitalization.										
Fatigue	Grade									
	Causality									
**GRADES** **0=** No symptom; **1=** Does not interfere with activity; **2=** Interferes with activity; **3=** Prevents daily activity; **4=** Visit to Emergency room or hospitalization.										
Joint aches	Grade									
	Causality									
**GRADES** **0=** No symptom; **1=** Does not interfere with activity; **2=** Interferes with activity but does not require medical intervention; **3=** Prevents daily activity and requires medical intervention; **4=** Visit to Emergency room or hospitalization.										
**Comments:**										
**MD initials and date:**				|__|__| dd	|__|__|__| mmm	|__|__|__|__| yyyy				

E ONL
T USE

DIARY (DAY 0 to DAY 7)

**Day 0 to Day 7**		**30 min, Post-dose**	**Evening, day of dose**	**1st Day after dose**	**2nd Day after dose**	**3rd Day after dose**	**4th Day after dose**	**5th Day after dose**	**6th Day after dose**	**7th Day after dose**
Chills	Grade									
	Causality									
Feelings of general discomfortor uneasiness	Grade									
	Causality									
Feeling of swelling in the neck	Grade									
	Causality									
Feeling of swelling in the axilla (armpit)	Grade									
	Causality									

SAMPLE ONLY. DO NOT USE

GRADES **0=** No symptom; **1=** Does not interfere with activity; **2=** Interferes with activity but does not require medical intervention; **3=** Prevents daily activity and requires medical intervention; **4=** Visit to Emergency room or hospitalization.

I confirm I have reviewed the 3 pages of solicited symptoms and evaluated the causalities when needed.

MD initials and date: |__|__|__|__|__|__|__|__|__|
dd mmm yyyy

Comments:

MD initials and date: |__|__|__|__|__|__|__|__|__|
dd mmm yyyy

Appendix 3 – Subject Memory Aid Sample Pages

MEMORY AID (DAY 0 to DAY 42)	
**Study Name**	A Randomized, Partially-Blinded, Dose-Ranging Phase 1 Study to Assess the Safety, Tolerability, and Immunogenicity of a Recombinant Coronavirus-Like Particle COVID-19 Vaccine in Adults 18-55 Years of Age
**Protocol Number**	CP-PRO-CoVLP-019
**Sponsor**	Medicago R&D Inc.<br>1020, route de l'Église, suite 600<br>Québec, QC, Canada G1V 3V9
**Clinical site Address**	
**Principal Investigator Name**	
**Clinical Research Coordinator**	
**24-hour Emergency Number**	

SAMPLE ONLY. DO NOT USE

MEMORY AID (DAY 0 to DAY 42) INSTRUCTIONS

SIDE EFFECTS (Symptoms) and MEDICATION:

Use the space provided to record important information that you want to tell us about changes in your health. Note any health problems you experience, worsening of previous problems, or new medicines you are taking. Examples may include prescription drugs (including vaccines), over- the-counter drugs, herbal supplements and/or vitamins. Indicate each intake of medication you are usually taking as needed.

It is especially important that you make a note of problems that required a visit to your doctor, a hospital stay, or a visit to an emergency room.

It is also important that you make a note of changes in your medications.

Each medication must be associated to a problem, but each problem does not require a medication associated with it.

Indicate the start and stop date (when available) for each symptom and medication. However, if it is still ongoing at the Day 21 or Day 42 visits, you will be required to tick the ongoing checkbox.

Please contact the study site if you experience any severe effects or if you have any concerns at any time by using the emergency contact number provided on the first page of this Memory Aid.

SAMPLE ONLY. DO NOT USE

You received the first study vaccine on: |__|__|__|__|__|__|__|__|__| at: |__|__| : |__|__|
dd mmm yyyy hh mm

You received the second study vaccine on: |__|__|__|__|__|__|__|__|__| at: |__|__| : |__|__|
dd mmm yyyy hh mm

COVID-19 INFORMATION

SYMPTOMS FOR COVID-19:

The symptoms of COVID-19 are similar to the flu. These symptoms include:Fever;
Cough;
Shortness of breath or difficulty breathing;Fatigue;
Chills
Muscle or body aches;Headache;
New loss of taste or smell;Sore throat;
Congestion or runny nose;Nausea or
vomiting; Diarrhea.

Individuals who have COVID-19 may show some symptoms or none at all.
Symptoms for COVID-19 can appear up to 14 days after exposure to the virus.

SAMPLE ONLY. DO NOT USE

EXPOSURE TO COVID-19:

Contact the study clinic site if you:
Develop any of the above listed symptoms for COVID-19;
Develop fever by itself or with one or more of the following symptoms in the first three days after vaccination:Chills;
Muscle aches;Malaise;
Are tested positive for COVID-19;
Come into contact with someone known to have COVID-19.
You will be asked to follow all of the recommended actions (e.g. quarantine, re-testing) by local Public Health authorities.

MEMORY AID (DAY 0 to DAY 21)

Day 0 to Day 21 ☐ Nothing to report

#	Side Effects (symptoms)	**Grade** ($See below)	**Date and timeit started**	**Date and timeit ended**	**Did you receive medical care?**	**Validated with subject**
			dd mmm yyyy<br>hh : mm	dd mmm yyyy<br>hh : mm<br>❑ Ongoing at Day 21	Yes<br>No	Initial and date:<br>dd mmm yyyy
			dd mmm yyyy<br>hh : mm	dd mmm yyyy<br>hh : mm<br>Ongoing at Day 21	Yes<br>No	Initial and date:<br>dd mmm yyyy

SAMPLE ONLY. DO NOT USE

**GRADES**	**0=** No symptom; **1=** Does not interfere with activity; **2=** Repeated use of non-narcotic pain reliever >24 hours or interferes with activity but does not require medical intervention; **3=** Any use of narcotic pain reliever or prevents daily activity and does require medical intervention;**4=** Visit to Emergency room or hospitalization.

Comments:

MD initials and date: dd mmm yyyy

MEMORY AID (DAY 0 to DAY 21)

Day 0 to Day 21

☐ Nothing to report

**Medication** # (Name, Dose, Route and Frequency)	**Start date**	**Stop date**	**Reason(s) why you are taking this medication?**	**Validated with subject**
				Initial and date:
	dd mmm yyyy	dd mmm yyyy<br>Ongoing at Day 21		dd mmm yyyy
				Initial and date:
	dd mmm yyyy	dd mmm yyyy<br>Ongoing at Day 21		dd mmm yyyy
				Initial and date:
	dd mmm yyyy	dd mmm yyyy<br>Ongoing at Day 21		dd mmm yyyy

**Dose**			**Route**			**Frequency**		
Capsule	Tablespoon	g	Oral	Intramuscular	Ophthalmic	Once a day	Every other day	Continuous
Tablet	Teaspoon	mcg	Topical	Nasal	Intra-articular	Twice a day	Every 3 days	Intermittent
Ring	Puff	mg	Inhalation	Intravenous	Rectal	Three times/day	Every 2 weeks	Once
Application	Drops	IU	Intrauterine	Transdermal	Sublingual	1x per week	Every 6 months	As needed
Lozenge	mL	Units	Vaginal	Subcutaneous	Unknown	2x per week	Every hour	Unknown
Syringe	%	Unknown				Once a month	Once a year	

Comments:

MD initials and date:

dd mmm yyyy

SAMPLE ONLY. DO NOT USE

MEMORY AID (DAY 22 to DAY 42)

Day 22 to Day 42 ☐ Nothing to report

#	Side Effects (symptoms)	**Grade** (See below)	**Date and timeit started**	**Date and timeit ended**	**Did you receive medical care?**	**Validated with subject**
			dd mmm yyyy<br>: hh mm<br>Ongoing at Day 42	dd mmm yyyy<br>: hh mm	Yes<br>No	Initial and date:<br>dd mmm yyyy
			dd mmm yyyy<br>: hh mm<br>Ongoing at Day 42	dd mmm yyyy<br>: hh mm	Yes<br>No	Initial and date:<br>dd mmm yyyy

SAMPLE ONLY. DO NOT USE

GRADES **0=** No symptom; **1=** Does not interfere with activity; **2=** Repeated use of non-narcotic pain reliever >24 hours or interferes with activity but does not require medical intervention; **3=** Any use of narcotic pain reliever or prevents daily activity and does require medical intervention; **4=** Visit to Emergency room or hospitalization.

Comments:

MD initials and date:
dd mmm yyyy

MEMORY AID (DAY 22 to DAY 42)

Day 22 to Day 42 ☐ Nothing to report

#	**Medication** (Name, Dose, Route and Frequency)	**Start date**	**Stop date**	**Reason(s) why you are taking this medication?**	**Validated with subject**
		dd mmm yyyy<br>Ongoing at Day 42	dd mmm yyyy		Initial and date:<br>dd mmm yyyy
		dd mmm yyyy	dd mmm yyyy<br>Ongoing at Day 42		Initial and date:<br>dd mmm yyyy
		dd mmm yyyy<br>Ongoing at Day 42	dd mmm yyyy		Initial and date:<br>dd mmm yyyy

SAMPLE ONLY. DO NOT USE

**Dose**			**Route**			**Frequency**		
Capsule	Tablespoon	g	Oral	Intramuscular	Ophthalmic	Once a day	Every other day	Continuous
Tablet	Teaspoon	mcg	Topical	Nasal	Intra-articular	Twice a day	Every 3 days	Intermittent
Ring	Puff	mg	Inhalation	Intravenous	Rectal	Three times/day	Every 2 weeks	Once
Application	Drops	IU	Intrauterine	Transdermal	Sublingual	1x per week	Every 6 months	As needed
Lozenge	mL	Units	Vaginal	Subcutaneous	Unknown	2x per week	Every hour	Unknown
Syringe	%	Unknown				Once a month	Once a year	

Comments:

MD initials and date:
dd mmm yyyy

Appendix 4 – Three-Day Safety Summary (Sample)

Study Description

Brief statement of the purpose of trial.

Recruitment Status

Listings of enrollment status by site – (# screened, # enrolled, # vaccinated, #withdrawn or dropped out).

Safety Data Listings – present data by subject

Severity (including hospitalization), duration, and relationship of any AEs;

Vital signs abnormalities;

Safety lab abnormalities;

Concomitant medication;

Physical examination abnormalities.

Safety Data – present data by treatment groups

Safety data overview (number of subjects experiencing AEs, SAEs, solicited AEs, unsolicited AEs, related AEs, lab abnormalities, vital signs abnormalities, physical examination abnormalities);

Present solicited AEs and solicited Grade 3-4 AEs;

Present related-solicited AEs and related-solicited Grade 3-4 AEs;

Present unsolicited non-serious AEs and unsolicited non-serious Grade 3-4 AEs;

Present related-unsolicited non-serious AEs and related-unsolicited none-serious Grade 3-4 AEs;

Present SAEs and related-SAEs by SOC/PT;

Present COVID-19 symptoms.

Appendix 5 – List of Potential Immune-Mediated Diseases

Neuroinflammatory disorders	Musculoskeletal disorders	Skin disorders
Cranial nerve neuropathy, including paralysis and paresis(eg, Bell's palsy).<br>Optic neuritis.<br>Multiple sclerosis.<br>Transverse myelitis.<br>Guillain-Barré syndrome, including Miller Fisher syndrome and other variants.<br>Acute disseminated encephalomyelitis, including site specific variants, eg, noninfectious encephalitis, encephalomyelitis, myelitis, myeloradiculoneuritis.<br>Myasthenia gravis, including Lambert-Eaton myasthenic syndrome.<br>Demyelinating peripheral neuropathies and plexopathies including:<br>Chronic inflammatory demyelinating polyneuropathy.<br>Multifocal motor neuropathy.<br>Polyneuropathies associatedwith monoclonal gammopathy.<br>Narcolepsy.<br>Tolosa Hunt syndrome.	Systemic lupus erythematosusand associated conditions.<br>Systemic scleroderma (systemic sclerosis), including:<br>Diffuse scleroderma.<br>CREST syndrome.<br>Idiopathic inflammatory myopathies, including:<br>Dermatomyositis.<br>Polymyositis.<br>Anti-synthetase syndrome.<br>Rheumatoid arthritis and associated conditions including:<br>Juvenile idiopathic arthritis.<br>Still's disease.<br>Polymyalgia rheumatica.<br>Spondyloarthropathies, including:<br>Ankylosing spondylitis.<br>Reactive arthritis (Reiter's syndrome).<br>Undifferentiated spondyloarthritis.<br>Psoriatic arthritis.<br>Enteropathic arthritis.<br>Relapsing polychondritis.<br>Mixed connective tissue disorder.<br>Gout.	Psoriasis.<br>Vitiligo.<br>Erythema nodosum.<br>Autoimmune bullous skin diseases (including pemphigus,pemphigoid, and dermatitis herpetiformis).<br>Lichen planus.<br>Sweet's syndrome.<br>Localized scleroderma (morphea).<br>Cutaneous lupus erythematosus.<br>Rosacea.
**Vasculitis**	**Blood disorders**	**Others**
Large vessels vasculitis including:<br>Giant cell arteritis (temporal arteritis).<br>Takayasu's arteritis.<br>Medium sized and/or small vessels vasculitis including:<br>Polyarteritis nodosa.<br>Kawasaki's disease.<br>Microscopic polyangiitis.<br>Wegener's granulomatosis (granulomatosis with polyangiitis).	Autoimmune hemolytic anemia.<br>Autoimmune thrombocytopenia.<br>Antiphospholipid syndrome.<br>Pernicious anemia.<br>Autoimmune aplastic anemia.<br>Autoimmune neutropenia.<br>Autoimmune pancytopenia.	Autoimmune glomerulonephritis including:<br>IgA nephropathy.<br>Glomerulonephritis rapidly progressive.<br>Membranous glomerulonephritis.<br>Membranoproliferative glomerulonephritis.<br>Mesangioproliferative glomerulonephritis.<br>Tubulointerstitial nephritisand uveitis syndrome.

Vasculitis (continued)	Blood disorders (continued)	Others (continued)
Churg–Strauss syndrome (allergic granulomatous angiitis or eosinophilic granulomatosis with polyangiitis).<br>Buerger's disease (thromboangiitis obliterans).<br>Necrotizing vasculitis (cutaneous or systemic).<br>Antineutrophil cytoplasmic antibody (ANCA) positive vasculitis (type unspecified).<br>Henoch-Schonlein purpura(IgA vasculitis).<br>Behcet's syndrome.<br>Leukocytoclastic vasculitis.		Ocular autoimmune diseases including:<br>Autoimmune uveitis.<br>Autoimmune retinitis.<br>Autoimmune myocarditis/cardiomyopathy.<br>Sarcoidosis.<br>Stevens-Johnson syndrome.<br>Sjögren's syndrome.<br>Alopecia areata.<br>Idiopathic pulmonary fibrosis.<br>Goodpasture syndrome.<br>Raynaud's phenomenon.
**Liver disorders**	**Gastrointestinal disorders**	**Endocrine disorders**
Autoimmune hepatitis.<br>Primary biliary cirrhosis.<br>Primary sclerosing cholangitis.<br>Autoimmune cholangitis.	Inflammatory bowel disease, including:<br>Crohn's disease.<br>Ulcerative colitis.<br>Microscopic colitis.<br>Ulcerative proctitis.<br>Celiac disease.<br>Autoimmune pancreatitis.	Autoimmune thyroiditis (including Hashimoto thyroiditis).<br>Grave's or Basedow's disease.<br>Diabetes mellitus type 1.<br>Addison's disease.<br>Polyglandular autoimmune syndrome.<br>Autoimmune hypophysitis.

Statistical Analysis Plan

Sponsor:	Medicago		
**Protocol:**	CP-PRO-CoVLP-019		
**Document Version No.:**	3.0	**Document Date:**	01-OCT-2020

Statistical Analysis Plan

Protocol CP-PRO-COVLP-019

A Randomized, Partially-Blinded, Dose-Ranging Phase 1 Study to Assess the Safety, Tolerability, and Immunogenicity of a Recombinant Coronavirus-Like Particle COVID-19 Vaccine in Adults 18-55 Years of Age

Protocol Number:
(Version Date) Final version 3.0 (21-SEP-2020)

Investigational Product: Coronavirus-Like Particle (CoVLP) COVID-19 Vaccine

Phase: 1

Methodology: Partially-Blinded, Dose-Ranging Study

Sponsor: Medicago R&D Inc.
1020 route de l'Église, bureau 600Québec (Qc), Canada G1V 3V9

Sponsor Representative: Annie Seguin, Manager - Biometrics, Clinical Studies

Sponsor Representative: Alexander Makarkov, Medical Director

Document Date: 01-OCT-2020

Document Version: Final version 3.0

Medicago/Cytel Confidential: This is a controlled document and shall not be changed, saved, copied or reproduced by any unauthorized person.

Confidential Information

SIGNATURE PAGE

Protocol Title: A Randomized, Partially-Blinded, Dose-Ranging Phase 1 Study to Assess the Safety, Tolerability, and Immunogenicity of a Recombinant Coronavirus-Like Particle COVID-19 Vaccine in Adults 18-55 Years of Age

Sponsor: Medicago R&D Inc.
1020 route de l'Église, bureau 600
Québec (Qc), Canada G1V 3V9

Protocol Number: CP-PRO-COVLP-019

Document Date/Version: 28-SEP-2020/final v3.0

Cytel, Inc. Author:
Cytel, Inc.
675 Massachusetts Avenue
Cambridge, MA 02139

Virginie Jego

Signature:____

Date:____

Sponsor Approval

By signing this document, I acknowledge that I have read the document and approve of the planned statistical analyses described herein. I agree that the planned statistical analyses are appropriate for this study, are in accordance with the study objectives, and are consistent with the statistical methodology described in the protocol, clinical development plan, and all applicable regulatory guidance's and guidelines.

I have discussed any questions I have regarding the contents of this document with the biostatistical author.

I also understand that any subsequent changes to the planned statistical analyses, as described herein, may have a regulatory impact and/or result in timeline adjustments. All changes to the planned analyses will be described in the clinical study report (CSR).

Sponsor Signatory:

Trevor Newhook, Lead Biostatistician
Medicago R&D Inc.
1020 route de l'Église, bureau 600
Québec (Qc), Canada G1V 3V9

Signature:____

Date:____

Medicago/Cytel Confidential: This is a controlled document and shall not be changed, saved, copied or reproduced by any unauthorized person.

Statistical Analysis Plan

**Sponsor:**	Medicago		
**Protocol:**	CP-PRO-CoVLP-019		
**Document Version No.:**	3.0	**Document Date:**	01-OCT-2020

Alexander Makarkov, Medical Director
Medicago R&D Inc.
1020 route de l'Église, bureau 600
Québec (Qc), Canada G1V 3V9

Signature:_______________________________________

Date:____

Medicago/Cytel *Confidential: This is a controlled document and shall not be changed, saved, copied or reproduced by any unauthorized person.*

TABLE OF CONTENTS

INTRODUCTION AND OBJECTIVES OF ANALYSIS 11

INTRODUCTION 11

OBJECTIVES OF STATISTICAL ANALYSIS 11

STUDY DESIGN 13

SYNOPSIS OF STUDY DESIGN 13

STUDY OBJECTIVES 14

SAMPLE SIZE JUSTIFICATION 16

SUBJECT SELECTION 16

Inclusion Criteria 16

Exclusion Criteria 18

RANDOMIZATION METHODOLOGY 21

UNBLINDING 21

STOPPING RULES 22

STUDY PROCEDURES 22

SAFETY AND IMMUNOGENICITY ENDPOINTS AND VARIABLE DEFINITION 25

Safety endpoints 25

Immunogenicity endpoints 32

OVERVIEW OF PLANNED ANALYSIS 34

ANALYSIS SETS 36

POPULATION DEFINITIONS 36

PROTOCOL DEVIATIONS 37

STATISTICAL METHODS 38

RESPONSIBILITIES 38

GENERAL STATISTICAL METHODS AND DATA HANDLING 38

General Methods 38

Computing Environment 40

Medicago/Cytel Confidential: This is a controlled document and shall not be changed, saved, copied or reproduced by any unauthorized person.

Methods of Pooling Data 40

Adjustments for Covariates 40

Multiple Comparisons/Multiplicity ... 40

Subpopulations 41

Withdrawals, Dropouts, Loss to Follow-up ... 41

Missing, Unused, and Spurious Data ... 41

Visit Windows 42

Definition 42

Computation of Derived data ... 43

Handling of reported values with “<” sign or values reported as Below Limit of Quantification (BLQ) ... 43

SUBJECT DISPOSITION ... 44

DEMOGRAPHIC AND BASELINE CHARACTERISTICS ... 44

Demographics 44

Protocol Deviations 45

Medical History 45

Prior and Concomitant Medications ... 45

EXPOSURE ANALYSIS ... 46

SAFETY ANALYSES ... 47

Adverse Events 47

Adverse Events by subgroups ... 51

Laboratory Data 52

Vital Signs 54

Physical Examinations 54

Electrocardiogram 54

COVID-19 Assessment or Experience ... 55

Exploratory Safety Analysis ... 55

IMMUNOGENICITY ANALYSIS ... 55

Analysis of Primary Endpoints ... 56

Medicago/Cytel Confidential: This is a controlled document and shall not be changed, saved, copied or reproduced by any unauthorized person.

Analysis of Secondary Endpoints 59
Analysis of Exploratory Endpoints 60
CHANGES TO PLANNED ANALYSES 62
QUALITY CONTROL 63
REFERENCES 64
APPENDICES 65
APPENDIX 1 – DAY 21 DATA TRANSFER 65
APPENDIX 2 – DAY 42 ANALYSIS 66
Tables and Figures to be generated 66
Data Listings to be generated 71
APPENDIX 3 – DAY 201 ANALYSIS (INTERIM ANALYSIS) 74
Tables and Figures to be generated 74
Data Listings to be Generated 76
APPENDIX 4 – DAY 386 ANALYSIS (FINAL ANALYSIS) 78
Tables and Figures to be generated 78
Data Listings to be Generated 80
APPENDIX 5 - LIST OF POTENTIAL IMMUNE-MEDIATED DISEASES 81
APPENDIX 6 - ORDERING OF SYSTEM ORGAN CLASS 83

Medicago/Cytel Confidential: This is a controlled document and shall not be changed, saved, copied or reproduced by any unauthorized person.

LIST OF IN-TEXT TABLES

Table **Page**

Table 1: Study Treatment Design....13

Table 2: Schedule of Assessments....23

Table 3: Safety Variable Definitions....27

Table 4 - Clinical Laboratory Tests and SI units....52

Medicago/Cytel Confidential: This is a controlled document and shall not be changed, saved, copied or reproduced by any unauthorized person.

ABBREVIATIONS

Abbreviation	Definition
ADaM	Analysis Data Model
AE	Adverse Event
AESI	Adverse Event of Special Interest
ANCOVA	Analysis of Covariance
ANOVA	Analysis of Variance
ATC	Anatomical Therapeutic Chemical
BMI	Body Mass Index
CI	Confidence Interval
CMI	Cell-Mediated Immune (response)
CSR	Clinical Study Report
DBP	Diastolic Blood Pressure
eCRF	electronic Case Report Form
ELISA	Enzyme-Linked Immunosorbent Assay
ELISpot	Enzyme-Linked Immuno Spot Assay
EOS	End of Study
FDA	Food and Drug Administration
GMT	Geometric Mean Titer
GMFR	Geometric Mean Folder Rise
HR	Heart rate
ICH	International Conference on Harmonisation
IDMC	Independent Data Monitoring Committee
IFN-γ	Interferon Gamma
IL	Interleukin
IM	Intramuscular
ITT	Intent-to-treat
LL	Lower Limit
MedDRA	Medical Dictionary for Regulatory Activities
MCH	Mean cell haemoglobin
MCHC	Mean cell haemoglobin concentration
MCV	Mean cell volume

Medicago/Cytel Confidential: This is a controlled document and shall not be changed, saved, copied or reproduced by any unauthorized person.

Abbreviation	Definition
MPV	Mean Platelet Volume
Nab	Neutralizing antibody
OT	Oral Temperature
PP	Per Protocol
PT	Preferred Term
Q1	1st Quartile
Q3	3rd Quartile
RR	Respiratory Rate
SAE	Serious Adverse Event
SAP	Statistical Analysis Plan
SARS-CoV-2	Serious Acute Respiratory Syndrome Coronavirus-2
SAS	Safety Analysis Set
SBP	Systolic Blood Pressure
SC	Seroconversion
SD	Standard Deviation
SDTM	Study Data Tabulation Model
SOC	System Organ Class
Th	T helper
UL	Upper Limit
VED	Vaccine-Enhanced Disease
VLP WBC	Virus-Like ParticleWhite Blood Count
WHO DDE	World Health Organization Drug Dictionary Enhanced

Medicago/Cytel Confidential: This is a controlled document and shall not be changed, saved, copied or reproduced by any unauthorized person.

MODIFICATION HISTORY

SAP Version	Date	Author	Changes from Previous version
1.0	03-Jul-2020	Virginie Jego	Not Applicable
2.0		Virginie Jego	Clarification that Neutralizing Antibodies data provided by Nexelis laboratory will be considered as the primary endpoint (section 2.9.2)<br>Clarification regarding definition of the VED AESI category (section 2.9.1)<br>Identification of topline results for Day42 analysis (section 9.2 Appendix 2)<br>Addition of one listing displaying outcome of SARS-Cov-2 test overtime(section 5.6.7)<br>Correction of typos
3.0			Analysis of IFN-γ ELISpot and IL-4 ELISpot parameters:<br>Due to the limited availability of sample material, the following updated are made Summary statistics at Day 0 presented across treatment armand not by treatment arm.<br>Comparison of response between timepoints has beenremoved<br>Implementation of changes reflected in protocol amendment 2.0:<br>Extension of follow up to Day386

Medicago/Cytel Confidential: This is a controlled document and shall not be changed, saved, copied or reproduced by any unauthorized person.

INTRODUCTION AND OBJECTIVES OF ANALYSIS

Introduction

Medicago has developed a plant-based system (*Nicotiana benthamiana*) for transient expressionof recombinant viral proteins, and this system has been used to produce virus-like particles (VLPs)bearing the SARS-CoV-2 S glycoprotein. This Phase 1 study is intended to assess the safety and immunogenicity profile of the Coronavirus-Like Particle (CoVLP) COVID-19 Vaccine in healthy adults and unblinded results generated from this study will inform design decisions (i.e. most safeand effective dose level[s] and regimen[s], more effective adjuvant) of a subsequent Phase 2 study.

The study will be a randomized, partially-blinded, prime-boost, staggered dose-escalation Phase 1study intended to assess the safety, tolerability, and immunogenicity of the Coronavirus-Like Particle COVID-19 Vaccine at three dose levels (3.75 µg, 7.5 µg, and 15 µg VLP) unadjuvantedor adjuvanted with either CpG 1018 or AS03 in healthy adults 18 to 55 years of age, who have been tested for the absence of SARS-CoV-2 antibodies.

Objectives of Statistical Analysis

This statistical analysis plan (SAP) is designed to outline the statistical methodology to be used in the Day 42, interim (Day 201) and final (Day 386) analyses of data collected for CP-PRO- COVLP-019 protocol. The statistical analyses and summary tabulations described in this SAP will provide the basis for the results sections of the Day 42 report, Day 201 clinical study report (CSR) and (Day 386) addendum CSR for this study. Analyses to be produced in the context of the Independent Data Monitoring Committee (IDMC) will not be covered in this document but specified outside of this document in a dedicated SAP.

Analysis sets for analyses, data handling rules, statistical methods, and formats for data presentation are provided in this document. This SAP will also outline any differences in the currently planned analytical objectives relative to those planned in the study protocol.

This SAP is developed based on the following study documents:

Protocol version 3.0 (original protocol, dated 21 September 2020)

Electronic Case Report Form (eCRF) version 2.0

The statistical analyses will be performed in accordance with International Conference on Harmonisation (ICH) E9 guidelines. This SAP conforms to the Cytel standard operating procedure CRS-BS-SOP-000002 *Timing and Content of Statistical Analysis Pla*ns. The SAP will

Medicago/Cytel Confidential: This is a controlled document and shall not be changed, saved, copied or reproduced by any unauthorized person.

be approved and signed before the Day 42 analysis database lock and before study unblinded tominimize the risk of bias in determination of the analytic methods.

Specifications of outputs (Tables, Listings and Figures shells) will be described in a separatedocument.

Medicago/Cytel Confidential: This is a controlled document and shall not be changed, saved, copied or reproduced by any unauthorized person.

STUDY DESIGN

Synopsis of Study Design

This is a randomized, partially-blinded, prime-boost, staggered dose-escalating study with approximately 180 healthy male and female subjects 18 to 55 years of age, who have been testedfor the absence of SARS-CoV-2 antibodies. Subjects will be enrolled and equally randomized into one of the nine treatment groups: Coronavirus-Like Particle COVID-19 Vaccine at either 3.75 µg, 7.5 µg, or 15 µg, unadjuvanted or adjuvanted with CpG 1018 or AS03 as summarized in Table 1. A total of 20 subjects will be treated in each treatment group.

Table 1: Study Treatment Design

Dose Level Group	Treatments	Treatment Group	No. of Subjects	Dose Level
1	Coronavirus-Like Particle COVID- 19 Vaccine	1	20	3.75 µg
	Coronavirus-Like Particle COVID- 19 Vaccine + CpG 1018	2	20	3.75 µg
	Coronavirus-Like Particle COVID- 19 Vaccine + AS03	3	20	3.75 µg
2	Coronavirus-Like Particle COVID- 19 Vaccine	4	20	7.5 µg
	Coronavirus-Like Particle COVID- 19 Vaccine + CpG 1018	5	20	7.5 µg
	Coronavirus-Like Particle COVID- 19 Vaccine + AS03	6	20	7.5 µg
3	Coronavirus-Like Particle COVID- 19 Vaccine	7	20	15 µg
	Coronavirus-Like Particle COVID- 19 Vaccine + CpG 1018	8	20	15 µg
	Coronavirus-Like Particle COVID- 19 Vaccine + AS03	9	20	15 µg

All subjects will receive Intra Muscular (IM) two doses of the same treatment group (dose level and adjuvant) of the vaccine 21 days apart, on Day 0 and Day 21 (one injection on each of these days) and the data collected will be used to determine if one or two doses is/are required.

For each dose level group, the three-day safety data after immunization of the first 10 % of subjects enrolled (6 subjects) in the group will be collected and reviewed by the IDMC, prior to

Medicago/Cytel Confidential: This is a controlled document and shall not be changed, saved, copied or reproduced by any unauthorized person.

permitting vaccination of the next 30 % of subjects enrolled (18 subjects) in the dose level groupand escalating to vaccination of subjects at the next higher dose level (until reaching the highest dose level). The three-day safety data after vaccination of these 18 subjects in the group will be collected and reviewed by the IDMC, prior to permitting vaccination of the remaining subjects inthe dose level group. The two planned three-day safety data reviews by the IDMC for each dose level will occur after each vaccination in the study. The first 10 % of subjects in each dose level group may be vaccinated in the same day at the clinical sites. After IDMC recommendation to proceed is received, no more than 18 subjects (from any dose level group) will be dosed at a clinical site per day to respect social distancing.

Subjects will be screened up to seven days in advance of the first vaccine administration and will demonstrate a satisfactory baseline medical assessment by history, general physical examination, haematological, biochemical, and serological analysis. In addition, a test for the absence of SARS-CoV-2 antibodies will be performed at screening and prior to vaccination on Day 0, as well as onDay 42, Day 201 and Day 386. On Days 0 and 21, vaccine administration will occur. Telephonecontacts will be made one day and eight days after each vaccine administration as well as monthlyafter Day 42 until the final study visit (Day 386), specifically for review of the subject's safety and concomitant medication data. Visits to the Investigator site will occur three days after each vaccination (Day 3 and Day 24) for key safety assessments and 21 days after each vaccination (Day 21 and Day 42) for key safety and immunogenicity assessments. Subjects will return to theInvestigator site on Day 201 and Day 386 for safety follow-ups and immunogenicity assessments(six-month and 12-month follow-ups).

Study Objectives

The primary objectives of this study are:

To assess the safety and tolerability of the Coronavirus-Like Particle COVID-19 Vaccine,as determined by the occurrence(s) of:

Immediate AEs within 30 minutes after each vaccination;

Solicited local and systemic AEs up to 7 days after each vaccination;

Unsolicited AEs, Serious adverse events (SAEs), AEs leading to withdrawal, AESIs,and deaths up to 21 days after each vaccination;

Subjects with normal and abnormal urine, haematological, and biochemical values;

To assess the immunogenicity of the Coronavirus-Like Particle COVID-19 Vaccine up to21 days after each vaccination, as determined by:

Medicago/Cytel Confidential: This is a controlled document and shall not be changed, saved, copied or reproduced by any unauthorized person.

Neutralizing antibody titers induced by the vaccine against the SARS-CoV-2 virus;

Interferon (IFN)-γ enzyme-linked immuno spot assay (ELISpot) response induced by the vaccine against the SARS-CoV-2 virus to assess T helper 1 (Th1) type response;

Interleukin (IL)-4 ELISpot response induced by the vaccine against the SARS-CoV-2 virus to assess T helper 2 (Th2) type response.

The secondary objectives of this study are:

To assess the immunogenicity of the Coronavirus-Like Particle COVID-19 Vaccine up to21 days after each vaccination, as determined by the:

IgG and/or IgM (enzyme-linked immunosorbent assay [ELISA]) titers induced bythe vaccine against the SARS-CoV-2 virus.

To assess the immunogenicity of the Coronavirus-Like Particle COVID-19 Vaccine up to201 and 386 days after the first vaccination, as determined by the:

Neutralizing antibody titers induced by the vaccine against the SARS-CoV-2 virus;

IFN-γ ELISpot response induced by the vaccine against the SARS-CoV-2 virus;

IL-4 ELISpot response induced by the vaccine against the SARS-CoV-2 virus;

IgM and/or IgG (ELISA) titers induced by the vaccine against the SARS-CoV-2virus;

To assess the safety and tolerability of the Coronavirus-Like Particle COVID-19 Vaccine,as determined by the occurrence(s) of:

SAEs, AEs leading to withdrawal, AESIs, and deaths from 22 days after the lastvaccination up to Day 201.

Occurrences of SAEs, AEs leading to withdrawal, AESIs (including VED), anddeaths from Day 202 up to the end of the study (Day 386)

The exploratory objectives of this study are:

To assess the specific Cell-mediated immune (CMI) response induced by the Coronavirus-Like Particle COVID-19 Vaccine against the SARS-CoV-2 virus up to 21 days after eachvaccination and up to 201 and 386 days after the first vaccination, as measured by the percentage of CD4+ T cells expressing functional markers;

Medicago/Cytel Confidential: This is a controlled document and shall not be changed, saved, copied or reproduced by any unauthorized person.

To measure the levels of serum IgE antibodies directed to plant-specific glycans prior to vaccination (Day 0), up to 21 days after the second vaccination (Day 42) and up to 201 and 386 days after the first vaccination (Day 201 and Day 386);

If deemed necessary, to further characterize the immune response and the safety profile ofthe Coronavirus-Like Particle COVID-19 Vaccine.

To measure the occurrence(s) of laboratory-confirmed (virologic or serologic methods) SARS-CoV-2 infection (with or without symptoms) up to the end of the study.

Sample Size Justification

As per protocol, the sample size of approximately 180 subjects with 20 subjects in each treatment group will make it possible to detect gross differences in rates of adverse events and to perform the initial evaluation of the vaccine immunogenicity. The sample size is not large enough to detectall types, including less frequent or rare, adverse reactions. The objective of this study is to quantify the type, percentage, intensity, duration, and relationship of short-term post-vaccinationevents to determine if they differ clinically among the treatment groups.

Subject Selection

Inclusion Criteria

Subjects must meet all of the following inclusion criteria at the Screening (Visit 1) and/or Vaccination (Visit 2) visits to be eligible for participation in this study; no protocol waivers are allowed. All Investigator assessment-based judgements must be carefully and fully documented in the source documents:

Subjects must have read, understood, and signed the informed consent form (ICF) prior toparticipating in the study; subjects must also complete study-related procedures and communicate with the study staff at visits and by phone during the study;

At the Screening visit (Visit 1), male and female subjects must be 18 to 55 (has not yet had his/her 56th birthday) years of age, inclusive;

At Screening (Visit 1) and Vaccination (Visit 2), subject must have a body mass index (BMI) of ≥ 18.5 and < 25 kg/m^2;

Subjects are considered by the Investigator to be reliable and likely to cooperate with theassessment procedures and be available for the duration of the study;

Medicago/Cytel Confidential: This is a controlled document and shall not be changed, saved, copied or reproduced by any unauthorized person.

Subjects must be healthy (no clinically significant health concerns) as determined by medical history, physical examination, vital signs, and clinical laboratory tests. Investigator discretion will be permitted with this inclusion criterion;

Female subjects of childbearing potential must have a negative serum pregnancy test resultat Screening (Visit 1) and a negative urine pregnancy test result at Vaccination (Visit 2 and Visit 4). Non-childbearing females are defined as:

Surgically-sterile (defined as bilateral tubal ligation, hysterectomy or bilateral oophorectomy performed more than one month prior to the first study vaccination); or

Post-menopausal (absence of menses for 12 consecutive months and age consistentwith natural cessation of ovulation);

Female subjects of childbearing potential must use an effective method of contraception for one month prior to vaccination (Visit 2) and agree to continue employing highly effective birth control measures for at least one month after the last administration of the investigational product (or in the case of early termination, she must not plan to become pregnant for at least one month after her last study vaccination). The following relationshipor methods of contraception are considered to be highly effective:

Combined (estrogen and progestogen containing) hormonal contraception associated with inhibition of ovulation:

Oral;
Intravaginal;
Transdermal;
Progestogen-only hormonal contraception associated with inhibition ofovulation:

Oral;
Injectable;
Implantable;
Intra-uterine device with or without hormonal release;

Vasectomised partner, provided that this partner is the sole sexual partner of thestudy participant and that the vasectomised partner has received a medical assessment of the surgical success;

Medicago/Cytel Confidential: This is a controlled document and shall not be changed, saved, copied or reproduced by any unauthorized person.

Credible self-reported history of heterosexual vaginal intercourse abstinence prior to and for at least one month after the last administration of the investigational product;

Female partner.

Exclusion Criteria

Subjects who meet any of the following criteria at the Screening (Visit 1) and/or Vaccination (Visit 2) visits will not be eligible for participation in this study; no protocol waivers are allowed.All Investigator assessment-based judgements must be thoroughly documented in the source documents:

Clinically significant acute or chronic pulmonary (including but not limited to chronic obstructive pulmonary disease or asthma), cardiovascular (including but not limited to arterial hypertension, coronary artery disease, or congestive heart failure), renal, metabolic(including but not limited to type 2 diabetes), or other somatic (medical) or neuropsychiatric illness within 3 months prior to Screening (Visit 1), excessive alcohol use or drug abuse, as determined by medical history, physical examination, vital signs, and clinical laboratory tests. Investigator discretion is permitted with this exclusion criterion and must be carefully and fully documented in the source documents;

Any unexplained clinical syndrome (including, but not limited to, chronic fatigue syndrome, Raynaud's syndrome, unexplained pain syndromes such as fibromyalgia, etc.);

Acute disease defined as presence of any moderate or severe acute illness with or without a fever within 48 hours prior to Vaccination (Visit 2);

Prior exposure to SARS-CoV-2 as determined by detection of IgM or IgG antibodies against SARS-CoV-2 at Screening (Visit 1) and Vaccination (Visit 2);

Any confirmed or suspected current immunosuppressive condition or immunodeficiency,including cancer, human immunodeficiency virus (HIV), hepatitis B or C infection(subjects with a history of cured hepatitis B or C infection without any signs of immunodeficiency at present time are allowed). Investigator discretion is permitted with this exclusion criterion;

Current autoimmune disease (such as rheumatoid arthritis, systemic lupus erythematosusor multiple sclerosis). Investigator discretion is permitted with this exclusion criterion, andsubjects may be eligible to participate with appropriate written justification in the source

Medicago/Cytel Confidential: This is a controlled document and shall not be changed, saved, copied or reproduced by any unauthorized person.

document (i.e. subjects with a history of autoimmune disease who are disease-free withouttreatment for three years or more, or on stable thyroid replacement therapy, mild psoriasis[i.e. a small number of minor plaques requiring no systemic treatment], etc.);

Administration of any medication or treatment that may alter the vaccine immune responses, such as:

Systemic glucocorticoids within one month prior to Vaccination (Visit 2). Inhaled, nasal, dermal, intraarticular, ophthalmic and other topical glucocorticoids are permitted;

Cytotoxic, antineoplastic, or immunosuppressant drugs - within 36 months prior to Vaccination (Visit 2);

Any immunoglobulin preparations or blood products, blood transfusion – within 6 months prior to Vaccination (Visit 2);

Administration of any vaccine within 30 days prior to Vaccination (Visit 2); planned administration of any vaccine during the study (up to blood sampling on Day 42 of the study). Immunization on an emergency basis during the study will be evaluated on case- by-case basis by the Investigator;

Administration of any other SARS-CoV-2 / COVID-19, or other experimental coronavirusvaccine at any time prior to or during the study;

Known current or previous laboratory-confirmed SARS-CoV-1 or SARS-CoV-2 / COVID-19 infection as documented by a positive PCR test or positive serological test;

Subjects at high risk of contracting SARS-CoV-2/COVID-19 infection, including but notlimited to the individuals with known close contact of anyone with laboratory-confirmedSARS-CoV-2 / COVID-19 infection within 2 weeks prior to vaccine administration, thosewho traveled outside Canada for any duration within 30 days before the study vaccination,healthcare workers in acute care hospitals, rehabilitation hospitals, mental health hospitals,long term care facilities, emergency departments, and others who through their work mustcome into close face-to-face contact with their clients or patients (including, but not limited to, physiotherapists, dentists, hair dressers/barbers, etc.);

Use of any investigational or non-registered product within 30 days or 5 half-lives, whichever is longer, prior to Vaccination (Visit 2) or planned use during the study period;

Medicago/Cytel Confidential: This is a controlled document and shall not be changed, saved, copied or reproduced by any unauthorized person.

Have a rash, dermatological condition, tattoos, muscle mass, or any other abnormalities atinjection site that may interfere with injection site reaction rating. Investigator discretion will be permitted with this exclusion criterion;

Use of any prescription medication on a regular basis for more than 30 continuous days within the last 3 months, with the following exceptions:

Medications listed among the methods of contraception in inclusion criterion 7;

Subjects on stable thyroid replacement therapy, as noted in exclusion criterion 6.

Use of any prescription antiviral drugs with the intention of COVID-19 prophylaxis, including those that are thought to be effective for prevention of COVID-19 but have notbeen licensed for this indication, within one month prior to Vaccination (Visit 2);

Use of any prescription antiviral drugs with the intention of COVID-19 prophylaxis, including those that are thought to be effective for prevention of COVID-19 but have notbeen licensed for this indication, within one month prior to Vaccination (Visit 2);

Use of prophylactic medications (e.g. antihistamines [H1 receptor antagonists],nonsteroidal anti-inflammatory drugs [NSAIDs], systemic and topical glucocorticoids, non-opioid and opioid analgesics) within 24 hours prior to the Vaccination (Visit 2) to prevent or pre-empt symptoms due to vaccination;

History of allergy to any of the constituents of the Coronavirus-Like Particle COVID-19 Vaccine, vaccine adjuvants, or tobacco;

History of anaphylactic allergic reactions to plants or plant components (including fruits and nuts);

Currently smoke more than 10 cigarettes per month or any use of vaping products;

Subjects with a history of Guillain-Barré Syndrome;

Any female subject who has a positive or doubtful pregnancy test result prior to vaccination or who is lactating;

Subjects identified as an Investigator or employee of the Investigator or clinical site withdirect involvement in the proposed study, or identified as an immediate family member (i.e. parent, spouse, natural or adopted child) of the Investigator or employee with direct involvement in the proposed study, or any employees of Medicago.

Medicago/Cytel Confidential: This is a controlled document and shall not be changed, saved, copied or reproduced by any unauthorized person.

Randomization Methodology

Randomization will be used to minimize bias in the assignment of subjects to treatment groups, to increase the likelihood that known and unknown subject attributes (e.g. demographic and baseline characteristics) are evenly balanced across treatment groups, and to enhance the validityof statistical comparisons across treatment groups.

The subjects will be randomized in a 1:1:1:1:1:1:1:1:1 ratio into one of the nine treatment groups presented in Table 1. Randomization will be done using a permuted block randomization schedule prepared under the supervision of Medicago. More particularly, subjects will be equally randomized between treatments pertaining to active/open dose levels. Opening/closure of a dose level will bedone according to IDMC recommendation. Randomization will only occur within treatment armsbelonging to an open/active dose level. No stratification factors will be used.

Unblinding

This is a partially-blinded study. The subjects, the Investigators, and all personnel involved in the clinical conduct of the study (except the staff involved in the preparation and administration of the study vaccine, the quality assurance auditor, and quality control reviewers), Medicago medicaland clinical staff involved in safety evaluations (e.g. causality assessments), and all personnel involved in sample analysis at the central and testing laboratories will not have access to treatmentallocation (i.e. randomization codes) for the entire duration of the study. Partially-blinded trial design will also allow all study staff to remain unblinded to dose level only in order to manage the staggered ascending dose escalation and IDMC coordination.

Since there will be differences in the Coronavirus-Like Particle COVID-19 Vaccine and adjuvant preparations (e.g. possibly physical appearance), the site staff involved in the preparation and administration of the treatments will not be involved in any activity that could introduce a bias, such as the evaluations of reactogenicity or AEs experienced by the subjects following vaccination.

The IDMC and the independent statistician involved in the preparation of the safety data summaryfor the IDMC reviews will have access to or be aware of treatment allocation (i.e. be “unblinded”)during the conduct of the study. Further details are provided in a dedicated IDMC charter.

In addition, selected Medicago and third-party statistical team (Cytel) personnel will have accessto treatment allocation and unblinded results (i.e. will be “unblinded”) during the conduct of the study for the following purposes:

Day 21 data transfer

Day 42 data analysis

Medicago/Cytel Confidential: This is a controlled document and shall not be changed, saved, copied or reproduced by any unauthorized person.

Day 201 data analysis

The process of unblinding and the selected individuals who have access to unblinded information during the conduct of the study will be documented outside of this document, in the Unblinding Management Plan.

Full study unblinding will take place after database lock of the Day 386 analysis.

Stopping Rules

The Investigator (in consultation with Medicago [or its designee]), Medicago, or Regulatory Authorities may interrupt or terminate the study for any reason.

Safety monitoring of safety signals will be performed throughout the study. Stopping rules or conditions for stopping this clinical trial would occur if there was clear evidence of harm or harmful effects such as SAEs related to the treatment (study vaccine). A SAE which was thoughtto be unrelated to the study vaccine would not warrant stopping the trial.

The following event(s) may result in a halt to the study, for further review and assessment of the event(s):

Any death occurring during the study;
Any vaccine-related SAE during the study;
Any life-threatening (Grade 4) vaccine-related AE during the study;
If 10 % or more of subjects in a single treatment group, experience the same orsimilar listed event(s) that cannot be clearly attributed to another cause:
a severe (Grade 3 or higher) vaccine-related AE during the study;
a severe (Grade 3 or higher) vaccine-related vital sign(s) abnormality;
a severe (Grade 3 or higher) vaccine-related clinical laboratoryabnormality.
In the case that a pre-defined safety signal is met in any treatment group, subsequent dosing willresult in at least a transient halt in the study to permit a complete evaluation of the reported event(s) and to consult an IDMC. A decision as to whether the study can progress as planned mustbe made and documented in the event of any safety signal. In addition, the IDMC may recommendinterrupting or halt the study for safety reasons at any time (refer to IDMC charter for further details). If a stopping rule has been met once all subjects have been vaccinated in the study, the IDMC will be notified by a Note To File (NTF) for their information purposes.

Study Procedures

The schedule of assessments, as outlined in the study protocol v2.0, is provided in Table 2: Schedule of Assessments.

Medicago/Cytel Confidential: This is a controlled document and shall not be changed, saved, copied or reproduced by any unauthorized person.

Table 2: Schedule of Assessments

i

**Visit Type**		**Screening**	**Vaccination**	**Post-vaccination Visit/Contacts**		**Vaccination**	**Post-vaccination Visits/Contacts**						
**Study Day**	**Day -7 to 0**	**Day 0**	**Day 1 (+ 1)**	**Day 3 (- 1)**	**Day 8 (± 1)**	**Day 21 (± 2)[7]**	**Day 22 (+ 1)**	**Day 24 (- 1)**	**Day 29 (± 1)**	**Day 42 (± 2)**	**Monthly Calls (± 14)[8]**	**Day 201 (± 14)**	**Day 386 (± 14)**
**Visit Number**	**1**	**2**	**Phone**	**3**	**Phone**	**4**	**Phone**	**5**	**Phone**	**6**	**Phone**	**7**	**8**
Informed consent	X												
Test for SARS-CoV-2	X	X[10,11]								X		X	X
Demographics	X												
Medical history/prior medication	X	X[1]											
Inclusion/exclusion criteria	X	X[1]											
History/symptom-directed physical examination	X[2]												
Vital Signs	X	X[3]		X		X[3]		X		X		X	X
Height, weight, and BMI	X	X											
Urinalysis	X			X		X[10]		X					
Pregnancy test (Urine)		X[10]				X[10]				X			
Pregnancy test[4] (Serum)	X												
Serology tests for HIV, Hepatitis B, and Hepatitis C	X												
Blood chemistry And Haematology	X			X		X[10]		X					
Immunogenicity -Serology (Nab assay, ELISA, and anti-plant		X[10]				X[9, 10]				X		X	X

Medicago/Cytel Confidential: This is a controlled document and shall not be changed, saved, copied or reproduced by any unauthorized person.

glycans IgE antibodies)[9]													
Immunogenicity – CMI Response (PBMC)		X[10]				X[10]				X		X	X
Randomization		X											
Vaccine admin		X				X							
Immediate surveillance (30 minutes)		X				X							
Provide and collect diary and/or memory aid instructions		X				X				X		X	X
Oral digital thermometer and instructions on AEs[5]		X				X							
Collection of solicited local/ systemic AEs		X	X	X	X	X	X	X	X				
Concomitant medications		At any time during the study period											
AEs, SAEs, AESIs, pregnancy reports and laboratory-confirmed reports of SARS-CoV-2 infection (with or without symptoms)[6]		At any time during the study period											
Termination record													X

[1] Record any changes in medical history and medications and confirmation that the subject continues to meet all inclusion and no exclusioncriteria since screening.
[2] History/symptom-directed physical examinations will not be routinely performed at any other visits, unless new complaints or concerns areraised by either the study subject or study staff, and if deemed to be necessary by the Investigator.
[3] Record prior to study vaccine, after the observation period and as deemed necessary.
[4] In all females of childbearing potential; at screening it will be tested in serum and at Days 0, 21, and 42 it will be tested in urine.

Medicago/Cytel Confidential: This is a controlled document and shall not be changed, saved, copied or reproduced by any unauthorized person.

[5] After vaccination, subjects will be instructed on the diary and memory aid provided for their use for recording AEs and concomitant medication use.
[6] AEs will be collected up to Day 42; SAEs, AEs leading to withdrawal, and AESIs will be collected through to the end of the study. Investigators should report SAEs, AESIs, and pregnancy reports to Sponsor within 24 hours of the Investigator learning of the event. Specific contacts for the collection of information regarding all of these events will occur on Day 201 and Day 386 for SAEs, AEs leading to withdrawal, and AESI. Also, all laboratory-confirmed reports of SARS-CoV-2 infection (with or without symptoms) will be collected through to the end of the study.
[7] All efforts will be done to have subjects returning on planned date for Day 21 activities. If for any reason the visit is done before or after this planned date, subsequent visits/procedures (Days 22, 24, 29, 42, 201, and 386 and the monthly calls) will be adjusted accordingly.
[8] Subjects should be reached once a month with no more than 45 days between phone contacts (use Day 42 date as starting reference or last day of contact with the clinic site or Investigator if a second vaccination did not occur).
[9] On Day 21, the immunogenicity – serology blood sample will be collected for the Nab assay and ELISA only. Anti-plant glycan IgE antibodies will only be measured on Days 0 (prior to vaccination), 42, 201, and 386.
[10] Blood and urine samples are to be collected prior to vaccination on the respective day.
[11] Vaccination on Day 0 can proceed prior to receiving the results of the confirmatory SARS-CoV-2 test on Day 0.

Safety and Immunogenicity Endpoints and Variable Definition

Safety endpoints

The primary safety endpoints are:

Occurrence, intensity and relationship of immediate Adverse Events (AEs) (30 minutesafter each vaccination administration);

Occurrence of solicited local and systemic adverse AEs (for seven days following eachvaccine administration);

Occurrence of unsolicited AEs for 21 days following each vaccine administration;

Occurrences of serious adverse events (SAEs), AEs leading to withdrawal, AESIs(including VED), and deaths for 21 days following each vaccine administration;

Occurrence of abnormal clinically significant urine, haematological and biochemicalvalues prior to and 3 days following each vaccination.

The secondary safety endpoints are:

Occurrences of SAEs, AEs leading to withdrawal, AESIs (including VED), and deathsfrom 22 days after the last vaccination up to the end of the study Day 201;
Occurrences of SAEs, AEs leading to withdrawal, AESIs (including VED), and deathsfrom Day 202 up to the end of the study (Day 386).

The exploratory safety endpoints are:

If deemed necessary, further characterization of the safety profile of the Coronavirus-Like Particle COVID-19 Vaccine;

Occurrence(s) of laboratory-confirmed (virologic or serologic methods) SARS-CoV-2infection (with or without symptoms) up to the end of the study.

Safety variables definition are provided in Table 3.

Medicago/Cytel Confidential: This is a controlled document and shall not be changed, saved, copied or reproduced by any unauthorized person.

Table 3: Safety Variable Definitions

**Adverse event (AE)**	An AE or adverse experience is defined as any untoward medical occurrence in a subject or clinical investigation subject who was administered a pharmaceutical product, with or without a causal relationship with the treatment. An AE can be any unfavorable and unintended sign, symptom, or disease temporally associated with the use of a medicinal product, whether or not related to a medicinal product.<br><br>The intensity of all AEs will be graded as mild (1), moderate (2) severe (3), or potentially life threatening (4), according to the FDA Guidance for Industry: Toxicity Grading Scale for Healthy Adult and Adolescent Volunteers Enrolled in Preventive Vaccine Clinical Trials [FDA 2007] for the events covered in the guidance and the following definitions for all other events:<br><br>Mild (Grade 1): The AE is easily tolerated and does not interfere with usual activity;<br>Moderate (Grade 2): The AE interferes with daily activity, but the subject is still able to function;<br>Severe (Grade 3): The AE is incapacitating, and the subject is unable to work or complete usual activity; Potentially life- The AE is likely to be life-threatening if notthreatening (Grade 4): treated in a timely manner.<br><br>All unsolicited AEs occurring within 21 days after each vaccination must be reported in the "Adverse Event" screen in the subject's eCRF, irrespective of intensity or whether or not they are considered to be vaccination-related. Thereafter, from 22 days after last vaccination through to Day 386, only SAEs, AEs leading to withdrawal, AESIs will be monitored and reported in the eCRF.<br><br>For the purpose of the analysis, only AE which began on or after the date and time of the first vaccination (Day 0) will be considered.
**Related event**	AE causal relationship with the study vaccine will be assessed by the Investigator (definitely not related, probably not related, possibly related, probably related or definitely related); see Section 13.1.9 in protocol for a definition of these causal relationships.

Medicago/Cytel Confidential: This is a controlled document and shall not be changed, saved, copied or reproduced by any unauthorized person.

	A related event is an event with a causal relationship with vaccination of "definitely related", "probably related", "possibly related". Missing relationship will not be imputed.
**Serious Adverse Event (SAE)**	An SAE is any untoward medical occurrence (whether or not considered to be related to the study vaccine) that, at any dose:<br><br>Results in death;<br><br>Is life-threatening (at the time of the event);<br><br>Note: the term "life-threatening" in the definition of an SAE refers toan event that put the subject at risk of death at the time of the event; it does not refer to an event that hypothetically might have caused death if it were more severe;<br><br>Requires inpatient hospitalization (≥ 24 hours) or prolongation of existing hospitalization (elective hospitalizations/procedures for pre-existing conditions that have not worsened are excluded);<br><br>Results in persistent or significant disability/incapacity;<br><br>Is a congenital abnormality/birth defect;<br><br>Is another medically important event<br>All SAEs (solicited or unsolicited) occurring during the study must be reported in the "Adverse Event" screen in the subject's eCRF (Adverse Events page), irrespective of intensity or whether or not they are considered to be vaccination-related.<br><br>SAEs will be identified by selecting events with "Yes" to the question "Was the adverse event serious?"
**Solicited adverse event (AE)**	A solicited AE is an "expected" adverse event observed and reported under the conditions (nature and onset) pre-listed in the protocol and CRF.<br>Solicited reactions can either be:<br>solicited injection site AE (local AE) or<br>solicited systemic AE.

	Subjects will be monitored for solicited AE from the time of vaccination (Day 0) through Day 7 after each vaccine administration. If any of the solicited AEs persist beyond Day 7 after each vaccination (when applicable), these will also be recorded as unsolicited AEs. In this case, the AE start will be set as eight days post-vaccination.<br>The intensity of the solicited AEs will be graded as: mild (1), moderate (2), severe (3) or potentially life threatening (4) as described in Table 5 of protocol.<br>The presence of a solicited AE will be derived from the maximum overall intensity observed during the period of interest (from Day 0 to Day 7):<br>if the maximum intensity is “None”: No presence;<br>if maximum intensity is Grade 1, Grade 2, Grade 3 or 4: Presence;<br>if maximum intensity is Missing or Unknown: Missing presence.
**Solicited injection site AE**	A solicited injection site AE is one of the following Solicited AE:<br>Erythema (redness);<br>Swelling;<br>Pain.<br><br>The causal relationship with the study vaccine of the solicited local AEs will be assessed by the Investigator (definitely not related, probably not related, possibly related, probably related or definitely related).
**Solicited systemic AE**	A solicited systemic AE is one of the following solicited AE:<br>Fever;<br>Headache;<br>Fatigue;<br>Muscle aches;<br>Joint aches;<br>Chills;<br>Feeling of general discomfort or uneasiness (malaise)<br>Swelling in the axilla;<br>Swelling in the neck;<br>Swelling in the groin;<br>Swelling in the chest wall.

Medicago/Cytel Confidential: This is a controlled document and shall not be changed, saved, copied or reproduced by any unauthorized person.

	The causal relationship with the study vaccine of the solicited systemic AEs will be assessed by the Investigator (definitely not related, probably not related, possibly related, probably related or definitely related).
**Unsolicited AE**	An unsolicited AE is an observed AE that does not fulfill the conditions prelisted in the CRF in terms of diagnosis and/or onset window post-vaccination. For example, if headache between D0 and D7 is a solicited AE (ie, prelisted in the protocol and CRF), then a headache starting on D7 is a solicited reaction, whereas headache starting on D8 post-vaccination is an unsolicited AE. Unsolicited AEs will be collected on the "Adverse Event" CRF page (**note**: serious solicited events reported on the "Adverse Event" CRF page should not be considered as unsolicited events)
**Immediate Event**	Immediate events (solicited or unsolicited) are recorded to capture AEs that occur within the first 30 minutes after vaccination. Immediate solicited events will be recorded on the Diary (FA) CRF page and will be identified with the pre-specified timepoint "Day 0 - 30 minute Post Vaccination". Immediate unsolicited events will be recorded on the Adverse event CRF page and will be identified using the "Did this AE start within the first 30 minutes after vaccination?" CRF flag.
**Adverse Event of Special Interest (AESI) for the Coronavirus-Like Particle COVID-19 Vaccine**	All reported events will be monitored for the possible safety signal of Vaccine Enhanced Disease (VED) and for hypersensitivity reactions after exposure to the Coronavirus-Like Particle COVID-19 Vaccine.<br><br>The following categories will be considered as AESI:<br><br>Vaccine-Enhanced Disease (VED);<br>Hypersensitivity Reactions<br><br>VED will be defined programmatically for any subjects with a laboratory confirmed SARS-CoV-2 infection at any time during the study and with AEs within the following terms:<br><br>System Organ Classes (SOC):<br>"Immune system disorders"<br>High Level Group Term (HLGT):<br>"Lower respiratory tract disorders (excluding obstruction andinfection)",<br>"Cardiac disorders, signs and symptoms not elsewhere classified(NEC)",<br>"Vascular disorders",

Medicago/Cytel Confidential: This is a controlled document and shall not be changed, saved, copied or reproduced by any unauthorized person.

	"Heart failures",<br>"Arteriosclerosis, stenosis, vascular insufficiency and necrosis",<br>"Cardiac arrhythmias",<br>"Myocardial disorders",<br>"Vascular hemorrhagic disorders".<br>High Level Terms (HLTs):<br>"Renal failure and impairment"<br>Preferred term (PT):<br>"Pericarditis"<br>"Coagulopathy"<br>"Deep vein thrombosis"<br>"Pulmonary embolism"<br>"Cerebrovascular accident"<br>"Peripheral ischemia"<br>"Liver injury"<br>"Guillain-Barre syndrome"<br>"Anosmia"<br>"Ageusia"<br>"Encephalitis"<br>"Chilblains"<br>"Vasculitis"<br>"Erythema multiforme"<br><br>**and**<br>requiring inpatient hospitalization (≥ 24 hours) i.e with seriousness criteria = "Results in initial or prolonged hospitalization".<br><br>Subjects with laboratory confirmed SARS-CoV-2 infection will be defined as follows:<br>Any subjects with a confirmed SARS-CoV-2 infection as recorded on the "Clinical Visit Assessments COVID-19", "phone contact", or"COVID-19 Assessment or Experience" CRF pages<br>Any subjects with a presence of SARS-CoV-2 antibodies identified after first vaccination as collected on the "Test for SARS-CoV-2 antibodies" CRF page<br>Hypersensitivity Reactions will be defined as any AEs within Hypersensitivity Standard MedDRA Query (SMQ).

Medicago/Cytel Confidential: This is a controlled document and shall not be changed, saved, copied or reproduced by any unauthorized person.

	AESI will be collected from vaccination on Day 0 to Day 386 and reported on the "Adverse Events" CRF.
**AESI for the Adjuvants**	AESI for adjuvants are potential immune-mediated diseases (pIMDs) which are a subset of AEs that include autoimmune diseases and other inflammatory and/or neurologic disorders of interest which may or may not have an autoimmune aetiology. Adverse events that need to be recorded and reported as pIMDs include those listed in Section 19.5 of protocol.<br>AESI will be collected from vaccination on Day 0 to Day 386 and reported on the "Adverse Events" CRF.<br>Identification of AESI will be done programmatically as any AE with a Preferred Term falling under selected SOC/standardized MedDRA classification provided in Appendix 5 - List of Potential Immune-Mediated Diseases.

Immunogenicity endpoints

The immunogenicity primary endpoints are:

Neutralizing antibody (Nab assay) as reported by Nexelis laboratory response induced by the vaccine against the SARS-CoV-2 virus 21 days after each vaccination;
Specific Th1 CMI response induced by the vaccine against the SARS-CoV-2 virus21 days after each vaccination, as measured by IFN-γ ELISpot;
Specific Th2 CMI response induced by the vaccine against the SARS-CoV-2 virus21 days after each vaccination, as measured by IL-4 ELISpot.

The immunogenicity secondary endpoints are:

Specific antibody response induced by the vaccine against the SARS-CoV-2 virus21 days after each vaccination and on Day 201 and Day 386, as measured by totalIgM and/or IgG levels;
Neutralizing antibody (Nab assay) response induced by the treatment groups against the SARS-CoV-2 virus on Day 201 and Day 386;
Specific Th1 CMI response induced by the vaccine against the SARS-CoV-2 viruson Day 201 and Day 386, as measured by IFN-γ ELISpot;

Medicago/Cytel Confidential: This is a controlled document and shall not be changed, saved, copied or reproduced by any unauthorized person.

Specific Th2 CMI response induced by the vaccine against the SARS-CoV-2 viruson Day 201 and Day 386, as measured by IL-4 ELISpot.

The immunogenicity exploratory endpoints are:

Specific CMI response induced by the vaccine against the SARS-CoV-2 virus 21 days after each vaccination and on Day 201 and Day 386, as measured by the number of CD4+ T cells expressing functional markers;
Specific antibody response induced by the vaccine against plant glycans 21 days after the second vaccination and on Day 201 and Day 386, as measured by serumIgE levels directed against CDD MUXF3 using bromelain glycoprotein;
If deemed necessary, further characterization of the immune response of the Coronavirus-Like Particle COVID-19 Vaccine;

Medicago/Cytel Confidential: This is a controlled document and shall not be changed, saved, copied or reproduced by any unauthorized person.

OVERVIEW OF PLANNED ANALYSISThe following
analysis are planned as per protocol:

For each dose level group, the three-day safety data after immunization of the first 10 % of subjects enrolled (6 subjects) in the group will be collected and reviewed by the IDMC,prior to permitting immunization of the next 30 % of subjects enrolled (18 subjects) in thedose level group and escalating to immunization of subjects at the next higher dose level (until reaching the highest dose level). The three-day safety data after immunization of these 18 subjects in the group will be collected and reviewed by the IDMC, prior to permitting immunization of the remaining subjects in the dose level group. The two planned three-day safety data reviews by the IDMC for each dose level will occur after each vaccination in the study. Further details are provided in a dedicated IDMC charter and IDMC SAP.

After the last subject has completed Day 21 assessments, the available **unblinded** safety and immunogenicity data will be transferred via secure platform to the authorized unblinded member(s) of the sponsor to utilize for the finalization of study design of the subsequent Phase 2 study. No data cutoff will be applied. Analyses using the selected Day21 data will be performed by unblinded member(s) of the sponsor and will be described in a separate document. The data transfer will be confidential and strictly limited to the authorized unblinded staff members. The list of data points to be transferred are providedin Appendix 1 – Day 21 data transfer.

Day 42 analysis will be performed after the last subject has completed Day 42 assessments.Data available up to Day 42 visit will be cleaned and included in the analyses. The unblinded results of the data analyses will be confidential and strictly limited to the authorized unblinded staff members. The list of outputs to be prepared for this analysis are provided in Appendix 2 – Day 42 analysis.

An interim analysis will be performed after the last subject has completed Day 201 assessments. Data available at that time will be cleaned and included in the analyses. Theresults of the interim analysis will be used to prepare the clinical study report (up to Day 201) for the purpose of reporting to regulatory agencies the safety and immune response profile of the Coronavirus-Like Particle COVID-19 Vaccine up to six months after the second vaccination. The list of outputs to be prepared for the Day 201 CSR are provided in Appendix 3 – Day 201 analysis.

Medicago/Cytel Confidential: This is a controlled document and shall not be changed, saved, copied or reproduced by any unauthorized person.

Final analysis will be performed after the last subject has completed End of Study assessment (Day 386). For this analysis, the database will be cleaned, locked and full unblinding will be performed. The results from the analysis of data up to Day 386 will bepresented in an addendum to the clinical study report. The list of outputs to be prepared for the final CSR are provided in Appendix 4 – Day 386 analysis.

Medicago/Cytel Confidential: This is a controlled document and shall not be changed, saved, copied or reproduced by any unauthorized person.

ANALYSIS SETS

Population Definitions

The following analysis set will be evaluated and used for presentation and analysis of the data:

<u>All Analysis Set:</u>
The All analysis set is defined as all subjects who signed an informed consent.

<u>Safety Analysis Set (SAS)</u>
The safety analysis set (SAS) is defined as all subjects who received at least one injection of eitherthe Coronavirus-Like Particle COVID-19 Vaccine with or without an adjuvant.

Demographic and baseline characteristics performed on the SAS analysis set will be done according to the actual vaccination received at the first vaccination. All safety analyses will be performed using the SAS and according to actual vaccination received. More particularly, subjectswill have their safety analyzed after each vaccination (D0-D21 period) according to the last vaccine they actually received, and after any vaccination (D0-D21 after last vaccination period/D0-EOS period) according to the actual vaccine received at the first dose.

Actual treatment received will be derived using the medication/Box ID list provided by the IRT and the lot numbers collected on the eCRF. If actual treatment cannot be unequivocally confirmed,assigned treatment will be used.

<u>Intent-To-Treat Set (ITT)</u>
The intention-to-treat (ITT) set will consist of all subjects who were randomized in the study. Subjects who received the wrong treatment will be analyzed as randomized.

<u>Per Protocol Set</u>
The per protocol
(PP) set will consist of a subset of the ITT who completed the study and who fulfill the followingcriteria:
Received at least one injection of Coronavirus-Like Particle COVID-19 Vaccine,
Completed the study with no major deviations related to subject eligibility,
Ability to develop a valid immune response,
No use of prohibited medication,
No events impacting the immunogenicity sample analyses.

The PP analysis set will be further defined for the following analysis timepoint:

<u>Medicago/Cytel</u> Confidential: This is a controlled document and shall not be changed, saved, copied or reproduced by any unauthorized person.

For the analysis of Day 21 timepoint, this should include the subjects who received thefirst vaccine dose and had Day 0 and Day 21 immunogenicity sample collections.
For the analysis of Day 42 timepoint, this should include the subjects who received bothvaccine doses and had Day 0 and Day 42 immunogenicity sample collections.
For the analysis of Day 201 timepoint, this should include the subjects who received bothvaccine doses and had Day 0 and Day 201 immunogenicity sample collections.
For the analysis of Day 386 timepoint, this should include the subjects who received bothvaccine doses and had Day 0 and Day 386 immunogenicity sample collections.

Note: Subjects who had blood samples for immunogenicity taken outside of the time window for blood sample collection are to be excluded from the PP set for the specific visit.
Subjects will be analyzed as treated. Subjects who receive a treatment at the second vaccination that is not the same treatment received at the first vaccination will not be included in the PP set atDay 42, Day 201 and Day 386; the subjects will still be included in the Day 21 analysis and analyzed as treated.

The analyses of all immunogenicity endpoints will be performed using the PP set, as the primary analysis set, and the ITT, as a secondary analysis set.

Protocol Deviations

A protocol deviation is any change, divergence, or departure from the study design or proceduresof a study protocol. Prematurely terminating study participation for reasons such as withdrawal of consent or occurrence of adverse events (including death) is not considered as a protocol deviation. The missing assessments that should have otherwise been collected for that subject later in the study are also not considered as a protocol deviation.

Protocol deviations will be identified and documented during the conduct of the study and classified as major versus minor protocol deviations. At the discretion of the sponsor, major protocol deviations as determined by a review of the data prior to database lock and unblinding of the study results may result in the removal of a subject's data from the PP analysis set.

The sponsor, or designee, will be responsible for producing the final protocol violation file (formatted as a Microsoft Excel file), in collaboration with the data monitoring group as applicable; this file will include a description of the protocol violation, the classification (major versus minor) and will clearly identify whether or not a deviations warrants exclusion from the PP Analysis set. This file will be finalized prior to hard database lock. All deviations will be included in the clinical study reports.

Medicago/Cytel Confidential: This is a controlled document and shall not be changed, saved, copied or reproduced by any unauthorized person.

STATISTICAL METHODS

Responsibilities
Cytel will perform the statistical analyses and is responsible for the production and quality controlof all tables, listings and figures (TLFs). Medicago will perform review of all tables, figures andlistings before the finalization.

General Statistical Methods and Data Handling

General Methods

All outputs will be incorporated into Word files, sorted and labelled according to the International Conference on Harmonisation (ICH) recommendations, and formatted to the appropriate page size(s).

The following labels will be used for the dose level:

CoVLP 3.75µg

CoVLP 7.5µg

CoVLP 15µg

The following labels will be used for the adjuvant type:

Unadjuvanted

Adjuvanted with CpG 1018

Adjuvanted with AS03

The following labels will be used for the treatment

CoVLP 3.75µg unadjuvanted

CoVLP 3.75µg adjuvanted with CpG 1018

CoVLP 3.75µg adjuvanted with AS03

CoVLP 7.5µg unadjuvanted

CoVLP 7.5µg adjuvanted with CpG 1018

CoVLP 7.5µg adjuvanted with AS03

Medicago/Cytel Confidential: This is a controlled document and shall not be changed, saved, copied or reproduced by any unauthorized person.

CoVLP 15µg unadjuvanted

CoVLP 15µg adjuvanted with CpG 1018

CoVLP 15µg adjuvanted with AS03

General Methods for listings

All subject data, including those derived, will be presented in individual subject data listings. All listings will be sorted by dose level, treatment group, subject number and scheduled visit when applicable.

All individual data (except for derived) will be listed as collected. Unscheduled measurements will be included in listings.

General Methods for Tables

Tabulations will be produced for appropriate demographic and baseline characteristics, safety and immunogenicity parameters. All data will be analyzed by dose level and treatment group, and scheduled timepoints as appropriate. Unscheduled visit will only be included in listings and hencewill not be used for statistical analysis or summary tables. Information will be reported by treatment group and overall within each dose level as well as overall treatments, regardless of dose level, unless otherwise indicated.

Presentation for continuous variables:

Continuous variables will be summarized using descriptive statistics, including number ofsubjects (n), number of subjects with missing values, mean (or geometric mean as applicable), median, standard deviation (SD), minimum (Min) and maximum (Max).

Presentation for categorical variables:

For categorical variables, summaries will include counts of subjects and percentages within each category (with a category for missing data) of the parameter. Unless otherwise stated thecalculation of proportions will be based on the number of subjects of the analysis set of interest. Therefore, counts of missing observations will be included in the denominator and a category. “Missing” will be presented. In case the analysis refers to certain visits, percentages will be basedon the number of subjects still present in the study at that visit, unless otherwise specified.

Precision:

For non-derived data, the following will be applied:

Medicago/Cytel Confidential: This is a controlled document and shall not be changed, saved, copied or reproduced by any unauthorized person.

Mean, Median: One more decimal place than the data in SDTM;

Minimum, Maximum: Same precision as available in the SDTM;

SD: Two more decimal place than the data in SDTM;

Geometric mean titer (GMT): One more decimal place than the data in SDTM;

Geometric mean fold rise (GMFR) including LL and UL of 95% CI: one moredecimal place than the data in SDTM.

Statistics on derived data will be rounded to reasonable digits whereas maximal digits would be available in ADaM datasets.

Percentages will be rounded to one decimal place.

Statistical testing:

Formal statistical hypothesis testing will be performed on the primary, secondary and exploratory immunogenicity endpoints. All p-values provided will be exploratory in nature. Unless otherwise stated, all statistical testing will be two-sided and will be performed using a significance (alpha) level of 0.05. Summary statistics will be presented, as well as two-sided 95% confidence intervalson selected parameters, as described in the sections below.

Computing Environment

All descriptive statistical analyses will be performed using SAS statistical software (Version 9.4 or higher), unless otherwise noted. Medical history and adverse events will be coding using Medical Dictionary for Regulatory Activities (MedDRA) version 23.0 or higher. The Prior and Concomitant Medications will be coded in the using World Health Organization (WHO) B3 WHODrug Global – Mar 2020 or higher.

Methods of Pooling Data

Not applicable to the present study.

Adjustments for Covariates

Geometric Mean Fold Rise (GMFR) will be derived using Analysis of Covariance (ANCOVA), with treatment group as main effect and baseline titer as covariate.

Multiple Comparisons/Multiplicity

Multiplicity is not of concern for this study with a descriptive interpretation.

Medicago/Cytel Confidential: This is a controlled document and shall not be changed, saved, copied or reproduced by any unauthorized person.

Subpopulations

Some safety analysis (refer to section 5.6.2 Adverse Events by subgroups) will be repeated onthe following subgroups:

Sex: Male, Female;

Race: White; Black or African American; Asian.

Withdrawals, Dropouts, Loss to Follow-up

Subjects who withdrew early from the study will not be replaced. If a subject is randomized in error (i.e. does not meet eligibility criteria) and has not been vaccinated, this subject will be included in analyses based on the Intent-to-Treat analysis set. Additional subjects could be randomized to ensure required number of treated subjects.

Missing, Unused, and Spurious Data

In general, there will be no substitutions made to accommodate missing data points. All data recorded on the CRF will be included in data listings that will accompany the clinical study report.

For a given subject and a given efficacy/immunogenicity measurement, missing measurementswill not be replaced.

<u>Missing or partial AE date:</u>

No imputation of AE partial/missing start/end dates or times will be performed. In case of missingor incomplete AE onset date/time, the AE will be included in the analysis unless the incomplete AE onset date/time information unequivocally indicates that the AE started prior to Day 0 (refer to section 5.6.1 Adverse Events for further details).

<u>Missing or partial concomitant medication start date:</u>
If only DAY is missing, use the first day of the month.
If DAY and Month are both missing, use the first day of the year.

<u>Missing or partial concomitant medication stop date:</u>
If only DAY is missing, use the last day of the month.
If DAY and Month are both missing, use the last day of the year.
If DAY, Month and year are all missing, assign ‘continuing’ status to stop date.

Medicago/Cytel Confidential: This is a controlled document and shall not be changed, saved, copied or reproduced by any unauthorized person.

Visit Windows

The collected data will be summarized by scheduled visits based on the scheduled events indicatedin Table 2. The visits indicated on the electronic Case Report Form (eCRF) will be used as the analysis visits.

Definition

Baseline definition:

If not otherwise specified, baseline will refer to the last non-missing measurement recorded beforethe date and time of the first vaccination. If no baseline evaluation exists, then baseline value willbe treated as missing. Test for SARS CoV-2, height, weight and Body Mass Index (BMI) and immunogenicity assessments performed on the day of the first vaccination (Day 0) will be considered as baseline. For laboratory parameters and vital signs, the following will be considered:

Baseline for first vaccination: last non-missing measurement recorded before the date and time of the first vaccination

Baseline for second vaccination (only applicable if second vaccination occurred):last non-missing measurement recorded before the date and time of the second vaccination

Post baseline definition:

Post baseline measurements will refer to any measurements recorded after date and time of thefirst vaccination.

For laboratory parameters and vital signs, the following will be considered:

Post baseline assessment for first vaccination: any measurements recorded after date and time of the first vaccination and before date and time of vaccination 2 (ifavailable)

Post baseline assessment for second vaccination (only applicable if second vaccination occurred): any measurements recorded after date and time of the second vaccination.

Study Day 0 and Day 21:

Study Day 0 (Day 0) will correspond to the day of the first vaccination;

Study Day 21 (Day 21) will correspond to the expected day of the secondvaccination.

Onset day:

Medicago/Cytel Confidential: This is a controlled document and shall not be changed, saved, copied or reproduced by any unauthorized person.

The onset day of an event according to first vaccination date will be computed as the onset dateof the event – date of the first vaccination.

The onset day of an event according to second vaccination date will be computed as the onset dateof the event – date of the second vaccination.

Example:

First vaccination date	Second vaccination date	Event onset date	Onset Day according to first vaccination	Onset Day according to secondvaccination
01JUL2020	22JUL2020	25JUL2020	24	3

Computation of Derived data

Age at time of consent

Age at time of consent (in years) will be defined as (date of informed consent – date of birth +1) / 365.25.

Body mass Index (BMI)

BMI (kg/m^2) is calculated using the following formula: Weight (kg) / $Height^2$ (m^2). BMI at baseline will be calculated using the baseline weight and height.

Temperature - Fahrenheit to Celsius conversion

Temperature (T) recorded in degree Fahrenheit (°F) will be converted in degree Celsius (°C)using the following formula: T(°C) = (T(°F) – 32)*0.5556.

Change from baseline

Change from baseline will be defined as post baseline value – baseline value.

Handling of reported values with “<” sign or values reported as Below Limit ofQuantification (BLQ)

Laboratory parameters values collected under the form “<y” will be imputed as y/2 for the analysis. Laboratory values collected under the form “>y” will be imputed as y for the analysis. The original results (“<y”, “>y”) will still be displayed in the listing.

Medicago/Cytel Confidential: This is a controlled document and shall not be changed, saved, copied or reproduced by any unauthorized person.

Nab titers reported below the lower limit of quantitation (BLQ, <10) will be attributed a value of5.

IgG and/or IgM (ELISA) titers reported below the lower limit of quantitation (BLQ, <100) will be attributed a value of 50 by the laboratory. No imputation will be done at the programming level.

Subject Disposition

All subjects who provide informed consent will be accounted for in this study. Subject dispositionwill be summarized by treatment group and overall within each dose level as well as overall treatments, regardless of dose level. Subject disposition information will be summarized as follows:

The number of screened subjects i.e. who signed an Informed consent;

The number of subject screen failures;

The number of randomized subjects (according to assigned treatment);

The frequencies and percentages of subjects who are vaccinated in each analysis set, the number of subjects who study completed through Day 21, Day 42, Day 201, the number of subjects who completed the study and the number of subjects who withdraw study earlyalong with primary reasons for withdrawal. The number of subjects vaccinated will be used as the denominator for the percentage calculation. Information will be reported according to actual treatment received at first dose.

Of note a subject will be considered as having completed through Day 21, Day 42, Day 201 and Day 386 if he/she had the corresponding visit (i.e. visit date none missing).

Subject disposition information will be listed. It will include analysis set flag, completion/discontinuation status and for those subjects who for those who discontinued early, the specific reason(s) for discontinuation. A listing restricted to subjects who withdraw along withreason for discontinuation will be provided. Screen failures will also be listed along with reason for not being randomized.

Demographic and Baseline Characteristics

Demographic and Baseline Characteristics will be summarized by treatment group and overall within each dose level as well as overall treatments, regardless of dose level.

Demographics

Demographic data and SARS-CoV-2 immunization status at baseline will be presented in listingsand summarized on the SAS, ITT (only if ITT analysis set is not identical to the SAS analysis set), and PP analysis sets.

Medicago/Cytel Confidential: This is a controlled document and shall not be changed, saved, copied or reproduced by any unauthorized person.

Continuous baseline variables including age (in years) at time of informed consent, weight (in kg), height (in cm), and BMI (in kg/m^2) will be summarized by means of descriptive statistics. Count and percentage of subjects will be presented for gender at birth, race, ethnicity, and resultsof test for SARS-CoV-2 at baseline (Absence of SARS-CoV-2 antibodies, Presence of SARS- CoV-2 Antibodies).

Serology tests for HIV, Hepatitis B, and Hepatitis C performed at screening will be listed.

Protocol Deviations

The major protocol deviations will be summarized as follows on the SAS population:

Number and proportion of subjects with at least one major protocol deviation;

Number and proportion of subjects with at least one major protocol deviation bycategory of major protocol deviations.

A listing presenting the analysis sets inclusion status (yes, no) will be presented on the All analysisset. For the PP analysis set, the reason for exclusion will be provided.

A listing will include the all protocol deviations identified based on protocol deviation logs (see section 4.2 for further details) on randomized patients and presented along with the date the deviation occurred, and deviation category.

Medical History

Medical history data will be tabulated for the SAS.

The frequencies and percentages of subjects with medical history findings will be presented by MedDRA system organ class (SOC) and Preferred Term (PT). The SOC will be order accordingto the internationally agreed order (please refer to Appendix 6 - Ordering of System Organ Class. Within SOC, PTs will be ordered alphabetically.

The details of medical history findings will be listed.

Prior and Concomitant Medications

As per protocol, concomitant medications must be reported (reason for use, dates of administration, dosage, and route) if the use meets the following conditions:

Within 30 days preceding vaccination: any treatments and/or medications specifically contraindicated (e.g. vaccines, any immunoglobulins or other blood products, or any immune modifying drugs, etc.);

Medicago/Cytel Confidential: This is a controlled document and shall not be changed, saved, copied or reproduced by any unauthorized person.

From randomization to Day 42, inclusive: any medication (including, but not limited to, over-the-counter medicines such as aspirin or antacids), vitamins, and mineral supplements;

From Day 43 to the end of the study, inclusive: any concomitant medication(s) administeredto treat an AESI, SAE, or AE leading to withdrawal; any concomitant medication used to treat an AE that occurred before Day 42 and that is still being used afterwards (i.e. on-goinguse);

Any concomitant medication used to treat conditions reported as medical history;

Any investigational medication or vaccine; any vaccine not foreseen in the study protocol.

Medications used in this study will be coded by using the World Health Organization Drug Dictionary Enhanced (WHO DDE).

Prior medications are medications used only before the first study vaccination (medication end date < first study vaccination date).

Concomitant medications are defined as those medications with a start date on or after first vaccination or the medications started before the first vaccination and continued on or after the first vaccination.

Prior and concomitant medications will be summarized on the SAS. Information will be tabulated descriptively (number and percent of subjects) by Anatomical Therapeutic Chemical (ATC) class (ATC Level 1) and preferred term (PT) (or ATC Level 3). ATC classes and PTs will be sorted by alphabetical order.

Prior and concomitant medications will be listed with ATC classification and preferred term (or ATC Level 3). Concomitant medications will be flagged.

Exposure Analysis

Exposure analyses will be conducted using the SAS, according to actual treatment received atfirst dose. Information will be summarized by treatment group and overall within each dose level as well as overall treatments, regardless of dose level.

The following information will be tabulated by dose level and treatment as well as overall:
Number of subjects receiving first vaccination;
Number of subjects receiving second vaccination: Yes, No and if No, reason.

The reason for not receiving the second dose will be collected on “Contraindications forSubsequent Vaccination” CRF form. Information collected on the exposure CRF page will be listed.

Medicago/Cytel Confidential: This is a controlled document and shall not be changed, saved, copied or reproduced by any unauthorized person.

Safety Analyses
Safety analyses will be conducted using the SAS and presented by treatment group and overall within each dose level as well as overall treatments, regardless of dose level. No formalhypothesis-testing analysis of adverse events incidence rates will be performed.

Adverse Events

Adverse event definitions are provided on Table 3. All analyses described in this section will be based on AE with an onset date on or after the date and time of the first vaccination (Day 0) if not otherwise specified. In case of missing or incomplete AE onset date/time, the AE will be includedin the analysis unless the incomplete AE onset date/time information unequivocally indicates thatthe AE started prior to Day 0, e.g. if a subject is vaccinated on the 15JUL2020 and the AE onset date is XXJUN2020, then the AE will not be included in the analysis. If the AE onset date is JUL2020, then the AE will be included in the analysis.

Adverse events analyses will be presented for the following reporting period, as applicable:
Day 0-21 period after vaccination 1: Any AEs meeting the following criteria will be allocated to the first vaccination (vaccination 1) and included in the Day 0-21 analysis forfirst vaccination:
Any AEs with:
onset date/time < date and time of second vaccine administration, ifsecond vaccination is done,
else if vaccination 2 not performed, any AEs with onset date ≤ date ofvaccination 1+ 21
Day 0-21 period after vaccination 2: Any AEs meeting the following criteria will be allocated to second vaccination (vaccination 2) and included in the Day 0-21 analysis forsecond vaccination:
Any AEs with onset date/time ≥ Date/time of vaccination 2 and with onset date
≤ date of vaccination 2 + 21.
Day 0-21 period after last vaccination: Any AEs meeting the following criteria will beincluded in the Day 0-21 period after last vaccination:
Any AEs with onset date ≤ date of last vaccination + 21 i.e.:
Any AEs with onset date ≤ date of vaccination 2 + 21, if vaccination 2 isperformed
Any AEs with onset date ≤ date of vaccination 1 + 21, if vaccination 2 isnot performed
Day 0 to Day 201: Any AEs with onset date ≤ Day 201 visit date (or vaccination 1 + 201if Day 201 visit not performed).

Medicago/Cytel Confidential: This is a controlled document and shall not be changed, saved, copied or reproduced by any unauthorized person.

Day 0 to End of Study (EOS): Any AEs recorded in the database
Day 22 after last vaccination to Day 201: Any AEs recorded in the database at time ofanalysis and meeting the following criteria will be included in the Day 22 after last vaccination – Day 201 analysis:
Any AEs with date of last vaccination + 21 < onset date ≤ Day 201 visit date (ordate of vaccination 1 + 201 if Day 201 visit is missing) i.e.:
Any AEs with date of vaccination 2 + 21 < onset date ≤ Day 201 visitdate (or date of vaccination 1 + 201 if Day 201 visit is missing), if vaccination 2 is performed
Any AEs with date of vaccination 1 + 21 < onset date ≤ Day 201 visitdate (or date of vaccination 1 + 201 if Day 201 visit is missing), if vaccination 2 is not performed

Day 201 to EOS: Any AEs recorded in the database at time of analysis with onset date >Day 201 visit date (or date of vaccination 1 + 201 if Day 201 visit is missing)

Note: If an AE allocation to a reporting cannot be unequivocally ascertained due to partial/missingdate, the AE will be allocated to all relevant period by. E.g. if a subject is vaccinated on the 01AUG2020 and then on the 23AUG2020 and has an AE with onset date on XXAUG2020, the AE will be allocated to Day 0-21 period after vaccination 1 and Day 0-21 period after vaccination2.

Unless otherwise stated, AEs will be displayed in terms of frequency tables (number of subjects and percentage) by MedDRA SOC and PT. The SOC will be order according to the internationallyagreed order (please refer to Appendix 6 - Ordering of System Organ Class) and within SOC, PTs will be ordered alphabetically.

Where the same reaction or same adverse event, based on preferred terminology, is reported multiple times for the same subject in a treatment period, the subject will only be counted once inthe preferred terminology level in summary frequency tables. It will be assigned the highest observed severity and the strongest relationship to vaccination among those events for the tablesin which those characteristics are summarised.

The maximum event severity will be considered to be the greatest severity associated with each event according to the following order: mild < moderate < severe < potentially life-threatening. Causality will be presented as “Unrelated” (definitely not related and probably not related) and “Related” (possibly related, probably related and definitely related). Missing causality will not beimputed. For analysis reporting information by relationship, if a subject experiences both related

Medicago/Cytel Confidential: This is a controlled document and shall not be changed, saved, copied or reproduced by any unauthorized person.

and unrelated events in the reporting period of interest, the subject will be reported only once under "Related" category.

Overall Summary:

Summary tables presenting an overall summary of solicited and unsolicited AEs will be provided.The following information will be provided:

Overall summary of safety for each Day 0-21 vaccination period:

Any Immediate AEs (occurring within 30 minutes after vaccination);

Immediate solicited AE;

Solicited injection site AE

Solicited systemic AE

Immediate unsolicited AE;

Any solicited AE:

Solicited injection site AE;

Solicited systemic AE;

Severe and potentially life-threatening solicited reactions;

Severe and potentially life-threatening related solicited reactions;

Unsolicited AEs;

Severe and potentially life-threatening unsolicited AEs;

Related unsolicited AEs;

Severe and potentially life-threatening related unsolicited AEs;

Serious AEs;

Related Serious AEs;

AEs leading to study withdrawal;

AEs leading to death

AESI: Overall, VED, Hypersensitivity, potential immune-mediated diseases

Medicago/Cytel Confidential: This is a controlled document and shall not be changed, saved, copied or reproduced by any unauthorized person.

Overall summary of safety for "Day 22 after last vaccination to Day 201" (for Day 201 analysis)and "Day 201 to EOS" (for Day 386 analysis):

Serious AEs;

Related Serious AEs

AEs leading to study withdrawal

AESI: Overall, VED, Hypersensitivity, potential immune-mediated diseases

AEs leading to death

Summary by type of events for each Day 0-21 vaccination period:

Summary tables presenting the number and proportion of subjects with at least one of the following events will be provided for Day 0-21 vaccination period (vaccination 1 and vaccination2):

Immediate events:

Immediate AEs (occurring within 30 minutes after vaccination): overall and by type ofevents

Immediate AEs (occurring within 30 minutes after vaccination) by Maximum Intensity:overall and by type of events

Related Immediate AEs (occurring within 30 minutes after vaccination) by MaximumIntensity: overall and by type of events

Solicited events:

Solicited AEs (Day 0 to Day 7): overall and by type of solicited events

Solicited AEs (Day 0 to Day 7): by Maximum Intensity: overall and by type of solicitedevents

Related Solicited AEs (Day 0 to Day 7): by Maximum Intensity: overall and by type ofsolicited events

Unsolicited events:

Unsolicited AEs (Day 0 to Day 21) by SOC and PT

Unsolicited AEs (Day 0 to Day 21) by SOC and PT and by Maximum Intensity

Medicago/Cytel Confidential: This is a controlled document and shall not be changed, saved, copied or reproduced by any unauthorized person.

Related Unsolicited AEs (Day 0 to Day 21) by SOC and PT and by Maximum IntensityOther

events:

Serious AEs (Day 0 to Day 21) by SOC and PT

Related Serious AEs (Day 0 to Day 21) by SOC and PT

AEs leading to study withdrawal (Day 0 to Day 21) by SOC and PT

AESI (Day 0 to Day 21) by SOC and PT: VED, Hypersensitivity, potential immune-mediated diseases

Related AESI (Day 0 to Day 21) by SOC and PT: VED, Hypersensitivity, potentialimmune-mediated diseases

Summary by type of events for the "Day 22 after last vaccination to Day 201" (for Day 201analysis) and "Day 201 to EOS" (for Day 386 analysis):

Summary tables presenting the number and proportion of subjects with at least one of thefollowing events after any vaccination will be provided:

Serious AEs by SOC and PT

Related Serious AEs by SOC and PT

AEs leading to study withdrawal by SOC and PT

AESI by SOC and PT: VED, Hypersensitivity, potential immune-mediated diseases

Related AESI by SOC and PT: VED, Hypersensitivity, potential immune-mediateddiseases

Summary by type of events for the D0 to Day 201/EOS (for Day 201 and Day 386 analyses):

Summary tables presenting the number and proportion of subjects with at least one of thefollowing events after any vaccination will be provided:

Serious AEs by SOC and PT

AESI by SOC and PT: VED, Hypersensitivity, potential immune-mediated diseases

Adverse Events by subgroups

The following tables presenting information will be repeated by sex and race:

Immediate AEs after each vaccination: overall and by type of type events;

Medicago/Cytel Confidential: This is a controlled document and shall not be changed, saved, copied or reproduced by any unauthorized person.

Solicited AEs (Day 0 to Day 7) after each vaccination: overall and by type of solicitedevents;

Unsolicited AEs (Day 0 to Day 21) after each vaccination by SOC and PT;

AESI (Day 0 to Day 21 after last vaccination) by SOC and PT: VED, Hypersensitivity,potential immune-mediated diseases;

Serious AEs (Day 0 to Day 21 after last vaccination) by SOC and PT

AEs leading to study withdrawal (Day 0 to Day 21 after last vaccination) by SOC andPT;

AESI (Day 0 to Day 201/EOS) by SOC and PT: VED, Hypersensitivity, potentialimmune-mediated diseases; (for final D201 analysis)

Serious AEs (Day 0 to Day 201EOS) by SOC and PT (for final D201 analysis):

AEs leading to study withdrawal (Day 0 to Day 201/EOS) by SOC and PT (for finalD201 analysis);

Individual subject listings will be provided for the categories below:

Solicited injection site and systemic reactions Day 0 to Day 7;

Unsolicited AEs;

Serious AEs;

AEs leading to study withdrawal;

AEs leading to death;

AESIs.

Laboratory Data

Laboratory (biochemical, haematological, and urine) measurements will be performed at a central laboratory and will be evaluated as part of screening procedures (prior to eligibility assessment on Day 0), at Day 3, Day 21, Day 24. The list of parameters to be measured are provided in Table 4. Clinical laboratory values will be expressed using conventional <*SI*> units for the analysis.

Table 4 - Clinical Laboratory Tests and SI units

**Biochemistry (serum) (SI unit):**	
Sodium (mmol/L)	Alkaline phosphate (ALK) (U/L)

Medicago/Cytel Confidential: This is a controlled document and shall not be changed, saved, copied or reproduced by any unauthorized person.

Potassium (mmol/L)	Alaninetransferase (ALT) (U/L)
Urea (mmol/L)	Aspartatetransferase (AST) (U/L)
Creatinine (umol/L)	Gamma glutamyltransferase (GGT) (U/L)
Glucose (mmol/L)	Cholesterol (total, HDL, LDL) (mmol/L)
Total Bilirubin (umol/L)	Calcium (mmol/L)
Albumin (g/L)	Triglyceride (mmol/L)
Total protein (g/L)	Phosphorus (mmol/L)
Chloride (mmol/L)	

Haematology (SI Unit)

Haemoglobin (g/L)	Mean cell haemoglobin (MCH) (fmol/cell or pg/cell)
Hematocrit (packed cell volume [PVC]) (%)	Mean cell concentration (MCHC) (g/L)
Red blood cells (x 10^{12}/L)	Mean cell volume (MCV) (g/L)
platelets (x10^9/L)	Lymphocytes (x10^9/L)
Mean platelet volume (MPV) (fL)	Monocytes (x10^9/L)
White cell count (total, WBC) (x10^9/L)	Eosinophils (x10^9/L)
Neutrophils (x10^9/L)	Basophils (x10^9/L)

Urinalysis (SI unit): Macroscopic

examination (color,aspect)	Glucose (mmol/L)
pH	Protein (mg/dL)
Specific gravity	Blood

The actual value and change from baseline will be summarized using descriptive statistics for each hematology and clinical chemistry laboratory parameter, at each scheduled timepoint (Day 3, Day 21, and Day 24). For Day 3 and Day 21, change from baseline will be computed using Day 0 measurements. For Day 24, change from baseline will be computed using Day 21 measurements.

As part of the primary objectives of the study, subjects with normal and abnormal urine, haematological, and biochemical values will be summarized: for each parameters (hematology, clinical chemistry, urinalysis), the number and proportions of subjects with clinically significant abnormal results (as reported by the investigator) will be provided overall and by scheduled visit.

Medicago/Cytel Confidential: This is a controlled document and shall not be changed, saved, copied or reproduced by any unauthorized person.

Shift tables for all laboratory tests based on laboratory normal range will be presented: changes from baseline (Day 0 and Day 21) will be presented in pre- versus post-vaccination cross tabulations (with results categorized as below normal range, within normal range, above normal range) for each post-baseline (i.e post Day 0 and Day 21) scheduled visit.

All laboratory data will be provided in data listings. A subset listing will be presented for all abnormal (i.e. outside of normal range) laboratory values.

Vital Signs

Vital signs measurements will be performed as part of screening procedures (prior to eligibility assessment on Day 0), after the post-vaccination 30-minute surveillance period on Day 0, at Day3, Day 21 (prior and after the post-vaccination 30-minute surveillance period), Day 24, Day 42, Day 201 and Day 386.

The following parameters will be measured at each visit:

Systolic Blood Pressure (SBP) (mmHg);

Diastolic Blood Pressure (DBP) (mmHg);

Respiratory Rate (RR) (breaths/min);

Heart Rate (HR) (beats/min);

Oral Temperature (OT) (°C).

Vital signs data (actual values and change from baseline) will be summarized using for each scheduled visit using standard summary statistics.

For Day 0 post vaccination, Day 3 and Day 21 (prior vaccination), change from baseline will be computed using Day 0 measurements. For Day 21 (post vaccination), Day 24, Day 42, Day 201 and Day 386, change from baseline will be computed using Day 21 measurements.

An individual listing will be provided for vital signs measurements by subject.

Physical Examinations

A history- or symptom-directed physical examination will be performed by the Investigator aspart of screening procedures. An individual listing will be provided for Physical Examinationresults by subject.

Electrocardiogram

Electrocardiograms are not planned for this study.

Medicago/Cytel Confidential: This is a controlled document and shall not be changed, saved, copied or reproduced by any unauthorized person.

COVID-19 Assessment or Experience

Information related to experience of any COVID-19-like symptoms will be collected at the following visits Day 1 (phone call), Day 3, Day 8 (phone call), Day 21, Day 22 (phone call), Day24, Day 29 (phone call), Day 42, monthly thereafter until Day 201 and Day 386 visits. All laboratory-confirmed reports of SARS-CoV-2 infection (with or without symptoms) will be monitored and reported in the subject's eCRF for the entire duration the study. Symptomatic cases(i.e. COVID-19) will be reported on the "Adverse Event" screen of the subject's eCRF. A listingpresenting the outcome of SARS-CoV-2 test (absence of SARS-CoV-2 antibodies, Presence of SARS-CoV-2 antibodies) over time will be provided. In addition, for subjects who have been tested positive to SARS-CoV-2 infection, a listing presenting the type of test use will be provided

Exploratory Safety Analysis

Additional exploratory analyses may be performed to further characterize the safety profile of the CoVLP-19 Vaccine. Those analyses will be described in a dedicated document and may not be included in the CSR.

Immunogenicity Analysis

Immunogenicity will be evaluated by the humoral immune response (Nab assay and IgG and/or IgM ELISA) and the CMI response induced in subjects on Days 0, 21, 42, and 201 as well as theIgE antibody response directed against plant glycans on Days 0, 42, 201 and 386.

The analyses of all immunogenicity endpoints will be performed using the PP set, as the primary analysis set, and the ITT set, as a secondary analysis population. Of note, PP analysis set at Day 21, Day 42, Day 201 and Day 386 can be different (see definition in section 4.1).

Regarding pairwise treatment comparisons at each timepoint and for each parameter, the p-values associated with the following comparisons and be reported in the corresponding tables:

All possible pairwise comparison within each dose level (3*3 p-values)

Comparison of the unadjuvanted CoVLP treatments between the 3 dose levels (3 p-values)

Comparison of the CoVLP adjuvanted with CpG 1018 treatments between the 3 dose levels (3 p-values)

Comparison of the CoVLP adjuvanted with AS03 treatments between the 3 dose levels (3p-values)

Medicago/Cytel Confidential: This is a controlled document and shall not be changed, saved, copied or reproduced by any unauthorized person.

Analysis of Primary Endpoints

The primary immunogenicity endpoints are defined in Section 2.9.2

Nab assay analyses:

The following analyses for the Nab assay will be performed on the PP set by treatment group:

Nab Titer at Day 0, Day 21 and Day 42:

Summary statistics including median, minimum and maximum

Geometric Mean Titer (GMT) and corresponding two-sided 95% ConfidenceInterval (CI);

Seroconversion (SC) rate at Day 21, and Day 42 along with corresponding two-sided 95%exact (Clopper-Pearson method): SC rate on Day 21 and Day 42 is defined as the proportion of subjects achieving SC in the analysis set i.e. subjects with:

For subjects with detectable Nab titer at Day 0 (i.e. baseline Nab titer ≥10): a ≥ 4-foldincrease in Nab titers between Day 0 and Day 21 and Day 42, respectively

For subjects with undetectable Nab titer at Day 0 (i.e. baseline Nab titer < 10): Nab titer of ≥ 40 on Day 21 and Day 42, respectively.

Note: For the analysis on ITT the percentages will be calculated based on available data.

Nab titer Fold Rise (Day 21/Day 0, Day 42/Day 0) defined as the ratio of Day 21 (or Day42) versus Day 0 titer:

Summary statistics including median, minimum and maximum;

Geometric Mean Fold Rise (GMFR) and corresponding 95% CI.

The GMT will be calculated and compared between treatment groups using an analysis of variance (ANOVA) on the log-transformed titers (LOG10 function in SAS) with actual dose level(3.75ug, 7.5ug, 15ug) and actual adjuvant type (no adjuvant, CpG 1018, AS03) as main effect aswell as dose level by adjuvant type interaction. At each timepoint, the GMT (and corresponding95% CI) of each treatment will be obtained by exponential back-transformation (anti-log with power 10) of the least square mean (and corresponding 95% CI). All possible treatment pairwisecomparisons will be performed using Tukey's adjustment method. Only p-values associated withtreatment pairwise comparison of interest (as presented in section 5.7) will be displayed in the outputs.

SAS code sample (SAS code will be fully validated at the analysis stage):

```
PROC MIXED data=tab(where=(avisitn = Day <0,21,42,201,386>)); ** to be repeated for each relevant timepoints
CLASS DL Adj;
```


Medicago/Cytel Confidential: This is a controlled document and shall not be changed, saved, copied or reproduced by any unauthorized person.

```
MODEL var = DL Adj DL*Adj; where var=LOG10(aval)
LSMEANS DL*AdJ / DIFF=ALL CL ALPHA=0.05 ADJUST=tukey;
RUN;
```

Note: for analysis on ITT, subjects will be analyzed as randomized, the planned dose level and adjuvant type) will be used for the ITT analysis.

The GMFR at Day 21 will be calculated using an ANCOVA on the difference between Day 21 and Day 0 of the log transformed titer values, with actual dose level and adjuvant type as main effect, dose level by adjuvant type interaction and log-transformed baseline titer as covariate. TheGMFR (and corresponding 95% CI) will be obtained for each treatment by exponential back- transformation (anti-log with power 10) of the least square means (and corresponding 95% CI).

Similar approach will be used for the computation of the GMFR at Day 42.

All possible treatment pairwise comparisons will be performed using Tukey's adjustment method.Only p-values associated with treatment pairwise comparison of interest (as presented in section5.7) will be displayed in the outputs.

<u>SAS code sample for GMFR at Day 21 (SAS code will be fully validated at the analysis stage):</u>

```
PROC MIXED data=tab;
CLASS DL Adj;
MODEL diff = DL Adj DL*Adj base; where diff=LOG10(Day21) – LOG10(Day0); base=LOG10(Day0)
LSMEANS DL*Adj/ DIFF=ALL CL ALPHA=0.05 ADJUST=tukey;
RUN;
```

Fisher's exact test will be used to compare SC rate between the treatment groups. An overall p- value of treatment effect will be provided at each timepoint. Pairwise comparisons will be performed without any adjustment method.

<u>Sample SAS code (SAS code will be fully validated at the analysis stage):</u>

```
** SC rate (and 95% CI) at specific visit by treatment group;

PROC FREQ data=tab(where=(avisitn = Day <21,42,201,386);
BY trtan;
TABLE SC / binomial;
EXACT binomial;
RUN;


** Treatment comparison (overall);

PROC FREQ data=tab;
TABLE SC*trtan / fisher;
RUN;
```

For the pairwise comparisons, a WHERE statement will need to be added to select the 2 treatments.

Medicago/Cytel Confidential: This is a controlled document and shall not be changed, saved, copied or reproduced by any unauthorized person.

In addition, a Reverse Cumulative Distribution Curves (RCDC) of log2-transformed Nab titer data will be provided by timepoint.

CMI assay analyses (ELISpot):

The specific Th1 CMI response induced on Day 0, Day 21 and Day 42 will be measured by the number of T cells expressing IFN-γ, using ELISpot, for each treatment group. The specific Th2 CMI response induced on Day 0, Day 21, and Day 42 will be measured by the number of T cellsexpressing IL-4, using ELISpot, for each treatment group.

Descriptive summary statistics of the number of T cells expressing IFN-γ and of T cells expressingIL-4 will be provided by timepoint and treatment group, unless otherwise specified. 95%distribution-free confidence interval will be provided for the median (using the CIPCTLDF optionon the PROC UNIVARIATE statement). For Day 0, descriptive summary statistics of the numberof T cells expressing IFN-γ and of T cells expressing IL-4 will be provided overall and not split by treatment arm.

The number of T cells expressing IFN-γ and of T cells expressing IL-4 will be analysed using appropriate non-parametric (Wilcoxon) models. Pairwise comparisons will be performed withoutany adjustment for multiplicity:

Kruskal-Wallis test (from PROC NPAR1WAY, based on asymptotic chi-square distribution of the test statistic) will be used for the overall treatment, dose level and adjuvant type comparisons of the T-cells number at Day 21 and Day 42. Wilcoxon rank- sum test (based on asymptotic normal distribution of the test statistic) will be used for allpairwise treatment comparisons at a given timepoint.

SAS code sample for treatment comparison of the T-cells numbers at Day 21 (SAS code will be fully validated at the analysis stage):

```
proc NPAR1WAY data=datain(where=(visit=Day 21)) wilcoxon;
            class trtan;
    ** for pairwise treatment comparison use: where trtan in (A B);
            var nTcells;

  run;
```

SAS code sample for dose level comparison of the T-cells numbers at Day 21 (SAS code will be fully validated at the analysis stage):

```
proc NPAR1WAY data=datain(where=(visit=Day 21)) wilcoxon;
```


Medicago/Cytel Confidential: This is a controlled document and shall not be changed, saved, copied or reproduced by any unauthorized person.

```
            class doselevel;
            var nTcells;

  run;
```

<u>SAS code sample for adjuvant type comparison of the T-cells numbers at Day 21 (SAS code will be fully validated at the analysis stage):</u>

```
proc NPAR1WAY data=datain(where=(visit=Day 21)) wilcoxon;
            class adjuv;
            var nTcells;

  run;
```

In addition, the following the following figures will be provided:

Box plot of the number of T cells expressing IFN-γ by timepoint and treatment group

Box plot of the number of T cells expressing IL-4 by timepoint and treatment groupAll analyses listed above will be repeated on the ITT analysis set.

An individual listing will be provided to include all assay results (Nab, Th1 and Th2 CMI response) and whether a particular subject has seroconverted based on ITT set. Subjects included in the PP analysis sets will be flagged.

Analysis of Secondary Endpoints

The secondary immunogenicity endpoints are defined in Section 2.9.2.

The following analyses for the total IgG and/or IgM antibody response will be performed bytreatment group on the PP set:

Titer at Day 0, Day 21, Day 42, Day 201 and Day 386:

Summary statistics including median, minimum and maximum;

GMT (and 95% CI) at Day 0, Day 21, Day 42, Day 201 and Day 386;

Titer Fold Rise (Day 21/Day 0, Day 42/Day 0, Day 201/Day 0 and Day 386/Day 0):

Summary statistics including median, minimum and maximum;

GMFR along with 95% CI.

Medicago/Cytel Confidential: This is a controlled document and shall not be changed, saved, copied or reproduced by any unauthorized person.

The following analyses for the Day 201 and Day 386 Nab assay will be performed for eachtreatment group on the PP set:

Titer at Day 201 and Day 386:

Summary statistics including median, minimum and maximum;

GMT (and 95% CI);

SC rate (and 95% CI) at Day 201 and Day 386;

Titer Fold rise (Day 201/Day 0, Day 386/Day 0):

Summary statistics including median, minimum and maximum;

GMFR and 95% CI.

The same methodology as in section 5.7.1 will be used.

The specific Th1 and Th2 CMI response induced on Day 201 and Day 386 will be analyzed according to the same methodology as in section 5.7.1.

Analysis of Exploratory Endpoints

The CMI response induced on Day 0, Day 21, Day 42, Day 201 and Day 386 will be measured by the percentage of T cells expressing functional markers, using flow cytometry, for each treatment group.

Descriptive summary statistics of the percentage of T cells expressing functional markers will be provided by timepoint and treatment group. The number of T cells expressing functional markerswill be compared using non-parametric (Wilcoxon) models as described in section 5.7.1.

Individual listing will be provided to include flow cytometry CMI CD4+ T cells response for each parameter by subject.

The specific antibody response against plant glycans induced on Day 0, Day 42, Day 201 and Day 386 will be analyzed by evaluating the percentage of subjects with detectable IgE levels at each timepoint for each treatment group and the percentage of subjects with undetectable IgE levels at each timepoint for each treatment group along with corresponding two-sided 95% exact(Clopper-Pearson method). The difference in percentage between Day 42 and Day 0, between Day 201 and Day 0 and between Day 386 and Day 0 and associated 95% CI for the difference will be calculated for each treatment group.

Sample SAS code (SAS code will be fully validated at the analysis stage):

** Difference in percentage along with exact 95% CI for the difference for Day 42–Day 0;

```
PROC FREQ data=tab;
```

```
TABLE trt*avisit*detect / riskdiff(cl=exact);
EXACT riskdiff;
WHERE avisit in ("Day 0" "Day 42");
RUN;
```

An individual listing will be provided to include all IgE assay results based on ITT set.

Additional exploratory analyses may be performed to further characterize the immune response of the CoVLP-19 Vaccine. Those analyses will be described in a dedicated document and will notbe included in the CSR.

Medicago/Cytel Confidential: This is a controlled document and shall not be changed, saved, copied or reproduced by any unauthorized person.

CHANGES TO PLANNED ANALYSES

The following are changes between the protocol-defined statistical analyses and those presented in this statistical analysis plan:

Rewording of the primary safety endpoint:

"Occurrence, intensity and relationship of immediate Adverse Events (AEs) (30 minutes after each vaccination administration)" is used instead of "Percentage, intensity and relationship of immediate Adverse Events (AEs) (30 minutes after each vaccination administration)". "Occurrence" terminology is used instead of "Percentage" in order to allow for definition at the subject level.

CMI assay analyses (ELISpot) – Section 5.7.1:

Due to the limited availability of sample material at Day 0, descriptive summary statistics at Day0 of the number of T cells expressing IFN-γ and of T cells expressing IL-4 will be provided overalland not split by treatment arm. In addition, then all response comparison between timepoints willnot be performed.

Medicago/Cytel Confidential: This is a controlled document and shall not be changed, saved, copied or reproduced by any unauthorized person.

QUALITY CONTROL

SAS programs developed to produce output such as analysis data sets, summary tables, data listings, figures or statistical analyses will follow Cytel statistical and programming Standard Operating Procedures (SOPs).

Medicago/Cytel Confidential: This is a controlled document and shall not be changed, saved, copied or reproduced by any unauthorized person.

REFERENCES

Medicago/Cytel Confidential: This is a controlled document and shall not be changed, saved, copied or reproduced by any unauthorized person.

APPENDICES

Appendix 1 – Day 21 data transfer

The following data will be transferred via a secure platform to the authorized unblinded member(s) of the sponsor for business decisions for Phase 2/3 study:

Randomization information including treatment assigned

Nab titer at Day 0 and Day 21

Specific Th1 CMI response, and Specific Th2 CMI response at Day 0 and Day 21

IgG levels at Day 0 and Day 21

Solicited AEs from Day 0 to Day 7

Medicago/Cytel Confidential: This is a controlled document and shall not be changed, saved, copied or reproduced by any unauthorized person.

Appendix 2 – Day 42 analysis

Tables and Figures to be generated

Disposition/Demographics/Baseline/Exposure (CSR Table Section 14.1)

Table 14.1.1	Summary of Subject Disposition - All Analysis set	Topline output
Table 14.1.2	Summary of Major Protocol Deviations – Safety Analysis Set	
Table 14.1.3.1	Demographic and Baseline Characteristics - Safety Analysis Set	
Table 14.1.3.2	Demographic and Baseline Characteristics - Intent to treat Analysis Set	
Table 14.1.3.3.1	Demographic and Baseline Characteristics - Day 21 Per Protocol Analysis Set	
Table 14.1.3.3.2	Demographic and Baseline Characteristics - Day 42 Per Protocol Analysis Set	
Table 14.1.4.1	Medical History - Safety Analysis Set	
Table 14.1.5.1	Summary of Prior Medications - Safety Analysis Set	
Table 14.1.5.2	Summary of Concomitant Medications - Safety Analysis Set	
Table 14.1.6	Exposure to Study Treatment - Safety Analysis Set	

Immunogenicity Results (CSR Table Section 14.2)

Table 14.2.1	Neutralization Antibody (Nab) Results: Geometric Mean Titer, Geometric Mean Fold Rise, Seroconversion Rate – Per Protocol Analysis Set	Topline output
Figure 14.2.2	Reverse Cumulative Distribution Curves (RCDC) for Neutralization Antibody (Nab) Titers – Per protocol Analysis Set	
Table 14.2.3	Neutralization Antibody (Nab) Results: Geometric Mean Titers, Geometric Mean Fold Rise), Seroconversion Rate by Treatment Group – Intent-To-Treat Analysis Set	
Figure 14.2.4	Reverse Cumulative Distribution Curves (RCDC) for Neutralization Antibody (Nab) Titers – Intent-To-Treat Analysis Set	
Table 14.2.5	Number of T cells expressing IFN-γ (CMI assay results) – Per Protocol Analysis Set	Topline output
Table 14.2.6	Number of T cells expressing IFN-γ (CMI assay results) – Intent-To-Treat Analysis Set	
Table 14.2.7	Number of T cells expressing IL-4 (CMI assay results) – Per-Protocol Analysis set	Topline output

Medicago/Cytel Confidential: This is a controlled document and shall not be changed, saved, copied or reproduced by any unauthorized person.

Table 14.2.8	Number of T cells expressing IL-4 (CMI assay results) – Intent-To-Treat Analysis Set	
Figure 14.2.9	Number of T Cells on Each Parameter by Treatment Group – Per Protocol Analysis Set	
Figure 14.2.10	Number of T Cells on Each Parameter by Treatment Group – Intent-To-Treat Analysis Set	
Table 14.2.11	IgG Antibody Response: Geometric Mean Titers and Geometric Mean Fold Rise – Per Protocol Analysis Set	Topline output
Table 14.2.12	IgG Antibody Response: Geometric Mean Titers, Geometric Mean Fold Rise – Intent-To-Treat Analysis Set	
Table 14.2.13	IgM Antibody Response: Geometric Mean Titers, Geometric Mean Fold Rise – Per Protocol Analysis Set<br>Note: will be produced if data available	
Table 14.2.14	IgM Antibody Response: Geometric Mean Titers, Geometric Mean Fold Rise – Intent-To-Treat Analysis Set<br>Note: will be produced if data available	
Figure 14.2.15	Reverse Cumulative Distribution Curves (RCDC) for IgG and IgM Titers – Per protocol Analysis Set<br>Note: IgM will be included upon availability	
Figure 14.2.16	Reverse Cumulative Distribution Curves (RCDC) for IgG and IgM Titers – Intent-To-Treat Analysis Set<br>Note: IgM will be included upon availability	
Table 14.2.17	Flow cytometry % Response CD4+ Cells on Each Parameter – Per Protocol Analysis Set	
Table 14.2.18	Flow cytometry % Response CD4+ Cells on Each Parameter – Intent-To-Treat Analysis Set	
Table 14.2.19	Serum IgE Levels – Per Protocol Analysis Set	
Table 14.2.20	Serum IgE Levels – Intent-To-Treat Analysis Set	

Displays of Adverse Events (CSR Section 14.3.1)

Table 14.3.1.1	Overall Summary of Adverse events – D0 to D21 after FirstDose Administration - Safety Analysis Set	Topline output
Table 14.3.1.2	Overall Summary of Adverse events – D0 to D21 after Second Dose Administration - Safety Analysis Set	Topline output
Table 14.3.1.3	Summary Incidence of Subjects with Immediate events - D0 to D21 after First Dose Administration - Safety Analysis Set	
Table 14.3.1.4	Summary Incidence of Subjects with Immediate events by Maximum Intensity - D0 to D21 after First Dose Administration - Safety Analysis Set	

Medicago/Cytel Confidential: This is a controlled document and shall not be changed, saved, copied or reproduced by any unauthorized person.

Table 14.3.1.5	Summary Incidence of Subjects with Related Immediate events by Maximum Intensity - D0 to D21 after First Dose Administration - Safety Analysis Set	
Table 14.3.1.6	Summary Incidence of Subjects with Solicited Events - D0 to D21 after First Dose Administration - Safety Analysis Set	
Table 14.3.1.7	Summary Incidence of Subjects with Solicited events by Maximum Intensity - D0 to D21 after First Dose Administration - Safety Analysis Set	
Table 14.3.1.8	Summary Incidence of Subjects with Related Solicited events by Maximum Intensity - D0 to D21 after First Dose Administration - Safety Analysis Set	
Table 14.3.1.9	Summary Incidence of Subjects with Unsolicited AEs by SOC and PT - D0 to D21 after First Dose Administration - Safety Analysis Set	
Table 14.3.1.10	Summary Incidence of Subjects with Unsolicited AEs by SOC and PT and Maximum Intensity - D0 to D21 after First Dose Administration - Safety Analysis Set	
Table 14.3.1.11	Summary Incidence of Subjects with Related Unsolicited AEs by SOC and PT and Maximum Intensity - D0 to D21 after First Dose Administration - Safety Analysis Set	
Table 14.3.1.12	Summary Incidence of Subjects with Serious AEs by SOC and PT - D0 to D21 after First Dose Administration Safety Analysis Set	
Table 14.3.1.13	Summary Incidence of Subjects with Related Serious AEs by SOC and PT - D0 to D21 after First Dose Administration - Safety Analysis Set	
Table 14.3.1.14	Summary Incidence of Subjects with AEs leading to withdrawal by SOC and PT - D0 to D21 after First Dose Administration - Safety Analysis Set	
Table 14.3.1.15	Summary Incidence of Subjects with AESI by Category, SOC and PT - D0 to D21 after First Dose Administration - Safety Analysis Set	Topline output
Table 14.3.1.16	Summary Incidence of Subjects with Related AESI by Category, SOC and PT - D0 to D21 after First Dose Administration - Safety Analysis Set	
Table 14.3.1.17	Summary Incidence of Subjects with Immediate Events - D0 to D21 after Second Dose Administration - Safety Analysis Set	
Table 14.3.1.18	Summary Incidence of Subjects with Immediate Events by Maximum Intensity - D0 to D21 after Second Dose Administration - Safety Analysis Set	

Medicago/Cytel Confidential: This is a controlled document and shall not be changed, saved, copied or reproduced by any unauthorized person.

Table 14.3.1.19	Summary Incidence of Subjects with Related Immediate Events by Maximum Intensity - D0 to D21 after Second Dose Administration - Safety Analysis Set	
Table 14.3.1.20	Summary Incidence of Subjects with Solicited Events - D0 to D21 after Second Dose Administration - Safety Analysis Set	
Table 14.3.1.21	Summary Incidence of Subjects with Solicited Events by Maximum Intensity - D0 to D21 after Second Dose Administration - Safety Analysis Set	
Table 14.3.1.22	Summary Incidence of Subjects with Related Solicited Events by Maximum Intensity - D0 to D21 after Second Dose Administration - Safety Analysis Set	
Table 14.3.1.23	Summary Incidence of Subjects with Unsolicited AEs by SOC and PT - D0 to D21 after Second Dose Administration - Safety Analysis Set	
Table 14.3.1.24	Summary Incidence of Subjects with Unsolicited AEs by SOC and PT and Maximum Intensity - D0 to D21 after Second Dose Administration - Safety Analysis Set	
Table 14.3.1.25	Summary Incidence of Subjects with Related Unsolicited AEs by SOC and PT and Maximum Intensity - D0 to D21 after Second Dose Administration - Safety Analysis Set	
Table 14.3.1.26	Summary Incidence Subjects with Serious AEs by SOC and PT - D0 to D21 after Second Dose Administration - Safety Analysis Set	
Table 14.3.1.27	Summary Incidence of Subjects with Related Serious AEs by SOC and PT - D0 to D21 after Second Dose Administration - Safety Analysis Set	
Table 14.3.1.28	Summary Incidence of Subjects with AEs leading to withdrawal by SOC and PT - D0 to D21 after Second Dose Administration - Safety Analysis Set	
Table 14.3.1.29	Summary Incidence of Subjects with AESI by SOC and PT - D0 to D21 after Second Dose Administration- Safety Analysis Set	Topline output
Table 14.3.1.30	Summary Incidence of Subjects with Related AESI by SOC and PT - D0 to D21 after Second Dose Administration - Safety Analysis Set	

Display of adverse events by subgroups

Table 14.3.1.31	Summary Incidence of Subjects with Immediate events - D0 to D21 after First Dose Administration - Subgroup Analysis - Safety Analysis Set	

Medicago/Cytel Confidential: This is a controlled document and shall not be changed, saved, copied or reproduced by any unauthorized person.

Table 14.3.1.32	Summary Incidence of Subjects with Solicited events - D0 to D21 after First Dose Administration - Subgroup Analysis - Safety Analysis Set	
Table 14.3.1.33	Summary Incidence of Subjects with Unsolicited AEs by SOC and PT - D0 to D21 after First Dose Administration - Subgroup Analysis - Safety Analysis Set	
Table 14.3.1.34	Summary Incidence of Subjects with Immediate events - D0 to D21 after Second Dose Administration - Subgroup Analysis - Safety Analysis Set	
Table 14.3.1.35	Summary Incidence of Subjects with Solicited events - D0 to D21 after Second Dose Administration - Subgroup Analysis - Safety Analysis Set	
Table 14.3.1.36	Summary Incidence of Subjects with Unsolicited AEs by SOC and PT - D0 to D21 after Second Dose Administration - Subgroup Analysis - Safety Analysis Set	
Table 14.3.1.37	Summary Incidence of Subjects with AESI by Category, SOC and PT - D0 to D21 after Last Administration - Subgroup Analysis - Safety Analysis Set	
Table 14.3.1.38	Summary Incidence of Subjects with Serious AEs by SOC and PT – D0 to D21 after Last Dose Administration - Subgroup Analysis - Safety Analysis Set	
Table 14.3.1.39	Summary Incidence of Subjects with AEs leading to withdrawal by SOC and PT - D0 to D21 after Last Dose Administration - Safety Analysis Set	

Listings of Deaths, Serious Adverse Events and Other Significant Events (CSRSection 14.3.2)

Table 14.3.2.1	Listings of Deaths - Safety Analysis Set	
Table 14.3.2.2	Listing of Serious Adverse Events	
Table 14.3.2.3	Listing of Adverse Events Leading to Withdrawal	

Abnormal Laboratory Value Listings (CSR Section 14.3.4)

Table 14.3.4.1	Listing of Abnormal Laboratory Results - Safety Analysis Set	

Other Safety Information (CSR Section 14.4)

Table 14.3.4.2	Summary Statistics of Hematology parameters: Results and Change from Baseline by Visit - Safety Analysis Set	Topline output
Table 14.3.4.3	Summary Statistics of Chemistry parameters: Results andChange from Baseline by Visit - Safety Analysis Set	Topline output

Medicago/Cytel Confidential: This is a controlled document and shall not be changed, saved, copied or reproduced by any unauthorized person.

Table 14.3.4.4	Hematology Laboratory parameters: Number and Proportion of Subjects with abnormal clinically significant results by visit- Safety Analysis Set	
Table 14.3.4.5	Biochemistry Laboratory parameters: Number and Proportion of Subjects with abnormal clinically significant results by visit- Safety Analysis Set	
Table 14.3.4.6	Urinalysis Laboratory parameters: Number and Proportion of Subjects with abnormal clinically significant results by visit- Safety Analysis Set	
Table 14.3.4.7	Hematology Laboratory parameters: Shifts from Day 0 to post Day 0 Category by visit- Safety Analysis Set	
Table 14.3.4.8	Chemistry Laboratory parameters: Shifts from Day 0 to post Day 0 Category by visit- Safety Analysis Set	
Table 14.3.4.9	Urinalysis Laboratory parameters: Shifts from Day 0 to post Day 0 Category by visit- Safety Analysis Set	
Table 14.3.4.10	Hematology Laboratory parameters: Shifts from Day 21 to post Day 21 Category by visit- Safety Analysis Set	
Table 14.3.4.11	Chemistry Laboratory parameters: Shifts from Day 21 to post Day 21 Category by visit- Safety Analysis Set	
Table 14.3.4.12	Urinalysis Laboratory parameters: Shifts from Day 21 to post Day 21 Category by visit- Safety Analysis Set	
Table 14.3.5.1	Summary Statistics of Vital Signs - Safety Analysis Set	

Data Listings to be generated

Discontinued Subjects (CSR Appendix 16.2.1)

Listing 16.2.1.1	Subject Disposition - All Subjects Set	
Listing 16.2.1.2	Subjects Who Withdraw from the Study - All Subjects Set	
Listing 16.2.1.3	Screen Failures - All Subjects Set	
Listing 16.2.1.4	Analysis Populations - All Analysis Set	

Protocol Deviations (CSR Appendix 16.2.2)

Listing 16.2.2.1	All Protocol Deviations - All Subjects Set	

Subjects Excluded from PP Analysis set (CSR Appendix 16.2.3)

Listing 16.2.3.1	Subjects Excluded from Per Protocol Population - Intent-To-Treat Analysis Set	

Medicago/Cytel Confidential: This is a controlled document and shall not be changed, saved, copied or reproduced by any unauthorized person.

Demographics Data (CSR Appendix 16.2.4)

Listing 16.2.4.1	Demographic and Baseline Information- Intent-To-TreatAnalysis Set	
Listing 16.2.4.2	Serology results at screening - Intent-To-Treat Analysis Set	
Listing 16.2.4.3	Medical History- Intent-To-Treat Analysis Set	
Listing 16.2.4.4	Prior Medications - Safety Analysis Set	
Listing 16.2.4.5	Concomitant Medications - Safety Analysis Set	

Compliance and/or Drug Concentration Data (CSR Appendix 16.2.5)

Listing 16.2.5.1	Vaccine Administration - Safety Analysis Set	

Individual Immunogenicity Data (CSR Appendix 16.2.6)

Listing 16.2.6.1	Nab Assay response – Intent-To-Treat Set	
Listing 16.2.6.2	ELISpot CMI Assay Response – Intent-To-Treat Set	
Listing 16.2.6.3	IgG and/or IgM Titers – Intent-To-Treat Set<br>*Note: IgM information will be displayed upon availability*	
Listing 16.2.6.4	Flow cytometry CMI CD4+ cells response – Intent-To-Treat Set	
Listing 16.2.6.5	Serum IgE – Intent-To-Treat Analysis Set	

Adverse Event Listings (each subject) (CSR Appendix 16.2.7)

Listing 16.2.7.1	Solicited injection Site and Systemic Reactions – First Vaccination - Safety Analysis Set	
Listing 16.2.7.2	Solicited Local and Systemic Reactions – Second Vaccination - Safety Analysis Set	
Listing 16.2.7.3	Unsolicited Adverse Events - Safety Analysis Set	
Listing16.2.7.4	Adverse Events of Special Interest - Safety Analysis Set	

Listing of Individual Laboratory Measurements by Subject (CSR Appendix 16.2.8)

Listing 16.2.8.1	Laboratory Results: Hematology - Safety Analysis Set	
Listing 16.2.8.2	Laboratory Results: Biochemistry - Safety Analysis Set	
Listing 16.2.8.3	Laboratory Results: Urinalysis - Safety Analysis Set	

Other Safety Data (CSR Appendix 16.2.9)

Listing 16.2.9.1	Vital Signs - Safety Analysis Set	
Listing 16.2.9.2	Physical Examination - Safety Analysis Set	

Medicago/Cytel Confidential: This is a controlled document and shall not be changed, saved, copied or reproduced by any unauthorized person.

Listing 16.2.9.3	COVID-19 Assessment or experience - Safety Analysis Set	
Listing 16.2.9.4	SARS-Cov-2 test outcome over time	Topline output

Medicago/Cytel Confidential: This is a controlled document and shall not be changed, saved, copied or reproduced by any unauthorized person.

Appendix 3 – Day 201 analysis (interim analysis)

Tables and Figures to be generated

Disposition/Demographics/Baseline/Exposure (CSR Table Section 14.1)

Table 14.1.1	Summary of Subject Disposition - All Analysis set
Table 14.1.3.1	Demographic and Baseline Characteristics - Day 201 Per Protocol Analysis set
Table 14.1.5.2	Summary of Concomitant Medications (Safety Analysis Set)

Immunogenicity Results (CSR Table Section 14.2)

Table 14.2.1	Neutralization Antibody (Nab) Results: Geometric Mean Titer, Geometric Mean Fold Rise, Seroconversion Rate – Per Protocol Analysis Set
Figure 14.2.2	Reverse Cumulative Distribution Curves (RCDC) for Neutralization Antibody (Nab) Titers – Per Protocol Analysis Set
Table 14.2.3	Neutralization Antibody (Nab) Results: Geometric Mean Titers, Geometric Mean Fold Rise), Seroconversion Rate by Treatment Group – Intent-To-Treat Analysis Set
Figure 14.2.4	Reverse Cumulative Distribution Curves (RCDC) for Neutralization Antibody (Nab) Titers – Intent-To-Treat Set
Table 14.2.5	Number of T cells expressing IFN-γ (CMI assay results) – Per Protocol Analysis Set
Table 14.2.6	Number of T cells expressing IFN-γ (CMI assay results) – Intent-To-Treat Analysis Set
Table 14.2.7	Number of T cells expressing IL-4 (CMI assay results) – Per-Protocol Analysis set
Table 14.2.8	Number of T cells expressing IL-4 (CMI assay results) – Intent-To-Treat Analysis Set
Figure 14.2.9	Number of T Cells on Each Parameter by Treatment Group – Per Protocol Set
Figure 14.2.10	Number of T Cells on Each Parameter by Treatment Group – Intent-To-Treat Set
Table 14.2.11	IgG Antibody Response: Geometric Mean Titers and Geometric Mean Fold Rise – Per Protocol Analysis Set
Table 14.2.12	IgG Antibody Response: Geometric Mean Titers, Geometric Mean Fold Rise – Intent-To-Treat Analysis Set
Table 14.2.13	IgM Antibody Response: Geometric Mean Titers, Geometric Mean Fold Rise – Per Protocol Analysis Set<br>*Note: will be provided upon availability of IgM data*

Medicago/Cytel Confidential: This is a controlled document and shall not be changed, saved, copied or reproduced by any unauthorized person.

Table 14.2.14	IgM Antibody Response: Geometric Mean Titers, Geometric Mean Fold Rise – Intent-To-Treat Analysis Set<br>*Note: will be provided upon availability of IgM data*
Figure 14.2.15	Reverse Cumulative Distribution Curves (RCDC) for IgG and IgM Titers – Per protocol Set<br>*Note: IgM data will be displayed upon availability*
Figure 14.2.16	Reverse Cumulative Distribution Curves (RCDC) for IgG and IgM Titers – Intent-To-Treat Set<br>*Note: IgM data will be displayed upon availability*
Table 14.2.17	Flow cytometry % Response CD4+ Cells on Each Parameter – Per Protocol Analysis Set
Table 14.2.18	Flow cytometry % Response CD4+ Cells on Each Parameter – Intent-To-Treat Analysis Set
Table 14.2.19	Serum IgE Levels – Per Protocol Analysis Set
Table 14.2.20	Serum IgE Levels – Intent-To-Treat Analysis Set

Displays of Adverse Events (CSR Section 14.3.1)

Table 14.3.1.1	Overall Summary of Adverse events – Day 22 after last vaccination to Day 201 - Safety Analysis Set
Table 14.3.1.2	Summary Incidence of Subjects with Serious AEs by SOC and PT – Day 22 after last vaccination to Day 201 - Safety Analysis Set
Table 14.3.1.3	Summary Incidence of Subjects with Related Serious AEs by SOC and PT – Day 22 after last vaccination to Day 201 - Safety Analysis Set
Table 14.3.1.4	Summary Incidence of Subjects with AEs leading to withdrawal by SOC and PT – Day 22 after last vaccination to Day 201 - Safety Analysis Set
Table 14.3.1.5	Summary Incidence of Subjects with AESI by Category, SOC and PT – Day 22 after last vaccination to Day 201 - Safety Analysis Set
Table 14.3.1.6	Summary Incidence of Subjects with Related AESI by Category, SOC and PT – Day 22 after last vaccination to Day 201 - Safety Analysis Set
Table 14.3.1.7	Summary Incidence of Subjects with Serious AEs by SOC and PT – Day 0 to Day 201- Safety Analysis Set
Table 14.3.1.8	Summary Incidence of Subjects with AESI by SOC and PT – Day 0 to Day 201 - Safety Analysis Set

Display of adverse events by subgroups

Medicago/Cytel Confidential: This is a controlled document and shall not be changed, saved, copied or reproduced by any unauthorized person.

Table 14.3.1.9	Summary Incidence of Subjects with Serious AEs by SOC and PT – Day 0 to Day 201 - Subgroup Analysis - Safety Analysis Set
Table 14.3.1.10	Summary Incidence of Subjects with AEs leading to withdrawal by SOC and PT – Day 0 to Day 201 - Subgroup Analysis - Safety Analysis Set
Table 14.3.1.11	Summary Incidence of Subjects with AESI by Category, SOC and PT – Day 0 to Day 201 - Subgroup Analysis - Safety Analysis Set

Listings of Deaths, Serious Adverse Events and Other Significant Events (CSR Section 14.3.2)

Table 14.3.2.1	Listings of Deaths - Safety Analysis Set
Table 14.3.2.2	Listing of Serious Adverse Events
Table 14.3.2.3	Listing of Adverse Events Leading to Withdrawal

Other Safety Information (CSR Section 14.4)

Table 14.3.5.1	Summary Statistics of Vital Signs - Safety Analysis Set

Data Listings to be Generated

Discontinued Subjects (CSR Appendix 16.2.1)

Listing 16.2.1.1	Subject Disposition - All Subjects Set
Listing 16.2.1.2	Analysis Populations - All Analysis Set

Demographics Data (CSR Appendix 16.2.4)

Listing 16.2.4.5	Concomitant Medications - Safety Analysis Set

Individual Immunogenicity Data (CSR Appendix 16.2.6)

Listing 16.2.6.1	Nab Assay response – Intent-To-Treat Set
Listing 16.2.6.2	ELISpot CMI Assay Response – Intent-To-Treat Set
Listing 16.2.6.3	IgG and/or IgM Titers – Intent-To-Treat Set<br>*Note: IgM data will be displayed upon availability*
Listing 16.2.6.4	Flow cytometry CMI CD4+ cells response – Intent-To-Treat Set
Listing 16.2.6.5	Serum IgE– Intent-To-Treat Set

Adverse Event Listings (each subject) (CSR Appendix 16.2.7)

Listing 16.2.7.4	Adverse Events of Special Interest - Safety Analysis Set

Other Safety Data (CSR Appendix 16.2.9)

Listing 16.2.9.1	Vital Signs - Safety Analysis Set

Medicago/Cytel Confidential: This is a controlled document and shall not be changed, saved, copied or reproduced by any unauthorized person.

Listing 16.2.9.3	COVID-19 Assessment or experience - Safety Analysis Set
Listing 16.2.9.4	SARS-Cov-2 test outcome over time

Medicago/Cytel Confidential: This is a controlled document and shall not be changed, saved, copied or reproduced by any unauthorized person.

Appendix 4 – Day 386 analysis (final analysis)

Tables and Figures to be generated

Disposition/Demographics/Baseline/Exposure (CSR Table Section 14.1)

Table 14.1.1	Summary of Subject Disposition - All Analysis set
Table 14.1.3.1	Demographic and Baseline Characteristics - Day 386 Per Protocol Analysis set
Table 14.1.5.2	Summary of Concomitant Medications (Safety Analysis Set)

Immunogenicity Results (CSR Table Section 14.2)

Table 14.2.1	Neutralization Antibody (Nab) Results: Geometric Mean Titer, Geometric Mean Fold Rise, Seroconversion Rate – Per Protocol Analysis Set
Figure 14.2.2	Reverse Cumulative Distribution Curves (RCDC) for Neutralization Antibody (Nab) Titers – Per protocol Set
Table 14.2.3	Neutralization Antibody (Nab) Results: Geometric Mean Titers, Geometric Mean Fold Rise), Seroconversion Rate by Treatment Group – Intent-To-Treat Analysis Set
Figure 14.2.4	Reverse Cumulative Distribution Curves (RCDC) for Neutralization Antibody (Nab) Titers – Intent-To-Treat Set
Table 14.2.5	Number of T cells expressing IFN-γ (CMI assay results) – Per Protocol Analysis Set
Table 14.2.6	Number of T cells expressing IFN-γ (CMI assay results) – Intent-To-Treat Analysis Set
Table 14.2.7	Number of T cells expressing IL-4 (CMI assay results) – Per-Protocol Analysis set
Table 14.2.8	Number of T cells expressing IL-4 (CMI assay results) – Intent-To-Treat Analysis Set
Figure 14.2.9	Number of T Cells on Each Parameter by Treatment Group – Per Protocol Set
Figure 14.2.10	Number of T Cells on Each Parameter by Treatment Group – Intent-To-Treat Set
Table 14.2.11	IgG Antibody Response: Geometric Mean Titers and Geometric Mean Fold Rise – Per Protocol Analysis Set
Table 14.2.12	IgG Antibody Response: Geometric Mean Titers, Geometric Mean Fold Rise – Intent-To-Treat Analysis Set
Table 14.2.13	IgM Antibody Response: Geometric Mean Titers, Geometric Mean Fold Rise – Per Protocol Analysis Set<br>*Note: will be provided upon availability of IgM data*

Medicago/Cytel Confidential: This is a controlled document and shall not be changed, saved, copied or reproduced by any unauthorized person.

Table 14.2.14	IgM Antibody Response: Geometric Mean Titers, Geometric Mean Fold Rise – Intent-To-Treat Analysis Set<br>*Note: will be provided upon availability of IgM data*
Figure 14.2.15	Reverse Cumulative Distribution Curves (RCDC) for IgG and IgM Titers – Per protocol Set<br>*Note: IgM data will be displayed upon availability*
Figure 14.2.16	Reverse Cumulative Distribution Curves (RCDC) for IgG and IgM Titers – Intent-To-Treat Set<br>*Note: IgM data will be displayed upon availability*
Table 14.2.17	Flow cytometry % Response CD4+ Cells on Each Parameter – Per Protocol Analysis Set
Table 14.2.18	Flow cytometry % Response CD4+ Cells on Each Parameter – Intent-To-Treat Analysis Set
Table 14.2.19	Serum IgE Levels – Per Protocol Analysis Set
Table 14.2.20	Serum IgE Levels – Intent-To-Treat Analysis Set

Displays of Adverse Events (CSR Section 14.3.1)

Table 14.3.1.1	Overall Summary of Adverse events – Day 201 to EOS - Safety Analysis Set
Table 14.3.1.2	Summary Incidence of Subjects with Serious AEs by SOC and PT – Day 201 to EOS - Safety Analysis Set
Table 14.3.1.3	Summary Incidence of Subjects with Related Serious AEs by SOC and PT – Day 201 to EOS - Safety Analysis Set
Table 14.3.1.4	Summary Incidence of Subjects with AEs leading to withdrawal by SOC and PT – Day 201 to EOS - Safety Analysis Set
Table 14.3.1.5	Summary Incidence of Subjects with AESI by Category, SOC and PT – Day 201 to EOS - Safety Analysis Set
Table 14.3.1.6	Summary Incidence of Subjects with Related AESI by Category, SOC and PT – Day 201 to EOS - Safety Analysis Set
Table 14.3.1.7	Summary Incidence of Subjects with Serious AEs by SOC and PT – Day 0 to EOS- Safety Analysis Set
Table 14.3.1.8	Summary Incidence of Subjects with AESI by SOC and PT – Day 0 to EOS - Safety Analysis Set

Display of adverse events by subgroups

Table 14.3.1.9	Summary Incidence of Subjects with Serious AEs by SOC and PT – Day 0 to EOS - Subgroup Analysis - Safety Analysis Set

Medicago/Cytel Confidential: This is a controlled document and shall not be changed, saved, copied or reproduced by any unauthorized person.

Table 14.3.1.10	Summary Incidence of Subjects with AEs leading to withdrawal by SOC and PT – Day 0 to EOS - Subgroup Analysis - Safety Analysis Set
Table 14.3.1.11	Summary Incidence of Subjects with AESI by Category, SOC and PT – Day 0 to EOS - Subgroup Analysis - Safety Analysis Set

Listings of Deaths, Serious Adverse Events and Other Significant Events (CSR Section 14.3.2)

Table 14.3.2.1	Listings of Deaths - Safety Analysis Set
Table 14.3.2.2	Listing of Serious Adverse Events
Table 14.3.2.3	Listing of Adverse Events Leading to Withdrawal

Other Safety Information (CSR Section 14.4)

Table 14.3.5.1	Summary Statistics of Vital Signs - Safety Analysis Set

Data Listings to be Generated

Discontinued Subjects (CSR Appendix 16.2.1)

Listing 16.2.1.1	Subject Disposition - All Subjects Set
Listing 16.2.1.2	Analysis Populations - All Analysis Set

Demographics Data (CSR Appendix 16.2.4)

Listing 16.2.4.5	Concomitant Medications - Safety Analysis Set

Individual Immunogenicity Data (CSR Appendix 16.2.6)

Listing 16.2.6.1	Nab Assay response – Intent-To-Treat Set
Listing 16.2.6.2	ELISpot CMI Assay Response – Intent-To-Treat Set
Listing 16.2.6.3	IgG and/or IgM Titers – Intent-To-Treat Set<br>*Note: IgM data will be displayed upon availability*
Listing 16.2.6.4	Flow cytometry CMI CD4+ cells response – Intent-To-Treat Set
Listing 16.2.6.5	Serum IgE– Intent-To-Treat Analysis Set

Adverse Event Listings (each subject) (CSR Appendix 16.2.7)

Listing 16.2.7.4	Adverse Events of Special Interest - Safety Analysis Set

Other Safety Data (CSR Appendix 16.2.9)

Listing 16.2.9.1	Vital Signs - Safety Analysis Set
Listing 16.2.9.3	COVID-19 Assessment or experience - Safety Analysis Set
Listing 16.2.9.4	SARS-Cov-2 test outcome over time - Safety Analysis Set

Medicago/Cytel Confidential: This is a controlled document and shall not be changed, saved, copied or reproduced by any unauthorized person.

Appendix 5 - List of Potential Immune-Mediated Diseases

Neuroinflammatory disorders	Musculoskeletal disorders	Skin disorders
Cranial nerve neuropathy, including paralysis and paresis(eg, Bell's palsy).<br>Optic neuritis.<br>Multiple sclerosis.<br>Transverse myelitis.<br>Guillain-Barré syndrome, including Miller Fisher syndrome and other variants.<br>Acute disseminated encephalomyelitis, includingsite specific variants, eg, noninfectious encephalitis, encephalomyelitis, myelitis, myeloradiculoneuritis.<br>Myasthenia gravis, including Lambert-Eaton myasthenic syndrome.<br>Demyelinating peripheral neuropathies and plexopathies including:<br>Chronic inflammatory demyelinating polyneuropathy.<br>Multifocal motor neuropathy.<br>Polyneuropathies associatedwith monoclonal gammopathy.<br>Narcolepsy.<br>Tolosa Hunt syndrome.	Systemic lupus erythematosusand associated conditions.<br>Systemic scleroderma (systemic sclerosis), including:<br>Diffuse scleroderma.<br>CREST syndrome.<br>Idiopathic inflammatory myopathies, including:<br>Dermatomyositis.<br>Polymyositis.<br>Anti-synthetase syndrome.<br>Rheumatoid arthritis and associated conditions including:<br>Juvenile idiopathic arthritis.<br>Still's disease.<br>Polymyalgia rheumatica.<br>Spondyloarthropathies, including:<br>Ankylosing spondylitis.<br>Reactive arthritis (Reiter's syndrome).<br>Undifferentiated spondyloarthritis.<br>Psoriatic arthritis.<br>Enteropathic arthritis.<br>Relapsing polychondritis.<br>Mixed connective tissue disorder.<br>Gout.	Psoriasis.<br>Vitiligo.<br>Erythema nodosum.<br>Autoimmune bullous skin diseases (including pemphigus,pemphigoid, and dermatitis herpetiformis).<br>Lichen planus.<br>Sweet's syndrome.<br>Localized scleroderma (morphea).<br>Cutaneous lupus erythematosus.<br>Rosacea.
**Vasculitis**	**Blood disorders**	**Others**
Large vessels vasculitis including:<br>Giant cell arteritis (temporal arteritis).<br>Takayasu's arteritis.<br>Medium sized and/or small vessels vasculitis including:<br>Polyarteritis nodosa.<br>Kawasaki's disease.<br>Microscopic polyangiitis.	Autoimmune hemolytic anemia.<br>Autoimmune thrombocytopenia.<br>Antiphospholipid syndrome.<br>Pernicious anemia.<br>Autoimmune aplastic anemia.<br>Autoimmune neutropenia.<br>Autoimmune pancytopenia.	Autoimmune glomerulonephritis including:<br>IgA nephropathy.<br>Glomerulonephritis rapidly progressive.<br>Membranous glomerulonephritis.<br>Membranoproliferative glomerulonephritis.<br>Mesangioproliferative glomerulonephritis.

Medicago/Cytel Confidential: This is a controlled document and shall not be changed, saved, copied or reproduced by any unauthorized person.

Wegener's granulomatosis (granulomatosis with polyangiitis).<br>Churg–Strauss syndrome (allergic granulomatous angiitis or eosinophilic granulomatosis with polyangiitis).<br>Buerger's disease (thromboangiitis obliterans).<br>Necrotizing vasculitis (cutaneous or systemic).<br>Antineutrophil cytoplasmic antibody (ANCA) positive vasculitis (type unspecified).<br>Henoch-Schonlein purpura(IgA vasculitis).<br>Behcet's syndrome.<br>Leukocytoclastic vasculitis.		- Tubulointerstitial nephritisand uveitis syndrome.<br><br>Ocular autoimmune diseases including:<br>Autoimmune uveitis.<br>Autoimmune retinitis.<br>Autoimmune myocarditis/cardiomyopathy.<br>Sarcoidosis.<br>Stevens-Johnson syndrome.<br>Sjögren's syndrome.<br>Alopecia areata.<br>Idiopathic pulmonary fibrosis.<br>Goodpasture syndrome.<br>Raynaud's phenomenon.
**Liver disorders**	**Gastrointestinal disorders**	**Endocrine disorders**
Autoimmune hepatitis.<br>Primary biliary cirrhosis.<br>Primary sclerosing cholangitis.<br>Autoimmune cholangitis.	Inflammatory bowel disease, including:<br>Crohn's disease.<br>Ulcerative colitis.<br>Microscopic colitis.<br>Ulcerative proctitis.<br>Celiac disease.<br>Autoimmune pancreatitis.	Autoimmune thyroiditis (including Hashimoto thyroiditis).<br>Grave's or Basedow's disease.<br>Diabetes mellitus type 1.<br>Addison's disease.<br>Polyglandular autoimmune syndrome.<br>Autoimmune hypophysitis.

Medicago/Cytel Confidential: This is a controlled document and shall not be changed, saved, copied or reproduced by any unauthorized person.

Appendix 6 - Ordering of System Organ Class

SOC *Infections and infestations*
SOC *Neoplasms benign, malignant and unspecified (incl cysts and polyps)*
SOC *Blood and lymphatic system disorders*
SOC *Immune system disorders*
SOC *Endocrine disorders*
SOC *Metabolism and nutrition disorders*
SOC Psychiatric disorders
SOC *Nervous system disorders*
SOC *Eye disorders*
SOC *Ear and labyrinth disorders*
SOC *Cardiac disorders*
SOC *Vascular disorders*
SOC *Respiratory, thoracic and mediastinal disorders*
SOC *Gastrointestinal disorders*
SOC *Hepatobiliary disorders*
SOC *Skin and subcutaneous tissue disorders*
SOC *Musculoskeletal and connective tissue disorders*
SOC *Renal and urinary disorders*
SOC *Pregnancy, puerperium and perinatal conditions*
SOC *Reproductive system and breast disorders*
SOC *Congenital, familial and genetic disorders*
SOC *General disorders and administration site conditions*
SOC *Investigations*
SOC *Injury, poisoning and procedural complications*
SOC *Surgical and medical procedures*
SOC *Social circumstances*
SOC *Product issues*

Medicago/Cytel Confidential: This is a controlled document and shall not be changed, saved, copied or reproduced by any unauthorized person.
