# Supplementary material for: Robust induction of functional humoral response by a plant-derived Coronavirus-like particle vaccine candidate for COVID-19
Source: NPJ Vaccines. 2023 Feb 13;8:13. doi: 10.1038/s41541-023-00612-2 (PMC9924894; doi:10.1038/s41541-023-00612-2)
Supplement: Supplementary file 2 — REPORTING SUMMARY [file 41541_2023_612_MOESM2_ESM.pdf]

# nature research

Corresponding author(s): Galit Alter

Last updated by author(s): November 8th, 2022

# Reporting Summary

Nature Research wishes to improve the reproducibility of the work that we publish. This form provides structure for consistency and transparency in reporting. For further information on Nature Research policies, see our Editorial Policies and the Editorial Policy Checklist.

## Statistics

For all statistical analyses, confirm that the following items are present in the figure legend, table legend, main text, or Methods section.

| n/a | Confirmed | |
|---|---|---|
| ☐ | ☒ | The exact sample size (*n*) for each experimental group/condition, given as a discrete number and unit of measurement |
| ☐ | ☒ | A statement on whether measurements were taken from distinct samples or whether the same sample was measured repeatedly |
| ☐ | ☒ | The statistical test(s) used AND whether they are one- or two-sided<br>*Only common tests should be described solely by name; describe more complex techniques in the Methods section.* |
| ☐ | ☒ | A description of all covariates tested |
| ☐ | ☒ | A description of any assumptions or corrections, such as tests of normality and adjustment for multiple comparisons |
| ☐ | ☒ | A full description of the statistical parameters including central tendency (e.g. means) or other basic estimates (e.g. regression coefficient) AND variation (e.g. standard deviation) or associated estimates of uncertainty (e.g. confidence intervals) |
| ☐ | ☒ | For null hypothesis testing, the test statistic (e.g. *F*, *t*, *r*) with confidence intervals, effect sizes, degrees of freedom and *P* value noted<br>*Give P values as exact values whenever suitable.* |
| ☒ | ☐ | For Bayesian analysis, information on the choice of priors and Markov chain Monte Carlo settings |
| ☒ | ☐ | For hierarchical and complex designs, identification of the appropriate level for tests and full reporting of outcomes |
| ☒ | ☐ | Estimates of effect sizes (e.g. Cohen's *d*, Pearson's *r*), indicating how they were calculated |

*Our web collection on statistics for biologists contains articles on many of the points above.*

## Software and code

Policy information about availability of computer code

| | |
|---|---|
| Data collection | ForeCyt® Standard Edition 8.1 was used to collect Luminex, ADNP, ADCP and ADCD assay.<br>Antibody glycosylation profiles were analyzed on a 3500xL genetic analyzer (Applied Biosystems) capillary electrophoresis instrument. |
| Data analysis | Microsoft Excel 365 was used to compile experimental data and patient information.<br>Data analysis was performed using R version 4.0.2 (2020-06-22).<br>Antibody Fc-glycan profiles were analyzed using ThermoFisher Glycan Assure Analysis software and plotted in GraphPad Prism Version 9.3.1 |

For manuscripts utilizing custom algorithms or software that are central to the research but not yet described in published literature, software must be made available to editors and reviewers. We strongly encourage code deposition in a community repository (e.g. GitHub). See the Nature Research guidelines for submitting code & software for further information.

## Data

Policy information about availability of data

All manuscripts must include a data availability statement. This statement should provide the following information, where applicable:

- Accession codes, unique identifiers, or web links for publicly available datasets
- A list of figures that have associated raw data
- A description of any restrictions on data availability

All relevant data are included in this manuscript. Source data are provided with this paper.

# Field-specific reporting

Please select the one below that is the best fit for your research. If you are not sure, read the appropriate sections before making your selection.

☒ Life sciences ☐ Behavioural & social sciences ☐ Ecological, evolutionary & environmental sciences

For a reference copy of the document with all sections, see nature.com/documents/nr-reporting-summary-flat.pdf

# Life sciences study design

All studies must disclose on these points even when the disclosure is negative.

| | |
|---|---|
| Sample size | No sample size calculation was performed. We used all samples received from the multi-site, double-blind, randomized, placebo-controlled trial, co-ordinated in South Africa at WITS-VIDA. |
| Data exclusions | The main exclusion criteria were human immunodeficiency virus (HIV) positivity at screening, previous or current laboratory-confirmed Covid-19, a history of anaphylaxis in relation to vaccination, and morbid obesity (body-mass index ≥40). |
| Replication | All experiments were run in duplicates and Luminex and functional assays (ADCD, ADNP and ADCD) repeated for most of the samples. Results between repeats were comparable. |
| Randomization | Samples were randomly distributed in 96 well plates |
| Blinding | Investigators were blinded during data collection. Group allocation had to be revealed to perform the data analysis. |

# Reporting for specific materials, systems and methods

We require information from authors about some types of materials, experimental systems and methods used in many studies. Here, indicate whether each material, system or method listed is relevant to your study. If you are not sure if a list item applies to your research, read the appropriate section before selecting a response.

## Materials & experimental systems

| n/a | Involved in the study | |
|---|---|---|
| ☐ | ☒ | Antibodies |
| ☐ | ☒ | Eukaryotic cell lines |
| ☒ | ☐ | Palaeontology and archaeology |
| ☒ | ☐ | Animals and other organisms |
| ☐ | ☒ | Human research participants |
| ☐ | ☒ | Clinical data |
| ☒ | ☐ | Dual use research of concern |

## Methods

| n/a | Involved in the study | |
|---|---|---|
| ☒ | ☐ | ChIP-seq |
| ☐ | ☒ | Flow cytometry |
| ☒ | ☐ | MRI-based neuroimaging |

## Antibodies

| | |
|---|---|
| Antibodies used | 1 .Mouse Anti-Human IgG1-PE (Southern-Biotech, #9054-09, clone:HP6001)<br>2. Mouse Anti-Human IgG2-PE (Southern-Biotech, #9060-09, clone:31-7-4)<br>3. Mouse Anti-Human IgG3-PE (Southern-Biotech, #9210-09, clone:HP6050)<br>4. Mouse Anti-Human IgG4-PE (Southern-Biotech, #9200-09, clone:HP6025)<br>5. Mouse Anti-Human IgM-PE (Southern-Biotech, #9020-09, clone:SA-DA4)<br>6. Mouse Anti-Human IgA1-PE (Southern-Biotech, #9130-09, clone: B3506B4)<br>7. Anti-human CD11b BV605 (BD, #562721, Clone ICRF44)<br>8. Anti-guinea pig complement C3 goat IgG fraction (MP Biomedical, #855385. polyclonal)<br>9. anti-human CD66b Pacific Blue (Biolegend, #305112 ,clone G10F5)<br>10. AntiCD107a– phycoerythrin (PE) – Cy5 (BD, #555798; clone: H4A3),<br>11. Anti-CD56 PE-Cy7 (BD, #557747, clone: B159)<br>12. Anti-CD16 APC-Cy5 (BD,#555408, clone: 3G8)<br>13. Anti- CD3 PacBlue (BD, #558117, UCHT1) |
| Validation | All antibodies are well established and quality controlled by the manufacturer. Additional information and references can be obtained on the company websites.<br>The use of antibodies 1-4 was previously validated: Brown EP, Licht AF, Dugast AS, Choi I, Bailey-Kellogg C, Alter G, et al. High-throughput, multiplexed IgG subclassing of antigen-specific antibodies from clinical samples. J Immunol Methods. 2012;386(1-2):117-23.<br>Antibody 8 was described here: Fischinger, S., J. K. Fallon, A. R. Michell, T. Broge, T. J. Suscovich, H. Streeck, and G. Alter. 2019. 'A high-throughput, bead-based, antigen-specific assay to assess the ability of antibodies to induce complement activation', J Immunol Methods, 473: 112630. |

Antibody 9: Karsten, C. B., N. Mehta, S. A. Shin, T. J. Diefenbach, M. D. Slein, W. Karpinski, E. B. Irvine, T. Broge, T. J. Suscovich, and G. Alter. 2019. 'A versatile high-throughput assay to characterize antibody-mediated neutrophil phagocytosis', J Immunol Methods, 471: 46-56.
The use of antibodies 10-13 was described here: M. F. Jennewein, I. Goldfarb, S. Dolatshahi, C. Cosgrove, F. J. Noelette, M. Krykbaeva, J. Das, A. Sarkar, M. J. Gorman, S. Fischinger, C. M. Boudreau, J. Brown, J. H. Cooperrider, J. Aneja, T. J. Suscovich, B. S. Graham, G. M. Lauer, T. Goetghebuer, A. Marchant, D. Lauffenburger, A. Y. Kim, L. E. Riley, G. Alter, Fc Glycan-Mediated Regulation of Placental Antibody Transfer. Cell 178, 202-215.e214 (2019).

## Eukaryotic cell lines

Policy information about cell lines

Cell line source(s): THP-1 cells (human acute monocytic leukemia)

Authentication: None of the cell lines used were authenticated.

Mycoplasma contamination: Cell lines were not tested for mycoplasma contamination

Commonly misidentified lines (See ICLAC register): No commonly misidentified lines were used.

## Human research participants

Policy information about studies involving human research participants

Population characteristics: This phase 1 randomized controlled trial (NCT04450004) was conducted at two sites in Quebec City (Syneos Health Clinique Inc.) and Montreal (Syneos Health Clinique Inc.) as previously described (Ward, B. J. et al. Phase 1 randomized trial of a plant-derived virus-like particle vaccine for COVID-19. Nature Medicine 27, 1071-1078, doi:10.1038/s41591-021-01370-1 (2021)). The study was approved by a central research ethics review board as well as the Health Products and Food Branch of Health Canada and was carried out in accordance with the Declaration of Helsinki and the principles of Good Clinical Practices. Healthy seronegative participants 18–55 years of age were randomized into groups in a 1:1 ratio and described in the manuscript

Recruitment: Participants were recruited from existing databases of volunteers, and written informed consent was obtained from all study participants. The health status was assessed by medical history, physical examination, and clinical laboratory findings, including detection of anti-N antibodies to SARS-CoV-2 (Elecsys, Roche Diagnostics).

Ethics oversight: The trial (Clinicaltrials.gov number: NCT04450004) was reviewed and approved. All participants were fully informed about the procedures and the possible risks, and all signed written informed consent documents.

Note that full information on the approval of the study protocol must also be provided in the manuscript.

## Clinical data

Policy information about clinical studies

All manuscripts should comply with the ICMJE guidelines for publication of clinical research and a completed CONSORT checklist must be included with all submissions.

Clinical trial registration: Clinicaltrials.gov number: NCT04450004

Study protocol: The full protocol is provided

Data collection: *Describe the settings and locales of data collection, noting the time periods of recruitment and data collection.*

Outcomes: All of the outcomes of the study are fully described in the Protocol (provided) and the primary outcomes are highlighted in the manuscript as submitted

## Flow Cytometry

### Plots

Confirm that:

- [x] The axis labels state the marker and fluorochrome used (e.g. CD4-FITC).
- [x] The axis scales are clearly visible. Include numbers along axes only for bottom left plot of group (a 'group' is an analysis of identical markers).
- [x] All plots are contour plots with outliers or pseudocolor plots.
- [x] A numerical value for number of cells or percentage (with statistics) is provided.

### Methodology

Sample preparation: For Luminex: Antigens were coupled to magnetic Luminex beads (Luminex Corp) by carbodiimide-NHS ester-coupling

| | |
|---|---|
| Sample preparation | (Thermo Fisher). Antigen-coupled microspheres were washed and incubated with plasma samples at an appropriate sample dilution (1:500 for IgG1 and all low-affinity Fcγ- receptors and 1:100 for all other readouts) for 2 hours at 37°C in 384-well plates (Greiner Bio-One). Unbound antibodies were washed away, and antigen-bound antibodies were detected by using a PE-coupled detection antibody for each subclass and isotype (IgG1, IgG3, IgA1, and IgM; Southern Biotech), and Fcγ-receptors were fluorescently labeled with PE before addition to immune complexes (FcγR2a, FcγR3a; Duke Protein Production facility). After one hour of incubation, plates were washed, and flow cytometry was performed with an IQue (Intellicyt)<br><br>Functional assyas: SARS-CoV-2 Spike proteins were coupled to yellow/green (505/515) or red/orange (565/580) fluorescent Neutravidin-conjugated beads (Thermo Fisher) for ADCP/ADNP and ADCD, respectively. Immune complexes were formed by incubating the diluted pooled samples (ADCP and ADNP 1:100 dilution) with the antigen-coupled beads for two h at 37  C.<br><br>Fc glycosylation analysis<br>Carboxy magnetic beads (Cytiva) were coated with WT SAR-CoV-2 Spike protein by carbodiimide-NHS ester-coupling method. Spike-specific antibodies were isolated from serum samples by incubating 25µL of serum with 25µL of antigen-coupled beads overnight at 4°C. Excess protein was washed off the beads with NEB buffer. |
| Instrument | IntelliCyt® iQue Screener PLUS; 3500xL genetic analyzer (Applied Biosystems) capillary electrophoresis instrument. |
| Software | ForeCyt® Standard Edition 8.1 was used to collect and analyze the data.<br>ThermoFisher Glycan Assure Analysis software. |
| Cell population abundance | Primary neutrophils were identified by CD66b surface expression. CD66b expression was donor dependent bu usually >95% within the single cell gate.<br>All single cells were considered THP-1 cells.<br>NK cells identified as CD3-, CD16+CD56+ |
| Gating strategy | Figure with gating strategy attached |

☒ Tick this box to confirm that a figure exemplifying the gating strategy is provided in the Supplementary Information.
